# Massively parallel pyrosequencing-based transcriptome analyses of small brown planthopper (*Laodelphax striatellus*), a vector insect transmitting rice stripe virus (RSV)

**DOI:** 10.1186/1471-2164-11-303

**Published:** 2010-05-13

**Authors:** Fujie Zhang, Hongyan Guo, Huajun Zheng, Tong Zhou, Yijun Zhou, Shengyue Wang, Rongxiang Fang, Wei Qian, Xiaoying Chen

**Affiliations:** 1 State Key Laboratory of Plant Genomics, Institute of Microbiology, Chinese Academy of Sciences, Beijing 100101, China; 2 National Plant Gene Research Center, Beijing 100101, China; 3 Graduate School of Chinese Academy of Sciences, Beijing 100039, China; 4 Shanghai-MOST Key Laboratory of Health and Disease Genomics, Chinese National Human Genome Center at Shanghai, Shanghai, 200032, China; 5 Jiangsu Academy of Agricultural Sciences, Nanjing 210014, China

## Abstract

**Background:**

The small brown planthopper (*Laodelphax striatellus*) is an important agricultural pest that not only damages rice plants by sap-sucking, but also acts as a vector that transmits rice stripe virus (RSV), which can cause even more serious yield loss. Despite being a model organism for studying entomology, population biology, plant protection, molecular interactions among plants, viruses and insects, only a few genomic sequences are available for this species. To investigate its transcriptome and determine the differences between viruliferous and naïve *L. striatellus*, we employed 454-FLX high-throughput pyrosequencing to generate EST databases of this insect.

**Results:**

We obtained 201,281 and 218,681 high-quality reads from viruliferous and naïve *L. striatellus*, respectively, with an average read length as 230 bp. These reads were assembled into contigs and two EST databases were generated. When all reads were combined, 16,885 contigs and 24,607 singletons (a total of 41,492 unigenes) were obtained, which represents a transcriptome of the insect. BlastX search against the NCBI-NR database revealed that only 6,873 (16.6%) of these unigenes have significant matches. Comparison of the distribution of GO classification among viruliferous, naïve, and combined EST databases indicated that these libraries are broadly representative of the *L. striatellus *transcriptomes. Functionally diverse transcripts from RSV, endosymbiotic bacteria *Wolbachia *and yeast-like symbiotes were identified, which reflects the possible lifestyles of these microbial symbionts that live in the cells of the host insect. Comparative genomic analysis revealed that *L. striatellus *encodes similar innate immunity regulatory systems as other insects, such as RNA interference, JAK/STAT and partial Imd cascades, which might be involved in defense against viral infection. In addition, we determined the differences in gene expression between vector and naïve samples, which generated a list of candidate genes that are potentially involved in the symbiosis of *L. striatellus *and RSV.

**Conclusions:**

To our knowledge, the present study is the first description of a genomic project for *L. striatellus*. The identification of transcripts from RSV, *Wolbachia*, yeast-like symbiotes and genes abundantly expressed in viruliferous insect, provided a starting-point for investigating the molecular basis of symbiosis among these organisms.

## Background

Most insects act as vectors for the transmission of viruses and are thus one of the most important factors in the study of the epidemiology and molecular pathology of plant or animal virus diseases. In the plant kingdom, it has been estimated that as much as 76% of viral diseases are transmitted by insects [1,2]. As the insects feed, insect-borne viruses easily penetrate the impermeable cuticle that covers the plant epidermis and are directly delivered into tissues or the cytoplasm. Insects belonging to Hemiptera, such as planthoppers, aphids, leafhoppers and whiteflies, have distinct piercing-sucking mouthparts that include needle-like stylet bundles and it is not surprising that Hemipteran insects transmit the majority (>55%) of the vectored plant viruses [1]. The mechanisms of virus transmission are diverse. Based on differences in the length of the time that insects can harbor infectious viral particles, the transmission mechanisms can be classified into nonpersistent (harboring viruses for a few seconds/minutes), semipersistent (a few hours/days, but lost upon molting), and persistent (often throughout the lifespan of the vectors) [3]. Taking advantage of insect transmission, viruses can spread among plant individuals, usually resulting in epidemic outbreaks of viral disease and severe agricultural yield loss. Therefore, understanding the biology of vector insects and the viral transmission mechanisms will provide insight into interactions among insects, viruses, and plants, which will promote the development of effective techniques to prevent viral diseases of plants.

The small brown planthopper (*Laodelphax striatellus *Fallén), which was previously deposited in the deprecated, paraphyletic order Homoptera, is a notorious rice pest that is now classified into the Delphacidae of Hemiptera [4]. Besides injuring rice plants (*Oryza sativa*) by sap-sucking with its piercing-sucking mouthparts, *L. striatellus *also acts as the most important vector of rice stripe virus (RSV, belonging to Tenuivirus) in a persistent and propagative manner [5]. After invading *L. striatellus*, RSV can escape from the midgut, salivary, and ovary barriers and propagate in *L. striatellus*. Evidences have revealed that amorphous or filamentous inclusions of RSV exist in the cytoplasm of midgut epithelial cells, salivary glands, and the fat body. Moreover, it has been confirmed that RSV particles exist in follicular cells of the ovarioles and can be transmitted from female adults to their progeny via eggs [6]. Concomitant with the changes in global climate and agricultural systems, the damage caused by *L. striatellus *and RSV increased dramatically after 1960s. For example, in Jiangsu Province, one of the major grain producing areas in China, rice fields suffering from *L. striatellus *and RSV reached 1.57 million hectares in recent years, accounting for about 80% of the rice fields and causing 30 - 40% yield loss in that area [7]. In practice, controlling the outbreak of populations of *L. striatellus *is the most effective way to prevent RSV infection. As a consequence, *L. striatellus *has long been used as a model organism in the study of insect ecology, physiology, biochemistry, as well as molecular interactions between vectors and plant viruses.

Although *L. striatellus *is important in both theoretical and applied studies, genomic information for this insect is quite limited. For example, to date, there is no whole-genome sequencing project for this species, and only a few DNA sequences are available in the public databases (< 100 entries). To collect genomic data efficiently, identification of expressed sequence tags (ESTs) that represent the transcribed genes from a given set of tissues or individuals is a cost-effective approach. We employed massively parallel pyrosequencing on the Roche 454-GS-FLX platform to create EST databases of *L. striatellus*. In total we identified 41,492 different unigenes from both viruliferous and naïve *L. striatellus*. Gene Ontology (GO) classification indicated they were involved in various biological processes. Within these transcripts, we identified 1,451 potential microsatellite loci that can be used as DNA markers. Transcripts from symbiotic microorganisms, including RSV, *Wolbachia *and yeast-like symbiotes, were identified, and functional annotation revealed the possible lifestyle of these microorganisms within the insect cells. Comparison of our EST database with known insect immune systems showed that *L. striatellus *encodes similar immune regulatory systems, such as RNA interference, and JAK/STAT and Imd pathways that might be involved in resistance to viral infection [8-10]. In addition, comparative analysis of the two transcriptomes generated from viruliferous (RSV-infected) and naïve (non-infected) *L. striatellus *provided a list of candidate transcripts that potentially represent the biological response to viral infection. To our knowledge, this work is the first to study the transcriptome of *L. striatellus*. The transcriptional information provided will have an immediate effect on gene cloning, annotation, and functional studies of insect-plant-virus symbiosis.

## Results and Discussion

### Pyrosequencing and contig assembly of ESTs

As described in the Methods, viruliferous and naïve *L. striatellus *whole body cDNA libraries were subjected to a full-plate production run on the 454-GS-FLX sequencing instrument, resulting in 207,918 and 225,158 reads, respectively. The length distribution of these reads is depicted in Additional file 1. After filtering for adaptors and low-quality sequences, the two libraries generated 201,281 (viruliferous sample) and 218,681 reads (naïve sample), respectively, with a total of 96,567,749 bases (Table 1). Files containing these data were deposited in the Short Read Archive of the National Center for Biotechnology Information (NCBI) with accession numbers of SRX016333 (viruliferous) and SRX016334 (naïve), respectively. As shown in Table 1, the general features of the contig assembly from the two libraries are similar: a total of 9,936 and 9,417 contigs were obtained from the viruliferous and naïve datasets, with average lengths of 376 bps and 362 bps, respectively. When sequence data from the two libraries were combined, the average length of the high quality reads was 229 bp. These combined reads were assembled into 16,885 contigs with an average length of 384 bp (ranging from 61 to 8,651 bp). In addition, there were 24,607 reads that failed to be covered by contigs and were treated as singletons. In total, 41,492 unigenes were obtained from the combined EST library. All files of assembled contigs and singletons from viruliferous, naïve, and combined EST libraries are available by request.

**Table 1:** General features of *L. striatellus *EST libraries

	Viruliferous sample	Naïve sample	Combined sample
Total bases (bp)	44741850	51825899	96567749
High-quality reads^a^	201281	218681	419962
Average reads length (bp)	222	237	230
Number of contigs	9936	9417	16885
Average contig length (bp)	376	362	384
Range of contig length (bp)	60~3752	61~4182	61~8651
Number of reads in contigs	174662	165212	383067
Number of singletons	14331	14117	24607
Unigenes (contigs + singletons)	24267	23534	41492
Matched CDSs ^b^	22664	22447	38019
Specific sequences	15746	14318	-

a. Adaptors and low-quality reads were excluded.

b. Matched CDS means the number of predicted protein coding sequences from unigenes.

### Similarity searches and gene ontology analysis

**Figure 1:**
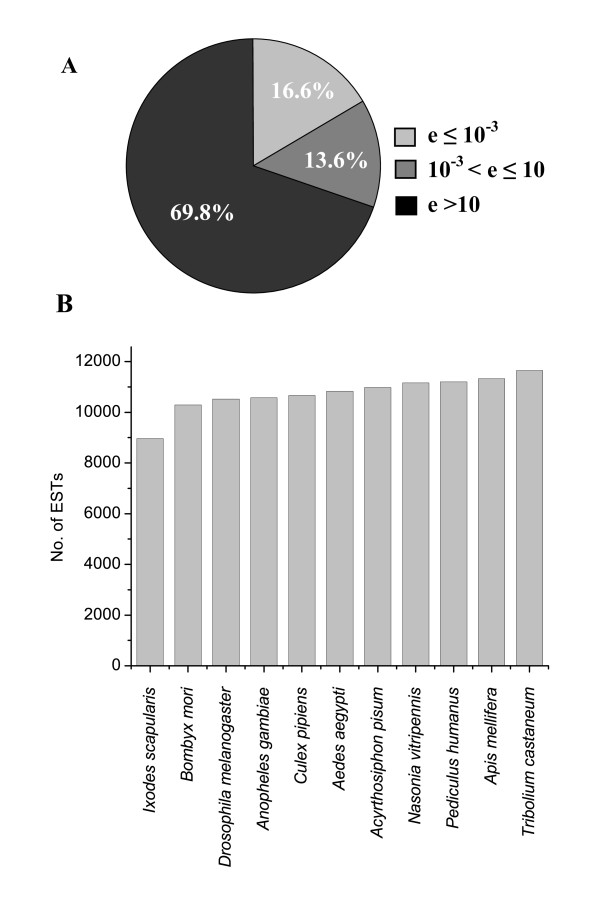
**BlastX similarity search of the combined *L. striatellus *EST library**. (A) Distribution of matched sequences by BlastX search against the NCBI-NR database. Significant matched sequences were defined as e-value ≤ 10^-3^. (B) Similarity of *L. striatellus *ESTs compared with putative proteomes of other arthropods. The cutoff e-value was set as ≤ 10^-3^.

ESTs were subjected to BlastX similarity search against the NCBI-NR protein database to determine their putative function. As shown in Fig. 1A, among the 41,492 unique sequences that contain ESTs from both viruliferous and naïve *L. striatellus*, 6,873 (16.6%) showed significant matches at the cutoff e-value ≤ 10^-3^, whereas 5,659 (13.6%) showed poor matches with e-values between 10^-3 ^and 10. The remaining 28,960 (69.8%) ESTs exhibited no useful matches (e-value > 10). As a result, we estimated that the majority of the genes of *L. striatellus *(> 80%) are function-unknown. To compare the similarity of *L. striatellus *genes with those of the other arthropods, EST sequences were also subjected to BlastX search against putative protein sequences (e-value ≤ 10^-3^) of eleven species of arthropod whose complete or draft genomes are available. As shown in Fig. 1B, the smallest number of database matches (8,959; 21.6%) was from deer tick (*Ixodes scapularis*). This can be explained by the fact that *I. scapularis *belongs to Arachnida and is not a species of Insecta. The other species belong to Insecta are phylogenetically distributed into Phthiraptera (*Pediculus humanus*), Coleoptera (*Tribolium castaneum*), Lepidoptera (*Bombyx mori*), Hymenoptera (*Apis mellifera *and *Nasonia vitripennis*), Diptera (*Drosophila melanogaster*, *Anopheles gambiae*, *Culex pipiens *and *Aedes aegypti*), and Hemiptera (*Acyrthosiphon pisum*); however, the numbers of matched sequences are very similar (from 10,290 - 11,646). Unexpectedly, although pea aphid (*A. pisum*) belongs to Hemiptera, as does *L. striatellus*, and currently its draft genome has the largest set of putative protein coding sequences (37,994), only 10,979 (26.5%) ESTs of *L. striatellus *were found to be similar to the proteome of *A. pisum*. This number is smaller than that of parasitoid of flies (*N. vitripennis*, 11,162; 26.9%), head louse (*P. humanus*, 11,203; 27.0%), honey bee (*A. mellifera*, 11,321; 27.3%), and red flour beetle (*T. castaneum*, 11,646; 28.1%). The number of matched sequences does not entirely reflect the phylogenetic relationships and genome sizes. This discordance might be simply attributed to the reason that our understanding of the genomes of insect is relatively inadequate.

**Figure 2:**
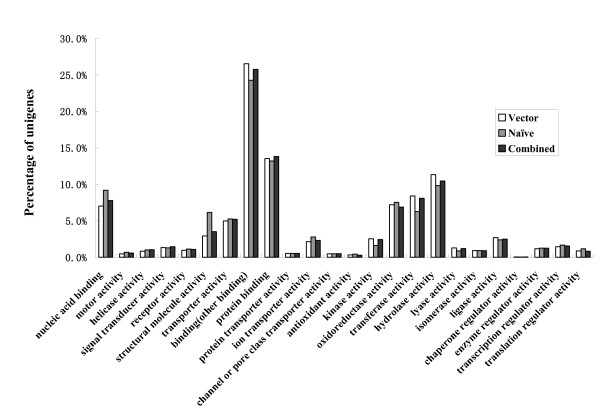
**Comparison of the distribution of GO terms**. The x-axis shows subgroups of molecular functions from GO, the Y-axis shows the percentage of the matched EST sequences. Distribution of GO terms of ESTs from viruliferous (vector), naïve, and combined samples are compared.

To assess whether our EST libraries were substantial representative samples of the transcriptome of *L. striatellus*, the search results of BlastX against the Swiss-Prot/TrEMBL database were combined, and matches were assigned GO (gene ontology) categories with biological process, molecular function, and cellular components [11], respectively. Fig. 2 depicts the results of GO classification of molecular function and reveals that, for EST libraries of viruliferous, naïve, and combined samples, the percentages of transcripts in each GO category are quite similar. This is also true for those ESTs classified as biological process as well as cellular components. These results suggested that our EST libraries sampled widely across the GO sub-categories and provided a good representation of the *L. striatellus *transcriptome.

The coverage of the combined EST library of *L. striatellus *was assessed by two analyses. Firstly, 366 contigs and singletons of this library are found to encode possible ribosomal proteins. We then compared these sequences by BlastX search against a database of the ribosomal proteins of *D. melanogaster *(e-value < 10^-3^), which contains 88 cytoplasmic ribosomal proteins and 79 mitochondrial ribosomal proteins. Except four ribosomal proteins of *D. melanogaster *(ribosomal protein L41, mitochondrial ribosomal protein S5, S31 and L51), the others have significantly matched orthologs in the EST library of *L. striatellus*. The second analysis used the 41,492 unigenes of *L. striatellus *to search against recently reported protein sequences of its close-relative pea aphid (OGS 1.0 of *A. pisium*, including 34,821 sequences from NCBI RefSeq plus non redundant GLEAN) [12]. A total of 12,361 non-redundant protein sequences of *A. pisium *exhibited significant similarity to the sequences from *L. striatellus *(BlastX, e-value < 10^-3^). The remaining unigenes that did not show significant matches to *A. pisum*, were used to search again against the protein sequences of *D. melanogaster *(containing 21,603 sequences). An extra 1,719 hits were obtained, suggesting that the combined transcriptome of *L. striatellus *has at least 14,080 protein coding genes (12,361 from *A. pisium *plus 1,719 from *D. melanogaster*). We suppose that the remaining ~20,000 unigenes which did not return significant BlastX hit may encode previously uncharacterized proteins, non-coding RNAs, or products from microbial endosymbionts.

### Identification of microsatellites

Microsatellites (or simple sequence repeats, SSRs) are hyper-polymorphic DNA fragments that contain repeating units of 1 - 6 base pairs [13]. Although they are widely used as molecular markers, no microsatellite sequences have been reported for *L. striatellus*. We identified many microsatellite loci with di-, tri-, tetra-, penta- and hexanucleotide repeats (minimum repeats > 6) from the combined EST library using SciRoKo ver 3.4 software [14]. Table 2 shows that, when perfect repeat motifs were considered, a total of 423 microsatellite markers were identified (ranging from 15 - 128 bps) and the mean microsatellite density is one per 27.7 kb. Among these molecular markers, tri-nucleotide repeats are predominant (76.6%), with (AAC)n being the most frequent motif (32.4%). When a conserved degree of base-pair mismatch (≤ 2 bps) was applied in the repeat motif search, a total of 1,451 microsatellite loci were identified (ranging from 15 - 132 bps) and the mean microsatellite density was one per 8.07 kb. Of them, only 564 (38.9%) were found in protein coding transcripts, including those annotated as hypothetical and conserved hypothetical proteins. Similar to the perfect repeats, tri-nucleotide repeats are also predominant (56.2%) and the motif (AAC)n (18.7%) is the most frequent, followed by (AAG)n (8.2%), (AAT)n (7.7%), (AGC)n (6.3%) and (AGG)n (5.1%). Among all possible di-nucleotide microsatellites [(CA)n, (GA)n, (AT)n and (GC)n], no perfect or imperfect (GC)n sequence repeat was found in EST library of *L. striatellus*. These identified microsatellites have the potential to be used in genetic mapping, parentage analysis, genotyping, gene flow, and in population genetics.

**Table 2:** Statistics of microsatellite loci derived from the EST library

		Perfect repeats				Imperfect repeats ^a^		
**Repeat size**	**No**.	**Average length (bp)**	**GC content**	**No./Mb**^b^	**No**.	**Average length (bp)**	**GC content**	**No./Mb**^b^

2	76	18.7 ± 5.07	0.43	6.49	88	21.9 ± 7.74	0.43	7.51
3	324	24.0 ± 10.31	0.39	27.66	816	22.1 ± 10.48	0.40	69.66
4	14	32.1 ± 11.51	0.20	1.20	169	20.2 ± 9.51	0.17	14.43
5	3	32.0 ± 2.83	0.06	0.26	242	17.7 ± 4.01	0.32	20.66
6	6	57.2 ± 23.07	0.37	0.51	136	30.4 ± 21.62	0.48	11.61

a. The conservative degree of mismatch of repeats was set as ≤ 2 bp.

b. No./Mb represents the average number of microsatellites per 1 Mb of EST sequence the library.

### Identification of transcripts of endosymbiotic microbes

#### Rice stripe virus (RSV)

**Figure 3:**
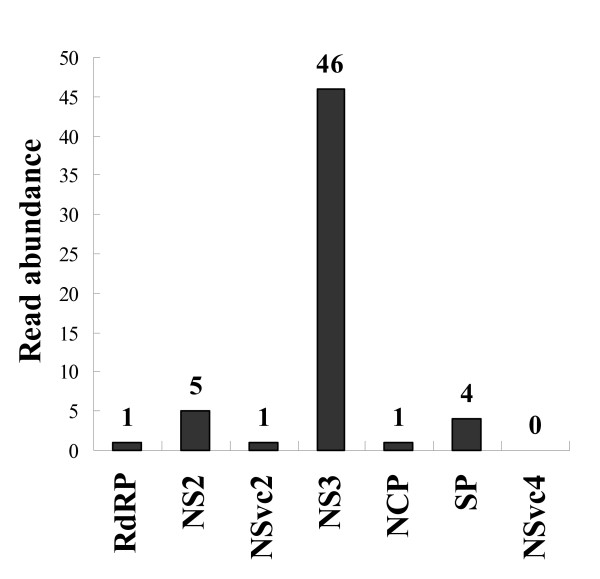
**Abundances of transcripts of RSV ORFs**. RdRP: RNA dependent RNA polymerase; NS2: function-unknown protein; NSvc2: putative glycoprotein; NS3: RNAi suppressor; NCP: nucleocapsid; SP: disease-specific protein; NSvc4: movement protein. The Y-axis shows the number of reads identified in the viruliferous EST library.

RSV is the type member of the genus *Tenuivirus *that has an unusual thread-like morphology under the electron microscope [5]. The RSV genome contains four segmented single-stranded RNAs (ssRNAs), which encode seven open reading frames (ORFs). Among them, RNA1 encodes a part of the RNA-dependent RNA polymerase (RdRP)[15]. RNA2, RNA3, and RNA4 are ambisense. Each of them has two ORFs: one located on the 5' part of the viral RNA and the other on the 5' part of the viral cRNA. RNA2 encodes a function-unknown protein (NS2) and a putative membrane glycoprotein, NSvc2. RNA3 encodes a suppressor protein (NS3) and a nucleocapsid, NCP. RNA4 encodes a nonstructural disease-specific protein (SP) and a movement protein, NSvc4 [5,16]. To investigate the expression level of these ORFs in *L. striatellus*, each RSV ORF sequence was searched against our EST libraries. No RSV transcript was found in the ESTs of naïve insects, confirming the naivety of this sample. For ESTs of the viruliferous insect, the abundance of RSV transcripts is shown in Fig. 3. NS3 is the most abundant transcript (46 reads in total) and is a 23 kDa protein that was experimentally demonstrated as a suppressor of gene silencing because it can significantly reduce the accumulation of small interference RNA (siRNA) in plant cells, and can bind 21-nucleotide single-strand (ss) siRNA, siRNA duplexes, and long ssRNA [17]. The dominant presence of the NS3 transcript suggests that after invading *L. striatellus*, RSV must suppress the immune response of the host insect as well as the host plant. Therefore, it would be interesting to identify the host cellular target that is suppressed by RSV NS3 in a future study. The other transcripts, including RdRP, NS2, NSvc2, NCP, and SP, were present in very low levels in the EST library of *L. striatellus *(matched reads from 1-5), suggesting that replication and assembly of RSV is not very active in the insect. In addition, we failed to identify transcripts of the movement protein NSvc4, which agrees with the existing hypothesis that the movement protein usually functions *in planta *during virus spreading to neighboring cells through the plasmodesmata [16], and thus its expression might be shut down or at a very low level in the insect.

#### Wolbachia

*Wolbachia *are obligatory, intracellular, Gram-negative bacteria that infect a wide range of arthropods and nematodes [18]. *Wolbachia *can enhance their maternal transmission by manipulating host reproductive systems through strategies such as cytoplasmic incompatibility, parthenogenesis, feminization, and male killing [7]. Interestingly, it has been recently discovered that this bacterial endosymbiont can reduce the RNA virus load in *D. melanogaster *and thus promote the antiviral potential of host insect [19,20]. In *L. striatellus*, *Wolbachia *infection is familiar and it was reported that the bacterium is distributed in the head, thorax, abdomen, salivary gland, guts, ovary, and testis [21]. To investigate whether our samples are infected by *Wolbachia *and to determine the gene expression pattern of the bacterium, we searched the EST libraries and annotated a total of 103 *Wolbachia *genes. Both viruliferous and naïve EST libraries contain transcripts of *Wolbachia*, and the number of matched reads in the naïve sample is four times larger than that of the viruliferous sample (528 vs. 132 reads). It is not clear whether this expression bias is associated with RSV infection.

The available sequenced genomes of *Wolbachia *encode approximately 1,000-1,400 proteins [22]; therefore, we estimated that the *Wolbachia *transcripts identified in our study represent about 10-13% of the transcriptome of the bacteria. Additional file 2 shows that the annotated *Wolbachia *genes can be classified into four major functional categories. (1) Function-unknown genes: this group contains 53 transcripts (51.5%) of *Wolbachia*, including the first three most abundant genes. (2) Genes essential for cell processes such as DNA and RNA synthesis, ribosomal assembly, and electronic transportation. (3) Genes responsible for *de novo *biosynthesis of nucleotides and cofactors, including purine, riboflavin, coenzyme A, and ubiquinone. Identification of these gene transcripts suggests that *Wolbachia *cannot obtain these chemicals from the host insect cells and have to synthesize them themselves, as predicted by genomic annotation [7]. (4) Gene products involved in transport, including an ABC transporter, proprotein translocase, permease, Na^+^/H^+ ^antiporter, and a set of chaperones (DnaK, GroES, and a cold shock protein). Among them, a gene encoding multidrug resistance protein D was identified. The product of this gene has an AcrF-like domain and belongs to the RND efflux pump family [23]. We hypothesize that the protein is responsible for pumping harmful host-derived chemicals out of the bacterial cell envelope to maintain homeostasis. Besides the above-mentioned genes, an ATP-dependent protease, Lon, was identified. In bacteria, this kind of protease degrades naturally unstable proteins and thus takes part in diverse cell processes. It has been reported that inactivation of Lon genes will attenuate the virulence of various pathogenic bacteria, such as *Salmonella enterica*, *Yersinia pestis*, and *Pseudomonas syringae *[24]. Based on this, we speculate that Lon of *Wolbachia *plays an important role in symbiosis with *L. striatellus*. Taken together, these identified *Wolbachia *genes may reveal facets of how an endosymbiotic bacterium adapts to the living environment within cells of host insects.

#### Yeast-like symbiotes (YLS)

The YLS are microbial, transovarially inherited endosymbiotes being found in the fat body of *Laodelphax striatellus*. Phylogenetic analysis has revealed that the YLS consists of fungal species from the ascomycetes and pyrenomycetes [25,26]. These endosymbiotes can benefit their hosts by providing nutrition such as vitamins and sterols, and loss of the YLS under high temperature can cause deleterious effect on the host insect [27]. In this study, although the whole bodies, rather than the fat bodies of insects, were used as samples to generate EST sequences, and the strategy constructing cDNA libraries (see Methods) has pre-excluded the large-scale contamination of 18S rRNA sequences that can be used to identify the species of YLS, close examination of the combined EST library retrieved 3,061 unigenes that matched well with the fungal species belonging to 45 genera (Additional file 3). Among them, 2,028 unigenes exhibit significantly matches (Blastx search, e-value < 10^-3^), and the majority of these unigenes (2,637, 86.1%) encode function-unknown proteins. For lacking of 18S rRNA sequences, it is hard to determine whether a unigene derives from the YLS. However, among the matched 45 fungal genera, 20 of them have been previously reported to be the YLS and their transcripts can provide useful information [25,26]. For example, in our library there are 44 unigenes matched well with *Aspergillus fumigatus*. Among them, seven genes encode products that may be involved in cell signaling, including two zinc finger proteins, two ankyrin-repeat domain containing proteins, a RING-finger protein and two proteins related to the ubiquitin-mediated protein degradation. Future functional studies will reveal whether they have association with the regulation of expression of the symbiosis-related genes.

### Putative immune regulatory proteins

One of the long-term goals in studying vector-virus symbiosis is to elucidate the molecular mechanisms by which viruses manipulate the host innate immune system to avoid injury from the defensive response. Therefore, understanding the host immune system is fundamental to answering this question. By comparing our EST database with known immune proteins encoded by other insects, especially *D. melanogaster*, putative regulatory pathways of *L. striatellus *immune system were annotated. Among them, we focused on antivirus related cascades, including RNAi silencing, JAK/STAT, and Imd pathways (Table 3).

**Table 3:** Putative immune regulatory genes of *L. striatellus *that might be involved in resistance to viral infection.

Regulatory protein	Function and description	Code of ESTs (e-value)
**RNAi systems**		
Dicer like protein	RNAIII family endo-ribonucleases that cleave double-stranded RNA and pre-microRNA into short dsRNA fragments.	Contig2793 (2e-27), Contig6128 (6e-06),
		FQ4QJ5301BI24S (6e-17),
		FQ92HJ001CXB6B (2e-13),
		FQ4QJ5301EGA00 (7e-09)
Argonaute like protein	Catalytic component of the RNA-induced silencing complex	Contig1164 (7e-24), Contig6509 (2e-18),
		Contig15043 (2e-21),
		FQ4QJ5301BLRSO (5e-38),
		FQ92HJ001AVX3O (4e-16),
		Q92HJ001B74KA (1e-10),
		FQ92HJ001DDNCB (1e-11)
R2D2	dsRNA-binding proteins	No match^b^
R3D1	dsRNA-binding proteins	FQ92HJ001EMI2C (1e-05)
**JAK/STAT pathway**		
UPD-like protein	Ligand activating JAK/STAT pathway	No match^b^
Domeless	Transmembrane cytokine receptor	FQ92HJ001EGTO6 (0.99)
Hopscotch	Janus Kinases (JAK) that have tyrosine kinase activity	Not determined ^c^
STAT	SH2-domain containing protein that can be phosphorylated by JAK	FQ4QJ5301ERDD2 (1e-15)
**Imd pathway**		
Imd	Death domain-containing protein that similar to receptor interacting protein (RIP) of TNF-R pathway	FQ92HJ001A68SH (0.031)
PGRP	Peptidoglycan recognition protein	Contig145 (6e-23), Contig6140 (1e-26),
		Contig6821 (3e-37)
Relish ^a^	NF-κB-like transcription factor	FQ4QJ5301E34NM (0.014)
MAP3K, TAK1	Mitogen-activated protein 3 kinase	Not determined ^c^
TAB2	TAK1 binding protein	FQ4QJ5301DF7ET (1e-18)
DIAP2	Inhibitor of apoptosis proteins	Contig3110 (1e-21)
IKKβ/ird5	Inhibitor of NF-κB kinase	Not determined ^c^
IKKγ/Kenny	Inhibitor of NF-κB kinase	Not determined ^c^
dFADD	Fas-associated death domain protein	FQ92HJ001A68SH (0.031)
Dredd caspase	A caspase involved in apoptosis	No match^b^

a. The protein Relish is usually regarded as a component of the NF-κB pathway.

b. No significant match (e ≤ 10^-3^) in searching EST libraries of *L. striatellus*.

c. This indicates that although there are significant matches, but it is hard to determine the preferred one since these matched proteins are highly similar in predicted secondary structure.

#### Putative RNA silencing related proteins

Currently RNA interference (RNAi) is the only mechanism that has been revealed to target viruses directly in insects [9,28]. The model insect *D. melanogaster *encodes two Dicers (Dicer-1 and Dicer-2), five Argonautes (AGO-1 ~AGO3, PIWI, and Aubergine), and two dsRNA-binding proteins (dsRBP, including R2D2 and R3D1) that are involved in RNAi. Among them, Dicer-2, AGO-2, and R2D2 are responsible for the antiviral response. After viral double-stranded RNAs (dsRNAs) are recognized, RNAse III enzyme Dicer-2 forms a complex with dsRBP R2D2, and then cleaves newly synthesized viral dsRNA to produce small interfering RNAs (siRNAs) that guide the AGO-2 containing RISC complex to specifically recognize and degrade viral RNA [29]. Among the ESTs of *L. striatellus*, five and seven contigs that might encode Dicer- and AGO-like proteins, respectively, were identified (Table 3). However, because Dicer and AGO proteins are similar in structure and our transcripts are not full-length, it is difficult to discriminate these Dicer- and AGO-like proteins into different sub-classes. In addition, we obtained an R3D1 homologous transcript (FQ92HJ001EMI2C) but failed to retrieve any R2D2 homologs from our database. Comparative analysis of EST abundance showed that there was no substantial difference in the levels of putative RNAi related genes between viruliferous and naïve samples of *L. striatellus*. This is in accordance with previous results showing that, even when challenged with pathogenic viruses, insect RNAi pathway genes are constitutively expressed and are not upregulated by viral infection [30,31]. Consequently, which RNAi process of *L. striatellus *is suppressed by RSV is an open question.

#### Putative JAK/STAT proteins

The JAK/STAT pathway consists of four major components: the ligand UPD, the receptor Domeless, the Janus kinase (JAK), and the signal transduction and activators of transcription (STAT) [9,32]. After receiving a specific signal, UPD binds to the fat body receptor Domeless and activates phosphorylation of the non-receptor tyrosine kinase JAK. In due course, STAT is phosphorylated by JAK to form dimmers and is recruited into the nucleus where it binds to palindromic region of promoters of downstream genes to activate their transcription [33]. In *D. melanogaster*, the JAK/STAT pathway is usually involved in differentiation of hemocytes and resistance to bacterial or fungal infection. However, evidences have accumulated that reveal that this pathway is also associated with the antiviral response, possibly through sensing cytokine signals and inducing antiviral response genes [30,34]. Using the above-mentioned proteins of *D. melanogaster *as search queries, we found that *L. striatellus *encodes homologs of JAK, Domeless, and STAT (Table 3), suggesting it contains a JAK/STAT cascade and that these matched proteins are conserved during insect evolution. However, no UPD transcript (UPD, UPD2, or UPD3) was found. This can be explained by that UPD transcripts are not present in our EST library or *L. striatellus *does not encode similar proteins. Considering that many other insects whose genomes have been sequenced (such as *A. gambiae *and *B. mori*) also lack UPD-like genes, we believe that the JAK/STAT pathway of *L. striatellus *might be activated by sensing ligands that differ from UPD of *D. melanogaster*.

#### Putative Imd pathway proteins

The Imd pathway regulates the expression of antimicrobial peptide (AMP) genes by perception of signals from pathogen-associated molecular patterns (PAMPs), such as the diaminopimelic acid (DAP)-type peptidoglycan of Gram-negative bacteria [35]. The Imd pathway can be activated through peptidoglycan recognition proteins PGRP-LC and PGRP-LE [36]. After receiving signals from PAMPs, these receptors drive the activation of a critical NF-κB-like transcription factor Relish (Rel) through a complicated signaling cascade that consists of IMD, dTAK1, dTAB2, IKKγ, IKKβ, ird5, and dFADD proteins to modulate AMP gene expression [37]. Studies revealed that mutations in several Imd pathway genes, including *PGRP-LC*, *Tak1*, *ird5*, *key*, and *rel *genes (but not *imd *and *dFADD*), resulted in high levels of susceptibility to viral infection in *D. melanogaster *[38,39]. Moreover, the mammalian tumor necrosis factor receptor (TNFR) pathway, which is the counterpart of the insect Imd pathway, has long been known to regulate the immune response to viral infections [40]. These results indicated that a branch of the Imd pathway is also required in defense against viruses. Interestingly, recent genomic annotation of pea aphid (*A. pisum*), a close relative of *L. striatellus *in phylogeny, revealed that this insect lacks the principal Imd, dFADD, Dredd, Relish and even PGRP proteins [41]. In the EST libraries of *L. striatellus*, homologs of Imd pathway gene transcripts, including Imd, TAB2, DIAP2, dFADD, and NF-κB like protein Relish, were identified, but it is noticeable that the homologs of Imd, Relish and dFADD have a relatively lower matched score (Table 3). In addition, the PGRP-like proteins are present in the EST libraries, which is different from that of the *A. pisum *(Table 3). Furthermore, although there are a number of matched targets that are similar to protein kinases TAK1, IKKγ, and IKKβ, it is hard to separate these transcripts from other kind of kinases because their predicted secondary structures (by pfam search, e < 0.1) are quite similar. In addition, we failed to identify a homolog of the Dredd caspase (Table 3). Taken the above information together, we suppose that the Imd pathway of *L. striatellus*, if it exists, is different from that of *D. melanogaster *and *A. pisum*. It will be interesting to determine whether this genetically differentiated immune pathway also has an association in defending RSV infection.

### Comparative analyses of EST libraries

There were 15,746 and 14,318 unigenes specifically identified in viruliferous and naïve EST databases, respectively (Table 1). However, due to the non-saturated nature of our EST databases and the relatively short average unigene length derived from pyrosequencing, these specific sequences cannot be a reliable representation of the transcriptional differences between the different samples. Therefore, we compared the differences in transcript abundance between viruliferous and naïve *L. striatellus*. Additional file 4 lists the top 30 contigs that are more abundant in the viruliferous sample. Although care must be taken in draw the conclusion that highly abundant ESTs play critical roles in symbiosis with RSV, the analysis provided a candidate gene list to further investigate the molecular basis of *L. striatellus *- RSV interactions.

It is interesting to note that transcripts encoding vitellogenin are the most abundant in the viruliferous insect sample (Additional file 4). We then retrieved all 34 unigenes of *L. striatellus *encoding vitellogenin and mapped them onto their ortholog (BAF75351.1) of brown planthopper (*Nilaparvata lugens*) by BlastX. The protein products of these unigenes have overlapped regions and a plenty of single nucleotide polymorphism were identified within these regions, indicating that *L. striatellus *contains multiple copies of vitellogenin genes. Vitellogenin is an egg yolk precursor for lipoproteins and phosphoproteins that is expressed in female insects. The protein is synthesized extraovarially in the fat body, transported through the hemolymph, and eventually internalized by competent oocytes through membrane-bound receptors named vitellogenin/lipophorin receptors [42]. Thus, it was suggested that the vitellogenin traffic pathway might be exploited by viruses to overcome the physical barrier between follicle cells and oocytes [43]. In fact, in *D. melanogaster*, it has been observed that when genes responsible for endosomal trafficking of vitellogenin from the follicle cells to the oocyte were mutated, the transport of retrovirus ZAM viral particles from follicle cells to oocytes was arrested. This result strongly supported the hypothesis that ZAM uses the vitellogenin traffic pathway to gain access to the oocyte to affect the maternal germ line [44]. Similarly, the extraordinary abundance of vitellogenin transcripts in viruliferous *L. striatellus *EST library suggested that RSV might modulate the host vitellogenin pathway genes to facilitate its persistent transmission via the insect ovum.

### Expression profiling by RT-PCR

**Figure 4:**
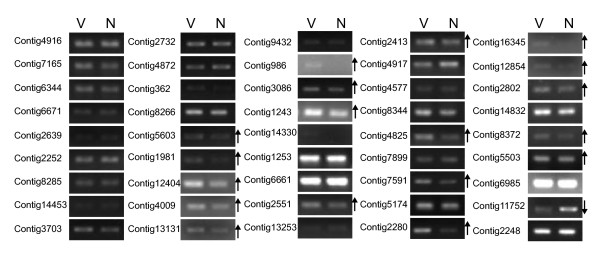
**RT-PCR profiles of putative transcripts from viruliferous and naïve *L. striatellus *samples**. V: viruliferous; N: naïve. The arrow next to the gel pictures indicates increased or decreased expression of a particular gene in viruliferous vs. naïve sample. At least two rounds of independent replication were used for each primer pair. The putative elongation factor gene (Contig2248) was used as a reference. The sequences of PCR primers used in this analysis are listed in Additional file 6.

Semi-quantitative RT-PCR analysis was used to confirm the expression of 52 selected contigs of *L. striatellus*. Most of these unigenes are selected because they exhibited high abundance in the EST library of viruliferous samples. A non-structural protein gene (Contig12404) that was supposed to be encoded by Himetobi P virus, another virus being reported to infect *L. striatellus*, was also included. The results of amplification showed that 45 of the transcripts (Additional file 5) are indeed expressed in both viruliferous and naïve *L. striatellus *(Fig. 4), demonstrating that they are not sequencing artifacts. This strongly supports the view that the parallel sequencing technique is a quick and highly effective approach for investigating the transcriptome of poorly-understand organisms.

Among successfully amplified contigs, 18 candidate genes showed reproducibly up-regulated expression patterns in viruliferous insects (Fig. 4), including genes encoding transferrin (Contig3086 and Contig6985), ribosomal protein L4 (Contig2551), ribosomal protein L15 (Contig5503), G-protein (Contig1243), malic enzyme (Contig7591), ATP-citrate synthase (Contig2280), and mitochondrial ATP synthase alpha subunit (Contig2413). The expression patterns are in accordance with the reads abundance of pyrosequencing, suggesting that these genes are indeed upregulated in viruliferous *L. striatellus*. Before in-depth functional study being carried out, how did these genes relate to symbiosis with RSV remains illusive. For example, it has been found that ribosomal protein S3 (RPS3) is a subunit of NF-κB transcriptional complexes in vertebrate. It forms a complex with NF-κB subunit p65 and promotes the selective affinity of NF-κB complexes for activating the expression of a group of immune-related genes [45]. This implies that ribosomal protein may be involved in regulating immune response by protein-DNA interactions. Our RT-PCR assay confirmed that two ribosomal large-subunit proteins, L4 and L15, were up-regulated in the viruliferous insects. The two proteins have quite different secondary structures compared with the RPS3 so that how they are involved in innate immunity system of *L. striatellus *will be an opening question.

Both Contig3086 and Contig6985 encode transferrins. Of them, there is no read of Contig6985 identified in the naïve insect samples whereas 300 reads were found in the viruliferous samples. Transferrins are glycoproteins that bind iron ion with high affinity. These proteins often take part in antibacterial response since they decrease the concentration of available iron ions in the animal tissues so that pathogenic bacteria can not grow and proliferate because the starvation of iron. However, excess cellular iron is also associated with increased infection rate for viral diseases such as acquired immune deficiency syndrome (AIDS) caused by human immunodeficiency virus (HIV) and hepatitis caused by hepatitis C virus (HCV) [46]. Viruses can affect the expression of host proteins involved in iron homeostasis and benefit from iron overload. For example, the HIV-1 Nef protein regulates the localization of HFE, a host nonclassical major histocompatibility complex (MHC) class I protein that binds to transferrin receptors. This process causes accumulation of iron in macrophages [47]. In addition, if the concentration of cellular iron is low, the activity of iron-dependent viral enzymes, such as ribonucleotide reductase, will be decreased and then through negative impact on viral replication [48]. Based on this, it will be interesting to determine the functional role of these transferrin genes during RSV infection and how they are up-regulated.

## Conclusions

Understanding the molecular interactions between *L. striatellus *and its symbiotic microbes is useful, not only for studies on the biology of species relationships, but also for agricultural practice that aims to develop effective strategies to prevent viral disease via controlling vector insects. However, these studies have been hampered by the lack of genomic resources of *L. striatellus*. Here, we employed the massively parallel pyrosequencing technique to collect ESTs from viruliferous and naïve samples of *L. striatellus*. When these ESTs were combined, 16,885 contigs and 24,607 singletons were generated, of which about 16.6% can be assigned a biological function. This genome-scale transcriptional information provides a basis for further investigation of the biology of *L. striatellus*. For example, we annotated a set of putative innate immunity regulatory genes that are similar to those of well-defined insects, including RNAi, JAK/STAT, and Imd pathways genes. This result will facilitate future studies on investigating the immune response of *L. striatellus *to microbial attack. Transcripts from RSV, the endosymbiotic bacteria *Wolbachia *and the yeast-like symbiotes were identified from our EST libraries. Functional annotation of these genes indicates that microbial processes, such as suppressor activity, de novo biosynthesis of cofactors, as well as molecule transportation, are critical for their survival in cells of *L. striatellus*. In addition, comparative genomic analysis advanced a repertoire of candidate genes that might be involved in the *L. striatellus *- RSV interaction. At present we are using more precise methods to investigate the functional roles of these candidate genes during RSV infection and transmission.

## Methods

### Insect rearing and RNA isolation

The culture of *L. striatellus *used in this study was collected from Jiangsu Province, China, and was maintained in laboratory for nearly six years. Both viruliferous and naïve *L. striatellus *were reared separately in glass beakers as stock populations. Rice (*Oryza sativa *cv. Nipponbare) plants were used as the planthoppers' diet throughout this experiment. Plants were grown in soil at 25°C under a long day photoperiod of 16 h under light and 8 h under dark in a growth incubator. The beakers were enclosed with a piece of nylon mesh after insects had been introduced into 2-3 cm high rice seedlings. The planthoppers were transferred to fresh seedlings every 10 - 14 days to assure sufficient nutrition.

For each sample, approximately 300 insect individuals were used for RNA extraction. The insects were anesthetized with ether and frozen with liquid nitrogen in mortars. Total RNA was isolated from whole bodies collected from populations of viruliferous and naïve insect populations, respectively, following the standard protocol of TRIzol reagent (Invitrogen). The concentration and quality of total RNA were determined by a NanoDrop spectrophotometer (Thermo Scientific).

### cDNA library construction and sequencing

Double-stranded cDNA was synthesized from the two RNA pools according to Ng' s full-length cDNA synthesis protocol [49] with modifications. A GsuI-oligo dT primer was used to prime first-strand cDNA synthesis from 20 μg of mRNA, using 1,000 units of Superscript II reverse transcriptase (Invitrogen). The diol group of the CAP structure of mRNA was then oxidized by NaIO4, followed by biotinylation with biotin hydrazide (long arm, Vector Laboratories). After RNaseONE digestion, Dynal M280 streptavidin Dynabeads were used to select biotinylated RNA/cDNA. The first-strand cDNA was then released by alkaline hydrolysis, and two 5' adaptors (N5 and N6 adaptors) were ligated to the 5'-end of the first-strand cDNA. Double-stranded cDNA was synthesized by primer extension using Ex Taq polymerase (TaKaRa). The polyA tail was removed by GsuI digestion, and cDNA size fractionation was performed using a cDNA size fractionation column (Invitrogen). Each cDNA fraction larger than 800 bp was sonicated to the range of 300-800 bp, and then pooled together with the other cDNA samples ranging from 300 bp to 800 bp. The prepared cDNAs were transformed into single-stranded template DNA (sstDNA) libraries by using the GS DNA Library Preparation kit (Roche Applied Science). sstDNA libraries were clonally amplified in a bead-immobilized form by using the GS emPCR kit (Roche Applied Science) and sequenced on the 454 Genome Sequencer FLX instrument.

### EST analyses and assembly

The 454 sequencing reads were filtered by an in-house developed program to remove 5' adaptor sequences and low quality reads. The qualified reads were then assembled by CAP3 [50] using default parameters. Two kinds of assembly method were used: (1) assembly of the viruliferous and naïve read libraries, separately; (2) combining the viruliferous and naïve reads and assembly of all reads as one library. A cluster containing ≥ 2 ESTs was named as a contig and those containing only one sequence were termed as singletons. The contigs and singletons were generally referred to as unigenes.

### Gene annotation

After assembly, the unigene sequences (contigs and singletons) were subjected to BlastX similarity search against NCBI-NR non-redundant protein database. The parameters were set to: amino acid substitution matrix BLOSUM62, a statistical significance threshold of 10 for similarity matches, and costs to open an alignment gap and extend a gap of 11 and 1, respectively. Furthermore, to compare our results with other arthropods, additional searches (BlastX) were also performed against putative proteomes of 11 arthropod species whose genomes are in draft or completed, including *Acyrthosiphon pisum*, *Aedes aegypti*, *Anopheles gambiae*, *Apis mellifera*, *Bombyx mori*, *Culex pipiens*, *Drosophila melanogaster*, *Ixodes scapularis*, *Nasonia vitripennis*, *Pediculus humanus*, and *Tribolium castaneum*. For prediction of the secondary structure of proteins, the pfam database was searched using HMMER 2.0 program. For Gene Ontology (GO) analysis, outputs of the results from BlastX search against Swiss-Prot/TrEMBL databases (e-value ≤ 10^-3^) were combined. Non-redundant matches were then functionally annotated with GO terminology using Go-Diff [51]. For identification of microsatellite markers that can be used in genetic mapping and population genetics, SciRoKo v3.4 software was used to search 41,492 unigenes generated from the combined EST library. Searches were carried out to identify both perfect and interrupted (≤ 2 bp mismatch) motifs, with a minimum number of repeat unit of six.

### Expression profiling by semi-quantitative RT-PCR

To verify the results of pyrosequencing of the ESTs of *L. striatellus*, we selected a total of 50 unigenes for RT-PCR profiling. Total RNAs from viruliferous and naïve samples were treated with RNase-free DNase I (TaKaRa), and about 2 μg of total RNA of each sample was reverse-transcribed by SuperScript™ III Reverse Transcriptase (Invitrogen) using oligo(dT) as primer. The PCR was carried out using the following thermal cycling profile: 95°C for 4 min, followed by 20-25 cycles of amplification (95°C for 30 sec, 55°C for 30 sec, and 72°C for 45 sec), and 72°C for 10 min. Primers were designed based on contig sequences generated from ESTs of the same cluster (Additional file 6). The PCR products and their sizes were examined using 1% agarose gel electrophoresis followed by ethidium bromide staining. The elongation factor 2 (EF2) gene of *L. striatellus *was amplified as an endogenous loading control for testing the validity of template preparation. The expression of each gene was confirmed in at least two rounds of independent RT-PCR reactions.

## Authors' contributions

FZ performed most of the analyses of the EST database and conducted the RT-PCR profiling. HG and TZ bred the insects and took part in extracting total RNA. HZ conducted 454 pyrosequencing, contig assembly and bioinformatic analysis. YZ provided the insect lines used in this study. WQ conducted the data analyses and prepared the manuscript. SW, RF, WQ and XC conceived, designed and coordinated the study. All authors have read, commented upon, and approved the final manuscript.

## Supplementary Material

Additional file 1 **Distribution of read lengths of viruliferous and naïve Laodelphax striatellus EST libraries**. The figure provides statistics of read lengths of L. striatellus ESTs sequencing by the Roche 454-FLX platform. Click here for file

Additional file 2 **List of Wolbachia genes identified in L. striatellus EST libraries**. This table provides a list of genes from endosymbiotic bacteria Wolbachia that are expressed in cells of L. striatellus. Click here for file

Additional file 3 **List of possible fungal genes identified in L. striatellus EST libraries**. The table provides a list of genes that possibly expressed by endosymbiotic fungi, including yeast-like symbiotes. Click here for file

Additional file 4 **L. striatellus genes abundantly transcribed in the viruliferous sample**. The table provides a list of top 30 genes that highly expressed in the sample of viruliferous insects. Click here for file

Additional file 5 **List of genes selected for RT-PCR assay**. This table shows the general feature of genes selected for RT-PCR analysis. Click here for file

Additional file 6 **Sequences of primers used in RT-PCR profiling**. Click here for file
