# Supplementary material for: Massively parallel pyrosequencing-based transcriptome analyses of small brown planthopper (Laodelphax striatellus), a vector insect transmitting rice stripe virus (RSV)
Source: BMC Genomics. 2010 May 13;11:303. doi: 10.1186/1471-2164-11-303 (PMC2885366; doi:10.1186/1471-2164-11-303)
Supplement: Additional file 1 — Distribution of read lengths of viruliferous and naïve Laodelphax striatellus EST libraries. The figure provides statistics of read lengths of L. striatellus ESTs sequencing by the Roche 454-FLX platform. [file 1471-2164-11-303-S1.PDF]

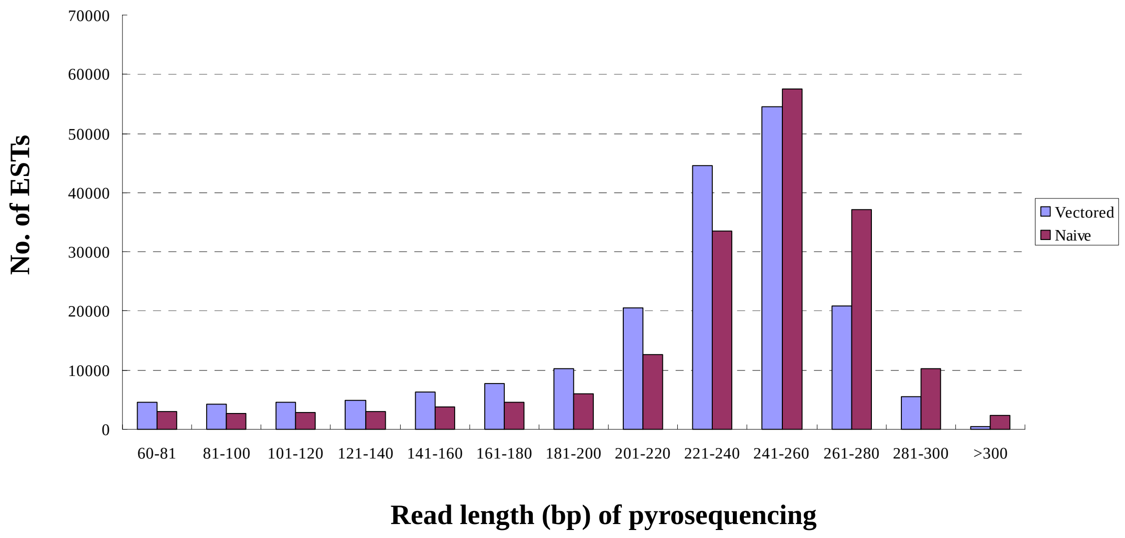

Additional file 1

Distribution of the read lengths of vectored (viruliferous) and naïve (non-infected) *Laodelphax striatellus* cDNA liberaries.
