# Supplementary material for: Massively parallel pyrosequencing-based transcriptome analyses of small brown planthopper (Laodelphax striatellus), a vector insect transmitting rice stripe virus (RSV)
Source: BMC Genomics. 2010 May 13;11:303. doi: 10.1186/1471-2164-11-303 (PMC2885366; doi:10.1186/1471-2164-11-303)
Supplement: Additional file 2 — List of Wolbachia genes identified in L. striatellus EST libraries. This table provides a list of genes from endosymbiotic bacteria Wolbachia that are expressed in cells of L. striatellus. [file 1471-2164-11-303-S2.HTM]

| Additional Table 2. Wolbachia transcripts identified in EST library | | | | | | | |
|  |  |  |  |  |  |  |  |
| Code of Contig | Description | BlastN score | BlastN e-value | BlastN identity | BlastX score | BlastX evalue | BlastX identity |
|  |  |  |  |  |  |  |  |
| Ribosome assemble | |  |  |  |  |  |  |
| Contig10729 | ribosomal protein S12 | 799 | 0.00E+00 | 427/435 | 239 | 6.00E-62 | 119/124 |
| Contig13086 | 30S ribosomal protein S11 | 605 | 1.00E-170 | 317/321 | 189 | 7.00E-47 | 93/106 |
| Contig13308 | 50S ribosomal protein L11 | 410 | 1.00E-111 | 225/231 | 70.9 | 3.00E-11 | 34/36 |
| Contig9924 | ribosomal protein L29 | 718 | 0.00E+00 | 413/430 | 101 | 2.00E-20 | 51/65 |
| Contig9908 | 50S ribosomal protein L2 | 428 | 1.00E-117 | 258/272 | 189 | 5.00E-47 | 88/90 |
| FQ92HJ001DMRFI | 50S ribosomal protein L16 | 472 | 1.00E-130 | 283/295 | 368 | 5.00E-34 | 73/85 |
| FQ92HJ001DPXPM | 30S ribosomal protein S3 | 450 | 1.00E-123 | 254/263 | 321 | 2.00E-28 | 59/77 |
| FQ92HJ001ATS7F | ribosomal protein L14 | 170 | 2.00E-39 | 171/179 | 142 | 2.00E-13 | 31/54 |
| FQ92HJ001EG47D | ribosomal protein S21 | 174 | 1.00E-40 | 192/201 | 186 | 7.00E-13 | 40/66 |
| FQ92HJ001BQ4TM | 50S ribosomal protein L21 | 129 | 2.00E-27 | 65/65 | 86 | 2.60E-01 | 17/17 |
|  |  |  |  |  |  |  |  |
| Nucleic acids metabolism | |  |  |  |  |  |  |
| Contig10501 | RPOB\_WOLPI DNA-directed RNA polymerase subunit beta (RNAP subunit beta) | 480 | 1.00E-132 | 254/258 | 169 | 6.00E-41 | 81/85 |
| FQ92HJ001BGDB0 | dnaJ protein | 498 | 1.00E-138 | 265/265 | 253 | 1.00E-21 | 48/49 |
| FQ92HJ001A8SI1 | dnaX | 484 | 1.00E-133 | 262/268 | 303 | 2.00E-26 | 57/67 |
| FQ92HJ001DZPMS | RPOB\_WOLPI DNA-directed RNA polymerase subunit beta (RNAP subunit beta) | 480 | 1.00E-132 | 248/250 | 415 | 2.00E-39 | 83/84 |
| FQ92HJ001CKLTI | RNA-directed DNA polymerase | 196 | 3.00E-47 | 189/219 | 337 | 2.00E-30 | 62/76 |
| FQ92HJ001DVEUU | lysyl-tRNA synthetase | 494 | 1.00E-136 | 270/277 | 359 | 6.00E-33 | 67/92 |
| FQ92HJ001EJXQ1 | amidophosphoribosyltransferase | 450 | 1.00E-123 | 255/262 | 312 | 2.00E-27 | 67/86 |
|  |  |  |  |  |  |  |  |
| Transpotation and chaperones | |  |  |  |  |  |  |
| Contig9015 | preprotein translocase SecY | 381 | 1.00E-102 | 220/232 | 92.8 | 7.00E-18 | 50/77 |
| FQ92HJ001D4Q4U | Molecular chaperone, DnaK | 462 | 1.00E-127 | 254/262 | 282 | 3.00E-28 | 56/69 |
| FQ92HJ001EWT6R | Na+/H+ antiporter, putative | 345 | 6.00E-92 | 213/226 | 259 | 2.00E-21 | 49/75 |
| FQ92HJ001D7NII | Permease of the major facilitator superfamily | 323 | 3.00E-85 | 197/206 | 260 | 4.00E-25 | 48/52 |
| FQ92HJ001EM2UN | type I secretion system ATPase | 268 | 8.00E-69 | 166/175 | 121 | 1.00E-10 | 27/41 |
| FQ92HJ001EADZ8 | similar to GroES protein | 505 | 1.00E-140 | 258/259 | 394 | 5.00E-37 | 79/80 |
| FQ92HJ001E5EMT | Cold shock protein | 438 | 1.00E-120 | 260/273 | 218 | 1.00E-16 | 39/47 |
|  |  |  |  |  |  |  |  |
| FQ92HJ001DEYFL | heat shock protein, class I | 377 | 1.00E-101 | 197/198 | 285 | 2.00E-24 | 54/59 |
|  |  |  |  |  |  |  |  |
| Energy metabolism |  |  |  |  |  |  |  |
| Contig15463 | succinate dehydrogenase | 458 | 1.00E-126 | 231/231 | 160 | 2.00E-38 | 74/80 |
|  |  |  |  |  |  |  |  |
| Cofactor biosynthesis | |  |  |  |  |  |  |
| FQ92HJ001B35OU | Succinyl-CoA synthetase, alpha subunit | 266 | 5.00E-68 | 215/242 | 309 | 4.00E-27 | 62/82 |
| FQ92HJ001E00JD | AF348330\_1 4-hydroxybenzoate octaprenyltransferase | 434 | 1.00E-119 | 238/242 | 284 | 7.00E-35 | 54/54 |
| FQ92HJ001C6L44 | 3-demethylubiquinone-9 3-methyltransferase | 297 | 1.00E-77 | 211/229 | 261 | 2.00E-22 | 52/68 |
| FQ92HJ001BDH9C | Mg chelatase-related protein | 204 | 1.00E-49 | 179/180 | 199 | 2.00E-14 | 40/42 |
| FQ92HJ001AS7V2 | phosphopantetheine adenylyltransferase | 278 | 1.00E-71 | 209/232 | 384 | 7.00E-36 | 76/80 |
| FQ92HJ001AD94B | riboflavin biosynthesis protein RibD | 220 | 2.00E-54 | 186/211 | 331 | 1.00E-29 | 61/70 |
|  |  |  |  |  |  |  |  |
| Protein modification and degradation | |  |  |  |  |  |  |
| FQ92HJ001B5UST | ATP-dependent protease La | 472 | 1.00E-130 | 238/238 | 325 | 5.00E-29 | 63/78 |
| Contig10978 | Thiol-disulfide isomerase, thioredoxin family | 581 | 1.00E-162 | 371/396 | 251 | 9.00E-66 | 116/127 |
|  |  |  |  |  |  |  |  |
| Cell signalling |  |  |  |  |  |  |  |
| FQ92HJ001DD868 | tldD protein | 470 | 1.00E-129 | 240/241 | 376 | 6.00E-35 | 70/80 |
| FQ92HJ001A6PDY | leucine rich protein | 412 | 1.00E-112 | 260/264 | 75 | 4.00E-04 | 15/22 |
|  |  |  |  |  |  |  |  |
| Stress tolerance |  |  |  |  |  |  |  |
| FQ92HJ001EIF2G | multidrug resistance protein D | 460 | 1.00E-126 | 232/232 | 327 | 3.00E-29 | 71/77 |
|  |  |  |  |  |  |  |  |
|  |  |  |  |  |  |  |  |
| Function unknown proteins | |  |  |  |  |  |  |
| Contig4613 | conserved hypothetical protein | 2365 | 0.00E+00 | 1285/1312 | 162 | 7.00E-38 | 114/265 |
| Contig11695 | unknow protein | 2329 | 0.00E+00 | 1208/1219 | 138 | 7.00E-31 | 92/178 |
| Contig12778 | conserved hypothetical protein | 2224 | 0.00E+00 | 1146/1154 | 117 | 4.00E-44 | 61/99 |
| Contig9178 | hypothetical protein RT0201 | 579 | 1.00E-162 | 324/337 | 114 | 2.00E-24 | 55/72 |
| Contig15257 | hypothetical protein Wbm0771 | 315 | 1.00E-82 | 232/245 | 34.7 | 7.00E-03 | 21/48 |
| Contig10702 | hypothetical protein WD0973 | 331 | 2.00E-87 | 265/300 | 55.8 | 9.00E-07 | 37/116 |
| Contig12819 | hypothetical protein Wendoof\_01000771 | 571 | 1.00E-160 | 311/320 | 65.9 | 1.00E-10 | 31/37 |
| Contig8531 | hypothetical protein Wendoof\_01000256 | 492 | 1.00E-136 | 254/256 | 151 | 1.00E-35 | 74/85 |
| Contig8625 | unnamed protein product | 468 | 1.00E-129 | 245/248 | 35.8 | 1.00E+00 | 19/36 |
| Contig10424 | conserved hypothetical protein | 392 | 1.00E-106 | 216/222 | 97.1 | 4.00E-19 | 48/74 |
| Contig11564 | conserved hypothetical protein | 339 | 5.00E-90 | 189/195 | 85.5 | 1.00E-15 | 47/76 |
| Contig9405 | hypothetical protein Wendoof\_01000802 | 303 | 1.00E-79 | 159/161 | 105 | 1.00E-21 | 51/54 |
| Contig14129 | conserved hypothetical protein | 628 | 1.00E-177 | 339/344 | 97.1 | 4.00E-19 | 43/51 |
| Contig11882 | hypothetical protein WD0576 | 454 | 1.00E-124 | 275/290 | 156 | 4.00E-37 | 86/125 |
| Contig16198 | hypothetical protein PEPMIC\_00056 | 577 | 1.00E-161 | 300/303 | 103 | 3.00E-21 | 55/99 |
| FQ92HJ001C4NYS | AF287482\_5 Orf122 | 278 | 1.00E-71 | 140/140 | 93 | 4.00E-02 | 20/40 |
| FQ4QJ5301CXVO5 | hypothetical protein Wendoof\_01000887 | 456 | 1.00E-125 | 250/259 | 289 | 8.00E-25 | 60/75 |
| FQ92HJ001AJAQH | conserved hypothetical protein | 252 | 9.00E-64 | 157/167 | 148 | 2.00E-08 | 26/58 |
| FQ92HJ001C0MMB | similar to hypothetical protein | 420 | 1.00E-114 | 245/249 | 364 | 1.00E-36 | 70/72 |
| FQ92HJ001CBCC7 | hypothetical protein Wendoof\_01000852 | 347 | 1.00E-92 | 178/179 | 168 | 8.00E-11 | 29/32 |
| FQ92HJ001BA1T6 | similar to hypothetical protein | 341 | 9.00E-91 | 203/212 | 236 | 3.00E-24 | 47/52 |
| FQ92HJ001BIYIG | Hypothetical protein COLAER\_01802 | 153 | 4.00E-34 | 77/77 | 108 | 8.00E-04 | 21/28 |
| FQ92HJ001D82BR | hypothetical protein Wendoof\_01000786 | 533 | 1.00E-148 | 290/297 | 410 | 7.00E-39 | 83/99 |
| FQ92HJ001EH9CO | hypothetical protein Wendoof\_01000801 | 509 | 1.00E-141 | 266/269 | 362 | 3.00E-33 | 70/89 |
| FQ92HJ001CLWQO | hypothetical protein Wendoof\_01000523 | 496 | 1.00E-137 | 262/265 | 341 | 7.00E-31 | 64/72 |
| FQ92HJ001CWGUL | hypothetical protein Wendoof\_01000244 | 444 | 1.00E-122 | 227/228 | 399 | 1.00E-37 | 70/76 |
| FQ92HJ001DQ8GK | hypothetical protein Wbm0253 | 430 | 1.00E-117 | 235/241 | 91 | 6.90E-02 | 21/47 |
| FQ92HJ001CX81Y | hypothetical protein WD0572 | 420 | 1.00E-114 | 255/267 | 379 | 3.00E-35 | 76/81 |
| FQ92HJ001AGHB8 | conserved hypothetical protein | 414 | 1.00E-113 | 231/231 | 163 | 3.00E-10 | 33/50 |
| FQ92HJ001A6B9G | hypothetical protein Wendoof\_01000884 | 408 | 1.00E-111 | 248/269 | 201 | 1.00E-14 | 40/49 |
| FQ92HJ001AYJ8B | hypothetical protein Wendoof\_01000922 | 408 | 1.00E-111 | 224/230 | 358 | 8.00E-33 | 70/72 |
| FQ92HJ001EGV44 | hypothetical protein Wendoof\_01000750 | 391 | 1.00E-105 | 209/213 | 333 | 6.00E-30 | 63/75 |
| FQ92HJ001BQ96C | hypothetical protein Wendoof\_01000461 | 367 | 2.00E-98 | 212/217 | 270 | 1.00E-22 | 53/72 |
| FQ92HJ001A0CA4 | hypothetical protein Wendoof\_01000684 | 321 | 9.00E-85 | 221/243 | 158 | 1.00E-09 | 41/77 |
| FQ92HJ001A066J | hypothetical protein WwAna1450 | 299 | 3.00E-78 | 240/248 | 267 | 3.00E-23 | 50/74 |
| FQ92HJ001DYT8J | hypothetical protein Wendoof\_01000390 | 289 | 2.00E-75 | 149/150 | 267 | 3.00E-22 | 50/50 |
| FQ92HJ001ENR11 | hypothetical protein Wendoof\_01000590 | 289 | 3.00E-75 | 188/202 | 325 | 5.00E-29 | 64/67 |
| FQ92HJ001DR656 | conserved hypothetical protein | 268 | 1.00E-68 | 196/218 | 218 | 3.00E-17 | 45/55 |
| FQ92HJ001D8CUH | hypothetical protein Wendoof\_01000684 | 258 | 9.00E-66 | 149/153 | 123 | 1.00E-05 | 28/51 |
| FQ4QJ5301DJZW1 | hypothetical protein CKO\_00099 | 234 | 2.00E-58 | 121/122 | 91 | 6.90E-02 | 22/42 |
| FQ92HJ001ENK0D | hypothetical protein WD0835 | 232 | 7.00E-58 | 179/188 | 115 | 1.00E-04 | 34/78 |
| FQ92HJ001DU870 | hypothetical protein Wendoof\_01000102 | 216 | 4.00E-53 | 195/221 | 137 | 3.00E-07 | 31/52 |
| FQ92HJ001D1U71 | hypothetical protein Wendoof\_01000389 | 210 | 3.00E-51 | 195/200 | 158 | 1.00E-09 | 31/37 |
| FQ92HJ001EF8Q7 | hypothetical protein WD0424 | 208 | 1.00E-50 | 232/240 | 210 | 1.00E-15 | 43/65 |
| FQ92HJ001BBGWY | conserved hypothetical protein | 198 | 1.00E-47 | 214/220 | 310 | 3.00E-27 | 65/98 |
| FQ92HJ001C6U8B | conserved hypothetical protein | 174 | 5.00E-41 | 99/99 | 105 | 2.00E-03 | 21/31 |
| FQ92HJ001ALAVB | hypothetical protein WD0434 | 172 | 4.00E-40 | 149/172 | 77 | 3.00E+00 | 18/49 |
| FQ92HJ001EAO8M | hypothetical protein Mlab\_0841 | 131 | 2.00E-27 | 90/93 | 84 | 4.60E-01 | 15/38 |
| FQ92HJ001BTAF6 | conserved hypothetical protein | 125 | 7.00E-26 | 63/63 | 78 | 2.20E+00 | 14/18 |
| FQ92HJ001CU80X | predicted protein | 91.7 | 1.00E-15 | 121/130 | 84 | 4.60E-01 | 17/33 |
| FQ92HJ001AUTA4 | hypothetical protein Wendoof\_01000204 | 353 | 2.00E-94 | 184/186 | 321 | 1.00E-28 | 60/63 |
| FQ92HJ001B5SZ8 | hypothetical protein WwAna1272 | 337 | 1.00E-89 | 170/170 | 170 | 5.00E-11 | 30/31 |
| FQ4QJ5301AQ19O | hypothetical protein WwAna1272 | 311 | 8.00E-82 | 157/157 | 126 | 6.00E-06 | 23/23 |
| FQ92HJ001BC5FN | conserved hypothetical protein | 115 | 1.00E-22 | 67/70 | 151 | 8.00E-09 | 28/36 |
| FQ92HJ001AY7O3 | hypothetical protein Wendoof\_01000204 | 95.6 | 2.00E-17 | 54/56 | 101 | 5.00E-03 | 19/21 |
| FQ92HJ001BY8QN | hypothetical protein Wendoof\_01000245 | 73.8 | 4.00E-10 | 55/61 | 214 | 4.00E-16 | 41/81 |
| FQ92HJ001AIU0U | hypothetical protein Wendoof\_01000049 | 468 | 1.00E-129 | 260/268 | 478 | 9.00E-47 | 89/90 |
| FQ92HJ001BHA1Y | hypothetical protein | 309 | 4.00E-81 | 234/260 | 295 | 2.00E-25 | 59/87 |
| FQ92HJ001DS8TW | hypothetical protein | 139 | 8.00E-30 | 219/265 | 286 | 2.00E-24 | 57/89 |
|  |  |  |  |  |  |  |  |
