# Supplementary material for: Massively parallel pyrosequencing-based transcriptome analyses of small brown planthopper (Laodelphax striatellus), a vector insect transmitting rice stripe virus (RSV)
Source: BMC Genomics. 2010 May 13;11:303. doi: 10.1186/1471-2164-11-303 (PMC2885366; doi:10.1186/1471-2164-11-303)
Supplement: Additional file 3 — List of possible fungal genes identified in L. striatellus EST libraries. The table provides a list of genes that possibly expressed by endosymbiotic fungi, including yeast-like symbiotes. [file 1471-2164-11-303-S3.HTM]

| Additional file 3. Unigenes that matched to fungi species | | | | | |
| �� | | | | | |
| Contig | Score | E-value | Identities | Organism | Description |
| Contig15381 | 55.5 | 3.00E-06 | 23/33 (69%) | Abronia aurita | cytochrome b |
| FQ92HJ001B9Y8G | 140 | 1.00E-07 | 25/40 (62%) | Abronia aurita | cytochrome b |
| Contig1431 | 35.8 | 2.70E+00 | 27/76 (35%) | Acanthamoeba castellanii | NADH dehydrogenase, subunit 11 |
| Contig15547 | 33.1 | 6.60E+00 | 21/61 (34%) | Acanthamoeba polyphaga mimivirus | serine/threonine protein kinase |
| Contig12262 | 104 | 2.00E-21 | 45/61 (73%) | Acremonium chrysogenum | aminoadipate reductase enzyme |
| FQ4QJ5301D4LYX | 87 | 2.10E-01 | 28/75 (37%) | Acremonium chrysogenum | aminoadipate reductase enzyme |
| FQ4QJ5301AO63Q | 232 | 3.00E-18 | 44/63 (69%) | Acremonium chrysogenum | aminoadipate reductase enzyme |
| FQ92HJ001CI5ZH | 106 | 1.00E-03 | 24/48 (50%) | Acremonium chrysogenum | aminoadipate reductase enzyme |
| FQ92HJ001CHGDM | 393 | 7.00E-37 | 71/82 (86%) | Acremonium chrysogenum | aminoadipate reductase enzyme |
| FQ92HJ001BLED4 | 390 | 2.00E-36 | 75/84 (89%) | Acremonium chrysogenum | AF286193\_1 cystathionine-gamma-lyase |
| FQ4QJ5301EBH92 | 146 | 2.00E-14 | 26/39 (66%) | Acremonium chrysogenum | AF286193\_2 unknown |
| FQ4QJ5301EWW5F | 290 | 6.00E-25 | 49/74 (66%) | Acremonium sp. OXF C13 | beta-1,3-glucanase |
| Contig7398 | 33.1 | 6.40E+00 | 20/60 (33%) | Amylomyces rouxii | AF157230\_1 translation elongation factor 1-alpha |
| Contig16862 | 39.3 | 9.20E-02 | 17/51 (33%) | Ashbya gossypii ATCC 10895 | AAL182Wp |
| FQ4QJ5301AFW6S | 81 | 1.00E+00 | 23/40 (57%) | Ashbya gossypii ATCC 10895 | AAR051Cp |
| Contig4307 | 43.1 | 2.70E-02 | 25/75 (33%) | Ashbya gossypii ATCC 10895 | AAR069Wp |
| Contig13951 | 32.7 | 8.40E+00 | 25/83 (30%) | Ashbya gossypii ATCC 10895 | AAR104Wp |
| FQ4QJ5301AUABW | 79 | 1.70E+00 | 23/65 (35%) | Ashbya gossypii ATCC 10895 | ACR148Wp |
| Contig2136 | 33.1 | 6.60E+00 | 18/45 (40%) | Ashbya gossypii ATCC 10895 | ADL186Cp |
| Contig7965 | 32.7 | 8.40E+00 | 12/29 (41%) | Ashbya gossypii ATCC 10895 | ADL189Wp |
| FQ4QJ5301BAMJ2 | 73 | 8.40E+00 | 17/45 (37%) | Ashbya gossypii ATCC 10895 | ADL274Wp |
| Contig4608 | 33.5 | 5.00E+00 | 22/55 (40%) | Ashbya gossypii ATCC 10895 | AEL113Cp |
| Contig10045 | 33.9 | 9.60E+00 | 28/97 (28%) | Ashbya gossypii ATCC 10895 | AER370Wp |
| FQ92HJ001CII51 | 77 | 2.90E+00 | 22/69 (31%) | Ashbya gossypii ATCC 10895 | AER441Cp |
| Contig12670 | 51.2 | 2.00E-05 | 18/53 (33%) | Ashbya gossypii ATCC 10895 | AFL214Cp |
| FQ92HJ001BBQ56 | 93 | 2.00E-05 | 14/26 (53%) | Ashbya gossypii ATCC 10895 | AFL220Cp |
| FQ92HJ001C9O4P | 77 | 2.90E+00 | 16/37 (43%) | Ashbya gossypii ATCC 10895 | AFR433Cp |
| Contig12736 | 38.1 | 2.00E-01 | 23/85 (27%) | Ashbya gossypii ATCC 10895 | AGR074Cp |
| FQ4QJ5301EGAT9 | 91 | 6.90E-02 | 14/18 (77%) | Ashbya gossypii ATCC 10895 | AGR199Wp |
| Contig11157 | 98.6 | 1.00E-19 | 46/66 (69%) | Aspergillus | C-1-tetrahydrofolate synthase, mitochondrial precursor |
| FQ4QJ5301AYFOP | 78 | 2.20E+00 | 21/66 (31%) | Aspergillus | chromosomal organization and DNA repair protein Mms21 |
| FQ4QJ5301CURT1 | 342 | 5.00E-31 | 67/79 (84%) | Aspergillus | DNA replication factor C subunit Rfc3, putative |
| FQ4QJ5301DHEAB | 74 | 6.50E+00 | 21/73 (28%) | Aspergillus clavatus | DEAD/DEAH box DNA helicase (Mer3), putative |
| Contig8923 | 34.3 | 4.70E+00 | 17/48 (35%) | Aspergillus clavatus | integral membrane protein (Ptm1), putative |
| FQ92HJ001A1QW7 | 211 | 1.00E-18 | 44/65 (67%) | Aspergillus clavatus | t-complex protein 1, beta subunit, putative |
| Contig8511 | 47.4 | 3.00E-08 | 22/28 (78%) | Aspergillus clavatus | vacuolar ATP synthase subunit D, putative |
| FQ92HJ001D1EU5 | 178 | 6.00E-12 | 39/86 (45%) | Aspergillus clavatus NRRL | GDSL lipase/acylhydrolase family protein |
| Contig14921 | 33.1 | 6.50E+00 | 15/29 (51%) | Aspergillus clavatus NRRL | Myb-like DNA-binding protein, putative |
| FQ92HJ001ET6LV | 74 | 6.40E+00 | 15/29 (51%) | Aspergillus clavatus NRRL | Myb-like DNA-binding protein, putative |
| FQ4QJ5301BVJNT | 74 | 6.60E+00 | 17/44 (38%) | Aspergillus clavatus NRRL | ubiquitin C-terminal hydrolase, putative |
| Contig14071 | 119 | 7.00E-26 | 56/57 (98%) | Aspergillus clavatus NRRL 1 | 40S ribosomal protein S4, putative |
| FQ4QJ5301CJJ4K | 106 | 1.00E-03 | 23/42 (54%) | Aspergillus clavatus NRRL 1 | 6-methylsalicylic acid synthase MsaS |
| FQ4QJ5301ETO4I | 73 | 8.50E+00 | 17/51 (33%) | Aspergillus clavatus NRRL 1 | acetyl-CoA carboxylase |
| FQ92HJ001DRFD2 | 284 | 3.00E-24 | 48/70 (68%) | Aspergillus clavatus NRRL 1 | adhesin, putative |
| Contig1420 | 35.4 | 1.30E+00 | 17/51 (33%) | Aspergillus clavatus NRRL 1 | allergen, putative |
| Contig4341 | 38.1 | 2.00E-01 | 30/96 (31%) | Aspergillus clavatus NRRL 1 | BTB domain and ankyrin repeat protein |
| FQ4QJ5301EQ7BG | 80 | 1.30E+00 | 18/67 (26%) | Aspergillus clavatus NRRL 1 | cell wall protein, putative |
| Contig5543 | 35.8 | 1.00E+00 | 21/42 (50%) | Aspergillus clavatus NRRL 1 | CNH domain protein |
| Contig3999 | 33.9 | 4.40E+00 | 21/49 (42%) | Aspergillus clavatus NRRL 1 | conserved hypothetical protein |
| Contig4679 | 32.7 | 8.60E+00 | 12/40 (30%) | Aspergillus clavatus NRRL 1 | conserved hypothetical protein |
| Contig9669 | 36.6 | 5.80E-01 | 19/45 (42%) | Aspergillus clavatus NRRL 1 | conserved hypothetical protein |
| Contig11897 | 34.7 | 2.20E+00 | 24/76 (31%) | Aspergillus clavatus NRRL 1 | conserved hypothetical protein |
| FQ4QJ5301B6Z62 | 131 | 8.00E-12 | 25/40 (62%) | Aspergillus clavatus NRRL 1 | conserved hypothetical protein |
| FQ4QJ5301B66XG | 74 | 6.60E+00 | 17/33 (51%) | Aspergillus clavatus NRRL 1 | conserved hypothetical protein |
| FQ92HJ001EP9WF | 143 | 7.00E-08 | 30/73 (41%) | Aspergillus clavatus NRRL 1 | conserved hypothetical protein |
| FQ92HJ001CAUGS | 75 | 5.00E+00 | 18/49 (36%) | Aspergillus clavatus NRRL 1 | conserved hypothetical protein |
| FQ92HJ001BNYTM | 149 | 1.00E-08 | 27/39 (69%) | Aspergillus clavatus NRRL 1 | conserved hypothetical protein |
| FQ4QJ5301AZLTV | 85 | 3.40E-01 | 18/67 (26%) | Aspergillus clavatus NRRL 1 | DEAD/DEAH box helicase, putative |
| Contig13393 | 32.7 | 8.50E+00 | 21/50 (42%) | Aspergillus clavatus NRRL 1 | F-box domain protein |
| Contig10285 | 110 | 8.00E-33 | 55/67 (82%) | Aspergillus clavatus NRRL 1 | hypothetical protein ACLA\_028940 |
| FQ92HJ001ALFZY | 146 | 5.00E-16 | 27/45 (60%) | Aspergillus clavatus NRRL 1 | hypothetical protein ACLA\_078250 |
| Contig5214 | 35.4 | 1.30E+00 | 18/59 (30%) | Aspergillus clavatus NRRL 1 | lactonohydrolase, putative |
| Contig1444 | 33.9 | 3.90E+00 | 15/26 (57%) | Aspergillus clavatus NRRL 1 | leucine rich repeat protein |
| FQ4QJ5301AX5KZ | 74 | 6.50E+00 | 15/49 (30%) | Aspergillus clavatus NRRL 1 | methylenetetrahydrofolate reductase |
| FQ92HJ001ATMNB | 76 | 3.90E+00 | 14/32 (43%) | Aspergillus clavatus NRRL 1 | RING finger protein |
| FQ92HJ001C6R5C | 79 | 1.70E+00 | 21/53 (39%) | Aspergillus clavatus NRRL 1 | SH3 domain protein |
| Contig2654 | 34.7 | 2.20E+00 | 25/70 (35%) | Aspergillus clavatus NRRL 1 | WD repeat protein |
| FQ4QJ5301A2HYU | 222 | 5.00E-17 | 43/72 (59%) | Aspergillus fumigatus | ATP dependent DNA ligase domain protein |
| FQ4QJ5301DPQ0I | 402 | 6.00E-38 | 81/84 (96%) | Aspergillus fumigatus | t-complex protein 1, alpha subunit, putative |
| Contig4179 | 124 | 2.00E-27 | 56/59 (94%) | Aspergillus fumigatus A1163 | 40S ribosomal protein S10b |
| Contig8566 | 33.5 | 4.90E+00 | 15/15 (100%) | Aspergillus fumigatus A1163 | 40S ribosomal protein S19 |
| FQ92HJ001AY5VF | 75 | 5.10E+00 | 11/43 (25%) | Aspergillus fumigatus A1163 | alpha-glucosidase AgdA, putative |
| FQ92HJ001DDFA3 | 79 | 1.70E+00 | 14/33 (42%) | Aspergillus fumigatus A1163 | C2H2 finger domain protein |
| Contig1129 | 33.9 | 3.80E+00 | 20/50 (40%) | Aspergillus fumigatus A1163 | conserved hypothetical protein |
| Contig3854 | 37 | 4.50E-01 | 21/54 (38%) | Aspergillus fumigatus A1163 | conserved hypothetical protein |
| Contig8291 | 110 | 4.00E-23 | 54/110 (49%) | Aspergillus fumigatus A1163 | conserved hypothetical protein |
| Contig13215 | 54.7 | 2.00E-06 | 21/30 (70%) | Aspergillus fumigatus A1163 | conserved hypothetical protein |
| Contig13252 | 33.1 | 6.50E+00 | 25/83 (30%) | Aspergillus fumigatus A1163 | conserved hypothetical protein |
| Contig16642 | 33.5 | 5.10E+00 | 21/88 (23%) | Aspergillus fumigatus A1163 | conserved hypothetical protein |
| FQ4QJ5301D565J | 77 | 3.00E+00 | 17/40 (42%) | Aspergillus fumigatus A1163 | conserved hypothetical protein |
| FQ4QJ5301C6DFE | 77 | 2.90E+00 | 17/40 (42%) | Aspergillus fumigatus A1163 | conserved hypothetical protein |
| FQ4QJ5301BN3QZ | 74 | 6.60E+00 | 13/37 (35%) | Aspergillus fumigatus A1163 | conserved hypothetical protein |
| FQ4QJ5301D35OM | 75 | 4.90E+00 | 16/52 (30%) | Aspergillus fumigatus A1163 | conserved hypothetical protein |
| FQ92HJ001AXKWE | 79 | 1.70E+00 | 19/60 (31%) | Aspergillus fumigatus A1163 | conserved hypothetical protein |
| FQ92HJ001C2XEW | 79 | 1.70E+00 | 16/32 (50%) | Aspergillus fumigatus A1163 | DIL and Ankyrin domain protein |
| Contig12357 | 39.7 | 6.90E-02 | 25/87 (28%) | Aspergillus fumigatus A1163 | glycosyl transferase, putative |
| Contig6388 | 35 | 3.40E+00 | 20/62 (32%) | Aspergillus fumigatus A1163 | HET domain protein |
| Contig11395 | 33.9 | 3.80E+00 | 14/49 (28%) | Aspergillus fumigatus A1163 | hypothetical protein AFUB\_075490 |
| FQ4QJ5301CO3O0 | 106 | 1.00E-03 | 19/37 (51%) | Aspergillus fumigatus A1163 | RING finger protein |
| Contig4550 | 34.7 | 2.20E+00 | 19/52 (36%) | Aspergillus fumigatus A1163 | salicylate hydroxylase, putative |
| FQ92HJ001AOELG | 282 | 5.00E-24 | 52/75 (69%) | Aspergillus fumigatus A1163 | transferase (Gpi7), putative |
| Contig1516 | 35 | 1.80E+00 | 20/57 (35%) | Aspergillus fumigatus Af293 | aquaglyceroporin |
| FQ4QJ5301CHPN2 | 73 | 8.60E+00 | 16/34 (47%) | Aspergillus fumigatus Af293 | CCCH zinc finger protein |
| FQ4QJ5301ALD8N | 90 | 9.30E-02 | 17/32 (53%) | Aspergillus fumigatus Af293 | cell surface protein |
| Contig5244 | 33.9 | 3.90E+00 | 18/45 (40%) | Aspergillus fumigatus Af293 | conserved hypothetical protein |
| Contig10806 | 36.6 | 6.00E-01 | 22/74 (29%) | Aspergillus fumigatus Af293 | conserved hypothetical protein |
| FQ4QJ5301CM5BA | 77 | 3.00E+00 | 19/55 (34%) | Aspergillus fumigatus Af293 | conserved hypothetical protein |
| FQ4QJ5301EHDTR | 73 | 8.60E+00 | 20/60 (33%) | Aspergillus fumigatus Af293 | conserved hypothetical protein |
| FQ92HJ001B0BO0 | 136 | 4.00E-07 | 35/62 (56%) | Aspergillus fumigatus Af293 | conserved hypothetical protein |
| FQ92HJ001AZHY7 | 147 | 2.00E-08 | 25/37 (67%) | Aspergillus fumigatus Af293 | Defensin domain protein |
| FQ4QJ5301APHBI | 76 | 3.80E+00 | 18/62 (29%) | Aspergillus fumigatus Af293 | DUF455 domain protein |
| FQ4QJ5301BGAJD | 182 | 4.00E-26 | 31/32 (96%) | Aspergillus fumigatus Af293 | fatty acid hydroxylase |
| FQ92HJ001BTI2X | 73 | 8.60E+00 | 18/34 (52%) | Aspergillus fumigatus Af293 | Hsp40 co-chaperone Jid1 |
| Contig5485 | 130 | 3.00E-29 | 65/75 (86%) | Aspergillus fumigatus Af293 | Hsp70 chaperone (HscA) |
| FQ4QJ5301E0JO9 | 74 | 6.50E+00 | 19/62 (30%) | Aspergillus fumigatus Af293 | integral membrane protein |
| FQ92HJ001ARPPP | 74 | 6.60E+00 | 15/39 (38%) | Aspergillus fumigatus Af293 | kinesin family protein (KinA) |
| Contig4422 | 32.7 | 8.50E+00 | 24/77 (31%) | Aspergillus fumigatus Af293 | MFS multidrug transporter |
| Contig14131 | 33.5 | 6.90E+00 | 16/36 (44%) | Aspergillus fumigatus Af293 | NACHT and Ankyrin domain protein |
| Contig945 | 43.9 | 4.00E-03 | 22/44 (50%) | Aspergillus fumigatus Af293 | polyubiquitin UbiD/Ubi4 |
| FQ4QJ5301DF8XY | 74 | 6.60E+00 | 15/44 (34%) | Aspergillus fumigatus Af293 | ubiquitin conjugating enzyme |
| Contig9811 | 37.7 | 3.70E-01 | 15/36 (41%) | Aspergillus nidulans | exopolygalacturonase |
| Contig6458 | 32.7 | 8.40E+00 | 19/38 (50%) | Aspergillus nidulans FGSC A4 | hypothetical protein AN0108.2 |
| FQ92HJ001EPUSQ | 106 | 1.00E-03 | 20/49 (40%) | Aspergillus nidulans FGSC A4 | hypothetical protein AN0263.2 |
| FQ4QJ5301E3QTX | 76 | 3.80E+00 | 16/43 (37%) | Aspergillus nidulans FGSC A4 | hypothetical protein AN0276.2 |
| FQ4QJ5301DEMWV | 86 | 2.70E-01 | 16/28 (57%) | Aspergillus nidulans FGSC A4 | hypothetical protein AN0305.2 |
| FQ92HJ001AL5IH | 76 | 3.80E+00 | 15/35 (42%) | Aspergillus nidulans FGSC A4 | hypothetical protein AN1264.2 |
| FQ4QJ5301AE213 | 73 | 8.60E+00 | 14/22 (63%) | Aspergillus nidulans FGSC A4 | hypothetical protein AN2238.2 |
| Contig14438 | 73.2 | 1.00E-11 | 38/94 (40%) | Aspergillus nidulans FGSC A4 | hypothetical protein AN2671.2 |
| Contig7904 | 57.4 | 3.00E-07 | 39/96 (40%) | Aspergillus nidulans FGSC A4 | hypothetical protein AN2833.2 |
| FQ4QJ5301AIDYC | 80 | 1.30E+00 | 20/51 (39%) | Aspergillus nidulans FGSC A4 | hypothetical protein AN2863.2 |
| FQ92HJ001DSLXD | 79 | 1.70E+00 | 16/49 (32%) | Aspergillus nidulans FGSC A4 | hypothetical protein AN3110.2 |
| Contig6563 | 34.3 | 3.00E+00 | 15/30 (50%) | Aspergillus nidulans FGSC A4 | hypothetical protein AN3889.2 |
| FQ92HJ001BLMR1 | 78 | 2.30E+00 | 15/35 (42%) | Aspergillus nidulans FGSC A4 | hypothetical protein AN3980.2 |
| FQ4QJ5301CT4HM | 80 | 1.30E+00 | 14/25 (56%) | Aspergillus nidulans FGSC A4 | hypothetical protein AN4158.2 |
| FQ4QJ5301E3J5Y | 74 | 6.60E+00 | 21/66 (31%) | Aspergillus nidulans FGSC A4 | hypothetical protein AN4460.2 |
| FQ4QJ5301BOYEX | 74 | 6.40E+00 | 24/73 (32%) | Aspergillus nidulans FGSC A4 | hypothetical protein AN4711.2 |
| FQ92HJ001DSTNM | 136 | 4.00E-07 | 27/30 (90%) | Aspergillus nidulans FGSC A4 | hypothetical protein AN5014.2 |
| Contig7461 | 35 | 1.70E+00 | 15/29 (51%) | Aspergillus nidulans FGSC A4 | hypothetical protein AN5066.2 |
| Contig3513 | 45.8 | 1.00E-03 | 21/59 (35%) | Aspergillus nidulans FGSC A4 | hypothetical protein AN5095.2 |
| Contig3868 | 35 | 1.70E+00 | 19/58 (32%) | Aspergillus nidulans FGSC A4 | hypothetical protein AN5139.2 |
| Contig13443 | 35.4 | 1.10E-02 | 17/28 (60%) | Aspergillus nidulans FGSC A4 | hypothetical protein AN5245.2 |
| FQ4QJ5301CJ2A9 | 98 | 1.10E-02 | 22/76 (28%) | Aspergillus nidulans FGSC A4 | hypothetical protein AN5254.2 |
| FQ4QJ5301DALRN | 73 | 8.50E+00 | 18/57 (31%) | Aspergillus nidulans FGSC A4 | hypothetical protein AN5518.2 |
| Contig3621 | 94.4 | 2.00E-18 | 42/76 (55%) | Aspergillus nidulans FGSC A4 | hypothetical protein AN5524.2 |
| FQ92HJ001B7YCF | 89 | 1.20E-01 | 22/81 (27%) | Aspergillus nidulans FGSC A4 | hypothetical protein AN5619.2 |
| FQ4QJ5301BBV1D | 88 | 1.50E-01 | 17/28 (60%) | Aspergillus nidulans FGSC A4 | hypothetical protein AN5655.2 |
| Contig9933 | 33.9 | 3.80E+00 | 24/71 (33%) | Aspergillus nidulans FGSC A4 | hypothetical protein AN5851.2 |
| Contig16591 | 35 | 1.70E+00 | 23/56 (41%) | Aspergillus nidulans FGSC A4 | hypothetical protein AN5931.2 |
| Contig16674 | 33.9 | 3.80E+00 | 24/89 (26%) | Aspergillus nidulans FGSC A4 | hypothetical protein AN5943.2 |
| FQ4QJ5301DZYF3 | 77 | 3.00E+00 | 18/65 (27%) | Aspergillus nidulans FGSC A4 | hypothetical protein AN6052.2 |
| FQ92HJ001A7NDU | 145 | 4.00E-08 | 31/55 (56%) | Aspergillus nidulans FGSC A4 | hypothetical protein AN6057.2 |
| FQ4QJ5301D62Z2 | 76 | 3.80E+00 | 16/42 (38%) | Aspergillus nidulans FGSC A4 | hypothetical protein AN6396.2 |
| Contig16162 | 33.5 | 4.90E+00 | 20/72 (27%) | Aspergillus nidulans FGSC A4 | hypothetical protein AN6782.2 |
| FQ4QJ5301D9KCO | 110 | 4.00E-04 | 22/47 (46%) | Aspergillus nidulans FGSC A4 | hypothetical protein AN6798.2 |
| FQ4QJ5301AS7R3 | 174 | 3.00E-17 | 31/58 (53%) | Aspergillus nidulans FGSC A4 | hypothetical protein AN6798.2 |
| Contig16183 | 87.4 | 3.00E-16 | 40/67 (59%) | Aspergillus nidulans FGSC A4 | hypothetical protein AN7322.2 |
| FQ4QJ5301BPS4K | 90 | 9.00E-02 | 17/27 (62%) | Aspergillus nidulans FGSC A4 | hypothetical protein AN7671.2 |
| FQ4QJ5301DMW00 | 140 | 1.00E-07 | 32/52 (61%) | Aspergillus nidulans FGSC A4 | hypothetical protein AN7845.2 |
| FQ4QJ5301CZP24 | 160 | 7.00E-10 | 36/71 (50%) | Aspergillus nidulans FGSC A4 | hypothetical protein AN7951.2 |
| FQ92HJ001AN83L | 241 | 3.00E-19 | 44/67 (65%) | Aspergillus nidulans FGSC A4 | hypothetical protein AN7955.2 |
| Contig1320 | 32.7 | 8.50E+00 | 18/52 (34%) | Aspergillus nidulans FGSC A4 | hypothetical protein AN9371.2 |
| FQ4QJ5301DRKWA | 125 | 8.00E-06 | 30/70 (42%) | Aspergillus nidulans FGSC A4 | hypothetical protein AN9416.2 |
| Contig13552 | 115 | 1.00E-24 | 62/135 (45%) | Aspergillus nidulans FGSC A4 | hypothetical protein AN9537.2 |
| FQ92HJ001AVVJ9 | 87 | 2.10E-01 | 16/29 (55%) | Aspergillus nidulans FGSC A4 | hypothetical protein AN9537.2 |
| FQ92HJ001BAMJ3 | 146 | 3.00E-08 | 28/29 (96%) | Aspergillus niger | hypothetical protein An01g04920 |
| Contig4137 | 35.4 | 1.30E+00 | 22/67 (32%) | Aspergillus niger | hypothetical protein An01g08110 |
| FQ92HJ001DHOSD | 79 | 1.80E+00 | 18/48 (37%) | Aspergillus niger | hypothetical protein An01g08680 |
| FQ92HJ001AYG0X | 78 | 2.30E+00 | 18/44 (40%) | Aspergillus niger | hypothetical protein An01g08680 |
| FQ4QJ5301AOMNF | 79 | 1.70E+00 | 18/46 (39%) | Aspergillus niger | hypothetical protein An01g08920 |
| FQ92HJ001DF4QR | 85 | 3.50E-01 | 17/43 (39%) | Aspergillus niger | hypothetical protein An01g10170 |
| FQ4QJ5301CV29L | 80 | 1.30E+00 | 17/37 (45%) | Aspergillus niger | hypothetical protein An01g11260 |
| Contig5093 | 32.7 | 8.40E+00 | 18/48 (37%) | Aspergillus niger | hypothetical protein An01g14040 |
| FQ92HJ001BOPXY | 73 | 8.70E+00 | 9/28 (32%) | Aspergillus niger | hypothetical protein An01g14690 |
| Contig10746 | 33.1 | 6.60E+00 | 19/60 (31%) | Aspergillus niger | hypothetical protein An02g01560 |
| Contig1775 | 81.3 | 2.00E-14 | 49/101 (48%) | Aspergillus niger | hypothetical protein An02g03250 |
| FQ92HJ001BH5V4 | 73 | 8.40E+00 | 20/62 (32%) | Aspergillus niger | hypothetical protein An02g04220 |
| FQ4QJ5301DTYEO | 194 | 7.00E-15 | 35/39 (89%) | Aspergillus niger | hypothetical protein An02g07470 |
| FQ4QJ5301EIF1I | 211 | 9.00E-16 | 36/44 (81%) | Aspergillus niger | hypothetical protein An02g09730 |
| Contig8689 | 33.9 | 3.90E+00 | 20/50 (40%) | Aspergillus niger | hypothetical protein An02g13300 |
| Contig3592 | 33.1 | 6.50E+00 | 15/54 (27%) | Aspergillus niger | hypothetical protein An03g01700 |
| Contig16303 | 33.9 | 3.80E+00 | 25/69 (36%) | Aspergillus niger | hypothetical protein An03g02600 |
| FQ4QJ5301BEBYN | 350 | 6.00E-32 | 67/76 (88%) | Aspergillus niger | hypothetical protein An03g04520 |
| FQ4QJ5301BVMOH | 82 | 7.80E-01 | 22/78 (28%) | Aspergillus niger | hypothetical protein An03g04790 |
| FQ4QJ5301CZ2Q2 | 85 | 3.40E-01 | 15/38 (39%) | Aspergillus niger | hypothetical protein An04g03830 |
| FQ92HJ001B8PPR | 80 | 1.30E+00 | 14/42 (33%) | Aspergillus niger | hypothetical protein An04g04630 |
| Contig5294 | 56.2 | 7.00E-07 | 24/34 (70%) | Aspergillus niger | hypothetical protein An04g05750 |
| Contig12259 | 33.1 | 6.50E+00 | 21/53 (39%) | Aspergillus niger | hypothetical protein An04g09840 |
| FQ4QJ5301BH2YV | 76 | 3.80E+00 | 17/38 (44%) | Aspergillus niger | hypothetical protein An07g03005 |
| FQ92HJ001CNXZI | 80 | 1.30E+00 | 18/43 (41%) | Aspergillus niger | hypothetical protein An07g03470 |
| FQ92HJ001CLO2A | 107 | 1.00E-03 | 22/38 (57%) | Aspergillus niger | hypothetical protein An07g06470 |
| Contig12455 | 35.4 | 1.30E+00 | 24/88 (27%) | Aspergillus niger | hypothetical protein An07g06630 |
| Contig13160 | 52 | 1.00E-05 | 22/27 (81%) | Aspergillus niger | hypothetical protein An07g07020 |
| FQ4QJ5301D5S9W | 405 | 3.00E-38 | 76/78 (97%) | Aspergillus niger | hypothetical protein An08g01610 |
| Contig15092 | 35.4 | 1.30E+00 | 23/75 (30%) | Aspergillus niger | hypothetical protein An08g02120 |
| Contig12766 | 38.9 | 1.20E-01 | 36/98 (36%) | Aspergillus niger | hypothetical protein An08g05390 |
| FQ4QJ5301EL7QK | 361 | 3.00E-33 | 64/76 (84%) | Aspergillus niger | hypothetical protein An08g06560 |
| FQ92HJ001CS4AL | 76 | 3.90E+00 | 23/61 (37%) | Aspergillus niger | hypothetical protein An08g07030 |
| FQ4QJ5301BY1R0 | 96 | 2.00E-05 | 16/20 (80%) | Aspergillus niger | hypothetical protein An09g00680 |
| FQ4QJ5301AMPVQ | 75 | 4.90E+00 | 19/49 (38%) | Aspergillus niger | hypothetical protein An09g02720 |
| Contig8321 | 80.5 | 4.00E-14 | 41/86 (47%) | Aspergillus niger | hypothetical protein An11g00260 |
| FQ4QJ5301C08NG | 85 | 3.40E-01 | 16/35 (45%) | Aspergillus niger | hypothetical protein An11g00930 |
| FQ4QJ5301D9DGQ | 108 | 2.00E-09 | 21/23 (91%) | Aspergillus niger | hypothetical protein An11g02380 |
| Contig10503 | 33.5 | 5.00E+00 | 16/47 (34%) | Aspergillus niger | hypothetical protein An11g04050 |
| FQ92HJ001ASF6N | 85 | 3.50E-01 | 17/35 (48%) | Aspergillus niger | hypothetical protein An11g10610 |
| Contig7262 | 35 | 1.70E+00 | 23/80 (28%) | Aspergillus niger | hypothetical protein An12g04030 |
| FQ4QJ5301DBUI6 | 75 | 4.90E+00 | 19/48 (39%) | Aspergillus niger | hypothetical protein An12g06510 |
| FQ4QJ5301DZLN6 | 145 | 5.00E-13 | 25/43 (58%) | Aspergillus niger | hypothetical protein An12g07580 |
| Contig1255 | 34.7 | 2.30E+00 | 14/25 (56%) | Aspergillus niger | hypothetical protein An13g01980 |
| Contig9707 | 34.3 | 2.90E+00 | 15/26 (57%) | Aspergillus niger | hypothetical protein An14g00100 |
| Contig2095 | 33.9 | 3.90E+00 | 18/56 (32%) | Aspergillus niger | hypothetical protein An14g01235 |
| Contig10154 | 35 | 1.70E+00 | 19/54 (35%) | Aspergillus niger | hypothetical protein An14g01630 |
| FQ4QJ5301D7LQJ | 76 | 3.90E+00 | 23/55 (41%) | Aspergillus niger | hypothetical protein An14g03040 |
| Contig15165 | 35 | 1.70E+00 | 21/65 (32%) | Aspergillus niger | hypothetical protein An14g03290 |
| FQ92HJ001CO8MK | 75 | 4.90E+00 | 15/44 (34%) | Aspergillus niger | hypothetical protein An15g02960 |
| Contig10280 | 37 | 7.60E-01 | 21/65 (32%) | Aspergillus niger | hypothetical protein An15g05710 |
| FQ4QJ5301D48BW | 157 | 2.00E-09 | 28/37 (75%) | Aspergillus niger | hypothetical protein An15g07930 |
| FQ4QJ5301AUE91 | 76 | 3.80E+00 | 19/63 (30%) | Aspergillus niger | hypothetical protein An16g00040 |
| FQ4QJ5301EFH0I | 76 | 3.80E+00 | 16/48 (33%) | Aspergillus niger | hypothetical protein An17g00790 |
| Contig11517 | 33.1 | 6.50E+00 | 15/33 (45%) | Aspergillus niger | hypothetical protein An17g01350 |
| FQ92HJ001DHCQL | 76 | 3.90E+00 | 15/45 (33%) | Aspergillus niger | hypothetical protein An18g01380 |
| Contig6382 | 90.1 | 5.00E-17 | 44/50 (88%) | Aspergillus niger | hypothetical protein An18g05680 |
| FQ4QJ5301ARYKC | 75 | 5.00E+00 | 20/55 (36%) | Aspergillus niger | hypothetical protein An18g05730 |
| FQ4QJ5301DW0BX | 137 | 3.00E-07 | 35/77 (45%) | Aspergillus niger | hypothetical protein An18g06710 |
| Contig10963 | 34.7 | 2.30E+00 | 15/43 (34%) | Aspergillus niger | unnamed protein product |
| Contig603 | 106 | 6.00E-22 | 53/66 (80%) | Aspergillus oryzae RIB40 | hypothetical protein |
| Contig1795 | 32.7 | 8.60E+00 | 19/55 (34%) | Aspergillus oryzae RIB40 | hypothetical protein |
| Contig2698 | 68.6 | 1.00E-10 | 32/36 (88%) | Aspergillus oryzae RIB40 | hypothetical protein |
| Contig4502 | 33.5 | 4.90E+00 | 25/58 (43%) | Aspergillus oryzae RIB40 | hypothetical protein |
| Contig7193 | 36.6 | 5.80E-01 | 18/40 (45%) | Aspergillus oryzae RIB40 | hypothetical protein |
| Contig8271 | 34.7 | 2.30E+00 | 20/41 (48%) | Aspergillus oryzae RIB40 | hypothetical protein |
| Contig11017 | 34.7 | 5.00E+00 | 15/51 (29%) | Aspergillus oryzae RIB40 | hypothetical protein |
| Contig11548 | 32.7 | 8.50E+00 | 13/41 (31%) | Aspergillus oryzae RIB40 | hypothetical protein |
| Contig13595 | 33.5 | 5.00E+00 | 15/37 (40%) | Aspergillus oryzae RIB40 | hypothetical protein |
| Contig15619 | 35 | 1.70E+00 | 29/99 (29%) | Aspergillus oryzae RIB40 | hypothetical protein |
| Contig15926 | 36.2 | 7.60E-01 | 24/73 (32%) | Aspergillus oryzae RIB40 | hypothetical protein |
| Contig16262 | 54.3 | 3.00E-06 | 24/28 (85%) | Aspergillus oryzae RIB40 | hypothetical protein |
| Contig16567 | 34.3 | 5.80E+00 | 16/60 (26%) | Aspergillus oryzae RIB40 | hypothetical protein |
| FQ4QJ5301AIHFZ | 394 | 5.00E-37 | 74/86 (86%) | Aspergillus oryzae RIB40 | hypothetical protein |
| FQ4QJ5301DQLRC | 75 | 5.00E+00 | 18/47 (38%) | Aspergillus oryzae RIB40 | hypothetical protein |
| FQ4QJ5301C9YUX | 73 | 8.40E+00 | 23/61 (37%) | Aspergillus oryzae RIB40 | hypothetical protein |
| FQ4QJ5301B3CAM | 217 | 2.00E-16 | 39/77 (50%) | Aspergillus oryzae RIB40 | hypothetical protein |
| FQ4QJ5301EG91C | 80 | 1.30E+00 | 19/43 (44%) | Aspergillus oryzae RIB40 | hypothetical protein |
| FQ4QJ5301CYBG1 | 75 | 4.90E+00 | 15/34 (44%) | Aspergillus oryzae RIB40 | hypothetical protein |
| FQ4QJ5301B3DPH | 182 | 2.00E-12 | 41/53 (77%) | Aspergillus oryzae RIB40 | hypothetical protein |
| FQ4QJ5301BNHA2 | 170 | 5.00E-11 | 37/83 (44%) | Aspergillus oryzae RIB40 | hypothetical protein |
| FQ4QJ5301EQXTN | 73 | 8.70E+00 | 18/55 (32%) | Aspergillus oryzae RIB40 | hypothetical protein |
| FQ4QJ5301CXOVX | 73 | 8.40E+00 | 16/54 (29%) | Aspergillus oryzae RIB40 | hypothetical protein |
| FQ4QJ5301DC1SF | 73 | 8.40E+00 | 20/64 (31%) | Aspergillus oryzae RIB40 | hypothetical protein |
| FQ4QJ5301EPOZA | 300 | 4.00E-26 | 56/66 (84%) | Aspergillus oryzae RIB40 | hypothetical protein |
| FQ4QJ5301BZ8CU | 138 | 3.00E-07 | 27/59 (45%) | Aspergillus oryzae RIB40 | hypothetical protein |
| FQ4QJ5301B5D8C | 146 | 3.00E-08 | 33/77 (42%) | Aspergillus oryzae RIB40 | hypothetical protein |
| FQ4QJ5301COU6N | 75 | 4.20E+00 | 11/20 (55%) | Aspergillus oryzae RIB40 | hypothetical protein |
| FQ92HJ001CP938 | 76 | 3.80E+00 | 23/70 (32%) | Aspergillus oryzae RIB40 | hypothetical protein |
| FQ92HJ001CJ42T | 114 | 1.00E-04 | 22/41 (53%) | Aspergillus oryzae RIB40 | hypothetical protein |
| FQ92HJ001D85V6 | 113 | 2.00E-04 | 24/60 (40%) | Aspergillus oryzae RIB40 | hypothetical protein |
| FQ92HJ001ASPU1 | 73 | 8.60E+00 | 25/73 (34%) | Aspergillus oryzae RIB40 | hypothetical protein |
| FQ92HJ001C1SR8 | 82 | 7.60E-01 | 18/34 (52%) | Aspergillus oryzae RIB40 | hypothetical protein |
| FQ92HJ001DC327 | 73 | 8.60E+00 | 14/24 (58%) | Aspergillus oryzae RIB40 | hypothetical protein |
| FQ92HJ001BHL3K | 78 | 2.20E+00 | 19/49 (38%) | Aspergillus oryzae RIB40 | hypothetical protein |
| FQ92HJ001BR5TZ | 77 | 2.90E+00 | 23/49 (46%) | Aspergillus oryzae RIB40 | hypothetical protein |
| FQ92HJ001BR4J6 | 122 | 2.00E-05 | 22/26 (84%) | Aspergillus oryzae RIB40 | hypothetical protein |
| FQ92HJ001B6PZW | 74 | 6.40E+00 | 14/36 (38%) | Aspergillus oryzae RIB40 | hypothetical protein |
| FQ92HJ001C0ORM | 112 | 3.00E-04 | 24/78 (30%) | Aspergillus oryzae RIB40 | hypothetical protein |
| Contig1833 | 121 | 1.00E-26 | 58/82 (70%) | Aspergillus terreus | 60S ribosome subunit biogenesis protein NIP7 |
| Contig6616 | 80.5 | 2.00E-25 | 40/46 (86%) | Aspergillus terreus | 78 kDa glucose-regulated protein precursor |
| Contig1266 | 115 | 1.00E-24 | 54/56 (96%) | Aspergillus terreus NIH2624 | 60S ribosomal protein L5 |
| FQ4QJ5301CUVH7 | 153 | 2.00E-12 | 26/30 (86%) | Aspergillus terreus NIH2624 | asparagine synthetase 1 |
| FQ4QJ5301BBMKN | 76 | 3.80E+00 | 15/36 (41%) | Aspergillus terreus NIH2624 | chitin synthase 2 |
| Contig7993 | 40.8 | 3.10E-02 | 22/42 (52%) | Aspergillus terreus NIH2624 | conserved hypothetical protein |
| Contig8749 | 32.7 | 8.50E+00 | 13/42 (30%) | Aspergillus terreus NIH2624 | conserved hypothetical protein |
| Contig9003 | 32.7 | 8.40E+00 | 17/57 (29%) | Aspergillus terreus NIH2624 | conserved hypothetical protein |
| Contig11802 | 32.7 | 8.40E+00 | 10/18 (55%) | Aspergillus terreus NIH2624 | conserved hypothetical protein |
| Contig15977 | 36.2 | 7.70E-01 | 32/90 (35%) | Aspergillus terreus NIH2624 | conserved hypothetical protein |
| Contig16612 | 33.1 | 6.50E+00 | 17/57 (29%) | Aspergillus terreus NIH2624 | conserved hypothetical protein |
| FQ4QJ5301CKQEU | 276 | 3.00E-23 | 56/88 (63%) | Aspergillus terreus NIH2624 | conserved hypothetical protein |
| FQ4QJ5301D0VV2 | 187 | 5.00E-13 | 39/72 (54%) | Aspergillus terreus NIH2624 | conserved hypothetical protein |
| FQ4QJ5301AQ07S | 308 | 5.00E-27 | 53/87 (60%) | Aspergillus terreus NIH2624 | conserved hypothetical protein |
| FQ4QJ5301AZ0I0 | 103 | 3.00E-03 | 21/34 (61%) | Aspergillus terreus NIH2624 | conserved hypothetical protein |
| FQ4QJ5301CJ6JA | 186 | 7.00E-13 | 39/70 (55%) | Aspergillus terreus NIH2624 | conserved hypothetical protein |
| FQ4QJ5301DZSXH | 74 | 6.50E+00 | 17/36 (47%) | Aspergillus terreus NIH2624 | conserved hypothetical protein |
| FQ92HJ001CPBD9 | 76 | 3.80E+00 | 25/67 (37%) | Aspergillus terreus NIH2624 | conserved hypothetical protein |
| FQ92HJ001B5NSM | 375 | 8.00E-35 | 71/76 (93%) | Aspergillus terreus NIH2624 | conserved hypothetical protein |
| FQ92HJ001BMOGE | 76 | 3.90E+00 | 17/48 (35%) | Aspergillus terreus NIH2624 | conserved hypothetical protein |
| FQ92HJ001APANH | 190 | 2.00E-13 | 33/55 (60%) | Aspergillus terreus NIH2624 | conserved hypothetical protein |
| FQ4QJ5301EIO2Q | 334 | 5.00E-30 | 67/81 (82%) | Aspergillus terreus NIH2624 | cysteine synthase |
| FQ4QJ5301CUAWG | 170 | 5.00E-11 | 33/48 (68%) | Aspergillus terreus NIH2624 | dihydroxy-acid dehydratase |
| FQ4QJ5301C3ADA | 371 | 2.00E-34 | 70/85 (82%) | Aspergillus terreus NIH2624 | heat shock 70 kDa protein |
| Contig7221 | 174 | 1.00E-42 | 93/121 (76%) | Aspergillus terreus NIH2624 | hypothetical protein ATEG\_00074 |
| Contig11495 | 268 | 2.00E-70 | 134/165 (81%) | Aspergillus terreus NIH2624 | hypothetical protein ATEG\_00074 |
| Contig1473 | 33.1 | 6.60E+00 | 14/35 (40%) | Aspergillus terreus NIH2624 | hypothetical protein ATEG\_05311 |
| FQ92HJ001ASH12 | 79 | 1.70E+00 | 23/54 (42%) | Aspergillus terreus NIH2624 | hypothetical protein ATEG\_05856 |
| Contig1594 | 85.9 | 9.00E-16 | 38/50 (76%) | Aspergillus terreus NIH2624 | lysyl-tRNA synthetase |
| Contig6491 | 35.4 | 1.30E+00 | 21/61 (34%) | Aspergillus terreus NIH2624 | peroxisomal biogenesis factor 6 |
| Contig1704 | 39.3 | 9.30E-02 | 23/67 (34%) | Aspergillus terreus NIH2624 | predicted protein |
| Contig5808 | 35 | 1.70E+00 | 22/88 (25%) | Aspergillus terreus NIH2624 | predicted protein |
| Contig6258 | 38.5 | 1.60E-01 | 24/80 (30%) | Aspergillus terreus NIH2624 | predicted protein |
| Contig13462 | 33.1 | 6.60E+00 | 23/71 (32%) | Aspergillus terreus NIH2624 | predicted protein |
| Contig14308 | 32.7 | 8.50E+00 | 14/50 (28%) | Aspergillus terreus NIH2624 | predicted protein |
| FQ4QJ5301DNXMC | 91 | 6.90E-02 | 18/43 (41%) | Aspergillus terreus NIH2624 | predicted protein |
| FQ4QJ5301C6BP0 | 168 | 8.00E-11 | 32/79 (40%) | Aspergillus terreus NIH2624 | predicted protein |
| FQ92HJ001EI1XL | 117 | 7.00E-05 | 20/47 (42%) | Aspergillus terreus NIH2624 | predicted protein |
| FQ92HJ001ETEOJ | 82 | 7.90E-01 | 16/38 (42%) | Aspergillus terreus NIH2624 | predicted protein |
| FQ92HJ001C3GFY | 74 | 6.50E+00 | 19/61 (31%) | Aspergillus terreus NIH2624 | protein dopey |
| Contig1015 | 33.1 | 6.60E+00 | 15/37 (40%) | Aspergillus terreus NIH2624 | retinal dehydrogenase 2 |
| FQ92HJ001CZ4YZ | 323 | 9.00E-29 | 64/82 (78%) | Aspergillus tubingensis | NADH dehydrogenase subunit 5 |
| FQ92HJ001CSUZ5 | 293 | 3.00E-25 | 54/61 (88%) | Beauveria bassiana | glyceraldehyde-3-phosphate dehydrogenase |
| Contig15968 | 157 | 3.00E-37 | 75/77 (97%) | Beauveria bassiana | putative enolase |
| Contig9012 | 95.9 | 8.00E-19 | 45/58 (77%) | Blastocladiella emersonii | beta-tubulin |
| Contig14868 | 118 | 8.00E-29 | 55/61 (90%) | Blastocladiella emersonii | elongation factor alpha-like protein |
| Contig11648 | 36.2 | 7.70E-01 | 19/54 (35%) | Blastopirellula | ribosomal protein S1-like RNA-binding domain protein |
| FQ92HJ001CU7V4 | 73 | 8.40E+00 | 15/39 (38%) | Blastopirellula marina DSM | hypothetical protein DSM3645\_07955 |
| FQ92HJ001AOPYD | 73 | 8.70E+00 | 18/52 (34%) | Blastopirellula marina DSM 3645 | Cellulose synthase (UDP-forming) |
| FQ92HJ001DFPQT | 73 | 8.50E+00 | 15/46 (32%) | Blastopirellula marina DSM 3645 | DNA polymerase III alpha subunit |
| FQ92HJ001AZ8NK | 77 | 3.00E+00 | 18/46 (39%) | Botryotinia fuckeliana | CND5p |
| Contig12033 | 36.6 | 5.90E-01 | 17/47 (36%) | Botryotinia fuckeliana | transcription regulator PACB |
| Contig16425 | 291 | 2.00E-77 | 143/152 (94%) | Botryotinia fuckeliana B05.10 | 40S ribosomal protein S11 |
| Contig16302 | 253 | 9.00E-66 | 124/133 (93%) | Botryotinia fuckeliana B05.10 | 40S ribosomal protein S18 |
| Contig7680 | 50.8 | 3.00E-05 | 22/27 (81%) | Botryotinia fuckeliana B05.10 | 60S ribosomal protein L10 |
| Contig5094 | 270 | 2.00E-71 | 116/137 (84%) | Botryotinia fuckeliana B05.10 | acyl-CoA desaturase |
| FQ4QJ5301C77XF | 251 | 2.00E-20 | 50/72 (69%) | Botryotinia fuckeliana B05.10 | ATP phosphoribosyltransferase |
| FQ4QJ5301B2F73 | 205 | 4.00E-15 | 40/54 (74%) | Botryotinia fuckeliana B05.10 | conserved hypothetical protein |
| FQ4QJ5301EO4LO | 355 | 2.00E-32 | 64/79 (81%) | Botryotinia fuckeliana B05.10 | conserved hypothetical protein |
| FQ92HJ001C559Q | 78 | 4.00E-04 | 17/32 (53%) | Botryotinia fuckeliana B05.10 | epoxide hydrolase |
| FQ4QJ5301BHB2M | 331 | 1.00E-29 | 60/77 (77%) | Botryotinia fuckeliana B05.10 | hypothetical protein BC1G\_00027 |
| Contig3590 | 105 | 4.00E-26 | 59/141 (41%) | Botryotinia fuckeliana B05.10 | hypothetical protein BC1G\_00103 |
| Contig10490 | 35.4 | 1.30E+00 | 28/95 (29%) | Botryotinia fuckeliana B05.10 | hypothetical protein BC1G\_00772 |
| Contig6282 | 33.9 | 3.90E+00 | 14/31 (45%) | Botryotinia fuckeliana B05.10 | hypothetical protein BC1G\_01108 |
| Contig4725 | 36.6 | 5.90E-01 | 21/57 (36%) | Botryotinia fuckeliana B05.10 | hypothetical protein BC1G\_01212 |
| Contig3318 | 32.7 | 8.50E+00 | 15/41 (36%) | Botryotinia fuckeliana B05.10 | hypothetical protein BC1G\_01221 |
| FQ4QJ5301DV56U | 303 | 2.00E-26 | 55/80 (68%) | Botryotinia fuckeliana B05.10 | hypothetical protein BC1G\_01725 |
| FQ4QJ5301EGMF9 | 351 | 5.00E-32 | 62/89 (69%) | Botryotinia fuckeliana B05.10 | hypothetical protein BC1G\_01937 |
| Contig4720 | 36.6 | 5.80E-01 | 23/51 (45%) | Botryotinia fuckeliana B05.10 | hypothetical protein BC1G\_03594 |
| FQ4QJ5301A6BEH | 97 | 1.40E-02 | 18/46 (39%) | Botryotinia fuckeliana B05.10 | hypothetical protein BC1G\_04630 |
| FQ92HJ001DKIVH | 74 | 6.50E+00 | 17/51 (33%) | Botryotinia fuckeliana B05.10 | hypothetical protein BC1G\_04813 |
| FQ92HJ001DJ5E1 | 77 | 2.90E+00 | 21/47 (44%) | Botryotinia fuckeliana B05.10 | hypothetical protein BC1G\_05097 |
| FQ4QJ5301B0NQZ | 136 | 4.00E-07 | 23/27 (85%) | Botryotinia fuckeliana B05.10 | hypothetical protein BC1G\_05123 |
| Contig4575 | 101 | 1.00E-20 | 47/78 (60%) | Botryotinia fuckeliana B05.10 | hypothetical protein BC1G\_05254 |
| FQ4QJ5301C4GJX | 363 | 2.00E-33 | 63/81 (77%) | Botryotinia fuckeliana B05.10 | hypothetical protein BC1G\_05888 |
| Contig885 | 147 | 3.00E-34 | 67/82 (81%) | Botryotinia fuckeliana B05.10 | hypothetical protein BC1G\_06103 |
| FQ92HJ001AQ95P | 73 | 8.60E+00 | 14/50 (28%) | Botryotinia fuckeliana B05.10 | hypothetical protein BC1G\_06167 |
| Contig664 | 137 | 2.00E-31 | 61/76 (80%) | Botryotinia fuckeliana B05.10 | hypothetical protein BC1G\_06301 |
| Contig11241 | 34.3 | 2.90E+00 | 20/53 (37%) | Botryotinia fuckeliana B05.10 | hypothetical protein BC1G\_06725 |
| FQ4QJ5301AHPHM | 78 | 2.20E+00 | 29/80 (36%) | Botryotinia fuckeliana B05.10 | hypothetical protein BC1G\_06959 |
| FQ4QJ5301E2QAZ | 163 | 3.00E-10 | 31/32 (96%) | Botryotinia fuckeliana B05.10 | hypothetical protein BC1G\_07225 |
| Contig10917 | 33.5 | 4.90E+00 | 17/40 (42%) | Botryotinia fuckeliana B05.10 | hypothetical protein BC1G\_07270 |
| Contig5029 | 47 | 5.00E-07 | 19/38 (50%) | Botryotinia fuckeliana B05.10 | hypothetical protein BC1G\_07345 |
| FQ92HJ001A52O3 | 80 | 1.30E+00 | 16/29 (55%) | Botryotinia fuckeliana B05.10 | hypothetical protein BC1G\_07459 |
| FQ4QJ5301B5XLW | 73 | 8.60E+00 | 23/71 (32%) | Botryotinia fuckeliana B05.10 | hypothetical protein BC1G\_08551 |
| Contig12476 | 33.1 | 6.60E+00 | 23/75 (30%) | Botryotinia fuckeliana B05.10 | hypothetical protein BC1G\_08650 |
| Contig14424 | 105 | 1.00E-21 | 45/60 (75%) | Botryotinia fuckeliana B05.10 | hypothetical protein BC1G\_08946 |
| FQ4QJ5301DZR12 | 78 | 2.20E+00 | 18/60 (30%) | Botryotinia fuckeliana B05.10 | hypothetical protein BC1G\_10559 |
| FQ92HJ001CX91F | 238 | 6.00E-19 | 43/66 (65%) | Botryotinia fuckeliana B05.10 | hypothetical protein BC1G\_10611 |
| FQ4QJ5301AS94X | 88 | 1.60E-01 | 17/33 (51%) | Botryotinia fuckeliana B05.10 | hypothetical protein BC1G\_10816 |
| FQ92HJ001CWBZ9 | 84 | 4.50E-01 | 24/64 (37%) | Botryotinia fuckeliana B05.10 | hypothetical protein BC1G\_10820 |
| FQ4QJ5301C32XO | 182 | 2.00E-12 | 40/68 (58%) | Botryotinia fuckeliana B05.10 | hypothetical protein BC1G\_10825 |
| FQ92HJ001AR1H3 | 119 | 4.00E-05 | 27/45 (60%) | Botryotinia fuckeliana B05.10 | hypothetical protein BC1G\_10864 |
| FQ4QJ5301CB09P | 346 | 2.00E-31 | 61/80 (76%) | Botryotinia fuckeliana B05.10 | hypothetical protein BC1G\_11034 |
| FQ92HJ001C7D3F | 173 | 2.00E-11 | 32/91 (35%) | Botryotinia fuckeliana B05.10 | hypothetical protein BC1G\_11081 |
| FQ4QJ5301ENNQU | 401 | 8.00E-38 | 75/86 (87%) | Botryotinia fuckeliana B05.10 | hypothetical protein BC1G\_11096 |
| FQ4QJ5301A1WQI | 106 | 8.00E-06 | 20/23 (86%) | Botryotinia fuckeliana B05.10 | hypothetical protein BC1G\_11096 |
| FQ4QJ5301EIN07 | 73 | 8.50E+00 | 13/22 (59%) | Botryotinia fuckeliana B05.10 | hypothetical protein BC1G\_11305 |
| FQ4QJ5301DHUVW | 310 | 3.00E-27 | 53/77 (68%) | Botryotinia fuckeliana B05.10 | hypothetical protein BC1G\_11320 |
| FQ4QJ5301B5BSH | 78 | 4.00E-03 | 20/43 (46%) | Botryotinia fuckeliana B05.10 | hypothetical protein BC1G\_11367 |
| Contig7225 | 127 | 7.00E-28 | 63/103 (61%) | Botryotinia fuckeliana B05.10 | hypothetical protein BC1G\_11661 |
| FQ92HJ001BV6SZ | 76 | 3.90E+00 | 15/38 (39%) | Botryotinia fuckeliana B05.10 | hypothetical protein BC1G\_12409 |
| FQ4QJ5301BTW3W | 133 | 9.00E-07 | 25/63 (39%) | Botryotinia fuckeliana B05.10 | hypothetical protein BC1G\_12600 |
| FQ4QJ5301CIKIZ | 131 | 2.00E-06 | 32/73 (43%) | Botryotinia fuckeliana B05.10 | hypothetical protein BC1G\_12639 |
| FQ4QJ5301CAXE3 | 111 | 3.00E-04 | 25/66 (37%) | Botryotinia fuckeliana B05.10 | hypothetical protein BC1G\_13029 |
| FQ92HJ001E4HAD | 77 | 2.90E+00 | 19/43 (44%) | Botryotinia fuckeliana B05.10 | hypothetical protein BC1G\_13247 |
| FQ4QJ5301DD6CC | 163 | 3.00E-10 | 33/77 (42%) | Botryotinia fuckeliana B05.10 | hypothetical protein BC1G\_13297 |
| FQ4QJ5301DEEAA | 86 | 2.70E-01 | 18/31 (58%) | Botryotinia fuckeliana B05.10 | hypothetical protein BC1G\_13297 |
| Contig4786 | 98.6 | 1.00E-19 | 45/71 (63%) | Botryotinia fuckeliana B05.10 | hypothetical protein BC1G\_13615 |
| Contig12780 | 35 | 1.70E+00 | 15/41 (36%) | Botryotinia fuckeliana B05.10 | hypothetical protein BC1G\_13624 |
| Contig9032 | 126 | 4.00E-28 | 60/81 (74%) | Botryotinia fuckeliana B05.10 | hypothetical protein BC1G\_13851 |
| FQ4QJ5301BNADV | 146 | 1.00E-18 | 30/45 (66%) | Botryotinia fuckeliana B05.10 | hypothetical protein BC1G\_13945 |
| FQ92HJ001DWT2C | 76 | 3.90E+00 | 13/33 (39%) | Botryotinia fuckeliana B05.10 | hypothetical protein BC1G\_14814 |
| Contig4688 | 52.8 | 8.00E-06 | 28/47 (59%) | Botryotinia fuckeliana B05.10 | hypothetical protein BC1G\_15349 |
| Contig12285 | 70.1 | 5.00E-11 | 33/68 (48%) | Botryotinia fuckeliana B05.10 | hypothetical protein BC1G\_15605 |
| Contig598 | 32.7 | 8.40E+00 | 16/40 (40%) | Botryotinia fuckeliana B05.10 | predicted protein |
| Contig6591 | 35 | 1.70E+00 | 16/47 (34%) | Botryotinia fuckeliana B05.10 | predicted protein |
| Contig15811 | 35 | 4.20E+00 | 22/61 (36%) | Botryotinia fuckeliana B05.10 | predicted protein |
| FQ4QJ5301D358J | 77 | 3.00E+00 | 15/30 (50%) | Botryotinia fuckeliana B05.10 | predicted protein |
| FQ4QJ5301EI9JA | 70 | 1.80E-01 | 15/32 (46%) | Botryotinia fuckeliana B05.10 | predicted protein |
| FQ4QJ5301AYTWW | 86 | 2.70E-01 | 20/52 (38%) | Botryotinia fuckeliana B05.10 | predicted protein |
| FQ92HJ001DD942 | 86 | 2.70E-01 | 28/87 (32%) | Botryotinia fuckeliana B05.10 | predicted protein |
| FQ92HJ001EYNGE | 74 | 6.50E+00 | 13/42 (30%) | Botryotinia fuckeliana B05.10 | predicted protein |
| FQ4QJ5301ECS0T | 183 | 1.00E-12 | 35/44 (79%) | Botryotinia fuckeliana B05.10 | translation initiation factor eIF3 |
| FQ4QJ5301EGKHW | 73 | 8.70E+00 | 14/40 (35%) | Candida albicans | dual specificity protein tyrosine phosphatase |
| Contig15531 | 35 | 1.70E+00 | 19/60 (31%) | Candida albicans | G1 cyclin |
| FQ92HJ001COE2I | 75 | 5.00E+00 | 18/43 (41%) | Candida albicans SC5314 | 5'-3' exoribonuclease |
| FQ4QJ5301BOIP2 | 78 | 2.30E+00 | 20/56 (35%) | Candida albicans SC5314 | bud site selection protein |
| Contig16511 | 34.7 | 2.20E+00 | 16/52 (30%) | Candida albicans SC5314 | GABA-specific transport protein |
| Contig194 | 33.1 | 6.60E+00 | 16/35 (45%) | Candida albicans SC5314 | GCN4 translational activator |
| FQ92HJ001EKC19 | 81 | 1.00E+00 | 22/56 (39%) | Candida albicans SC5314 | hypothetical protein CaO19.1292 |
| FQ4QJ5301AK127 | 73 | 8.60E+00 | 22/76 (28%) | Candida albicans SC5314 | hypothetical protein CaO19.3555 |
| Contig9502 | 36.2 | 7.80E-01 | 16/50 (32%) | Candida albicans SC5314 | hypothetical protein CaO19.4487 |
| FQ4QJ5301D6UQU | 80 | 1.30E+00 | 30/94 (31%) | Candida albicans SC5314 | hypothetical protein CaO19.5177 |
| Contig4360 | 33.1 | 6.60E+00 | 25/76 (32%) | Candida albicans SC5314 | hypothetical protein CaO19.6157 |
| Contig6102 | 41.6 | 1.80E-02 | 21/74 (28%) | Candida albicans SC5314 | hypothetical protein CaO19.7169 |
| FQ92HJ001C5G0R | 73 | 8.60E+00 | 18/52 (34%) | Candida albicans SC5314 | hypothetical protein CaO19.7728 |
| Contig972 | 33.5 | 4.90E+00 | 15/56 (26%) | Candida albicans SC5314 | hypothetical protein CaO19.9380 |
| FQ92HJ001EOESZ | 66 | 6.20E+00 | 12/24 (50%) | Candida albicans SC5314 | hypothetical protein CaO19.9463 |
| FQ4QJ5301D4YEK | 76 | 3.90E+00 | 15/39 (38%) | Candida albicans SC5314 | hypothetical protein CaO19\_10014 |
| Contig9728 | 33.9 | 7.50E+00 | 23/74 (31%) | Candida albicans SC5314 | hypothetical protein CaO19\_11253 |
| Contig452 | 34.3 | 2.90E+00 | 17/58 (29%) | Candida albicans SC5314 | hypothetical protein CaO19\_1995 |
| Contig12241 | 35 | 2.60E+00 | 20/40 (50%) | Candida albicans SC5314 | hypothetical protein CaO19\_2005 |
| FQ92HJ001AT5WP | 78 | 2.20E+00 | 14/38 (36%) | Candida albicans SC5314 | hypothetical protein CaO19\_3046 |
| FQ92HJ001CVZKN | 77 | 2.90E+00 | 18/59 (30%) | Candida albicans SC5314 | hypothetical protein CaO19\_5755 |
| FQ4QJ5301D48HO | 84 | 4.60E-01 | 20/50 (40%) | Candida albicans SC5314 | hypothetical protein CaO19\_5760 |
| Contig16348 | 38.1 | 7.10E-01 | 27/87 (31%) | Candida albicans SC5314 | hypothetical protein CaO19\_5935 |
| Contig15470 | 35.4 | 3.70E+00 | 19/55 (34%) | Candida albicans SC5314 | hypothetical protein CaO19\_6805 |
| Contig14146 | 33.5 | 6.50E+00 | 19/63 (30%) | Candida albicans SC5314 | mitochondrial Complex I, subunit 1 |
| Contig7951 | 34.3 | 2.90E+00 | 15/41 (36%) | Candida albicans SC5314 | oligosaccharyl transferase |
| FQ4QJ5301AW3F5 | 80 | 1.30E+00 | 15/32 (46%) | Candida albicans SC5314 | regulator of salt tolerance |
| Contig8821 | 38.5 | 1.50E-01 | 25/81 (30%) | Candida albicans SC5314 | spindle pole body-associated protein |
| FQ4QJ5301B1E87 | 78 | 2.30E+00 | 17/62 (27%) | Candida albicans SC5314 | transcription factor |
| Contig341 | 32.7 | 8.50E+00 | 19/41 (46%) | Candida glabrata | unnamed protein product |
| Contig5943 | 96.7 | 1.00E-26 | 47/52 (90%) | Candida glabrata | unnamed protein product |
| Contig6485 | 34.7 | 2.20E+00 | 15/40 (37%) | Candida glabrata | unnamed protein product |
| Contig7263 | 119 | 5.00E-26 | 54/74 (72%) | Candida glabrata | unnamed protein product |
| Contig8582 | 33.5 | 5.00E+00 | 22/53 (41%) | Candida glabrata | unnamed protein product |
| Contig10413 | 33.9 | 3.90E+00 | 13/41 (31%) | Candida glabrata | unnamed protein product |
| Contig10798 | 45.8 | 1.00E-03 | 17/20 (85%) | Candida glabrata | unnamed protein product |
| Contig10881 | 35.4 | 1.50E+00 | 16/48 (33%) | Candida glabrata | unnamed protein product |
| Contig15085 | 33.5 | 5.00E+00 | 11/28 (39%) | Candida glabrata | unnamed protein product |
| Contig15304 | 35.4 | 1.30E+00 | 17/50 (34%) | Candida glabrata | unnamed protein product |
| Contig16325 | 35.8 | 1.00E+00 | 33/104 (31%) | Candida glabrata | unnamed protein product |
| FQ4QJ5301EG107 | 82 | 7.70E-01 | 15/38 (39%) | Candida glabrata | unnamed protein product |
| FQ4QJ5301EM3A6 | 80 | 1.30E+00 | 12/22 (54%) | Candida glabrata | unnamed protein product |
| FQ92HJ001EOU44 | 75 | 5.00E+00 | 18/59 (30%) | Candida glabrata | unnamed protein product |
| FQ92HJ001BGZEG | 74 | 6.50E+00 | 16/38 (42%) | Candida glabrata | unnamed protein product |
| FQ92HJ001CNQOL | 75 | 5.10E+00 | 18/48 (37%) | Candida glabrata | unnamed protein product |
| FQ92HJ001DQ78B | 76 | 3.80E+00 | 19/57 (33%) | Candida glabrata | unnamed protein product |
| FQ92HJ001CMUC4 | 76 | 3.80E+00 | 15/31 (48%) | Candida glabrata | unnamed protein product |
| FQ92HJ001C1ZMF | 73 | 8.40E+00 | 12/29 (41%) | Candida glabrata | unnamed protein product |
| FQ92HJ001EP5P0 | 81 | 1.00E+00 | 17/37 (45%) | Candida glabrata | unnamed protein product |
| FQ92HJ001AIKZ5 | 75 | 5.10E+00 | 21/60 (35%) | Candida glabrata | unnamed protein product |
| FQ92HJ001BKMOB | 73 | 8.70E+00 | 20/46 (43%) | Candida glabrata CBS138 | hypothetical protein CAGL0A00913g |
| Contig16138 | 33.9 | 3.80E+00 | 19/61 (31%) | Candida glabrata CBS138 | hypothetical protein CAGL0A04455g |
| FQ4QJ5301CQEDP | 73 | 8.50E+00 | 21/60 (35%) | Candida glabrata CBS138 | hypothetical protein CAGL0M05027g |
| Contig12618 | 36.6 | 5.80E-01 | 22/60 (36%) | Candida glabrata CBS138 | hypothetical protein CAGL0M06919g |
| Contig5843 | 37.7 | 2.60E-01 | 20/72 (27%) | Candida glabrata CBS138 | hypothetical protein CAGL0M13035g |
| FQ92HJ001D21G9 | 139 | 2.00E-07 | 24/34 (70%) | Candida glabrata CBS138 | hypothetical protein CAGL0M13277g |
| Contig10801 | 65.1 | 1.00E-16 | 28/45 (62%) | Chaetomium | ATP synthase gamma chain, mitochondrial precursor |
| Contig3231 | 186 | 4.00E-46 | 89/106 (83%) | Chaetomium | mitochondrial carrier protein YHM1/SHM1, putative |
| Contig12218 | 160 | 3.00E-38 | 77/94 (81%) | Chaetomium globosum | conserved hypothetical protein |
| Contig14955 | 95.9 | 8.00E-19 | 43/59 (72%) | Chaetomium globosum | conserved hypothetical protein |
| Contig11260 | 112 | 8.00E-24 | 51/64 (79%) | Chaetomium globosum | HIT family protein 1 |
| Contig5375 | 132 | 1.00E-29 | 64/114 (56%) | Chaetomium globosum | probable peroxisomal membrane protein |
| Contig7179 | 80.5 | 2.00E-31 | 39/45 (86%) | Chaetomium globosum CBS | arg-6 protein, mitochondrial precursor |
| Contig15607 | 58.9 | 1.00E-07 | 33/44 (75%) | Chaetomium globosum CBS 148.51 | 40S ribosomal protein S14 |
| Contig818 | 150 | 9.00E-51 | 73/84 (86%) | Chaetomium globosum CBS 148.51 | 40S ribosomal protein S4 |
| Contig9320 | 128 | 1.00E-28 | 58/65 (89%) | Chaetomium globosum CBS 148.51 | 40S ribosomal protein S4 |
| Contig9760 | 123 | 5.00E-27 | 59/65 (90%) | Chaetomium globosum CBS 148.51 | 60S ribosomal protein L12 |
| Contig16113 | 57.4 | 3.00E-07 | 26/27 (96%) | Chaetomium globosum CBS 148.51 | 60S ribosomal protein L3 |
| Contig16484 | 174 | 2.00E-42 | 85/95 (89%) | Chaetomium globosum CBS 148.51 | 60s ribosomal protein L31 |
| Contig11021 | 77 | 4.00E-13 | 38/41 (92%) | Chaetomium globosum CBS 148.51 | 60S ribosomal protein L32 |
| FQ4QJ5301B7S56 | 376 | 6.00E-35 | 73/84 (86%) | Chaetomium globosum CBS 148.51 | ADP,ATP carrier protein |
| Contig3638 | 132 | 8.00E-30 | 64/66 (96%) | Chaetomium globosum CBS 148.51 | cell cycle control protein-related |
| Contig3964 | 45.4 | 6.00E-09 | 22/38 (57%) | Chaetomium globosum CBS 148.51 | conserved hypothetical protein |
| Contig4593 | 181 | 2.00E-44 | 87/108 (80%) | Chaetomium globosum CBS 148.51 | conserved hypothetical protein |
| Contig6506 | 118 | 2.00E-25 | 55/80 (68%) | Chaetomium globosum CBS 148.51 | conserved hypothetical protein |
| Contig7947 | 141 | 5.00E-49 | 69/95 (72%) | Chaetomium globosum CBS 148.51 | conserved hypothetical protein |
| FQ4QJ5301BN9NW | 165 | 2.00E-10 | 32/35 (91%) | Chaetomium globosum CBS 148.51 | conserved hypothetical protein |
| FQ4QJ5301AYSC0 | 222 | 5.00E-17 | 45/63 (71%) | Chaetomium globosum CBS 148.51 | conserved hypothetical protein |
| FQ4QJ5301BUNN0 | 306 | 8.00E-27 | 56/79 (70%) | Chaetomium globosum CBS 148.51 | conserved hypothetical protein |
| FQ4QJ5301A3IBV | 208 | 2.00E-30 | 38/49 (77%) | Chaetomium globosum CBS 148.51 | conserved hypothetical protein |
| FQ4QJ5301EGZV8 | 238 | 6.00E-19 | 54/89 (60%) | Chaetomium globosum CBS 148.51 | conserved hypothetical protein |
| FQ4QJ5301D8R62 | 273 | 6.00E-23 | 54/58 (93%) | Chaetomium globosum CBS 148.51 | conserved hypothetical protein |
| FQ4QJ5301EXU5K | 234 | 2.00E-18 | 47/53 (88%) | Chaetomium globosum CBS 148.51 | conserved hypothetical protein |
| FQ4QJ5301CNSDX | 224 | 3.00E-17 | 43/66 (65%) | Chaetomium globosum CBS 148.51 | conserved hypothetical protein |
| FQ4QJ5301DPEVN | 297 | 9.00E-26 | 54/66 (81%) | Chaetomium globosum CBS 148.51 | conserved hypothetical protein |
| FQ4QJ5301B8KZZ | 99 | 8.00E-03 | 19/19 (100%) | Chaetomium globosum CBS 148.51 | conserved hypothetical protein |
| FQ92HJ001A7F9T | 110 | 4.00E-04 | 21/26 (80%) | Chaetomium globosum CBS 148.51 | conserved hypothetical protein |
| FQ92HJ001CBGRG | 226 | 2.00E-17 | 42/47 (89%) | Chaetomium globosum CBS 148.51 | conserved hypothetical protein |
| FQ92HJ001D2DJ8 | 127 | 1.00E-10 | 24/29 (82%) | Chaetomium globosum CBS 148.51 | conserved hypothetical protein |
| FQ92HJ001B3CG9 | 122 | 1.00E-13 | 24/25 (96%) | Chaetomium globosum CBS 148.51 | conserved hypothetical protein |
| Contig10249 | 199 | 7.00E-50 | 92/105 (87%) | Chaetomium globosum CBS 148.51 | cytochrome c |
| Contig15414 | 197 | 3.00E-49 | 92/105 (87%) | Chaetomium globosum CBS 148.51 | cytochrome c |
| Contig4471 | 41.2 | 2.40E-02 | 20/25 (80%) | Chaetomium globosum CBS 148.51 | DNA mismatch repair protein msh-2 |
| Contig13657 | 167 | 3.00E-40 | 80/86 (93%) | Chaetomium globosum CBS 148.51 | heat shock 70 kDa protein |
| Contig2282 | 109 | 1.00E-28 | 51/84 (60%) | Chaetomium globosum CBS 148.51 | hypothetical protein CHGG\_00044 |
| FQ4QJ5301D6FDU | 113 | 2.00E-07 | 22/43 (51%) | Chaetomium globosum CBS 148.51 | hypothetical protein CHGG\_00081 |
| Contig2332 | 28.1 | 7.40E+00 | 15/39 (38%) | Chaetomium globosum CBS 148.51 | hypothetical protein CHGG\_00127 |
| Contig2396 | 54.3 | 3.00E-06 | 26/61 (42%) | Chaetomium globosum CBS 148.51 | hypothetical protein CHGG\_00206 |
| Contig11971 | 33.9 | 3.90E+00 | 20/43 (46%) | Chaetomium globosum CBS 148.51 | hypothetical protein CHGG\_00263 |
| FQ4QJ5301DXSBL | 74 | 6.50E+00 | 14/29 (48%) | Chaetomium globosum CBS 148.51 | hypothetical protein CHGG\_00278 |
| Contig13617 | 34.7 | 2.30E+00 | 13/26 (50%) | Chaetomium globosum CBS 148.51 | hypothetical protein CHGG\_00303 |
| Contig929 | 45.1 | 2.00E-03 | 21/40 (52%) | Chaetomium globosum CBS 148.51 | hypothetical protein CHGG\_00358 |
| Contig9773 | 49.3 | 9.00E-05 | 28/76 (36%) | Chaetomium globosum CBS 148.51 | hypothetical protein CHGG\_00358 |
| FQ4QJ5301BM9DZ | 190 | 2.00E-14 | 36/53 (67%) | Chaetomium globosum CBS 148.51 | hypothetical protein CHGG\_00449 |
| FQ92HJ001A5PHD | 387 | 3.00E-36 | 72/82 (87%) | Chaetomium globosum CBS 148.51 | hypothetical protein CHGG\_00695 |
| FQ92HJ001B5GUW | 117 | 7.00E-05 | 30/85 (35%) | Chaetomium globosum CBS 148.51 | hypothetical protein CHGG\_00913 |
| FQ4QJ5301BO91P | 151 | 8.00E-09 | 28/33 (84%) | Chaetomium globosum CBS 148.51 | hypothetical protein CHGG\_00983 |
| FQ4QJ5301CGD08 | 258 | 3.00E-21 | 54/76 (71%) | Chaetomium globosum CBS 148.51 | hypothetical protein CHGG\_01082 |
| FQ4QJ5301CDVI5 | 233 | 2.00E-18 | 45/59 (76%) | Chaetomium globosum CBS 148.51 | hypothetical protein CHGG\_01228 |
| FQ4QJ5301CJIXB | 93 | 4.10E-02 | 16/44 (36%) | Chaetomium globosum CBS 148.51 | hypothetical protein CHGG\_01365 |
| Contig13633 | 33.5 | 5.00E+00 | 14/28 (50%) | Chaetomium globosum CBS 148.51 | hypothetical protein CHGG\_01382 |
| FQ4QJ5301DJC8P | 190 | 2.00E-13 | 40/80 (50%) | Chaetomium globosum CBS 148.51 | hypothetical protein CHGG\_01464 |
| FQ92HJ001EQ2GX | 73 | 8.40E+00 | 20/56 (35%) | Chaetomium globosum CBS 148.51 | hypothetical protein CHGG\_01464 |
| Contig138 | 140 | 3.00E-32 | 68/95 (71%) | Chaetomium globosum CBS 148.51 | hypothetical protein CHGG\_01486 |
| Contig16809 | 115 | 2.00E-24 | 54/81 (66%) | Chaetomium globosum CBS 148.51 | hypothetical protein CHGG\_01527 |
| FQ92HJ001DEMS9 | 73 | 8.50E+00 | 19/41 (46%) | Chaetomium globosum CBS 148.51 | hypothetical protein CHGG\_01542 |
| FQ4QJ5301A5HSR | 176 | 1.00E-11 | 42/82 (51%) | Chaetomium globosum CBS 148.51 | hypothetical protein CHGG\_01555 |
| Contig2979 | 35.8 | 1.00E+00 | 20/36 (55%) | Chaetomium globosum CBS 148.51 | hypothetical protein CHGG\_01597 |
| FQ4QJ5301D94AR | 194 | 8.00E-14 | 41/73 (56%) | Chaetomium globosum CBS 148.51 | hypothetical protein CHGG\_01683 |
| Contig5122 | 100 | 9.00E-27 | 46/58 (79%) | Chaetomium globosum CBS 148.51 | hypothetical protein CHGG\_01773 |
| FQ4QJ5301DB62K | 115 | 1.00E-04 | 27/50 (54%) | Chaetomium globosum CBS 148.51 | hypothetical protein CHGG\_01840 |
| FQ4QJ5301ETR6Z | 220 | 8.00E-17 | 42/49 (85%) | Chaetomium globosum CBS 148.51 | hypothetical protein CHGG\_01885 |
| FQ4QJ5301AW1X4 | 178 | 6.00E-12 | 35/75 (46%) | Chaetomium globosum CBS 148.51 | hypothetical protein CHGG\_02135 |
| FQ92HJ001C8TP5 | 77 | 3.00E+00 | 20/55 (36%) | Chaetomium globosum CBS 148.51 | hypothetical protein CHGG\_02270 |
| FQ92HJ001BEWMD | 175 | 1.00E-11 | 36/71 (50%) | Chaetomium globosum CBS 148.51 | hypothetical protein CHGG\_02293 |
| FQ92HJ001B0O39 | 68 | 2.90E+00 | 13/28 (46%) | Chaetomium globosum CBS 148.51 | hypothetical protein CHGG\_02312 |
| FQ92HJ001CD4ET | 279 | 1.00E-23 | 51/75 (68%) | Chaetomium globosum CBS 148.51 | hypothetical protein CHGG\_02316 |
| Contig8131 | 92.8 | 7.00E-18 | 43/56 (76%) | Chaetomium globosum CBS 148.51 | hypothetical protein CHGG\_02339 |
| FQ4QJ5301D1J3A | 80 | 8.20E-02 | 14/15 (93%) | Chaetomium globosum CBS 148.51 | hypothetical protein CHGG\_02438 |
| FQ92HJ001EPMZC | 82 | 7.70E-01 | 23/59 (38%) | Chaetomium globosum CBS 148.51 | hypothetical protein CHGG\_02600 |
| Contig11272 | 36.6 | 6.00E-01 | 34/118 (28%) | Chaetomium globosum CBS 148.51 | hypothetical protein CHGG\_02677 |
| FQ4QJ5301B5ZR6 | 396 | 3.00E-37 | 78/85 (91%) | Chaetomium globosum CBS 148.51 | hypothetical protein CHGG\_02831 |
| Contig1502 | 34.3 | 2.90E+00 | 20/66 (30%) | Chaetomium globosum CBS 148.51 | hypothetical protein CHGG\_02862 |
| Contig11471 | 60.1 | 5.00E-08 | 22/40 (55%) | Chaetomium globosum CBS 148.51 | hypothetical protein CHGG\_02884 |
| FQ4QJ5301A3QX5 | 108 | 7.00E-04 | 22/38 (57%) | Chaetomium globosum CBS 148.51 | hypothetical protein CHGG\_02884 |
| FQ4QJ5301CBKAR | 262 | 1.00E-21 | 50/72 (69%) | Chaetomium globosum CBS 148.51 | hypothetical protein CHGG\_02966 |
| FQ4QJ5301CM3N7 | 75 | 5.10E+00 | 12/19 (63%) | Chaetomium globosum CBS 148.51 | hypothetical protein CHGG\_02969 |
| Contig10274 | 38.9 | 1.20E-01 | 21/34 (61%) | Chaetomium globosum CBS 148.51 | hypothetical protein CHGG\_03010 |
| FQ4QJ5301B9949 | 242 | 2.00E-36 | 46/46 (100%) | Chaetomium globosum CBS 148.51 | hypothetical protein CHGG\_03010 |
| FQ92HJ001DT5Y9 | 77 | 3.00E+00 | 20/62 (32%) | Chaetomium globosum CBS 148.51 | hypothetical protein CHGG\_03083 |
| FQ4QJ5301ANDN2 | 216 | 2.00E-16 | 39/50 (78%) | Chaetomium globosum CBS 148.51 | hypothetical protein CHGG\_03226 |
| Contig3434 | 73.9 | 1.00E-11 | 58/201 (28%) | Chaetomium globosum CBS 148.51 | hypothetical protein CHGG\_03236 |
| FQ4QJ5301D4SAY | 97 | 1.40E-02 | 17/17 (100%) | Chaetomium globosum CBS 148.51 | hypothetical protein CHGG\_03287 |
| Contig11208 | 122 | 6.00E-27 | 50/68 (73%) | Chaetomium globosum CBS 148.51 | hypothetical protein CHGG\_03302 |
| FQ4QJ5301DJC3J | 77 | 3.00E+00 | 11/22 (50%) | Chaetomium globosum CBS 148.51 | hypothetical protein CHGG\_03312 |
| Contig11219 | 74.3 | 3.00E-12 | 45/101 (44%) | Chaetomium globosum CBS 148.51 | hypothetical protein CHGG\_03326 |
| FQ4QJ5301DBQOT | 125 | 8.00E-11 | 27/59 (45%) | Chaetomium globosum CBS 148.51 | hypothetical protein CHGG\_03696 |
| Contig6181 | 40.4 | 4.00E-02 | 15/41 (36%) | Chaetomium globosum CBS 148.51 | hypothetical protein CHGG\_03743 |
| Contig6783 | 105 | 1.00E-21 | 52/73 (71%) | Chaetomium globosum CBS 148.51 | hypothetical protein CHGG\_03930 |
| FQ92HJ001DQUEE | 262 | 1.00E-21 | 51/84 (60%) | Chaetomium globosum CBS 148.51 | hypothetical protein CHGG\_03963 |
| Contig3573 | 39.7 | 2.00E-03 | 21/43 (48%) | Chaetomium globosum CBS 148.51 | hypothetical protein CHGG\_04008 |
| FQ92HJ001A22ZF | 76 | 3.80E+00 | 21/62 (33%) | Chaetomium globosum CBS 148.51 | hypothetical protein CHGG\_04011 |
| FQ4QJ5301ADEBN | 111 | 3.00E-04 | 20/39 (51%) | Chaetomium globosum CBS 148.51 | hypothetical protein CHGG\_04072 |
| FQ4QJ5301EHUM5 | 83 | 5.90E-01 | 20/56 (35%) | Chaetomium globosum CBS 148.51 | hypothetical protein CHGG\_04345 |
| FQ4QJ5301D2F0D | 189 | 3.00E-13 | 35/44 (79%) | Chaetomium globosum CBS 148.51 | hypothetical protein CHGG\_04415 |
| FQ4QJ5301BCAXM | 179 | 4.00E-12 | 36/69 (52%) | Chaetomium globosum CBS 148.51 | hypothetical protein CHGG\_04449 |
| FQ4QJ5301DF7LN | 180 | 4.00E-16 | 35/48 (72%) | Chaetomium globosum CBS 148.51 | hypothetical protein CHGG\_04536 |
| FQ92HJ001ELJPF | 76 | 3.90E+00 | 21/69 (30%) | Chaetomium globosum CBS 148.51 | hypothetical protein CHGG\_04637 |
| FQ4QJ5301DEM3N | 208 | 2.00E-15 | 39/72 (54%) | Chaetomium globosum CBS 148.51 | hypothetical protein CHGG\_04643 |
| Contig12984 | 77 | 4.00E-13 | 40/65 (61%) | Chaetomium globosum CBS 148.51 | hypothetical protein CHGG\_04695 |
| Contig1022 | 58.9 | 3.00E-12 | 35/63 (55%) | Chaetomium globosum CBS 148.51 | hypothetical protein CHGG\_04765 |
| FQ4QJ5301AQJ6D | 214 | 4.00E-16 | 45/74 (60%) | Chaetomium globosum CBS 148.51 | hypothetical protein CHGG\_04782 |
| FQ92HJ001BXRN9 | 80 | 1.30E+00 | 20/60 (33%) | Chaetomium globosum CBS 148.51 | hypothetical protein CHGG\_04818 |
| Contig2996 | 94 | 3.00E-18 | 41/56 (73%) | Chaetomium globosum CBS 148.51 | hypothetical protein CHGG\_04834 |
| FQ92HJ001COTIG | 73 | 8.60E+00 | 17/44 (38%) | Chaetomium globosum CBS 148.51 | hypothetical protein CHGG\_04878 |
| FQ4QJ5301BXSYC | 174 | 2.00E-11 | 38/77 (49%) | Chaetomium globosum CBS 148.51 | hypothetical protein CHGG\_04946 |
| FQ4QJ5301CHIK1 | 306 | 8.00E-27 | 54/76 (71%) | Chaetomium globosum CBS 148.51 | hypothetical protein CHGG\_05057 |
| FQ4QJ5301EZB51 | 77 | 2.90E+00 | 16/46 (34%) | Chaetomium globosum CBS 148.51 | hypothetical protein CHGG\_05057 |
| FQ92HJ001EX9LW | 119 | 4.00E-05 | 25/61 (40%) | Chaetomium globosum CBS 148.51 | hypothetical protein CHGG\_05229 |
| FQ4QJ5301DVUSM | 105 | 1.00E-06 | 20/35 (57%) | Chaetomium globosum CBS 148.51 | hypothetical protein CHGG\_05292 |
| FQ4QJ5301BG1V3 | 111 | 3.00E-04 | 22/35 (62%) | Chaetomium globosum CBS 148.51 | hypothetical protein CHGG\_05313 |
| Contig421 | 34.7 | 2.30E+00 | 36/104 (34%) | Chaetomium globosum CBS 148.51 | hypothetical protein CHGG\_05343 |
| FQ4QJ5301CSBRF | 73 | 8.50E+00 | 14/19 (73%) | Chaetomium globosum CBS 148.51 | hypothetical protein CHGG\_05360 |
| FQ92HJ001EVDR6 | 376 | 6.00E-35 | 72/86 (83%) | Chaetomium globosum CBS 148.51 | hypothetical protein CHGG\_05396 |
| FQ4QJ5301CKZND | 167 | 1.00E-10 | 29/39 (74%) | Chaetomium globosum CBS 148.51 | hypothetical protein CHGG\_05505 |
| FQ4QJ5301COWSJ | 285 | 2.00E-29 | 51/55 (92%) | Chaetomium globosum CBS 148.51 | hypothetical protein CHGG\_05557 |
| FQ4QJ5301EDXIY | 403 | 5.00E-38 | 77/85 (90%) | Chaetomium globosum CBS 148.51 | hypothetical protein CHGG\_05724 |
| Contig11505 | 103 | 3.00E-21 | 47/76 (61%) | Chaetomium globosum CBS 148.51 | hypothetical protein CHGG\_05733 |
| FQ4QJ5301ELUZK | 80 | 1.30E+00 | 21/44 (47%) | Chaetomium globosum CBS 148.51 | hypothetical protein CHGG\_05779 |
| FQ4QJ5301BFZ2Y | 204 | 2.00E-16 | 41/48 (85%) | Chaetomium globosum CBS 148.51 | hypothetical protein CHGG\_05918 |
| FQ4QJ5301D969V | 433 | 2.00E-41 | 79/85 (92%) | Chaetomium globosum CBS 148.51 | hypothetical protein CHGG\_05982 |
| Contig14226 | 82.8 | 7.00E-15 | 40/83 (48%) | Chaetomium globosum CBS 148.51 | hypothetical protein CHGG\_06083 |
| FQ4QJ5301CKGZG | 187 | 5.00E-13 | 36/66 (54%) | Chaetomium globosum CBS 148.51 | hypothetical protein CHGG\_06165 |
| Contig4665 | 157 | 2.00E-37 | 81/135 (60%) | Chaetomium globosum CBS 148.51 | hypothetical protein CHGG\_06173 |
| FQ4QJ5301DQG74 | 85 | 3.20E-01 | 16/20 (80%) | Chaetomium globosum CBS 148.51 | hypothetical protein CHGG\_06298 |
| FQ92HJ001C9O8W | 75 | 4.90E+00 | 16/45 (35%) | Chaetomium globosum CBS 148.51 | hypothetical protein CHGG\_06335 |
| FQ4QJ5301DTATN | 107 | 1.00E-03 | 15/26 (57%) | Chaetomium globosum CBS 148.51 | hypothetical protein CHGG\_06379 |
| FQ92HJ001EER13 | 88 | 1.50E-01 | 21/45 (46%) | Chaetomium globosum CBS 148.51 | hypothetical protein CHGG\_06540 |
| FQ92HJ001C2QQ7 | 197 | 4.00E-14 | 36/59 (61%) | Chaetomium globosum CBS 148.51 | hypothetical protein CHGG\_06639 |
| FQ4QJ5301E060X | 74 | 6.50E+00 | 20/58 (34%) | Chaetomium globosum CBS 148.51 | hypothetical protein CHGG\_06715 |
| FQ4QJ5301C1VYM | 96 | 1.80E-02 | 25/45 (55%) | Chaetomium globosum CBS 148.51 | hypothetical protein CHGG\_06721 |
| Contig4543 | 160 | 4.00E-38 | 67/114 (58%) | Chaetomium globosum CBS 148.51 | hypothetical protein CHGG\_06765 |
| FQ4QJ5301BY59G | 97 | 1.40E-02 | 28/68 (41%) | Chaetomium globosum CBS 148.51 | hypothetical protein CHGG\_06803 |
| FQ4QJ5301C9M7L | 189 | 3.00E-13 | 31/36 (86%) | Chaetomium globosum CBS 148.51 | hypothetical protein CHGG\_06808 |
| FQ4QJ5301DBR4D | 241 | 3.00E-19 | 45/71 (63%) | Chaetomium globosum CBS 148.51 | hypothetical protein CHGG\_06808 |
| FQ92HJ001ENXI7 | 87 | 2.00E-01 | 26/74 (35%) | Chaetomium globosum CBS 148.51 | hypothetical protein CHGG\_06813 |
| FQ4QJ5301BY1UC | 82 | 7.80E-01 | 20/59 (33%) | Chaetomium globosum CBS 148.51 | hypothetical protein CHGG\_06856 |
| Contig7345 | 46.6 | 6.00E-04 | 26/60 (43%) | Chaetomium globosum CBS 148.51 | hypothetical protein CHGG\_06981 |
| Contig11079 | 33.1 | 6.60E+00 | 17/43 (39%) | Chaetomium globosum CBS 148.51 | hypothetical protein CHGG\_07010 |
| Contig1602 | 53.1 | 6.00E-06 | 25/69 (36%) | Chaetomium globosum CBS 148.51 | hypothetical protein CHGG\_07090 |
| FQ4QJ5301B2QKN | 113 | 2.00E-04 | 20/26 (76%) | Chaetomium globosum CBS 148.51 | hypothetical protein CHGG\_07140 |
| Contig12939 | 89.7 | 6.00E-17 | 44/60 (73%) | Chaetomium globosum CBS 148.51 | hypothetical protein CHGG\_07216 |
| Contig2939 | 73.6 | 4.00E-12 | 34/46 (73%) | Chaetomium globosum CBS 148.51 | hypothetical protein CHGG\_07289 |
| Contig4785 | 159 | 6.00E-38 | 75/97 (77%) | Chaetomium globosum CBS 148.51 | hypothetical protein CHGG\_07289 |
| Contig15588 | 40.4 | 4.00E-02 | 24/56 (42%) | Chaetomium globosum CBS 148.51 | hypothetical protein CHGG\_07475 |
| FQ92HJ001DCE4B | 118 | 5.00E-05 | 24/49 (48%) | Chaetomium globosum CBS 148.51 | hypothetical protein CHGG\_07566 |
| Contig8662 | 33.5 | 5.00E+00 | 21/60 (35%) | Chaetomium globosum CBS 148.51 | hypothetical protein CHGG\_07609 |
| FQ92HJ001CFATX | 276 | 2.00E-23 | 47/59 (79%) | Chaetomium globosum CBS 148.51 | hypothetical protein CHGG\_07655 |
| FQ4QJ5301DYSXT | 80 | 1.30E+00 | 18/39 (46%) | Chaetomium globosum CBS 148.51 | hypothetical protein CHGG\_07745 |
| FQ4QJ5301B2W7V | 82 | 7.70E-01 | 19/41 (46%) | Chaetomium globosum CBS 148.51 | hypothetical protein CHGG\_07745 |
| FQ4QJ5301D25MA | 284 | 3.00E-24 | 55/71 (77%) | Chaetomium globosum CBS 148.51 | hypothetical protein CHGG\_07784 |
| Contig3947 | 34.7 | 2.20E+00 | 16/34 (47%) | Chaetomium globosum CBS 148.51 | hypothetical protein CHGG\_07797 |
| FQ4QJ5301B1TUI | 111 | 3.00E-04 | 19/32 (59%) | Chaetomium globosum CBS 148.51 | hypothetical protein CHGG\_07836 |
| FQ92HJ001CC988 | 108 | 7.00E-04 | 21/26 (80%) | Chaetomium globosum CBS 148.51 | hypothetical protein CHGG\_07917 |
| FQ4QJ5301CDE7R | 155 | 3.00E-09 | 42/76 (55%) | Chaetomium globosum CBS 148.51 | hypothetical protein CHGG\_07992 |
| FQ4QJ5301DKAU1 | 86 | 2.70E-01 | 16/20 (80%) | Chaetomium globosum CBS 148.51 | hypothetical protein CHGG\_07997 |
| FQ4QJ5301EW945 | 195 | 6.00E-14 | 38/56 (67%) | Chaetomium globosum CBS 148.51 | hypothetical protein CHGG\_07997 |
| FQ4QJ5301CHL82 | 85 | 3.50E-01 | 16/56 (28%) | Chaetomium globosum CBS 148.51 | hypothetical protein CHGG\_08137 |
| FQ4QJ5301BL66S | 170 | 5.00E-11 | 34/64 (53%) | Chaetomium globosum CBS 148.51 | hypothetical protein CHGG\_08137 |
| Contig2061 | 40 | 5.40E-02 | 23/49 (46%) | Chaetomium globosum CBS 148.51 | hypothetical protein CHGG\_08138 |
| FQ4QJ5301C3JC8 | 296 | 1.00E-25 | 62/85 (72%) | Chaetomium globosum CBS 148.51 | hypothetical protein CHGG\_08209 |
| Contig14019 | 42 | 1.40E-02 | 27/73 (36%) | Chaetomium globosum CBS 148.51 | hypothetical protein CHGG\_08231 |
| Contig9185 | 171 | 2.00E-41 | 75/89 (84%) | Chaetomium globosum CBS 148.51 | hypothetical protein CHGG\_08251 |
| Contig13187 | 36.2 | 7.60E-01 | 21/66 (31%) | Chaetomium globosum CBS 148.51 | hypothetical protein CHGG\_08290 |
| Contig16868 | 52 | 1.00E-05 | 24/35 (68%) | Chaetomium globosum CBS 148.51 | hypothetical protein CHGG\_08293 |
| FQ4QJ5301E5L61 | 79 | 1.70E+00 | 17/45 (37%) | Chaetomium globosum CBS 148.51 | hypothetical protein CHGG\_08296 |
| FQ92HJ001D0HU3 | 78 | 8.00E-04 | 16/30 (53%) | Chaetomium globosum CBS 148.51 | hypothetical protein CHGG\_08296 |
| FQ4QJ5301BOAJ2 | 85 | 3.50E-01 | 17/35 (48%) | Chaetomium globosum CBS 148.51 | hypothetical protein CHGG\_08331 |
| Contig16190 | 107 | 4.00E-22 | 50/82 (60%) | Chaetomium globosum CBS 148.51 | hypothetical protein CHGG\_08337 |
| FQ92HJ001D57VF | 109 | 6.00E-04 | 30/80 (37%) | Chaetomium globosum CBS 148.51 | hypothetical protein CHGG\_08347 |
| Contig3014 | 68.6 | 1.00E-10 | 39/84 (46%) | Chaetomium globosum CBS 148.51 | hypothetical protein CHGG\_08368 |
| FQ4QJ5301CITOA | 177 | 7.00E-12 | 34/55 (61%) | Chaetomium globosum CBS 148.51 | hypothetical protein CHGG\_08393 |
| FQ4QJ5301D8DQ6 | 89 | 1.20E-01 | 17/34 (50%) | Chaetomium globosum CBS 148.51 | hypothetical protein CHGG\_08393 |
| FQ92HJ001CPAPN | 128 | 4.00E-06 | 24/67 (35%) | Chaetomium globosum CBS 148.51 | hypothetical protein CHGG\_08418 |
| FQ4QJ5301AZLZ1 | 115 | 1.00E-04 | 32/68 (47%) | Chaetomium globosum CBS 148.51 | hypothetical protein CHGG\_08449 |
| FQ4QJ5301DDDLY | 81 | 1.00E+00 | 14/36 (38%) | Chaetomium globosum CBS 148.51 | hypothetical protein CHGG\_08513 |
| FQ92HJ001D0OS7 | 191 | 2.00E-13 | 39/72 (54%) | Chaetomium globosum CBS 148.51 | hypothetical protein CHGG\_08718 |
| Contig14029 | 150 | 4.00E-35 | 74/76 (97%) | Chaetomium globosum CBS 148.51 | hypothetical protein CHGG\_08832 |
| FQ4QJ5301EIAHD | 76 | 3.80E+00 | 12/23 (52%) | Chaetomium globosum CBS 148.51 | hypothetical protein CHGG\_08982 |
| Contig8628 | 100 | 3.00E-20 | 57/125 (45%) | Chaetomium globosum CBS 148.51 | hypothetical protein CHGG\_09042 |
| Contig7916 | 34.7 | 2.20E+00 | 22/61 (36%) | Chaetomium globosum CBS 148.51 | hypothetical protein CHGG\_09223 |
| FQ4QJ5301CLF2E | 231 | 4.00E-18 | 48/77 (62%) | Chaetomium globosum CBS 148.51 | hypothetical protein CHGG\_09254 |
| FQ92HJ001BLC1L | 106 | 1.00E-03 | 18/40 (45%) | Chaetomium globosum CBS 148.51 | hypothetical protein CHGG\_09370 |
| FQ92HJ001CX4EI | 103 | 3.00E-04 | 19/44 (43%) | Chaetomium globosum CBS 148.51 | hypothetical protein CHGG\_09370 |
| Contig4462 | 35.4 | 1.30E+00 | 21/50 (42%) | Chaetomium globosum CBS 148.51 | hypothetical protein CHGG\_09474 |
| FQ4QJ5301DKNPW | 122 | 2.00E-05 | 19/36 (52%) | Chaetomium globosum CBS 148.51 | hypothetical protein CHGG\_09560 |
| FQ4QJ5301CYVLS | 152 | 6.00E-09 | 29/32 (90%) | Chaetomium globosum CBS 148.51 | hypothetical protein CHGG\_09682 |
| FQ4QJ5301B256Q | 360 | 4.00E-33 | 65/80 (81%) | Chaetomium globosum CBS 148.51 | hypothetical protein CHGG\_09688 |
| FQ92HJ001CDAQO | 266 | 4.00E-22 | 52/76 (68%) | Chaetomium globosum CBS 148.51 | hypothetical protein CHGG\_09786 |
| Contig11686 | 48.5 | 1.00E-04 | 27/58 (46%) | Chaetomium globosum CBS 148.51 | hypothetical protein CHGG\_09793 |
| Contig9041 | 38.9 | 1.20E-01 | 19/46 (41%) | Chaetomium globosum CBS 148.51 | hypothetical protein CHGG\_09897 |
| Contig105 | 58.5 | 1.00E-07 | 27/61 (44%) | Chaetomium globosum CBS 148.51 | hypothetical protein CHGG\_09951 |
| FQ4QJ5301DZB75 | 402 | 6.00E-38 | 80/82 (97%) | Chaetomium globosum CBS 148.51 | hypothetical protein CHGG\_09993 |
| Contig4239 | 75.1 | 2.00E-12 | 36/71 (50%) | Chaetomium globosum CBS 148.51 | hypothetical protein CHGG\_09996 |
| FQ92HJ001BYZZ8 | 150 | 1.00E-08 | 28/43 (65%) | Chaetomium globosum CBS 148.51 | hypothetical protein CHGG\_09996 |
| FQ4QJ5301BOAR0 | 110 | 4.00E-04 | 19/23 (82%) | Chaetomium globosum CBS 148.51 | hypothetical protein CHGG\_10313 |
| FQ4QJ5301AVHQM | 128 | 4.00E-06 | 25/58 (43%) | Chaetomium globosum CBS 148.51 | hypothetical protein CHGG\_10328 |
| FQ4QJ5301DS1B0 | 418 | 8.00E-40 | 80/87 (91%) | Chaetomium globosum CBS 148.51 | hypothetical protein CHGG\_10374 |
| Contig5269 | 75.1 | 2.00E-12 | 31/64 (48%) | Chaetomium globosum CBS 148.51 | hypothetical protein CHGG\_10731 |
| FQ4QJ5301ARJ6B | 110 | 4.00E-04 | 21/35 (60%) | Chaetomium globosum CBS 148.51 | hypothetical protein CHGG\_10731 |
| FQ4QJ5301BQS63 | 315 | 7.00E-28 | 56/80 (70%) | Chaetomium globosum CBS 148.51 | hypothetical protein CHGG\_10731 |
| FQ92HJ001DRQE8 | 142 | 5.00E-19 | 25/41 (60%) | Chaetomium globosum CBS 148.51 | hypothetical protein CHGG\_10731 |
| FQ92HJ001B03DU | 163 | 3.00E-10 | 30/50 (60%) | Chaetomium globosum CBS 148.51 | hypothetical protein CHGG\_10731 |
| FQ92HJ001EX95M | 229 | 7.00E-18 | 43/79 (54%) | Chaetomium globosum CBS 148.51 | hypothetical protein CHGG\_10731 |
| FQ92HJ001CV0EV | 122 | 2.00E-05 | 23/76 (30%) | Chaetomium globosum CBS 148.51 | hypothetical protein CHGG\_10826 |
| Contig5648 | 44.3 | 2.00E-04 | 24/56 (42%) | Chaetomium globosum CBS 148.51 | hypothetical protein CHGG\_10828 |
| FQ92HJ001DBXHD | 322 | 1.00E-28 | 58/71 (81%) | Chaetomium globosum CBS 148.51 | hypothetical protein CHGG\_10857 |
| FQ92HJ001C2G1M | 74 | 6.50E+00 | 13/38 (34%) | Chaetomium globosum CBS 148.51 | hypothetical protein CHGG\_11067 |
| Contig8664 | 128 | 9.00E-28 | 62/85 (72%) | Chaetomium globosum CBS 148.51 | hypothetical protein CHGG\_11100 |
| Contig14229 | 49.3 | 9.00E-05 | 22/25 (88%) | Chaetomium globosum CBS 148.51 | hypothetical protein CHGG\_11101 |
| Contig15259 | 125 | 2.00E-28 | 62/90 (68%) | Chaetomium globosum CBS 148.51 | hypothetical protein CHGG\_11101 |
| FQ92HJ001ESJY6 | 109 | 6.00E-04 | 21/24 (87%) | Chaetomium globosum CBS 148.51 | hypothetical protein CHGG\_11101 |
| FQ92HJ001B62Y4 | 206 | 1.00E-28 | 39/40 (97%) | Chaetomium globosum CBS 148.51 | hypothetical protein CHGG\_11101 |
| FQ92HJ001DS9UQ | 159 | 9.00E-10 | 29/45 (64%) | Chaetomium globosum CBS 148.51 | hypothetical protein CHGG\_11103 |
| Contig8195 | 58.2 | 7.00E-14 | 28/52 (53%) | Chaetomium globosum CBS 148.51 | inorganic pyrophosphatase |
| Contig609 | 33.5 | 5.00E+00 | 14/30 (46%) | Chaetomium globosum CBS 148.51 | predicted protein |
| Contig1620 | 65.1 | 2.00E-09 | 28/35 (80%) | Chaetomium globosum CBS 148.51 | predicted protein |
| Contig3359 | 36.2 | 7.80E-01 | 21/45 (46%) | Chaetomium globosum CBS 148.51 | predicted protein |
| Contig4611 | 32.7 | 8.50E+00 | 23/62 (37%) | Chaetomium globosum CBS 148.51 | predicted protein |
| Contig11521 | 37 | 4.50E-01 | 24/72 (33%) | Chaetomium globosum CBS 148.51 | predicted protein |
| Contig12051 | 35.8 | 1.00E+00 | 17/21 (80%) | Chaetomium globosum CBS 148.51 | predicted protein |
| FQ4QJ5301A7XZD | 83 | 5.90E-01 | 20/66 (30%) | Chaetomium globosum CBS 148.51 | predicted protein |
| FQ4QJ5301DERYU | 79 | 1.70E+00 | 22/66 (33%) | Chaetomium globosum CBS 148.51 | predicted protein |
| FQ4QJ5301BR50K | 118 | 5.00E-05 | 20/34 (58%) | Chaetomium globosum CBS 148.51 | predicted protein |
| FQ4QJ5301DF5QK | 84 | 4.50E-01 | 17/51 (33%) | Chaetomium globosum CBS 148.51 | predicted protein |
| FQ92HJ001DQ161 | 75 | 5.00E+00 | 13/22 (59%) | Chaetomium globosum CBS 148.51 | predicted protein |
| FQ92HJ001AWTTQ | 100 | 6.00E-03 | 20/23 (86%) | Chaetomium globosum CBS 148.51 | predicted protein |
| FQ4QJ5301EKESG | 236 | 2.00E-27 | 44/54 (81%) | Cladosporium fulvum | AF275347\_1 aldehyde dehydrogenase; ALDH |
| FQ4QJ5301DBWP1 | 314 | 1.00E-27 | 68/94 (72%) | Claviceps purpurea | putative multicopperoxidase |
| FQ4QJ5301BEU7H | 78 | 2.30E+00 | 27/87 (31%) | Cochliobolus heterostrophus | kinesin |
| FQ92HJ001E5K7F | 77 | 2.90E+00 | 13/27 (48%) | Cochliobolus heterostrophus | polyketide synthase |
| Contig1827 | 33.1 | 6.60E+00 | 14/43 (32%) | Cochliobolus heterostrophus | putative histidine kinase HHK18p |
| Contig5847 | 96.7 | 5.00E-19 | 52/82 (63%) | Colletotrichum | calmodulin-dependent protein kinase |
| Contig4534 | 92.8 | 7.00E-18 | 41/95 (43%) | Colletotrichum cereale | hypothetical protein |
| Contig10042 | 32.3 | 4.90E-01 | 9/28 (32%) | Colletotrichum cereale | putative integrase |
| FQ4QJ5301BDNBC | 137 | 1.00E-12 | 28/65 (43%) | Colletotrichum cereale | putative integrase |
| Contig13702 | 51.2 | 2.00E-05 | 27/53 (50%) | Coprinopsis cinerea okayama7#130 | conserved hypothetical protein |
| FQ92HJ001DDO9W | 75 | 5.00E+00 | 17/32 (53%) | Coprinopsis cinerea okayama7#130 | hypothetical protein CC1G\_00087 |
| FQ92HJ001AGQQK | 114 | 1.00E-04 | 23/32 (71%) | Coprinopsis cinerea okayama7#130 | hypothetical protein CC1G\_00135 |
| FQ92HJ001DW3H6 | 311 | 2.00E-27 | 59/75 (78%) | Coprinopsis cinerea okayama7#130 | hypothetical protein CC1G\_00238 |
| FQ4QJ5301ANP9C | 332 | 8.00E-30 | 61/76 (80%) | Coprinopsis cinerea okayama7#130 | hypothetical protein CC1G\_00368 |
| Contig13769 | 92.8 | 7.00E-18 | 45/78 (57%) | Coprinopsis cinerea okayama7#130 | hypothetical protein CC1G\_00377 |
| FQ92HJ001CPNY6 | 306 | 8.00E-27 | 63/66 (95%) | Coprinopsis cinerea okayama7#130 | hypothetical protein CC1G\_01907 |
| FQ92HJ001A0BA0 | 138 | 2.00E-07 | 26/50 (52%) | Coprinopsis cinerea okayama7#130 | hypothetical protein CC1G\_02011 |
| FQ92HJ001BHXL6 | 73 | 8.40E+00 | 19/66 (28%) | Coprinopsis cinerea okayama7#130 | hypothetical protein CC1G\_02057 |
| FQ92HJ001A4Q2A | 77 | 2.90E+00 | 20/57 (35%) | Coprinopsis cinerea okayama7#130 | hypothetical protein CC1G\_02192 |
| FQ92HJ001D5UGL | 77 | 2.90E+00 | 18/43 (41%) | Coprinopsis cinerea okayama7#130 | hypothetical protein CC1G\_02717 |
| Contig11992 | 33.1 | 6.50E+00 | 16/37 (43%) | Coprinopsis cinerea okayama7#130 | hypothetical protein CC1G\_03539 |
| FQ92HJ001B7IEL | 277 | 2.00E-23 | 50/80 (62%) | Coprinopsis cinerea okayama7#130 | hypothetical protein CC1G\_03630 |
| Contig8813 | 55.1 | 2.00E-06 | 32/85 (37%) | Coprinopsis cinerea okayama7#130 | hypothetical protein CC1G\_03706 |
| Contig10006 | 44.3 | 3.00E-03 | 16/39 (41%) | Coprinopsis cinerea okayama7#130 | hypothetical protein CC1G\_04246 |
| Contig13719 | 35 | 1.70E+00 | 19/62 (30%) | Coprinopsis cinerea okayama7#130 | hypothetical protein CC1G\_04416 |
| Contig12370 | 35.8 | 1.90E+00 | 15/55 (27%) | Coprinopsis cinerea okayama7#130 | hypothetical protein CC1G\_04487 |
| Contig7487 | 32.7 | 8.60E+00 | 18/47 (38%) | Coprinopsis cinerea okayama7#130 | hypothetical protein CC1G\_04554 |
| Contig1091 | 74.7 | 2.00E-12 | 31/50 (62%) | Coprinopsis cinerea okayama7#130 | hypothetical protein CC1G\_04782 |
| FQ92HJ001EZDHG | 125 | 8.00E-06 | 27/73 (36%) | Coprinopsis cinerea okayama7#130 | hypothetical protein CC1G\_04782 |
| FQ92HJ001DFNSD | 301 | 3.00E-26 | 61/77 (79%) | Coprinopsis cinerea okayama7#130 | hypothetical protein CC1G\_05118 |
| FQ4QJ5301EZL5W | 79 | 1.70E+00 | 18/41 (43%) | Coprinopsis cinerea okayama7#130 | hypothetical protein CC1G\_05122 |
| FQ92HJ001DCNU4 | 315 | 7.00E-28 | 58/75 (77%) | Coprinopsis cinerea okayama7#130 | hypothetical protein CC1G\_05413 |
| Contig1643 | 32.7 | 8.60E+00 | 14/31 (45%) | Coprinopsis cinerea okayama7#130 | hypothetical protein CC1G\_05935 |
| Contig2363 | 35.4 | 1.30E+00 | 18/55 (32%) | Coprinopsis cinerea okayama7#130 | hypothetical protein CC1G\_06175 |
| FQ92HJ001DKF4G | 174 | 2.00E-11 | 31/52 (59%) | Coprinopsis cinerea okayama7#130 | hypothetical protein CC1G\_06520 |
| Contig2124 | 32.7 | 8.40E+00 | 14/35 (40%) | Coprinopsis cinerea okayama7#130 | hypothetical protein CC1G\_06785 |
| FQ92HJ001ECP6S | 76 | 3.90E+00 | 21/56 (37%) | Coprinopsis cinerea okayama7#130 | hypothetical protein CC1G\_07262 |
| FQ92HJ001B08S8 | 252 | 1.00E-20 | 47/71 (66%) | Coprinopsis cinerea okayama7#130 | hypothetical protein CC1G\_07500 |
| FQ4QJ5301B9V8D | 80 | 1.30E+00 | 21/51 (41%) | Coprinopsis cinerea okayama7#130 | hypothetical protein CC1G\_07901 |
| FQ92HJ001CEE34 | 75 | 4.90E+00 | 19/45 (42%) | Coprinopsis cinerea okayama7#130 | hypothetical protein CC1G\_08142 |
| Contig6905 | 41.2 | 3.60E-02 | 20/36 (55%) | Coprinopsis cinerea okayama7#130 | hypothetical protein CC1G\_08293 |
| FQ4QJ5301CWCNB | 80 | 1.30E+00 | 14/43 (32%) | Coprinopsis cinerea okayama7#130 | hypothetical protein CC1G\_09360 |
| FQ4QJ5301EJ7WB | 73 | 8.50E+00 | 17/49 (34%) | Coprinopsis cinerea okayama7#130 | hypothetical protein CC1G\_11055 |
| FQ92HJ001CDQH5 | 76 | 3.80E+00 | 19/35 (54%) | Coprinopsis cinerea okayama7#130 | hypothetical protein CC1G\_11606 |
| FQ92HJ001DQ6CP | 77 | 2.90E+00 | 15/32 (46%) | Coprinopsis cinerea okayama7#130 | hypothetical protein CC1G\_12019 |
| FQ4QJ5301EBMC2 | 75 | 5.00E+00 | 15/33 (45%) | Coprinopsis cinerea okayama7#130 | hypothetical protein CC1G\_12165 |
| FQ4QJ5301CD3FI | 109 | 6.00E-04 | 24/60 (40%) | Coprinopsis cinerea okayama7#130 | hypothetical protein CC1G\_12511 |
| Contig13098 | 52 | 1.00E-05 | 25/57 (43%) | Coprinopsis cinerea okayama7#130 | hypothetical protein CC1G\_12653 |
| Contig14956 | 70.5 | 4.00E-11 | 30/58 (51%) | Coprinopsis cinerea okayama7#130 | hypothetical protein CC1G\_12655 |
| Contig1249 | 34.3 | 3.00E+00 | 15/29 (51%) | Coprinopsis cinerea okayama7#130 | predicted protein |
| Contig4538 | 37.7 | 7.00E-01 | 25/70 (35%) | Coprinopsis cinerea okayama7#130 | predicted protein |
| Contig4615 | 33.9 | 3.80E+00 | 15/24 (62%) | Coprinopsis cinerea okayama7#130 | predicted protein |
| Contig5155 | 33.9 | 3.80E+00 | 26/76 (34%) | Coprinopsis cinerea okayama7#130 | predicted protein |
| Contig12607 | 33.9 | 3.80E+00 | 27/98 (27%) | Coprinopsis cinerea okayama7#130 | predicted protein |
| Contig16398 | 73.9 | 3.00E-12 | 37/63 (58%) | Coprinopsis cinerea okayama7#130 | predicted protein |
| FQ4QJ5301BIOYS | 75 | 5.10E+00 | 15/29 (51%) | Coprinopsis cinerea okayama7#130 | predicted protein |
| FQ4QJ5301BHH0P | 84 | 4.50E-01 | 26/61 (42%) | Coprinopsis cinerea okayama7#130 | predicted protein |
| FQ4QJ5301BA31S | 77 | 3.00E+00 | 25/73 (34%) | Coprinopsis cinerea okayama7#130 | predicted protein |
| FQ4QJ5301DO9E2 | 76 | 3.80E+00 | 20/55 (36%) | Coprinopsis cinerea okayama7#130 | predicted protein |
| FQ92HJ001CXJKO | 77 | 3.00E+00 | 15/30 (50%) | Coprinopsis cinerea okayama7#130 | predicted protein |
| FQ92HJ001DEFO1 | 211 | 9.00E-16 | 39/57 (68%) | Coprinopsis cinerea okayama7#130 | predicted protein |
| FQ92HJ001A6AZE | 107 | 1.00E-03 | 27/54 (50%) | Coprinopsis cinerea okayama7#130 | predicted protein |
| FQ92HJ001DV33D | 74 | 6.60E+00 | 13/35 (37%) | Coprinopsis cinerea okayama7#130 | predicted protein |
| FQ92HJ001CUAPA | 74 | 6.60E+00 | 16/42 (38%) | Coprinopsis cinerea okayama7#130 | predicted protein |
| Contig14580 | 37.4 | 3.50E-01 | 31/107 (28%) | Debaryomyces hansenii | endonuclease |
| FQ4QJ5301CHV0U | 81 | 1.00E+00 | 21/69 (30%) | Debaryomyces hansenii CBS767 | hypothetical protein DEHA0A01166g |
| FQ92HJ001AWCH0 | 75 | 5.00E+00 | 18/67 (26%) | Debaryomyces hansenii CBS767 | hypothetical protein DEHA0A02651g |
| Contig12154 | 33.5 | 4.90E+00 | 29/82 (35%) | Debaryomyces hansenii CBS767 | hypothetical protein DEHA0A05192g |
| Contig9519 | 36.6 | 1.60E+00 | 23/66 (34%) | Debaryomyces hansenii CBS767 | hypothetical protein DEHA0A06996g |
| FQ4QJ5301CHPBU | 73 | 8.50E+00 | 21/68 (30%) | Debaryomyces hansenii CBS767 | hypothetical protein DEHA0B09856g |
| FQ4QJ5301AIEA5 | 74 | 6.50E+00 | 17/36 (47%) | Debaryomyces hansenii CBS767 | hypothetical protein DEHA0B15070g |
| Contig13853 | 53.1 | 6.00E-06 | 21/26 (80%) | Debaryomyces hansenii CBS767 | hypothetical protein DEHA0C01441g |
| FQ4QJ5301DM8MT | 81 | 1.00E+00 | 14/44 (31%) | Debaryomyces hansenii CBS767 | hypothetical protein DEHA0C15224g |
| Contig13538 | 33.9 | 3.80E+00 | 22/73 (30%) | Debaryomyces hansenii CBS767 | hypothetical protein DEHA0C18579g |
| Contig5025 | 32.7 | 8.60E+00 | 13/34 (38%) | Debaryomyces hansenii CBS767 | hypothetical protein DEHA0D05335g |
| Contig11344 | 34.7 | 2.30E+00 | 15/49 (30%) | Debaryomyces hansenii CBS767 | hypothetical protein DEHA0D15774g |
| FQ92HJ001BUNUR | 73 | 8.60E+00 | 22/57 (38%) | Debaryomyces hansenii CBS767 | hypothetical protein DEHA0D18117g |
| Contig6927 | 35.4 | 1.30E+00 | 22/70 (31%) | Debaryomyces hansenii CBS767 | hypothetical protein DEHA0E05885g |
| Contig10616 | 33.1 | 6.50E+00 | 15/35 (42%) | Debaryomyces hansenii CBS767 | hypothetical protein DEHA0E08129g |
| FQ92HJ001AT5MI | 80 | 1.30E+00 | 13/45 (28%) | Debaryomyces hansenii CBS767 | hypothetical protein DEHA0E09559g |
| FQ92HJ001ER6ZX | 76 | 3.80E+00 | 25/79 (31%) | Debaryomyces hansenii CBS767 | hypothetical protein DEHA0E13970g |
| Contig13265 | 36.2 | 7.70E-01 | 17/49 (34%) | Debaryomyces hansenii CBS767 | hypothetical protein DEHA0E20559g |
| Contig9905 | 34.7 | 2.60E+00 | 17/40 (42%) | Debaryomyces hansenii CBS767 | hypothetical protein DEHA0F05390g |
| Contig3576 | 37.4 | 3.50E-01 | 19/43 (44%) | Debaryomyces hansenii CBS767 | hypothetical protein DEHA0F07843g |
| FQ92HJ001CV96G | 77 | 3.00E+00 | 16/45 (35%) | Debaryomyces hansenii CBS767 | hypothetical protein DEHA0F08987g |
| FQ4QJ5301CLHTL | 78 | 2.30E+00 | 26/71 (36%) | Debaryomyces hansenii CBS767 | hypothetical protein DEHA0F21142g |
| FQ92HJ001BBOQB | 73 | 8.50E+00 | 14/34 (41%) | Debaryomyces hansenii CBS767 | hypothetical protein DEHA0F25300g |
| FQ4QJ5301AUN7D | 79 | 1.70E+00 | 14/56 (25%) | Debaryomyces hansenii CBS767 | hypothetical protein DEHA0F25652g |
| Contig5894 | 33.9 | 3.80E+00 | 22/74 (29%) | Debaryomyces hansenii CBS767 | hypothetical protein DEHA0G13024g |
| FQ92HJ001ENWJV | 79 | 1.70E+00 | 17/45 (37%) | Debaryomyces hansenii CBS767 | hypothetical protein DEHA0G13959g |
| FQ92HJ001EHSN8 | 246 | 7.00E-20 | 44/79 (55%) | Emericella nidulans | AF123461\_1 adenylosuccinate synthetase |
| FQ4QJ5301DSF8U | 368 | 5.00E-34 | 70/79 (88%) | Epichloe festucae | cobalamin-independent methionine synthase |
| FQ4QJ5301DX0E3 | 273 | 5.00E-23 | 53/67 (79%) | Epichloe festucae | cobalamin-independent methionine synthase |
| FQ4QJ5301DS3S0 | 219 | 1.00E-16 | 42/65 (64%) | Epichloe festucae | Gim complex component GIM3-like protein |
| FQ4QJ5301BX9K2 | 358 | 8.00E-33 | 63/69 (91%) | Epichloe festucae | hypothetical protein |
| FQ4QJ5301D63R6 | 77 | 3.20E-01 | 14/20 (70%) | Epichloe festucae | hypothetical protein |
| FQ92HJ001BZMKE | 119 | 4.00E-08 | 22/27 (81%) | Epichloe festucae | hypothetical protein 17A8-02 |
| Contig14404 | 97.4 | 3.00E-19 | 42/52 (80%) | Epichloe festucae | ribosomal protein S15 precursor-like protein |
| FQ4QJ5301BS4FC | 96 | 4.00E-05 | 17/29 (58%) | Epichloe festucae | subtilisin-like protease |
| FQ4QJ5301ETSK2 | 397 | 2.00E-37 | 80/80 (100%) | Epichloe festucae | TATA binding protein |
| FQ92HJ001CKT7S | 420 | 5.00E-40 | 80/80 (100%) | Fusarium culmorum | beta-tubulin |
| FQ92HJ001BLERZ | 82 | 7.70E-01 | 15/18 (83%) | Fusarium oxysporum | NADH dehydrogenase subunit 4L |
| FQ92HJ001CHOBA | 198 | 3.00E-14 | 49/103 (47%) | Fusarium oxysporum | ribosomal protein S3 |
| FQ4QJ5301EQIU4 | 72 | 2.30E-02 | 12/29 (41%) | Fusarium oxysporum | transposase-like protein |
| FQ92HJ001CCJI6 | 158 | 1.00E-09 | 28/72 (38%) | Fusarium oxysporum | transposase-like protein |
| FQ92HJ001C0QJK | 117 | 4.00E-05 | 21/49 (42%) | Fusarium oxysporum | transposase-like protein |
| Contig4570 | 192 | 2.00E-47 | 90/129 (69%) | Fusarium oxysporum f. sp. lycopersici | hypothetical protein |
| FQ4QJ5301CTMA6 | 146 | 7.00E-09 | 31/46 (67%) | Fusarium oxysporum f. sp. lycopersici | hypothetical protein |
| FQ92HJ001BI80J | 109 | 6.00E-04 | 22/35 (62%) | Fusarium oxysporum f. sp. lycopersici | hypothetical protein |
| Contig7018 | 55.8 | 1.00E-11 | 20/64 (31%) | Fusarium oxysporum f. sp. lycopersici | transposase |
| FQ92HJ001B24HL | 126 | 6.00E-06 | 26/69 (37%) | Fusarium oxysporum f. sp. lycopersici | transposase |
| FQ4QJ5301B6WOT | 135 | 6.00E-07 | 24/32 (75%) | Fusarium oxysporum f. sp. lycopersici | white collar 1 |
| Contig1677 | 62 | 4.00E-16 | 28/57 (49%) | Fusarium oxysporum f. sp. melonis | transposase |
| FQ92HJ001DSWXY | 77 | 2.90E+00 | 14/41 (34%) | Fusarium oxysporum f. sp. melonis | transposase |
| Contig14299 | 33.5 | 6.50E+00 | 17/43 (39%) | Fusarium proliferatum | RNA polymerase |
| FQ4QJ5301CZVME | 401 | 8.00E-38 | 77/79 (97%) | Fusarium sp. NRRL 28014 | RNA polymerase II |
| Contig3654 | 108 | 1.00E-22 | 54/60 (90%) | Gibberella | PSA2\_NEUCR Probable proteasome subunit alpha type 2 |
| FQ4QJ5301BVCYS | 250 | 3.00E-20 | 50/80 (62%) | Gibberella | putative two-component response regulator SKN7p |
| FQ4QJ5301AWHOA | 372 | 5.00E-35 | 68/77 (88%) | Gibberella fujikuroi | glutamate synthase |
| Contig11327 | 66.2 | 7.00E-10 | 33/48 (68%) | Gibberella fujikuroi | putative 40S ribosomal protein |
| FQ4QJ5301AXXOY | 85 | 4.00E-04 | 19/27 (70%) | Gibberella fujikuroi | white collar 1-like protein |
| FQ4QJ5301C59GG | 248 | 4.00E-20 | 45/74 (60%) | Gibberella moniliformis | kinesin |
| FQ4QJ5301DE8E0 | 269 | 2.00E-22 | 49/62 (79%) | Gibberella moniliformis | Npt1p |
| Contig16374 | 32.7 | 8.50E+00 | 12/37 (32%) | Gibberella moniliformis | polyketide synthase |
| FQ4QJ5301B8AGG | 410 | 7.00E-39 | 79/89 (88%) | Gibberella pulicaris | ATP-citrat-lyase |
| FQ4QJ5301CVOU7 | 426 | 1.00E-40 | 80/81 (98%) | Gibberella zeae | alpha tubulin |
| Contig12424 | 53.5 | 5.00E-06 | 26/29 (89%) | Gibberella zeae | cytochrome c oxidase subunit 1 |
| FQ92HJ001D09EO | 432 | 2.00E-41 | 83/85 (97%) | Gibberella zeae | cytochrome c oxidase subunit 1 |
| Contig6866 | 99.8 | 6.00E-20 | 54/67 (80%) | Gibberella zeae | PMA1\_NEUCR Plasma membrane ATPase (Proton pump) |
| FQ4QJ5301BFVSZ | 398 | 2.00E-37 | 73/82 (89%) | Gibberella zeae | PMA1\_NEUCR Plasma membrane ATPase (Proton pump) |
| FQ4QJ5301CG392 | 146 | 9.00E-14 | 26/31 (83%) | Gibberella zeae | PWP2\_NEUCR Periodic tryptophan protein 2 homolog |
| FQ4QJ5301DXMUR | 144 | 5.00E-08 | 29/37 (78%) | Gibberella zeae | RING-12 protein |
| FQ92HJ001CVV4C | 282 | 5.00E-24 | 56/62 (90%) | Gibberella zeae | RING-5 |
| Contig1067 | 41.6 | 1.90E-02 | 24/66 (36%) | Gibberella zeae | RING-6 |
| Contig4639 | 108 | 1.00E-22 | 48/60 (80%) | Gibberella zeae PH-1 | hypothetical protein FG00168.1 |
| FQ4QJ5301BS6JY | 257 | 4.00E-21 | 49/80 (61%) | Gibberella zeae PH-1 | hypothetical protein FG00185.1 |
| FQ4QJ5301C10H0 | 129 | 3.00E-06 | 34/77 (44%) | Gibberella zeae PH-1 | hypothetical protein FG00186.1 |
| FQ92HJ001EWSCT | 104 | 2.00E-03 | 34/99 (34%) | Gibberella zeae PH-1 | hypothetical protein FG00187.1 |
| FQ4QJ5301BZLDA | 225 | 2.00E-17 | 44/78 (56%) | Gibberella zeae PH-1 | hypothetical protein FG00189.1 |
| Contig14431 | 69.3 | 8.00E-11 | 31/60 (51%) | Gibberella zeae PH-1 | hypothetical protein FG00190.1 |
| FQ4QJ5301BXRZD | 306 | 8.00E-27 | 61/78 (78%) | Gibberella zeae PH-1 | hypothetical protein FG00243.1 |
| FQ4QJ5301B4EHR | 99 | 8.00E-03 | 19/22 (86%) | Gibberella zeae PH-1 | hypothetical protein FG00261.1 |
| Contig15621 | 49.3 | 5.00E-07 | 20/35 (57%) | Gibberella zeae PH-1 | hypothetical protein FG00281.1 |
| FQ4QJ5301EL5D7 | 227 | 1.00E-17 | 49/74 (66%) | Gibberella zeae PH-1 | hypothetical protein FG00281.1 |
| FQ4QJ5301E3T1S | 252 | 2.00E-20 | 49/60 (81%) | Gibberella zeae PH-1 | hypothetical protein FG00281.1 |
| FQ4QJ5301D8H1W | 341 | 7.00E-31 | 66/75 (88%) | Gibberella zeae PH-1 | hypothetical protein FG00291.1 |
| FQ4QJ5301COC71 | 92 | 5.30E-02 | 14/21 (66%) | Gibberella zeae PH-1 | hypothetical protein FG00294.1 |
| Contig1694 | 57 | 4.00E-07 | 36/91 (39%) | Gibberella zeae PH-1 | hypothetical protein FG00297.1 |
| Contig12437 | 92.8 | 7.00E-18 | 45/55 (81%) | Gibberella zeae PH-1 | hypothetical protein FG00300.1 |
| Contig14944 | 72.8 | 7.00E-12 | 36/45 (80%) | Gibberella zeae PH-1 | hypothetical protein FG00303.1 |
| Contig15365 | 122 | 8.00E-27 | 57/66 (86%) | Gibberella zeae PH-1 | hypothetical protein FG00303.1 |
| FQ4QJ5301B7EHU | 159 | 9.00E-10 | 31/43 (72%) | Gibberella zeae PH-1 | hypothetical protein FG00310.1 |
| FQ4QJ5301CQH0T | 100 | 6.00E-03 | 19/22 (86%) | Gibberella zeae PH-1 | hypothetical protein FG00320.1 |
| FQ4QJ5301EGPK1 | 176 | 1.00E-11 | 34/70 (48%) | Gibberella zeae PH-1 | hypothetical protein FG00321.1 |
| FQ4QJ5301CWCIK | 378 | 4.00E-35 | 70/85 (82%) | Gibberella zeae PH-1 | hypothetical protein FG00328.1 |
| FQ4QJ5301E3RM9 | 161 | 5.00E-10 | 29/41 (70%) | Gibberella zeae PH-1 | hypothetical protein FG00332.1 |
| Contig15266 | 63.2 | 6.00E-09 | 43/103 (41%) | Gibberella zeae PH-1 | hypothetical protein FG00334.1 |
| FQ4QJ5301C9HV4 | 197 | 4.00E-14 | 35/75 (46%) | Gibberella zeae PH-1 | hypothetical protein FG00341.1 |
| Contig2595 | 112 | 9.00E-24 | 49/75 (65%) | Gibberella zeae PH-1 | hypothetical protein FG00348.1 |
| Contig5199 | 96.7 | 5.00E-19 | 47/81 (58%) | Gibberella zeae PH-1 | hypothetical protein FG00348.1 |
| Contig5831 | 82.8 | 7.00E-15 | 41/48 (85%) | Gibberella zeae PH-1 | hypothetical protein FG00360.1 |
| FQ4QJ5301DSM3S | 288 | 1.00E-24 | 61/83 (73%) | Gibberella zeae PH-1 | hypothetical protein FG00362.1 |
| FQ4QJ5301BR2NF | 283 | 4.00E-24 | 57/79 (72%) | Gibberella zeae PH-1 | hypothetical protein FG00364.1 |
| FQ4QJ5301A8RVW | 333 | 6.00E-30 | 64/75 (85%) | Gibberella zeae PH-1 | hypothetical protein FG00364.1 |
| FQ4QJ5301AL0GI | 207 | 6.00E-22 | 34/42 (80%) | Gibberella zeae PH-1 | hypothetical protein FG00364.1 |
| FQ4QJ5301EWMXI | 189 | 3.00E-13 | 36/53 (67%) | Gibberella zeae PH-1 | hypothetical protein FG00366.1 |
| FQ4QJ5301A11K2 | 391 | 1.00E-36 | 74/76 (97%) | Gibberella zeae PH-1 | hypothetical protein FG00367.1 |
| FQ4QJ5301A3ABW | 132 | 1.00E-06 | 24/26 (92%) | Gibberella zeae PH-1 | hypothetical protein FG00369.1 |
| FQ4QJ5301ES5I9 | 133 | 9.00E-07 | 24/49 (48%) | Gibberella zeae PH-1 | hypothetical protein FG00372.1 |
| Contig94 | 74.3 | 3.00E-12 | 41/61 (67%) | Gibberella zeae PH-1 | hypothetical protein FG00373.1 |
| FQ92HJ001CM1ND | 150 | 1.00E-08 | 32/39 (82%) | Gibberella zeae PH-1 | hypothetical protein FG00373.1 |
| FQ4QJ5301CKEB9 | 302 | 2.00E-26 | 55/72 (76%) | Gibberella zeae PH-1 | hypothetical protein FG00375.1 |
| Contig5217 | 57 | 4.00E-07 | 23/37 (62%) | Gibberella zeae PH-1 | hypothetical protein FG00393.1 |
| Contig15638 | 35 | 1.70E+00 | 17/33 (51%) | Gibberella zeae PH-1 | hypothetical protein FG00393.1 |
| Contig8465 | 75.5 | 2.00E-12 | 37/117 (31%) | Gibberella zeae PH-1 | hypothetical protein FG00415.1 |
| FQ4QJ5301BQAIF | 318 | 3.00E-28 | 64/80 (80%) | Gibberella zeae PH-1 | hypothetical protein FG00417.1 |
| FQ92HJ001D4B97 | 255 | 7.00E-21 | 50/62 (80%) | Gibberella zeae PH-1 | hypothetical protein FG00420.1 |
| Contig6817 | 49.3 | 9.00E-05 | 29/66 (43%) | Gibberella zeae PH-1 | hypothetical protein FG00462.1 |
| Contig16568 | 132 | 6.00E-30 | 63/75 (84%) | Gibberella zeae PH-1 | hypothetical protein FG00471.1 |
| Contig4904 | 138 | 1.00E-31 | 69/75 (92%) | Gibberella zeae PH-1 | hypothetical protein FG00478.1 |
| Contig2229 | 38.9 | 1.20E-01 | 20/25 (80%) | Gibberella zeae PH-1 | hypothetical protein FG00491.1 |
| FQ92HJ001CQTW4 | 290 | 6.00E-25 | 50/59 (84%) | Gibberella zeae PH-1 | hypothetical protein FG00493.1 |
| Contig5881 | 177 | 2.00E-43 | 83/110 (75%) | Gibberella zeae PH-1 | hypothetical protein FG00499.1 |
| Contig2830 | 59.3 | 9.00E-08 | 27/48 (56%) | Gibberella zeae PH-1 | hypothetical protein FG00505.1 |
| Contig2335 | 63.5 | 4.00E-09 | 31/46 (67%) | Gibberella zeae PH-1 | hypothetical protein FG00506.1 |
| FQ4QJ5301CK4C1 | 386 | 4.00E-36 | 74/75 (98%) | Gibberella zeae PH-1 | hypothetical protein FG00524.1 |
| Contig11728 | 140 | 4.00E-32 | 68/119 (57%) | Gibberella zeae PH-1 | hypothetical protein FG00525.1 |
| FQ92HJ001BNRM0 | 335 | 4.00E-30 | 58/77 (75%) | Gibberella zeae PH-1 | hypothetical protein FG00530.1 |
| Contig13170 | 43.1 | 6.00E-03 | 18/35 (51%) | Gibberella zeae PH-1 | hypothetical protein FG00556.1 |
| Contig14807 | 66.2 | 7.00E-10 | 28/39 (71%) | Gibberella zeae PH-1 | hypothetical protein FG00556.1 |
| FQ4QJ5301AMHUL | 350 | 7.00E-32 | 68/74 (91%) | Gibberella zeae PH-1 | hypothetical protein FG00558.1 |
| FQ4QJ5301EEGDI | 118 | 5.00E-05 | 26/47 (55%) | Gibberella zeae PH-1 | hypothetical protein FG00558.1 |
| FQ4QJ5301B3ZPE | 231 | 4.00E-18 | 42/48 (87%) | Gibberella zeae PH-1 | hypothetical protein FG00561.1 |
| FQ4QJ5301CNHPF | 172 | 3.00E-11 | 34/43 (79%) | Gibberella zeae PH-1 | hypothetical protein FG00562.1 |
| FQ92HJ001DM8IM | 168 | 8.00E-11 | 31/52 (59%) | Gibberella zeae PH-1 | hypothetical protein FG00563.1 |
| FQ92HJ001AQ2RK | 338 | 2.00E-30 | 61/75 (81%) | Gibberella zeae PH-1 | hypothetical protein FG00564.1 |
| Contig7141 | 92 | 1.00E-17 | 41/78 (52%) | Gibberella zeae PH-1 | hypothetical protein FG00566.1 |
| FQ4QJ5301CN2ZM | 186 | 7.00E-13 | 37/40 (92%) | Gibberella zeae PH-1 | hypothetical protein FG00595.1 |
| FQ4QJ5301CG3AG | 333 | 6.00E-30 | 68/85 (80%) | Gibberella zeae PH-1 | hypothetical protein FG00597.1 |
| FQ4QJ5301BNFGQ | 189 | 4.00E-14 | 35/37 (94%) | Gibberella zeae PH-1 | hypothetical protein FG00600.1 |
| FQ92HJ001EPAQA | 175 | 1.00E-11 | 30/45 (66%) | Gibberella zeae PH-1 | hypothetical protein FG00609.1 |
| FQ4QJ5301DP77D | 206 | 3.00E-15 | 34/45 (75%) | Gibberella zeae PH-1 | hypothetical protein FG00611.1 |
| FQ4QJ5301AOWEK | 129 | 3.00E-06 | 30/71 (42%) | Gibberella zeae PH-1 | hypothetical protein FG00613.1 |
| Contig11946 | 115 | 8.00E-25 | 55/66 (83%) | Gibberella zeae PH-1 | hypothetical protein FG00626.1 |
| FQ4QJ5301APWW4 | 319 | 3.00E-28 | 68/103 (66%) | Gibberella zeae PH-1 | hypothetical protein FG00627.1 |
| FQ4QJ5301AUCK5 | 244 | 1.00E-19 | 44/76 (57%) | Gibberella zeae PH-1 | hypothetical protein FG00645.1 |
| Contig4697 | 162 | 5.00E-39 | 77/82 (93%) | Gibberella zeae PH-1 | hypothetical protein FG00646.1 |
| FQ4QJ5301C30QK | 149 | 6.00E-11 | 29/39 (74%) | Gibberella zeae PH-1 | hypothetical protein FG00647.1 |
| Contig12389 | 129 | 5.00E-29 | 65/87 (74%) | Gibberella zeae PH-1 | hypothetical protein FG00649.1 |
| FQ92HJ001A55VY | 127 | 5.00E-06 | 23/29 (79%) | Gibberella zeae PH-1 | hypothetical protein FG00656.1 |
| FQ4QJ5301CZBC3 | 163 | 3.00E-10 | 28/28 (100%) | Gibberella zeae PH-1 | hypothetical protein FG00658.1 |
| Contig4336 | 101 | 2.00E-20 | 52/88 (59%) | Gibberella zeae PH-1 | hypothetical protein FG00670.1 |
| FQ4QJ5301CXLBR | 197 | 4.00E-14 | 38/62 (61%) | Gibberella zeae PH-1 | hypothetical protein FG00679.1 |
| FQ4QJ5301ERA51 | 223 | 3.00E-17 | 42/77 (54%) | Gibberella zeae PH-1 | hypothetical protein FG00686.1 |
| FQ4QJ5301CT0RC | 188 | 1.00E-17 | 35/48 (72%) | Gibberella zeae PH-1 | hypothetical protein FG00688.1 |
| FQ92HJ001C9QN5 | 182 | 2.00E-12 | 42/72 (58%) | Gibberella zeae PH-1 | hypothetical protein FG00690.1 |
| FQ4QJ5301EY0UQ | 179 | 4.00E-12 | 36/72 (50%) | Gibberella zeae PH-1 | hypothetical protein FG00693.1 |
| FQ92HJ001DU5D5 | 132 | 1.00E-06 | 31/80 (38%) | Gibberella zeae PH-1 | hypothetical protein FG00699.1 |
| FQ4QJ5301ELJ3H | 180 | 3.00E-12 | 40/67 (59%) | Gibberella zeae PH-1 | hypothetical protein FG00709.1 |
| FQ4QJ5301C00R8 | 263 | 8.00E-22 | 50/70 (71%) | Gibberella zeae PH-1 | hypothetical protein FG00723.1 |
| Contig8405 | 34.7 | 2.20E+00 | 18/40 (45%) | Gibberella zeae PH-1 | hypothetical protein FG00729.1 |
| FQ4QJ5301BD9JV | 240 | 4.00E-19 | 39/60 (65%) | Gibberella zeae PH-1 | hypothetical protein FG00730.1 |
| Contig6949 | 89.4 | 8.00E-17 | 37/53 (69%) | Gibberella zeae PH-1 | hypothetical protein FG00743.1 |
| Contig15037 | 117 | 5.00E-32 | 48/69 (69%) | Gibberella zeae PH-1 | hypothetical protein FG00744.1 |
| Contig16678 | 112 | 1.00E-23 | 68/133 (51%) | Gibberella zeae PH-1 | hypothetical protein FG00746.1 |
| FQ4QJ5301A087A | 239 | 5.00E-19 | 39/72 (54%) | Gibberella zeae PH-1 | hypothetical protein FG00754.1 |
| FQ92HJ001BR8DR | 78 | 2.30E+00 | 15/18 (83%) | Gibberella zeae PH-1 | hypothetical protein FG00754.1 |
| FQ4QJ5301A4NMY | 176 | 1.00E-11 | 35/65 (53%) | Gibberella zeae PH-1 | hypothetical protein FG00758.1 |
| Contig1726 | 38.5 | 1.50E-01 | 20/44 (45%) | Gibberella zeae PH-1 | hypothetical protein FG00776.1 |
| FQ4QJ5301B27GA | 357 | 1.00E-32 | 67/70 (95%) | Gibberella zeae PH-1 | hypothetical protein FG00781.1 |
| Contig1107 | 117 | 3.00E-25 | 53/77 (68%) | Gibberella zeae PH-1 | hypothetical protein FG00797.1 |
| Contig5347 | 132 | 8.00E-30 | 66/66 (100%) | Gibberella zeae PH-1 | hypothetical protein FG00802.1 |
| Contig1197 | 106 | 5.00E-22 | 50/81 (61%) | Gibberella zeae PH-1 | hypothetical protein FG00809.1 |
| Contig11028 | 53.9 | 4.00E-06 | 25/34 (73%) | Gibberella zeae PH-1 | hypothetical protein FG00816.1 |
| FQ92HJ001EHVU1 | 356 | 1.00E-32 | 66/80 (82%) | Gibberella zeae PH-1 | hypothetical protein FG00816.1 |
| Contig12876 | 164 | 2.00E-39 | 84/115 (73%) | Gibberella zeae PH-1 | hypothetical protein FG00822.1 |
| Contig6751 | 49.7 | 7.00E-05 | 22/32 (68%) | Gibberella zeae PH-1 | hypothetical protein FG00847.1 |
| Contig13438 | 64.7 | 6.00E-09 | 34/63 (53%) | Gibberella zeae PH-1 | hypothetical protein FG00847.1 |
| FQ4QJ5301C0K14 | 111 | 3.00E-04 | 21/27 (77%) | Gibberella zeae PH-1 | hypothetical protein FG00855.1 |
| FQ4QJ5301CBALE | 198 | 5.00E-22 | 39/64 (60%) | Gibberella zeae PH-1 | hypothetical protein FG00857.1 |
| FQ4QJ5301D3Y7J | 96 | 2.00E-07 | 20/39 (51%) | Gibberella zeae PH-1 | hypothetical protein FG00862.1 |
| FQ92HJ001C0YUY | 121 | 2.00E-05 | 23/26 (88%) | Gibberella zeae PH-1 | hypothetical protein FG00863.1 |
| FQ4QJ5301EACUD | 382 | 1.00E-35 | 71/77 (92%) | Gibberella zeae PH-1 | hypothetical protein FG00874.1 |
| FQ4QJ5301DNC46 | 107 | 1.00E-03 | 22/70 (31%) | Gibberella zeae PH-1 | hypothetical protein FG00876.1 |
| FQ4QJ5301C331G | 248 | 4.00E-27 | 43/45 (95%) | Gibberella zeae PH-1 | hypothetical protein FG00884.1 |
| FQ92HJ001C8DAC | 137 | 3.00E-07 | 28/36 (77%) | Gibberella zeae PH-1 | hypothetical protein FG00894.1 |
| FQ4QJ5301C9LAO | 216 | 2.00E-16 | 46/69 (66%) | Gibberella zeae PH-1 | hypothetical protein FG00900.1 |
| FQ92HJ001B4FSU | 77 | 2.90E+00 | 16/23 (69%) | Gibberella zeae PH-1 | hypothetical protein FG00902.1 |
| FQ4QJ5301ESV90 | 143 | 7.00E-08 | 31/66 (46%) | Gibberella zeae PH-1 | hypothetical protein FG00908.1 |
| FQ4QJ5301DMC11 | 155 | 7.00E-10 | 27/41 (65%) | Gibberella zeae PH-1 | hypothetical protein FG00909.1 |
| Contig13322 | 149 | 6.00E-35 | 68/103 (66%) | Gibberella zeae PH-1 | hypothetical protein FG00913.1 |
| FQ4QJ5301EAT7R | 292 | 3.00E-25 | 53/79 (67%) | Gibberella zeae PH-1 | hypothetical protein FG00930.1 |
| FQ4QJ5301CM78K | 297 | 9.00E-26 | 53/67 (79%) | Gibberella zeae PH-1 | hypothetical protein FG00941.1 |
| Contig9802 | 133 | 5.00E-30 | 65/76 (85%) | Gibberella zeae PH-1 | hypothetical protein FG00969.1 |
| Contig13151 | 175 | 8.00E-43 | 87/92 (94%) | Gibberella zeae PH-1 | hypothetical protein FG00969.1 |
| FQ4QJ5301ATVNI | 326 | 4.00E-29 | 66/76 (86%) | Gibberella zeae PH-1 | hypothetical protein FG00969.1 |
| FQ4QJ5301ATI1Q | 85 | 3.40E-01 | 22/63 (34%) | Gibberella zeae PH-1 | hypothetical protein FG00970.1 |
| Contig9826 | 121 | 2.00E-26 | 54/77 (70%) | Gibberella zeae PH-1 | hypothetical protein FG01008.1 |
| Contig8788 | 127 | 3.00E-28 | 59/78 (75%) | Gibberella zeae PH-1 | hypothetical protein FG01016.1 |
| FQ4QJ5301ECAK0 | 176 | 1.00E-11 | 35/40 (87%) | Gibberella zeae PH-1 | hypothetical protein FG01016.1 |
| FQ92HJ001CEN1C | 284 | 3.00E-24 | 60/68 (88%) | Gibberella zeae PH-1 | hypothetical protein FG01016.1 |
| Contig3394 | 166 | 5.00E-40 | 85/98 (86%) | Gibberella zeae PH-1 | hypothetical protein FG01021.1 |
| FQ4QJ5301DVTVX | 208 | 2.00E-15 | 43/76 (56%) | Gibberella zeae PH-1 | hypothetical protein FG01025.1 |
| Contig11050 | 40 | 5.40E-02 | 20/37 (54%) | Gibberella zeae PH-1 | hypothetical protein FG01029.1 |
| FQ4QJ5301D3KGT | 129 | 3.00E-09 | 23/27 (85%) | Gibberella zeae PH-1 | hypothetical protein FG01032.1 |
| Contig432 | 101 | 1.00E-20 | 47/80 (58%) | Gibberella zeae PH-1 | hypothetical protein FG01035.1 |
| FQ4QJ5301BKRY0 | 278 | 1.00E-23 | 53/83 (63%) | Gibberella zeae PH-1 | hypothetical protein FG01037.1 |
| FQ4QJ5301A1Z7T | 231 | 4.00E-18 | 45/68 (66%) | Gibberella zeae PH-1 | hypothetical protein FG01053.1 |
| FQ4QJ5301A0NE3 | 281 | 6.00E-24 | 51/58 (87%) | Gibberella zeae PH-1 | hypothetical protein FG01059.1 |
| Contig14778 | 76.6 | 5.00E-13 | 33/42 (78%) | Gibberella zeae PH-1 | hypothetical protein FG01063.1 |
| Contig7571 | 211 | 1.00E-53 | 108/111 (97%) | Gibberella zeae PH-1 | hypothetical protein FG01081.1 |
| FQ92HJ001BQ8ER | 137 | 3.00E-07 | 24/31 (77%) | Gibberella zeae PH-1 | hypothetical protein FG01081.1 |
| Contig16527 | 53.5 | 5.00E-06 | 25/33 (75%) | Gibberella zeae PH-1 | hypothetical protein FG01082.1 |
| Contig2535 | 37.7 | 2.60E-01 | 21/50 (42%) | Gibberella zeae PH-1 | hypothetical protein FG01085.1 |
| FQ92HJ001ELECU | 221 | 6.00E-17 | 33/69 (47%) | Gibberella zeae PH-1 | hypothetical protein FG01085.1 |
| FQ92HJ001AL3WT | 156 | 2.00E-09 | 32/49 (65%) | Gibberella zeae PH-1 | hypothetical protein FG01091.1 |
| FQ4QJ5301DN63V | 85 | 3.50E-01 | 17/46 (36%) | Gibberella zeae PH-1 | hypothetical protein FG01095.1 |
| Contig8226 | 52 | 1.00E-05 | 27/50 (54%) | Gibberella zeae PH-1 | hypothetical protein FG01106.1 |
| FQ92HJ001EM18O | 59 | 2.30E+00 | 12/12 (100%) | Gibberella zeae PH-1 | hypothetical protein FG01111.1 |
| FQ4QJ5301BSGNS | 327 | 3.00E-29 | 57/78 (73%) | Gibberella zeae PH-1 | hypothetical protein FG01112.1 |
| Contig3206 | 79 | 1.00E-13 | 39/41 (95%) | Gibberella zeae PH-1 | hypothetical protein FG01123.1 |
| FQ4QJ5301B9AQ9 | 108 | 8.00E-04 | 21/21 (100%) | Gibberella zeae PH-1 | hypothetical protein FG01124.1 |
| FQ4QJ5301A8QXZ | 155 | 3.00E-09 | 32/59 (54%) | Gibberella zeae PH-1 | hypothetical protein FG01129.1 |
| FQ4QJ5301BI5FR | 274 | 4.00E-23 | 50/74 (67%) | Gibberella zeae PH-1 | hypothetical protein FG01130.1 |
| FQ4QJ5301CTNZ5 | 141 | 1.00E-07 | 31/64 (48%) | Gibberella zeae PH-1 | hypothetical protein FG01130.1 |
| Contig1970 | 179 | 7.00E-44 | 90/133 (67%) | Gibberella zeae PH-1 | hypothetical protein FG01136.1 |
| FQ4QJ5301EC6EJ | 159 | 3.00E-14 | 30/54 (55%) | Gibberella zeae PH-1 | hypothetical protein FG01142.1 |
| FQ4QJ5301BBUL9 | 173 | 5.00E-25 | 32/43 (74%) | Gibberella zeae PH-1 | hypothetical protein FG01150.1 |
| FQ92HJ001EL802 | 414 | 2.00E-39 | 80/83 (96%) | Gibberella zeae PH-1 | hypothetical protein FG01157.1 |
| FQ4QJ5301DD8YW | 239 | 5.00E-19 | 48/88 (54%) | Gibberella zeae PH-1 | hypothetical protein FG01167.1 |
| FQ4QJ5301CQGI1 | 368 | 5.00E-34 | 67/82 (81%) | Gibberella zeae PH-1 | hypothetical protein FG01193.1 |
| FQ4QJ5301CGRIU | 378 | 4.00E-35 | 66/79 (83%) | Gibberella zeae PH-1 | hypothetical protein FG01235.1 |
| Contig11559 | 46.2 | 7.00E-04 | 18/21 (85%) | Gibberella zeae PH-1 | hypothetical protein FG01236.1 |
| Contig15646 | 226 | 4.00E-58 | 108/117 (92%) | Gibberella zeae PH-1 | hypothetical protein FG01236.1 |
| Contig12657 | 35.8 | 1.00E+00 | 15/26 (57%) | Gibberella zeae PH-1 | hypothetical protein FG01247.1 |
| FQ4QJ5301BZEGO | 327 | 3.00E-29 | 57/77 (74%) | Gibberella zeae PH-1 | hypothetical protein FG01248.1 |
| FQ4QJ5301E0LUZ | 248 | 4.00E-20 | 44/86 (51%) | Gibberella zeae PH-1 | hypothetical protein FG01254.1 |
| Contig3662 | 99 | 1.00E-19 | 59/123 (47%) | Gibberella zeae PH-1 | hypothetical protein FG01262.1 |
| Contig9071 | 46.6 | 6.00E-04 | 21/22 (95%) | Gibberella zeae PH-1 | hypothetical protein FG01262.1 |
| FQ92HJ001BYMLM | 238 | 6.00E-19 | 41/53 (77%) | Gibberella zeae PH-1 | hypothetical protein FG01265.1 |
| Contig6855 | 91.3 | 2.00E-17 | 40/62 (64%) | Gibberella zeae PH-1 | hypothetical protein FG01267.1 |
| FQ92HJ001CJCRU | 260 | 2.00E-21 | 46/80 (57%) | Gibberella zeae PH-1 | hypothetical protein FG01268.1 |
| FQ4QJ5301B482B | 129 | 3.00E-06 | 29/68 (42%) | Gibberella zeae PH-1 | hypothetical protein FG01270.1 |
| FQ4QJ5301CI6TX | 234 | 2.00E-18 | 42/65 (64%) | Gibberella zeae PH-1 | hypothetical protein FG01275.1 |
| FQ4QJ5301A9ZWD | 190 | 2.00E-13 | 36/41 (87%) | Gibberella zeae PH-1 | hypothetical protein FG01278.1 |
| FQ4QJ5301C9XDQ | 84 | 4.50E-01 | 16/22 (72%) | Gibberella zeae PH-1 | hypothetical protein FG01299.1 |
| FQ4QJ5301EF4ZI | 79 | 1.70E+00 | 27/76 (35%) | Gibberella zeae PH-1 | hypothetical protein FG01299.1 |
| FQ92HJ001ECIVJ | 122 | 2.00E-05 | 23/28 (82%) | Gibberella zeae PH-1 | hypothetical protein FG01306.1 |
| Contig1425 | 124 | 2.00E-27 | 59/82 (71%) | Gibberella zeae PH-1 | hypothetical protein FG01309.1 |
| FQ4QJ5301CPCEM | 328 | 2.00E-29 | 61/86 (70%) | Gibberella zeae PH-1 | hypothetical protein FG01309.1 |
| FQ4QJ5301DWU72 | 363 | 2.00E-33 | 67/74 (90%) | Gibberella zeae PH-1 | hypothetical protein FG01314.1 |
| FQ4QJ5301DVTQ4 | 64 | 5.30E-01 | 11/12 (91%) | Gibberella zeae PH-1 | hypothetical protein FG01315.1 |
| FQ4QJ5301CHR2U | 110 | 4.00E-04 | 26/55 (47%) | Gibberella zeae PH-1 | hypothetical protein FG01316.1 |
| Contig5529 | 45.8 | 1.00E-03 | 20/32 (62%) | Gibberella zeae PH-1 | hypothetical protein FG01318.1 |
| Contig4027 | 102 | 1.00E-28 | 51/54 (94%) | Gibberella zeae PH-1 | hypothetical protein FG01329.1 |
| Contig8303 | 99 | 1.00E-19 | 48/66 (72%) | Gibberella zeae PH-1 | hypothetical protein FG01329.1 |
| FQ92HJ001A3ER0 | 164 | 2.00E-10 | 32/45 (71%) | Gibberella zeae PH-1 | hypothetical protein FG01330.1 |
| Contig12452 | 33.5 | 5.00E+00 | 23/59 (38%) | Gibberella zeae PH-1 | hypothetical protein FG01335.1 |
| Contig16829 | 42 | 1.40E-02 | 20/47 (42%) | Gibberella zeae PH-1 | hypothetical protein FG01335.1 |
| FQ4QJ5301C6VQU | 220 | 8.00E-17 | 45/57 (78%) | Gibberella zeae PH-1 | hypothetical protein FG01339.1 |
| Contig16763 | 54.7 | 2.00E-06 | 31/87 (35%) | Gibberella zeae PH-1 | hypothetical protein FG01351.1 |
| FQ4QJ5301CG60A | 177 | 7.00E-12 | 40/82 (48%) | Gibberella zeae PH-1 | hypothetical protein FG01357.1 |
| FQ92HJ001EHK93 | 83 | 5.80E-01 | 17/37 (45%) | Gibberella zeae PH-1 | hypothetical protein FG01357.1 |
| FQ92HJ001CLKKS | 204 | 5.00E-15 | 42/80 (52%) | Gibberella zeae PH-1 | hypothetical protein FG01370.1 |
| Contig6584 | 139 | 7.00E-32 | 66/87 (75%) | Gibberella zeae PH-1 | hypothetical protein FG01371.1 |
| FQ4QJ5301DV42S | 236 | 5.00E-27 | 44/46 (95%) | Gibberella zeae PH-1 | hypothetical protein FG01371.1 |
| FQ4QJ5301EAQDI | 210 | 1.00E-15 | 44/82 (53%) | Gibberella zeae PH-1 | hypothetical protein FG01376.1 |
| Contig9774 | 137 | 2.00E-31 | 66/78 (84%) | Gibberella zeae PH-1 | hypothetical protein FG01388.1 |
| FQ4QJ5301D7ET8 | 375 | 8.00E-35 | 70/76 (92%) | Gibberella zeae PH-1 | hypothetical protein FG01391.1 |
| FQ4QJ5301EYWWN | 355 | 2.00E-32 | 72/86 (83%) | Gibberella zeae PH-1 | hypothetical protein FG01392.1 |
| FQ4QJ5301EQENY | 337 | 2.00E-30 | 57/88 (64%) | Gibberella zeae PH-1 | hypothetical protein FG01395.1 |
| FQ4QJ5301BYTNK | 170 | 2.00E-16 | 33/65 (50%) | Gibberella zeae PH-1 | hypothetical protein FG01412.1 |
| FQ92HJ001DP2SD | 295 | 2.00E-25 | 51/87 (58%) | Gibberella zeae PH-1 | hypothetical protein FG01415.1 |
| FQ4QJ5301DUJA2 | 103 | 3.00E-03 | 21/47 (44%) | Gibberella zeae PH-1 | hypothetical protein FG01420.1 |
| FQ92HJ001C02PK | 80 | 1.30E+00 | 20/47 (42%) | Gibberella zeae PH-1 | hypothetical protein FG01424.1 |
| FQ92HJ001DV1NP | 283 | 4.00E-24 | 51/74 (68%) | Gibberella zeae PH-1 | hypothetical protein FG01448.1 |
| FQ4QJ5301CMV7K | 351 | 5.00E-32 | 64/93 (68%) | Gibberella zeae PH-1 | hypothetical protein FG01449.1 |
| FQ92HJ001B5WBK | 301 | 3.00E-26 | 53/77 (68%) | Gibberella zeae PH-1 | hypothetical protein FG01449.1 |
| Contig8300 | 111 | 2.00E-23 | 55/86 (63%) | Gibberella zeae PH-1 | hypothetical protein FG01470.1 |
| FQ4QJ5301DFPZ7 | 86 | 2.60E-01 | 16/19 (84%) | Gibberella zeae PH-1 | hypothetical protein FG01510.1 |
| Contig13402 | 123 | 9.00E-28 | 56/62 (90%) | Gibberella zeae PH-1 | hypothetical protein FG01516.1 |
| Contig4161 | 78.2 | 2.00E-13 | 31/64 (48%) | Gibberella zeae PH-1 | hypothetical protein FG01519.1 |
| FQ92HJ001B8J85 | 193 | 1.00E-13 | 38/61 (62%) | Gibberella zeae PH-1 | hypothetical protein FG01521.1 |
| FQ4QJ5301DS71T | 203 | 7.00E-15 | 39/46 (84%) | Gibberella zeae PH-1 | hypothetical protein FG01526.1 |
| FQ4QJ5301CBVMD | 178 | 9.00E-23 | 35/39 (89%) | Gibberella zeae PH-1 | hypothetical protein FG01552.1 |
| Contig16342 | 64.7 | 2.00E-09 | 32/40 (80%) | Gibberella zeae PH-1 | hypothetical protein FG01573.1 |
| FQ4QJ5301A491S | 297 | 9.00E-26 | 57/80 (71%) | Gibberella zeae PH-1 | hypothetical protein FG01573.1 |
| FQ4QJ5301B5OZA | 348 | 1.00E-31 | 62/79 (78%) | Gibberella zeae PH-1 | hypothetical protein FG01573.1 |
| FQ4QJ5301AXEA9 | 235 | 3.00E-29 | 41/53 (77%) | Gibberella zeae PH-1 | hypothetical protein FG01573.1 |
| FQ4QJ5301A6W39 | 165 | 2.00E-10 | 30/38 (78%) | Gibberella zeae PH-1 | hypothetical protein FG01575.1 |
| Contig7072 | 177 | 9.00E-48 | 80/90 (88%) | Gibberella zeae PH-1 | hypothetical protein FG01576.1 |
| Contig11697 | 62.8 | 8.00E-09 | 35/79 (44%) | Gibberella zeae PH-1 | hypothetical protein FG01580.1 |
| FQ4QJ5301CPEW7 | 245 | 1.00E-19 | 47/76 (61%) | Gibberella zeae PH-1 | hypothetical protein FG01600.1 |
| Contig2950 | 94.4 | 2.00E-18 | 39/62 (62%) | Gibberella zeae PH-1 | hypothetical protein FG01601.1 |
| Contig5994 | 106 | 5.00E-22 | 47/73 (64%) | Gibberella zeae PH-1 | hypothetical protein FG01601.1 |
| FQ4QJ5301BR02J | 203 | 7.00E-15 | 39/67 (58%) | Gibberella zeae PH-1 | hypothetical protein FG01601.1 |
| FQ92HJ001CDK6O | 206 | 3.00E-15 | 38/46 (82%) | Gibberella zeae PH-1 | hypothetical protein FG01601.1 |
| FQ92HJ001BLRGF | 113 | 2.00E-04 | 21/23 (91%) | Gibberella zeae PH-1 | hypothetical protein FG01622.1 |
| FQ4QJ5301AJMY1 | 388 | 3.00E-36 | 70/84 (83%) | Gibberella zeae PH-1 | hypothetical protein FG01632.1 |
| Contig15123 | 99.8 | 6.00E-20 | 43/49 (87%) | Gibberella zeae PH-1 | hypothetical protein FG01640.1 |
| Contig431 | 110 | 3.00E-23 | 53/77 (68%) | Gibberella zeae PH-1 | hypothetical protein FG01643.1 |
| FQ4QJ5301BHAIT | 332 | 8.00E-30 | 63/85 (74%) | Gibberella zeae PH-1 | hypothetical protein FG01647.1 |
| FQ92HJ001D3YPM | 350 | 6.00E-32 | 65/80 (81%) | Gibberella zeae PH-1 | hypothetical protein FG01656.1 |
| Contig14165 | 69.3 | 8.00E-11 | 34/47 (72%) | Gibberella zeae PH-1 | hypothetical protein FG01692.1 |
| FQ4QJ5301C6Y07 | 128 | 4.00E-06 | 33/81 (40%) | Gibberella zeae PH-1 | hypothetical protein FG01692.1 |
| Contig6538 | 71.6 | 2.00E-11 | 57/171 (33%) | Gibberella zeae PH-1 | hypothetical protein FG01709.1 |
| FQ4QJ5301BGXGC | 79 | 2.00E-03 | 14/19 (73%) | Gibberella zeae PH-1 | hypothetical protein FG01732.1 |
| FQ92HJ001CXCDD | 202 | 9.00E-15 | 36/82 (43%) | Gibberella zeae PH-1 | hypothetical protein FG01824.1 |
| FQ4QJ5301EMOHA | 286 | 4.00E-25 | 51/68 (75%) | Gibberella zeae PH-1 | hypothetical protein FG01839.1 |
| Contig3646 | 137 | 2.00E-31 | 66/84 (78%) | Gibberella zeae PH-1 | hypothetical protein FG01846.1 |
| FQ4QJ5301CMMJY | 251 | 2.00E-20 | 48/78 (61%) | Gibberella zeae PH-1 | hypothetical protein FG01846.1 |
| FQ4QJ5301BHN03 | 197 | 4.00E-14 | 39/53 (73%) | Gibberella zeae PH-1 | hypothetical protein FG01873.1 |
| Contig8524 | 68.2 | 4.00E-16 | 31/51 (60%) | Gibberella zeae PH-1 | hypothetical protein FG01888.1 |
| FQ4QJ5301CKXJ0 | 367 | 7.00E-34 | 71/74 (95%) | Gibberella zeae PH-1 | hypothetical protein FG01904.1 |
| FQ92HJ001BTEU2 | 122 | 2.00E-05 | 28/50 (56%) | Gibberella zeae PH-1 | hypothetical protein FG01904.1 |
| FQ92HJ001CKTKC | 209 | 1.00E-15 | 42/67 (62%) | Gibberella zeae PH-1 | hypothetical protein FG01907.1 |
| Contig15488 | 89 | 1.00E-16 | 38/58 (65%) | Gibberella zeae PH-1 | hypothetical protein FG01912.1 |
| FQ4QJ5301EUHZ2 | 126 | 6.00E-06 | 23/28 (82%) | Gibberella zeae PH-1 | hypothetical protein FG01932.1 |
| FQ92HJ001EUZ7D | 315 | 7.00E-28 | 59/69 (85%) | Gibberella zeae PH-1 | hypothetical protein FG01932.1 |
| FQ4QJ5301DZFXA | 98 | 1.10E-02 | 27/77 (35%) | Gibberella zeae PH-1 | hypothetical protein FG01945.1 |
| FQ4QJ5301A6MXZ | 186 | 7.00E-13 | 43/76 (56%) | Gibberella zeae PH-1 | hypothetical protein FG01948.1 |
| FQ4QJ5301DAJSU | 356 | 1.00E-32 | 65/81 (80%) | Gibberella zeae PH-1 | hypothetical protein FG01950.1 |
| FQ4QJ5301CBOTA | 360 | 4.00E-33 | 69/80 (86%) | Gibberella zeae PH-1 | hypothetical protein FG01950.1 |
| Contig13997 | 62 | 1.00E-08 | 33/80 (41%) | Gibberella zeae PH-1 | hypothetical protein FG01952.1 |
| FQ92HJ001AX11M | 116 | 9.00E-05 | 21/31 (67%) | Gibberella zeae PH-1 | hypothetical protein FG01969.1 |
| FQ4QJ5301BES83 | 100 | 1.00E-04 | 18/30 (60%) | Gibberella zeae PH-1 | hypothetical protein FG01975.1 |
| FQ4QJ5301BE35A | 137 | 3.00E-07 | 24/46 (52%) | Gibberella zeae PH-1 | hypothetical protein FG01981.1 |
| FQ92HJ001CA6A5 | 228 | 9.00E-18 | 49/79 (62%) | Gibberella zeae PH-1 | hypothetical protein FG02009.1 |
| FQ4QJ5301ET816 | 230 | 5.00E-18 | 43/53 (81%) | Gibberella zeae PH-1 | hypothetical protein FG02010.1 |
| FQ4QJ5301EVIPG | 172 | 3.00E-11 | 32/53 (60%) | Gibberella zeae PH-1 | hypothetical protein FG02018.1 |
| FQ4QJ5301CGP81 | 222 | 4.00E-17 | 43/48 (89%) | Gibberella zeae PH-1 | hypothetical protein FG02028.1 |
| FQ4QJ5301D7KZV | 84 | 4.50E-01 | 16/18 (88%) | Gibberella zeae PH-1 | hypothetical protein FG02030.1 |
| Contig12172 | 53.1 | 6.00E-06 | 23/35 (65%) | Gibberella zeae PH-1 | hypothetical protein FG02032.1 |
| Contig6558 | 104 | 2.00E-25 | 49/59 (83%) | Gibberella zeae PH-1 | hypothetical protein FG02056.1 |
| Contig2174 | 88.6 | 6.00E-16 | 35/63 (55%) | Gibberella zeae PH-1 | hypothetical protein FG02077.1 |
| Contig2144 | 73.2 | 6.00E-12 | 35/81 (43%) | Gibberella zeae PH-1 | hypothetical protein FG02093.1 |
| FQ92HJ001BFEDL | 209 | 1.00E-15 | 43/76 (56%) | Gibberella zeae PH-1 | hypothetical protein FG02093.1 |
| Contig8525 | 72 | 1.00E-11 | 34/41 (82%) | Gibberella zeae PH-1 | hypothetical protein FG02096.1 |
| Contig6734 | 142 | 1.00E-32 | 68/106 (64%) | Gibberella zeae PH-1 | hypothetical protein FG02097.1 |
| Contig4624 | 94.7 | 2.00E-18 | 44/72 (61%) | Gibberella zeae PH-1 | hypothetical protein FG02127.1 |
| FQ4QJ5301BUJAX | 239 | 5.00E-19 | 47/52 (90%) | Gibberella zeae PH-1 | hypothetical protein FG02146.1 |
| Contig15952 | 238 | 1.00E-60 | 123/246 (50%) | Gibberella zeae PH-1 | hypothetical protein FG02173.1 |
| FQ4QJ5301EIBG8 | 143 | 7.00E-08 | 25/54 (46%) | Gibberella zeae PH-1 | hypothetical protein FG02197.1 |
| FQ4QJ5301AN50G | 396 | 3.00E-37 | 71/80 (88%) | Gibberella zeae PH-1 | hypothetical protein FG02432.1 |
| FQ92HJ001BD2NR | 121 | 2.00E-05 | 23/37 (62%) | Gibberella zeae PH-1 | hypothetical protein FG02432.1 |
| FQ92HJ001BMB14 | 93 | 4.00E-02 | 16/31 (51%) | Gibberella zeae PH-1 | hypothetical protein FG02446.1 |
| FQ4QJ5301E0M0N | 218 | 1.00E-16 | 42/66 (63%) | Gibberella zeae PH-1 | hypothetical protein FG02453.1 |
| FQ4QJ5301BAB3H | 216 | 2.00E-17 | 41/62 (66%) | Gibberella zeae PH-1 | hypothetical protein FG02454.1 |
| Contig16734 | 34.7 | 2.20E+00 | 13/34 (38%) | Gibberella zeae PH-1 | hypothetical protein FG02458.1 |
| FQ4QJ5301BZ8F2 | 379 | 3.00E-35 | 75/81 (92%) | Gibberella zeae PH-1 | hypothetical protein FG02461.1 |
| FQ4QJ5301CSTHY | 372 | 2.00E-34 | 70/79 (88%) | Gibberella zeae PH-1 | hypothetical protein FG02470.1 |
| FQ4QJ5301DVU3H | 175 | 1.00E-21 | 33/38 (86%) | Gibberella zeae PH-1 | hypothetical protein FG02470.1 |
| Contig16778 | 128 | 1.00E-28 | 58/88 (65%) | Gibberella zeae PH-1 | hypothetical protein FG02494.1 |
| FQ4QJ5301CV9W8 | 418 | 8.00E-40 | 72/72 (100%) | Gibberella zeae PH-1 | hypothetical protein FG02497.1 |
| FQ4QJ5301CDPNK | 98 | 1.10E-02 | 18/38 (47%) | Gibberella zeae PH-1 | hypothetical protein FG02500.1 |
| Contig5293 | 111 | 2.00E-23 | 51/70 (72%) | Gibberella zeae PH-1 | hypothetical protein FG02503.1 |
| FQ4QJ5301E1E8D | 259 | 2.00E-21 | 48/64 (75%) | Gibberella zeae PH-1 | hypothetical protein FG02509.1 |
| FQ4QJ5301AM7H4 | 86 | 2.70E-01 | 16/22 (72%) | Gibberella zeae PH-1 | hypothetical protein FG02511.1 |
| FQ92HJ001AQOP6 | 281 | 6.00E-24 | 58/82 (70%) | Gibberella zeae PH-1 | hypothetical protein FG02523.1 |
| FQ4QJ5301AK1QG | 113 | 2.00E-04 | 21/28 (75%) | Gibberella zeae PH-1 | hypothetical protein FG02531.1 |
| FQ4QJ5301EXSDY | 123 | 1.00E-05 | 31/82 (37%) | Gibberella zeae PH-1 | hypothetical protein FG02534.1 |
| FQ4QJ5301DTNOS | 163 | 3.00E-10 | 32/40 (80%) | Gibberella zeae PH-1 | hypothetical protein FG02542.1 |
| FQ4QJ5301DT25K | 242 | 2.00E-19 | 43/73 (58%) | Gibberella zeae PH-1 | hypothetical protein FG02543.1 |
| FQ4QJ5301BGAF3 | 212 | 7.00E-16 | 39/56 (69%) | Gibberella zeae PH-1 | hypothetical protein FG02563.1 |
| FQ4QJ5301ER3ML | 231 | 4.00E-18 | 42/49 (85%) | Gibberella zeae PH-1 | hypothetical protein FG02565.1 |
| FQ4QJ5301B9TFX | 312 | 2.00E-27 | 58/64 (90%) | Gibberella zeae PH-1 | hypothetical protein FG02571.1 |
| FQ4QJ5301E00YR | 321 | 1.00E-28 | 64/78 (82%) | Gibberella zeae PH-1 | hypothetical protein FG02571.1 |
| FQ4QJ5301AQQ47 | 138 | 2.00E-07 | 27/73 (36%) | Gibberella zeae PH-1 | hypothetical protein FG02576.1 |
| Contig14455 | 41.6 | 3.00E-08 | 19/30 (63%) | Gibberella zeae PH-1 | hypothetical protein FG02593.1 |
| FQ4QJ5301B0GGI | 181 | 3.00E-12 | 38/68 (55%) | Gibberella zeae PH-1 | hypothetical protein FG02593.1 |
| FQ4QJ5301D79V6 | 89 | 1.40E-02 | 16/20 (80%) | Gibberella zeae PH-1 | hypothetical protein FG02593.1 |
| FQ92HJ001CKG8K | 253 | 1.00E-20 | 47/68 (69%) | Gibberella zeae PH-1 | hypothetical protein FG02595.1 |
| Contig4759 | 89.7 | 6.00E-17 | 45/95 (47%) | Gibberella zeae PH-1 | hypothetical protein FG02605.1 |
| Contig6324 | 73.6 | 9.00E-12 | 45/116 (38%) | Gibberella zeae PH-1 | hypothetical protein FG02607.1 |
| FQ92HJ001DIIJN | 192 | 1.00E-13 | 37/66 (56%) | Gibberella zeae PH-1 | hypothetical protein FG02626.1 |
| FQ92HJ001EMAEM | 285 | 2.00E-24 | 52/69 (75%) | Gibberella zeae PH-1 | hypothetical protein FG02637.1 |
| FQ92HJ001BHEBU | 118 | 5.00E-05 | 21/26 (80%) | Gibberella zeae PH-1 | hypothetical protein FG02637.1 |
| FQ4QJ5301C7M98 | 293 | 3.00E-25 | 52/64 (81%) | Gibberella zeae PH-1 | hypothetical protein FG02640.1 |
| FQ4QJ5301E4MNK | 128 | 4.00E-06 | 22/27 (81%) | Gibberella zeae PH-1 | hypothetical protein FG02702.1 |
| FQ4QJ5301BZU2Y | 128 | 4.00E-06 | 25/54 (46%) | Gibberella zeae PH-1 | hypothetical protein FG02706.1 |
| FQ4QJ5301ARR8F | 209 | 1.00E-15 | 39/72 (54%) | Gibberella zeae PH-1 | hypothetical protein FG02721.1 |
| FQ4QJ5301AYG55 | 225 | 2.00E-17 | 37/71 (52%) | Gibberella zeae PH-1 | hypothetical protein FG02725.1 |
| FQ92HJ001B4EDR | 168 | 8.00E-11 | 29/40 (72%) | Gibberella zeae PH-1 | hypothetical protein FG02726.1 |
| Contig12907 | 111 | 2.00E-23 | 52/80 (65%) | Gibberella zeae PH-1 | hypothetical protein FG02767.1 |
| Contig3084 | 100 | 3.00E-20 | 51/67 (76%) | Gibberella zeae PH-1 | hypothetical protein FG02770.1 |
| FQ4QJ5301EWJDA | 331 | 1.00E-29 | 64/75 (85%) | Gibberella zeae PH-1 | hypothetical protein FG02770.1 |
| FQ4QJ5301COC0L | 252 | 2.00E-20 | 48/75 (64%) | Gibberella zeae PH-1 | hypothetical protein FG02771.1 |
| FQ4QJ5301DB7Z8 | 166 | 1.00E-10 | 34/84 (40%) | Gibberella zeae PH-1 | hypothetical protein FG02775.1 |
| FQ92HJ001EIS43 | 180 | 3.00E-12 | 36/80 (45%) | Gibberella zeae PH-1 | hypothetical protein FG02775.1 |
| Contig1430 | 42.4 | 1.10E-02 | 20/46 (43%) | Gibberella zeae PH-1 | hypothetical protein FG02778.1 |
| FQ4QJ5301D6AAO | 377 | 5.00E-35 | 74/85 (87%) | Gibberella zeae PH-1 | hypothetical protein FG02782.1 |
| FQ92HJ001ASUWR | 103 | 3.00E-03 | 35/64 (54%) | Gibberella zeae PH-1 | hypothetical protein FG02782.1 |
| FQ4QJ5301BR7SD | 289 | 8.00E-25 | 51/75 (68%) | Gibberella zeae PH-1 | hypothetical protein FG02783.1 |
| Contig12685 | 73.9 | 3.00E-12 | 32/56 (57%) | Gibberella zeae PH-1 | hypothetical protein FG02784.1 |
| FQ4QJ5301D16OL | 137 | 3.00E-07 | 26/42 (61%) | Gibberella zeae PH-1 | hypothetical protein FG02786.1 |
| FQ4QJ5301CUP2K | 213 | 5.00E-16 | 42/61 (68%) | Gibberella zeae PH-1 | hypothetical protein FG02787.1 |
| FQ4QJ5301BNPC1 | 171 | 4.00E-11 | 30/67 (44%) | Gibberella zeae PH-1 | hypothetical protein FG02811.1 |
| FQ92HJ001EPNEX | 208 | 2.00E-15 | 36/53 (67%) | Gibberella zeae PH-1 | hypothetical protein FG03028.1 |
| FQ4QJ5301B5OWJ | 132 | 1.00E-06 | 24/61 (39%) | Gibberella zeae PH-1 | hypothetical protein FG03035.1 |
| FQ4QJ5301DGUNW | 77 | 2.90E+00 | 18/50 (36%) | Gibberella zeae PH-1 | hypothetical protein FG03106.1 |
| Contig1583 | 138 | 1.00E-31 | 65/79 (82%) | Gibberella zeae PH-1 | hypothetical protein FG03180.1 |
| Contig10420 | 40.8 | 3.10E-02 | 16/22 (72%) | Gibberella zeae PH-1 | hypothetical protein FG03180.1 |
| Contig13861 | 179 | 6.00E-44 | 80/103 (77%) | Gibberella zeae PH-1 | hypothetical protein FG03180.1 |
| FQ92HJ001C5QQG | 90 | 9.30E-02 | 17/22 (77%) | Gibberella zeae PH-1 | hypothetical protein FG03257.1 |
| Contig2091 | 158 | 1.00E-37 | 69/118 (58%) | Gibberella zeae PH-1 | hypothetical protein FG03405.1 |
| FQ4QJ5301EAH2Z | 287 | 1.00E-24 | 52/86 (60%) | Gibberella zeae PH-1 | hypothetical protein FG03405.1 |
| FQ92HJ001CI2F0 | 235 | 1.00E-18 | 45/79 (56%) | Gibberella zeae PH-1 | hypothetical protein FG03445.1 |
| Contig5380 | 33.5 | 5.00E+00 | 15/25 (60%) | Gibberella zeae PH-1 | hypothetical protein FG03879.1 |
| Contig11721 | 33.1 | 6.50E+00 | 19/61 (31%) | Gibberella zeae PH-1 | hypothetical protein FG03919.1 |
| FQ4QJ5301DMH7X | 87 | 2.00E-01 | 21/63 (33%) | Gibberella zeae PH-1 | hypothetical protein FG03997.1 |
| FQ4QJ5301BP92B | 123 | 1.00E-05 | 25/58 (43%) | Gibberella zeae PH-1 | hypothetical protein FG04023.1 |
| Contig2230 | 45.1 | 2.00E-03 | 21/40 (52%) | Gibberella zeae PH-1 | hypothetical protein FG04039.1 |
| FQ4QJ5301D05HB | 81 | 1.00E+00 | 18/57 (31%) | Gibberella zeae PH-1 | hypothetical protein FG04050.1 |
| FQ4QJ5301AZL05 | 92 | 5.30E-02 | 14/35 (40%) | Gibberella zeae PH-1 | hypothetical protein FG04054.1 |
| FQ4QJ5301DMGAF | 178 | 6.00E-12 | 41/82 (50%) | Gibberella zeae PH-1 | hypothetical protein FG04118.1 |
| FQ4QJ5301DQE1R | 104 | 2.00E-03 | 19/58 (32%) | Gibberella zeae PH-1 | hypothetical protein FG04122.1 |
| Contig3912 | 194 | 3.00E-48 | 94/104 (90%) | Gibberella zeae PH-1 | hypothetical protein FG04136.1 |
| Contig10080 | 87.4 | 3.00E-16 | 41/46 (89%) | Gibberella zeae PH-1 | hypothetical protein FG04136.1 |
| Contig1856 | 65.1 | 2.00E-09 | 36/76 (47%) | Gibberella zeae PH-1 | hypothetical protein FG04152.1 |
| FQ92HJ001EMXA0 | 96 | 1.80E-02 | 22/51 (43%) | Gibberella zeae PH-1 | hypothetical protein FG04168.1 |
| Contig8063 | 62.4 | 1.00E-08 | 37/71 (52%) | Gibberella zeae PH-1 | hypothetical protein FG04184.1 |
| FQ92HJ001D6QZ7 | 355 | 2.00E-32 | 64/70 (91%) | Gibberella zeae PH-1 | hypothetical protein FG04184.1 |
| FQ92HJ001B0KFG | 182 | 2.00E-12 | 41/88 (46%) | Gibberella zeae PH-1 | hypothetical protein FG04184.1 |
| Contig16041 | 46.2 | 2.00E-07 | 22/42 (52%) | Gibberella zeae PH-1 | hypothetical protein FG04186.1 |
| FQ4QJ5301BKF9H | 311 | 2.00E-27 | 57/70 (81%) | Gibberella zeae PH-1 | hypothetical protein FG04186.1 |
| FQ4QJ5301CS9W9 | 218 | 1.00E-16 | 42/70 (60%) | Gibberella zeae PH-1 | hypothetical protein FG04212.1 |
| FQ4QJ5301D5DFE | 187 | 5.00E-13 | 39/41 (95%) | Gibberella zeae PH-1 | hypothetical protein FG04232.1 |
| FQ92HJ001DLVXA | 296 | 1.00E-25 | 62/74 (83%) | Gibberella zeae PH-1 | hypothetical protein FG04232.1 |
| FQ4QJ5301CFCYE | 214 | 4.00E-16 | 42/53 (79%) | Gibberella zeae PH-1 | hypothetical protein FG04242.1 |
| FQ4QJ5301AJIWK | 83 | 6.00E-01 | 18/30 (60%) | Gibberella zeae PH-1 | hypothetical protein FG04254.1 |
| FQ4QJ5301BRRIX | 263 | 8.00E-22 | 46/84 (54%) | Gibberella zeae PH-1 | hypothetical protein FG04258.1 |
| Contig533 | 93.6 | 4.00E-18 | 48/76 (63%) | Gibberella zeae PH-1 | hypothetical protein FG04259.1 |
| FQ4QJ5301ECWI3 | 199 | 2.00E-14 | 37/86 (43%) | Gibberella zeae PH-1 | hypothetical protein FG04259.1 |
| FQ4QJ5301DQYZH | 259 | 2.00E-21 | 51/56 (91%) | Gibberella zeae PH-1 | hypothetical protein FG04261.1 |
| FQ4QJ5301D4IOL | 269 | 2.00E-22 | 51/73 (69%) | Gibberella zeae PH-1 | hypothetical protein FG04266.1 |
| FQ4QJ5301D9M1B | 260 | 2.00E-21 | 44/73 (60%) | Gibberella zeae PH-1 | hypothetical protein FG04266.1 |
| FQ4QJ5301EKM7L | 264 | 6.00E-22 | 46/78 (58%) | Gibberella zeae PH-1 | hypothetical protein FG04270.1 |
| FQ4QJ5301A3MRY | 314 | 1.00E-27 | 59/82 (71%) | Gibberella zeae PH-1 | hypothetical protein FG04270.1 |
| FQ92HJ001AF0J8 | 196 | 5.00E-14 | 44/67 (65%) | Gibberella zeae PH-1 | hypothetical protein FG04271.1 |
| Contig8738 | 127 | 3.00E-28 | 63/81 (77%) | Gibberella zeae PH-1 | hypothetical protein FG04273.1 |
| Contig12058 | 144 | 2.00E-33 | 65/75 (86%) | Gibberella zeae PH-1 | hypothetical protein FG04273.1 |
| FQ92HJ001E4JOM | 151 | 8.00E-09 | 31/63 (49%) | Gibberella zeae PH-1 | hypothetical protein FG04274.1 |
| FQ4QJ5301A0MLO | 293 | 3.00E-25 | 50/71 (70%) | Gibberella zeae PH-1 | hypothetical protein FG04275.1 |
| FQ4QJ5301CSQ8U | 119 | 4.00E-05 | 24/46 (52%) | Gibberella zeae PH-1 | hypothetical protein FG04279.1 |
| FQ4QJ5301EBP73 | 149 | 1.00E-08 | 36/91 (39%) | Gibberella zeae PH-1 | hypothetical protein FG04282.1 |
| FQ4QJ5301EDOFW | 94 | 3.10E-02 | 17/21 (80%) | Gibberella zeae PH-1 | hypothetical protein FG04287.1 |
| FQ4QJ5301BJY36 | 119 | 7.00E-10 | 23/33 (69%) | Gibberella zeae PH-1 | hypothetical protein FG04287.1 |
| FQ4QJ5301CTPSD | 326 | 4.00E-29 | 65/84 (77%) | Gibberella zeae PH-1 | hypothetical protein FG04291.1 |
| FQ4QJ5301BOLGC | 262 | 1.00E-21 | 49/69 (71%) | Gibberella zeae PH-1 | hypothetical protein FG04295.1 |
| Contig5412 | 178 | 1.00E-43 | 85/97 (87%) | Gibberella zeae PH-1 | hypothetical protein FG04310.1 |
| Contig1718 | 171 | 1.00E-41 | 78/99 (78%) | Gibberella zeae PH-1 | hypothetical protein FG04313.1 |
| Contig2589 | 144 | 3.00E-33 | 68/81 (83%) | Gibberella zeae PH-1 | hypothetical protein FG04314.1 |
| FQ4QJ5301AIRDT | 285 | 2.00E-24 | 54/77 (70%) | Gibberella zeae PH-1 | hypothetical protein FG04314.1 |
| FQ92HJ001CDPH1 | 83 | 5.90E-01 | 13/29 (44%) | Gibberella zeae PH-1 | hypothetical protein FG04315.1 |
| FQ92HJ001C7QNS | 94 | 3.20E-02 | 17/20 (85%) | Gibberella zeae PH-1 | hypothetical protein FG04327.1 |
| FQ4QJ5301EPR37 | 156 | 2.00E-09 | 34/65 (52%) | Gibberella zeae PH-1 | hypothetical protein FG04329.1 |
| FQ92HJ001DZ59D | 322 | 1.00E-28 | 61/81 (75%) | Gibberella zeae PH-1 | hypothetical protein FG04337.1 |
| FQ4QJ5301CF8YZ | 155 | 3.00E-09 | 37/74 (50%) | Gibberella zeae PH-1 | hypothetical protein FG04353.1 |
| FQ4QJ5301EJ3XM | 275 | 3.00E-23 | 53/67 (79%) | Gibberella zeae PH-1 | hypothetical protein FG04378.1 |
| FQ4QJ5301EZXK8 | 214 | 4.00E-16 | 41/85 (48%) | Gibberella zeae PH-1 | hypothetical protein FG04397.1 |
| FQ4QJ5301D57P3 | 257 | 6.00E-29 | 46/59 (77%) | Gibberella zeae PH-1 | hypothetical protein FG04404.1 |
| FQ4QJ5301CI31A | 305 | 1.00E-26 | 56/77 (72%) | Gibberella zeae PH-1 | hypothetical protein FG04404.1 |
| FQ4QJ5301EVAHD | 188 | 5.00E-15 | 37/53 (69%) | Gibberella zeae PH-1 | hypothetical protein FG04405.1 |
| FQ92HJ001BUJX5 | 154 | 4.00E-09 | 31/59 (52%) | Gibberella zeae PH-1 | hypothetical protein FG04405.1 |
| Contig8093 | 58.5 | 1.00E-07 | 26/42 (61%) | Gibberella zeae PH-1 | hypothetical protein FG04411.1 |
| Contig3219 | 60.1 | 5.00E-08 | 25/59 (42%) | Gibberella zeae PH-1 | hypothetical protein FG04412.1 |
| Contig10779 | 57.8 | 2.00E-08 | 25/27 (92%) | Gibberella zeae PH-1 | hypothetical protein FG04423.1 |
| FQ4QJ5301B9XOE | 293 | 3.00E-25 | 48/70 (68%) | Gibberella zeae PH-1 | hypothetical protein FG04451.1 |
| Contig8415 | 86.7 | 2.00E-24 | 34/46 (73%) | Gibberella zeae PH-1 | hypothetical protein FG04460.1 |
| Contig6338 | 147 | 2.00E-34 | 71/77 (92%) | Gibberella zeae PH-1 | hypothetical protein FG04476.1 |
| Contig11797 | 94.7 | 2.00E-18 | 45/50 (90%) | Gibberella zeae PH-1 | hypothetical protein FG04482.1 |
| Contig5997 | 82.4 | 9.00E-15 | 50/92 (54%) | Gibberella zeae PH-1 | hypothetical protein FG04567.1 |
| FQ92HJ001BESPR | 74 | 6.40E+00 | 14/45 (31%) | Gibberella zeae PH-1 | hypothetical protein FG04865.1 |
| FQ4QJ5301EPPU3 | 321 | 1.00E-28 | 61/83 (73%) | Gibberella zeae PH-1 | hypothetical protein FG04886.1 |
| Contig14841 | 123 | 5.00E-27 | 60/62 (96%) | Gibberella zeae PH-1 | hypothetical protein FG04915.1 |
| FQ92HJ001B7X06 | 309 | 4.00E-27 | 59/62 (95%) | Gibberella zeae PH-1 | hypothetical protein FG04915.1 |
| FQ92HJ001D0E0J | 78 | 2.30E+00 | 19/46 (41%) | Gibberella zeae PH-1 | hypothetical protein FG04939.1 |
| FQ4QJ5301BWTG6 | 190 | 2.00E-13 | 39/58 (67%) | Gibberella zeae PH-1 | hypothetical protein FG04946.1 |
| Contig16754 | 132 | 6.00E-30 | 61/84 (72%) | Gibberella zeae PH-1 | hypothetical protein FG04947.1 |
| FQ92HJ001DTZW9 | 137 | 3.00E-07 | 31/68 (45%) | Gibberella zeae PH-1 | hypothetical protein FG04986.1 |
| FQ92HJ001BAESK | 121 | 2.00E-05 | 27/54 (50%) | Gibberella zeae PH-1 | hypothetical protein FG05001.1 |
| Contig6473 | 93.6 | 4.00E-18 | 43/71 (60%) | Gibberella zeae PH-1 | hypothetical protein FG05013.1 |
| FQ92HJ001EYXA8 | 238 | 6.00E-19 | 44/62 (70%) | Gibberella zeae PH-1 | hypothetical protein FG05027.1 |
| FQ4QJ5301EW78R | 209 | 1.00E-15 | 37/47 (78%) | Gibberella zeae PH-1 | hypothetical protein FG05029.1 |
| Contig10573 | 157 | 3.00E-37 | 78/81 (96%) | Gibberella zeae PH-1 | hypothetical protein FG05035.1 |
| FQ4QJ5301A5KPW | 264 | 6.00E-22 | 51/55 (92%) | Gibberella zeae PH-1 | hypothetical protein FG05035.1 |
| Contig27 | 47.4 | 3.00E-04 | 23/45 (51%) | Gibberella zeae PH-1 | hypothetical protein FG05057.1 |
| Contig13421 | 126 | 4.00E-28 | 60/79 (75%) | Gibberella zeae PH-1 | hypothetical protein FG05057.1 |
| FQ4QJ5301C5BWP | 292 | 3.00E-25 | 58/74 (78%) | Gibberella zeae PH-1 | hypothetical protein FG05072.1 |
| FQ92HJ001DDH7Z | 148 | 2.00E-14 | 29/45 (64%) | Gibberella zeae PH-1 | hypothetical protein FG05082.1 |
| FQ4QJ5301CBPL7 | 377 | 5.00E-35 | 70/83 (84%) | Gibberella zeae PH-1 | hypothetical protein FG05083.1 |
| Contig11570 | 37.4 | 3.50E-01 | 15/32 (46%) | Gibberella zeae PH-1 | hypothetical protein FG05089.1 |
| FQ92HJ001C3DN2 | 132 | 1.00E-06 | 26/66 (39%) | Gibberella zeae PH-1 | hypothetical protein FG05092.1 |
| FQ4QJ5301D79G5 | 291 | 5.00E-25 | 58/88 (65%) | Gibberella zeae PH-1 | hypothetical protein FG05100.1 |
| FQ4QJ5301AXX20 | 135 | 2.00E-10 | 25/49 (51%) | Gibberella zeae PH-1 | hypothetical protein FG05108.1 |
| FQ4QJ5301B3RR3 | 132 | 1.00E-06 | 27/37 (72%) | Gibberella zeae PH-1 | hypothetical protein FG05131.1 |
| FQ92HJ001AWILD | 111 | 3.00E-04 | 22/23 (95%) | Gibberella zeae PH-1 | hypothetical protein FG05134.1 |
| FQ4QJ5301B5O25 | 257 | 4.00E-21 | 48/78 (61%) | Gibberella zeae PH-1 | hypothetical protein FG05143.1 |
| FQ4QJ5301ARVHP | 112 | 3.00E-04 | 23/34 (67%) | Gibberella zeae PH-1 | hypothetical protein FG05148.1 |
| FQ4QJ5301EOZ94 | 156 | 2.00E-09 | 28/62 (45%) | Gibberella zeae PH-1 | hypothetical protein FG05161.1 |
| FQ92HJ001EGFZU | 147 | 2.00E-08 | 30/54 (55%) | Gibberella zeae PH-1 | hypothetical protein FG05171.1 |
| Contig3476 | 170 | 3.00E-41 | 79/84 (94%) | Gibberella zeae PH-1 | hypothetical protein FG05174.1 |
| FQ4QJ5301ASYQT | 112 | 3.00E-04 | 22/33 (66%) | Gibberella zeae PH-1 | hypothetical protein FG05190.1 |
| FQ92HJ001EWUIX | 346 | 2.00E-31 | 63/78 (80%) | Gibberella zeae PH-1 | hypothetical protein FG05191.1 |
| FQ92HJ001DJ1CQ | 331 | 1.00E-29 | 61/76 (80%) | Gibberella zeae PH-1 | hypothetical protein FG05194.1 |
| FQ92HJ001BCQ6R | 299 | 5.00E-26 | 59/75 (78%) | Gibberella zeae PH-1 | hypothetical protein FG05202.1 |
| Contig1706 | 113 | 5.00E-24 | 47/67 (70%) | Gibberella zeae PH-1 | hypothetical protein FG05218.1 |
| FQ4QJ5301EN0RF | 378 | 4.00E-35 | 75/78 (96%) | Gibberella zeae PH-1 | hypothetical protein FG05222.1 |
| Contig3267 | 218 | 8.00E-56 | 93/134 (69%) | Gibberella zeae PH-1 | hypothetical protein FG05243.1 |
| FQ4QJ5301B3G4P | 93 | 4.10E-02 | 20/60 (33%) | Gibberella zeae PH-1 | hypothetical protein FG05244.1 |
| FQ4QJ5301CJVJE | 75 | 5.10E+00 | 13/24 (54%) | Gibberella zeae PH-1 | hypothetical protein FG05244.1 |
| Contig3574 | 153 | 4.00E-36 | 72/78 (92%) | Gibberella zeae PH-1 | hypothetical protein FG05250.1 |
| Contig13927 | 62 | 1.00E-08 | 31/62 (50%) | Gibberella zeae PH-1 | hypothetical protein FG05271.1 |
| FQ92HJ001E3P0K | 220 | 8.00E-17 | 40/82 (48%) | Gibberella zeae PH-1 | hypothetical protein FG05287.1 |
| FQ4QJ5301ASMC9 | 112 | 3.00E-04 | 23/23 (100%) | Gibberella zeae PH-1 | hypothetical protein FG05293.1 |
| FQ4QJ5301C7703 | 281 | 6.00E-24 | 49/66 (74%) | Gibberella zeae PH-1 | hypothetical protein FG05297.1 |
| Contig3215 | 111 | 1.00E-23 | 49/59 (83%) | Gibberella zeae PH-1 | hypothetical protein FG05298.1 |
| FQ92HJ001EFP4N | 185 | 9.00E-13 | 42/78 (53%) | Gibberella zeae PH-1 | hypothetical protein FG05306.1 |
| FQ4QJ5301AV24Y | 193 | 1.00E-13 | 46/77 (59%) | Gibberella zeae PH-1 | hypothetical protein FG05315.1 |
| FQ4QJ5301BQWMG | 116 | 9.00E-05 | 25/42 (59%) | Gibberella zeae PH-1 | hypothetical protein FG05328.1 |
| FQ4QJ5301A4TTU | 238 | 4.00E-21 | 40/58 (68%) | Gibberella zeae PH-1 | hypothetical protein FG05344.1 |
| FQ92HJ001BZLZP | 228 | 9.00E-18 | 46/78 (58%) | Gibberella zeae PH-1 | hypothetical protein FG05352.1 |
| FQ92HJ001DEMOR | 179 | 2.00E-18 | 36/41 (87%) | Gibberella zeae PH-1 | hypothetical protein FG05352.1 |
| FQ92HJ001E2545 | 165 | 2.00E-10 | 31/43 (72%) | Gibberella zeae PH-1 | hypothetical protein FG05361.1 |
| Contig5965 | 105 | 1.00E-21 | 50/54 (92%) | Gibberella zeae PH-1 | hypothetical protein FG05365.1 |
| Contig16285 | 44.7 | 2.00E-03 | 20/30 (66%) | Gibberella zeae PH-1 | hypothetical protein FG05388.1 |
| FQ4QJ5301BIAJO | 194 | 1.00E-22 | 33/45 (73%) | Gibberella zeae PH-1 | hypothetical protein FG05403.1 |
| FQ92HJ001C1L93 | 150 | 1.00E-08 | 26/40 (65%) | Gibberella zeae PH-1 | hypothetical protein FG05412.1 |
| FQ4QJ5301BDFAP | 231 | 4.00E-18 | 44/81 (54%) | Gibberella zeae PH-1 | hypothetical protein FG05413.1 |
| FQ92HJ001CSD5G | 91 | 7.00E-02 | 14/22 (63%) | Gibberella zeae PH-1 | hypothetical protein FG05417.1 |
| FQ4QJ5301ANEIE | 89 | 1.20E-01 | 16/24 (66%) | Gibberella zeae PH-1 | hypothetical protein FG05419.1 |
| FQ4QJ5301DGEFO | 242 | 2.00E-19 | 47/58 (81%) | Gibberella zeae PH-1 | hypothetical protein FG05423.1 |
| FQ4QJ5301CCJ5X | 281 | 7.00E-24 | 56/61 (91%) | Gibberella zeae PH-1 | hypothetical protein FG05428.1 |
| FQ92HJ001ETCZ6 | 157 | 3.00E-12 | 29/34 (85%) | Gibberella zeae PH-1 | hypothetical protein FG05432.1 |
| FQ92HJ001CX6X7 | 323 | 9.00E-29 | 61/83 (73%) | Gibberella zeae PH-1 | hypothetical protein FG05451.1 |
| FQ4QJ5301C8B21 | 362 | 3.00E-33 | 67/80 (83%) | Gibberella zeae PH-1 | hypothetical protein FG05454.1 |
| FQ92HJ001E0ASJ | 202 | 9.00E-15 | 36/76 (47%) | Gibberella zeae PH-1 | hypothetical protein FG05462.1 |
| FQ4QJ5301E3NOW | 162 | 4.00E-10 | 33/35 (94%) | Gibberella zeae PH-1 | hypothetical protein FG05465.1 |
| FQ92HJ001DMCY6 | 234 | 2.00E-18 | 45/63 (71%) | Gibberella zeae PH-1 | hypothetical protein FG05465.1 |
| FQ92HJ001CSC4H | 153 | 5.00E-09 | 28/36 (77%) | Gibberella zeae PH-1 | hypothetical protein FG05465.1 |
| FQ92HJ001C7GEF | 246 | 7.00E-20 | 49/66 (74%) | Gibberella zeae PH-1 | hypothetical protein FG05469.1 |
| FQ4QJ5301APD24 | 282 | 6.00E-29 | 56/64 (87%) | Gibberella zeae PH-1 | hypothetical protein FG05484.1 |
| FQ4QJ5301D81X2 | 192 | 3.00E-23 | 30/51 (58%) | Gibberella zeae PH-1 | hypothetical protein FG05495.1 |
| FQ92HJ001CKUKV | 149 | 1.00E-08 | 25/34 (73%) | Gibberella zeae PH-1 | hypothetical protein FG05495.1 |
| FQ4QJ5301CD4DW | 234 | 2.00E-18 | 48/65 (73%) | Gibberella zeae PH-1 | hypothetical protein FG05498.1 |
| FQ92HJ001CW5YS | 323 | 9.00E-29 | 60/78 (76%) | Gibberella zeae PH-1 | hypothetical protein FG05506.1 |
| FQ4QJ5301CU7PB | 212 | 7.00E-16 | 39/58 (67%) | Gibberella zeae PH-1 | hypothetical protein FG05531.1 |
| FQ92HJ001DZH8K | 155 | 3.00E-09 | 38/80 (47%) | Gibberella zeae PH-1 | hypothetical protein FG05539.1 |
| FQ4QJ5301CH4AF | 288 | 1.00E-24 | 51/62 (82%) | Gibberella zeae PH-1 | hypothetical protein FG05541.1 |
| Contig10697 | 115 | 1.00E-24 | 50/66 (75%) | Gibberella zeae PH-1 | hypothetical protein FG05543.1 |
| FQ4QJ5301EQ9PV | 159 | 9.00E-10 | 35/75 (46%) | Gibberella zeae PH-1 | hypothetical protein FG05544.1 |
| FQ4QJ5301ELHES | 291 | 5.00E-25 | 56/62 (90%) | Gibberella zeae PH-1 | hypothetical protein FG05547.1 |
| Contig6272 | 115 | 1.00E-24 | 56/71 (78%) | Gibberella zeae PH-1 | hypothetical protein FG05561.1 |
| FQ4QJ5301AFD5A | 349 | 8.00E-32 | 64/81 (79%) | Gibberella zeae PH-1 | hypothetical protein FG05562.1 |
| FQ4QJ5301EHYQG | 209 | 1.00E-15 | 43/83 (51%) | Gibberella zeae PH-1 | hypothetical protein FG05571.1 |
| FQ4QJ5301CQGYS | 236 | 1.00E-18 | 44/74 (59%) | Gibberella zeae PH-1 | hypothetical protein FG05572.1 |
| FQ92HJ001EKXFI | 92 | 4.50E-02 | 17/21 (80%) | Gibberella zeae PH-1 | hypothetical protein FG05586.1 |
| FQ4QJ5301AR0RC | 310 | 3.00E-27 | 56/81 (69%) | Gibberella zeae PH-1 | hypothetical protein FG05593.1 |
| FQ4QJ5301DYYN3 | 155 | 3.00E-09 | 30/58 (51%) | Gibberella zeae PH-1 | hypothetical protein FG05599.1 |
| FQ92HJ001E4UTS | 107 | 6.00E-11 | 20/38 (52%) | Gibberella zeae PH-1 | hypothetical protein FG05599.1 |
| Contig14874 | 195 | 1.00E-48 | 86/116 (74%) | Gibberella zeae PH-1 | hypothetical protein FG05602.1 |
| FQ92HJ001ANKRL | 236 | 1.00E-18 | 47/51 (92%) | Gibberella zeae PH-1 | hypothetical protein FG05602.1 |
| FQ4QJ5301A69O0 | 85 | 3.50E-01 | 16/16 (100%) | Gibberella zeae PH-1 | hypothetical protein FG05616.1 |
| FQ4QJ5301D85M5 | 351 | 5.00E-32 | 68/72 (94%) | Gibberella zeae PH-1 | hypothetical protein FG05619.1 |
| FQ4QJ5301B4CYW | 373 | 1.00E-34 | 69/78 (88%) | Gibberella zeae PH-1 | hypothetical protein FG05631.1 |
| FQ4QJ5301EQY5M | 196 | 5.00E-14 | 42/69 (60%) | Gibberella zeae PH-1 | hypothetical protein FG05633.1 |
| FQ92HJ001DUVBW | 78 | 2.30E+00 | 20/52 (38%) | Gibberella zeae PH-1 | hypothetical protein FG05639.1 |
| FQ92HJ001ASVRB | 113 | 2.00E-04 | 21/44 (47%) | Gibberella zeae PH-1 | hypothetical protein FG05655.1 |
| FQ4QJ5301EFGYJ | 222 | 5.00E-17 | 39/67 (58%) | Gibberella zeae PH-1 | hypothetical protein FG05665.1 |
| FQ4QJ5301DYJMH | 225 | 2.00E-17 | 44/76 (57%) | Gibberella zeae PH-1 | hypothetical protein FG05671.1 |
| FQ4QJ5301D0IWN | 140 | 1.00E-07 | 23/34 (67%) | Gibberella zeae PH-1 | hypothetical protein FG05679.1 |
| FQ4QJ5301AGLG5 | 334 | 5.00E-30 | 63/74 (85%) | Gibberella zeae PH-1 | hypothetical protein FG05696.1 |
| FQ92HJ001AH7CU | 130 | 2.00E-06 | 27/53 (50%) | Gibberella zeae PH-1 | hypothetical protein FG05696.1 |
| FQ92HJ001CKTD8 | 74 | 6.50E+00 | 16/43 (37%) | Gibberella zeae PH-1 | hypothetical protein FG05716.1 |
| FQ92HJ001CGW3Q | 162 | 4.00E-10 | 28/38 (73%) | Gibberella zeae PH-1 | hypothetical protein FG05723.1 |
| Contig2781 | 68.6 | 1.00E-10 | 31/43 (72%) | Gibberella zeae PH-1 | hypothetical protein FG05727.1 |
| FQ4QJ5301EUH7Q | 159 | 8.00E-17 | 33/47 (70%) | Gibberella zeae PH-1 | hypothetical protein FG05736.1 |
| FQ4QJ5301ENXT1 | 98 | 1.10E-02 | 19/57 (33%) | Gibberella zeae PH-1 | hypothetical protein FG05736.1 |
| Contig8248 | 53.5 | 5.00E-06 | 27/48 (56%) | Gibberella zeae PH-1 | hypothetical protein FG05750.1 |
| FQ4QJ5301EF27P | 165 | 2.00E-10 | 30/35 (85%) | Gibberella zeae PH-1 | hypothetical protein FG05750.1 |
| Contig1381 | 112 | 6.00E-24 | 53/82 (64%) | Gibberella zeae PH-1 | hypothetical protein FG05757.1 |
| Contig5974 | 100 | 4.00E-20 | 46/86 (53%) | Gibberella zeae PH-1 | hypothetical protein FG05757.1 |
| Contig4206 | 43.5 | 5.00E-03 | 24/80 (30%) | Gibberella zeae PH-1 | hypothetical protein FG05763.1 |
| FQ4QJ5301ETGAI | 75 | 2.30E-01 | 12/17 (70%) | Gibberella zeae PH-1 | hypothetical protein FG05766.1 |
| FQ4QJ5301DGY03 | 131 | 2.00E-06 | 21/36 (58%) | Gibberella zeae PH-1 | hypothetical protein FG05766.1 |
| FQ92HJ001B9ZGG | 196 | 5.00E-14 | 40/74 (54%) | Gibberella zeae PH-1 | hypothetical protein FG05766.1 |
| FQ4QJ5301EIEMD | 378 | 4.00E-35 | 67/87 (77%) | Gibberella zeae PH-1 | hypothetical protein FG05775.1 |
| Contig14755 | 62.8 | 8.00E-09 | 36/84 (42%) | Gibberella zeae PH-1 | hypothetical protein FG05780.1 |
| FQ92HJ001A55UY | 116 | 9.00E-05 | 24/44 (54%) | Gibberella zeae PH-1 | hypothetical protein FG05852.1 |
| FQ4QJ5301BYA7A | 113 | 2.00E-04 | 19/29 (65%) | Gibberella zeae PH-1 | hypothetical protein FG05862.1 |
| Contig3354 | 110 | 2.00E-23 | 56/77 (72%) | Gibberella zeae PH-1 | hypothetical protein FG05884.1 |
| FQ4QJ5301DHXBS | 180 | 3.00E-12 | 39/75 (52%) | Gibberella zeae PH-1 | hypothetical protein FG05909.1 |
| Contig14592 | 95.9 | 8.00E-19 | 45/77 (58%) | Gibberella zeae PH-1 | hypothetical protein FG05910.1 |
| Contig13899 | 104 | 2.00E-21 | 52/74 (70%) | Gibberella zeae PH-1 | hypothetical protein FG05916.1 |
| Contig2893 | 84.7 | 2.00E-15 | 41/70 (58%) | Gibberella zeae PH-1 | hypothetical protein FG05932.1 |
| FQ4QJ5301CAFC4 | 112 | 3.00E-04 | 21/29 (72%) | Gibberella zeae PH-1 | hypothetical protein FG05932.1 |
| Contig10689 | 60.8 | 3.00E-08 | 25/35 (71%) | Gibberella zeae PH-1 | hypothetical protein FG05946.1 |
| Contig16205 | 133 | 5.00E-30 | 61/87 (70%) | Gibberella zeae PH-1 | hypothetical protein FG05954.1 |
| FQ4QJ5301BURMC | 200 | 2.00E-14 | 38/62 (61%) | Gibberella zeae PH-1 | hypothetical protein FG05954.1 |
| Contig1882 | 72.8 | 7.00E-12 | 32/49 (65%) | Gibberella zeae PH-1 | hypothetical protein FG05955.1 |
| FQ4QJ5301BWZR2 | 184 | 1.00E-12 | 33/52 (63%) | Gibberella zeae PH-1 | hypothetical protein FG05955.1 |
| FQ92HJ001C0314 | 279 | 1.00E-23 | 46/71 (64%) | Gibberella zeae PH-1 | hypothetical protein FG05955.1 |
| FQ4QJ5301AZ44S | 386 | 4.00E-36 | 75/86 (87%) | Gibberella zeae PH-1 | hypothetical protein FG05998.1 |
| Contig15810 | 231 | 1.00E-59 | 113/123 (91%) | Gibberella zeae PH-1 | hypothetical protein FG05999.1 |
| Contig3843 | 85.5 | 1.00E-15 | 49/107 (45%) | Gibberella zeae PH-1 | hypothetical protein FG06005.1 |
| Contig5231 | 155 | 9.00E-37 | 76/83 (91%) | Gibberella zeae PH-1 | hypothetical protein FG06020.1 |
| FQ4QJ5301AQSTX | 256 | 5.00E-21 | 50/63 (79%) | Gibberella zeae PH-1 | hypothetical protein FG06027.1 |
| Contig10958 | 86.3 | 7.00E-16 | 39/59 (66%) | Gibberella zeae PH-1 | hypothetical protein FG06029.1 |
| Contig9898 | 40.4 | 4.00E-02 | 22/60 (36%) | Gibberella zeae PH-1 | hypothetical protein FG06034.1 |
| FQ4QJ5301B3XE2 | 117 | 7.00E-05 | 30/54 (55%) | Gibberella zeae PH-1 | hypothetical protein FG06040.1 |
| FQ92HJ001BQGJD | 306 | 8.00E-27 | 59/78 (75%) | Gibberella zeae PH-1 | hypothetical protein FG06051.1 |
| Contig1102 | 150 | 2.00E-35 | 68/82 (82%) | Gibberella zeae PH-1 | hypothetical protein FG06052.1 |
| FQ4QJ5301DHYDE | 186 | 3.00E-14 | 31/43 (72%) | Gibberella zeae PH-1 | hypothetical protein FG06052.1 |
| FQ4QJ5301EI6OQ | 209 | 1.00E-15 | 38/58 (65%) | Gibberella zeae PH-1 | hypothetical protein FG06061.1 |
| Contig13280 | 49.7 | 7.00E-05 | 24/49 (48%) | Gibberella zeae PH-1 | hypothetical protein FG06064.1 |
| FQ92HJ001C9GPT | 232 | 2.00E-23 | 45/59 (76%) | Gibberella zeae PH-1 | hypothetical protein FG06068.1 |
| Contig11551 | 99.4 | 7.00E-20 | 47/66 (71%) | Gibberella zeae PH-1 | hypothetical protein FG06069.1 |
| FQ4QJ5301COUBQ | 268 | 2.00E-22 | 49/78 (62%) | Gibberella zeae PH-1 | hypothetical protein FG06069.1 |
| FQ4QJ5301EBPIR | 158 | 1.00E-09 | 28/57 (49%) | Gibberella zeae PH-1 | hypothetical protein FG06097.1 |
| Contig6341 | 220 | 2.00E-56 | 101/130 (77%) | Gibberella zeae PH-1 | hypothetical protein FG06098.1 |
| Contig11110 | 95.9 | 8.00E-19 | 48/76 (63%) | Gibberella zeae PH-1 | hypothetical protein FG06102.1 |
| FQ92HJ001EZFGS | 194 | 8.00E-14 | 38/39 (97%) | Gibberella zeae PH-1 | hypothetical protein FG06112.1 |
| FQ4QJ5301EKAXZ | 350 | 6.00E-32 | 62/93 (66%) | Gibberella zeae PH-1 | hypothetical protein FG06115.1 |
| Contig7234 | 52.4 | 1.00E-05 | 37/99 (37%) | Gibberella zeae PH-1 | hypothetical protein FG06157.1 |
| FQ4QJ5301DRCR7 | 339 | 1.00E-30 | 64/69 (92%) | Gibberella zeae PH-1 | hypothetical protein FG06159.1 |
| FQ4QJ5301C3NYH | 81 | 9.90E-01 | 24/57 (42%) | Gibberella zeae PH-1 | hypothetical protein FG06161.1 |
| Contig7053 | 80.1 | 5.00E-14 | 39/48 (81%) | Gibberella zeae PH-1 | hypothetical protein FG06166.1 |
| FQ4QJ5301DFBEL | 286 | 2.00E-24 | 56/65 (86%) | Gibberella zeae PH-1 | hypothetical protein FG06166.1 |
| FQ4QJ5301AFOVG | 81 | 1.00E+00 | 21/38 (55%) | Gibberella zeae PH-1 | hypothetical protein FG06167.1 |
| Contig1403 | 147 | 2.00E-34 | 72/84 (85%) | Gibberella zeae PH-1 | hypothetical protein FG06183.1 |
| FQ4QJ5301CQW8O | 394 | 5.00E-37 | 75/84 (89%) | Gibberella zeae PH-1 | hypothetical protein FG06184.1 |
| FQ4QJ5301E189V | 436 | 7.00E-42 | 74/82 (90%) | Gibberella zeae PH-1 | hypothetical protein FG06185.1 |
| FQ4QJ5301AFV70 | 262 | 1.00E-21 | 54/76 (71%) | Gibberella zeae PH-1 | hypothetical protein FG06186.1 |
| FQ4QJ5301EVEZ0 | 344 | 3.00E-31 | 65/86 (75%) | Gibberella zeae PH-1 | hypothetical protein FG06193.1 |
| Contig4005 | 202 | 8.00E-51 | 92/109 (84%) | Gibberella zeae PH-1 | hypothetical protein FG06219.1 |
| FQ4QJ5301D8IVO | 252 | 2.00E-20 | 47/71 (66%) | Gibberella zeae PH-1 | hypothetical protein FG06219.1 |
| Contig16420 | 96.7 | 5.00E-19 | 48/105 (45%) | Gibberella zeae PH-1 | hypothetical protein FG06244.1 |
| FQ4QJ5301DVJAZ | 99 | 8.00E-03 | 17/22 (77%) | Gibberella zeae PH-1 | hypothetical protein FG06244.1 |
| Contig12301 | 40 | 2.00E-06 | 16/28 (57%) | Gibberella zeae PH-1 | hypothetical protein FG06250.1 |
| FQ4QJ5301BIJYI | 241 | 3.00E-19 | 42/73 (57%) | Gibberella zeae PH-1 | hypothetical protein FG06264.1 |
| FQ92HJ001C1FS5 | 157 | 2.00E-09 | 28/32 (87%) | Gibberella zeae PH-1 | hypothetical protein FG06265.1 |
| Contig10103 | 40 | 5.40E-02 | 18/28 (64%) | Gibberella zeae PH-1 | hypothetical protein FG06267.1 |
| FQ4QJ5301E03XU | 174 | 2.00E-11 | 35/70 (50%) | Gibberella zeae PH-1 | hypothetical protein FG06269.1 |
| FQ92HJ001AWU87 | 224 | 3.00E-17 | 43/60 (71%) | Gibberella zeae PH-1 | hypothetical protein FG06269.1 |
| FQ4QJ5301E1GDA | 228 | 9.00E-18 | 44/78 (56%) | Gibberella zeae PH-1 | hypothetical protein FG06272.1 |
| FQ92HJ001A98ED | 308 | 5.00E-27 | 57/77 (74%) | Gibberella zeae PH-1 | hypothetical protein FG06272.1 |
| FQ92HJ001CJ47B | 129 | 3.00E-06 | 29/55 (52%) | Gibberella zeae PH-1 | hypothetical protein FG06276.1 |
| FQ4QJ5301C3GLN | 209 | 1.00E-15 | 38/50 (76%) | Gibberella zeae PH-1 | hypothetical protein FG06277.1 |
| FQ4QJ5301B99QA | 362 | 3.00E-33 | 68/83 (81%) | Gibberella zeae PH-1 | hypothetical protein FG06277.1 |
| FQ4QJ5301CY3K3 | 100 | 6.00E-03 | 28/74 (37%) | Gibberella zeae PH-1 | hypothetical protein FG06288.1 |
| FQ4QJ5301CU4YN | 187 | 5.00E-13 | 40/59 (67%) | Gibberella zeae PH-1 | hypothetical protein FG06291.1 |
| FQ4QJ5301DNUCD | 74 | 6.50E+00 | 13/22 (59%) | Gibberella zeae PH-1 | hypothetical protein FG06299.1 |
| FQ4QJ5301EMNMB | 195 | 5.00E-20 | 38/47 (80%) | Gibberella zeae PH-1 | hypothetical protein FG06302.1 |
| FQ4QJ5301EM8IV | 336 | 3.00E-30 | 63/77 (81%) | Gibberella zeae PH-1 | hypothetical protein FG06313.1 |
| FQ92HJ001EAF4N | 190 | 4.00E-17 | 36/56 (64%) | Gibberella zeae PH-1 | hypothetical protein FG06314.1 |
| FQ92HJ001DFBRC | 148 | 2.00E-08 | 29/48 (60%) | Gibberella zeae PH-1 | hypothetical protein FG06320.1 |
| FQ92HJ001A3SEL | 237 | 8.00E-19 | 52/91 (57%) | Gibberella zeae PH-1 | hypothetical protein FG06325.1 |
| Contig4095 | 91.3 | 2.00E-17 | 42/49 (85%) | Gibberella zeae PH-1 | hypothetical protein FG06331.1 |
| Contig12411 | 71.6 | 2.00E-11 | 34/49 (69%) | Gibberella zeae PH-1 | hypothetical protein FG06336.1 |
| FQ4QJ5301EXWFS | 343 | 4.00E-31 | 64/79 (81%) | Gibberella zeae PH-1 | hypothetical protein FG06352.1 |
| FQ4QJ5301CHKBJ | 285 | 3.00E-30 | 52/64 (81%) | Gibberella zeae PH-1 | hypothetical protein FG06353.1 |
| FQ4QJ5301A0QU8 | 294 | 2.00E-25 | 52/68 (76%) | Gibberella zeae PH-1 | hypothetical protein FG06359.1 |
| FQ4QJ5301EVLTF | 241 | 3.00E-19 | 45/76 (59%) | Gibberella zeae PH-1 | hypothetical protein FG06368.1 |
| FQ92HJ001EGOGU | 387 | 3.00E-36 | 73/77 (94%) | Gibberella zeae PH-1 | hypothetical protein FG06384.1 |
| Contig4128 | 38.5 | 6.60E-02 | 24/67 (35%) | Gibberella zeae PH-1 | hypothetical protein FG06390.1 |
| FQ4QJ5301AFCS0 | 170 | 5.00E-11 | 48/96 (50%) | Gibberella zeae PH-1 | hypothetical protein FG06390.1 |
| FQ4QJ5301B2U1F | 251 | 1.00E-22 | 48/61 (78%) | Gibberella zeae PH-1 | hypothetical protein FG06392.1 |
| FQ92HJ001DNG71 | 206 | 3.00E-15 | 42/72 (58%) | Gibberella zeae PH-1 | hypothetical protein FG06406.1 |
| FQ4QJ5301BL5GB | 254 | 9.00E-21 | 51/83 (61%) | Gibberella zeae PH-1 | hypothetical protein FG06427.1 |
| FQ92HJ001CJ3RO | 341 | 7.00E-31 | 58/75 (77%) | Gibberella zeae PH-1 | hypothetical protein FG06447.1 |
| FQ4QJ5301EWAK1 | 136 | 4.00E-07 | 23/39 (58%) | Gibberella zeae PH-1 | hypothetical protein FG06448.1 |
| Contig2912 | 93.2 | 5.00E-18 | 42/89 (47%) | Gibberella zeae PH-1 | hypothetical protein FG06508.1 |
| FQ4QJ5301EEN7X | 286 | 2.00E-24 | 53/58 (91%) | Gibberella zeae PH-1 | hypothetical protein FG06532.1 |
| FQ92HJ001DSB88 | 87 | 2.00E-01 | 15/16 (93%) | Gibberella zeae PH-1 | hypothetical protein FG06532.1 |
| FQ4QJ5301ESYHE | 114 | 2.00E-06 | 22/44 (50%) | Gibberella zeae PH-1 | hypothetical protein FG06539.1 |
| Contig16588 | 78.6 | 1.00E-13 | 33/56 (58%) | Gibberella zeae PH-1 | hypothetical protein FG06543.1 |
| FQ4QJ5301A8FLM | 91 | 7.00E-02 | 20/39 (51%) | Gibberella zeae PH-1 | hypothetical protein FG06580.1 |
| FQ92HJ001AVAQY | 145 | 4.00E-08 | 25/45 (55%) | Gibberella zeae PH-1 | hypothetical protein FG06586.1 |
| Contig6653 | 229 | 5.00E-59 | 108/139 (77%) | Gibberella zeae PH-1 | hypothetical protein FG06616.1 |
| Contig14436 | 134 | 2.00E-30 | 59/87 (67%) | Gibberella zeae PH-1 | hypothetical protein FG06616.1 |
| Contig7942 | 55.5 | 1.00E-06 | 30/63 (47%) | Gibberella zeae PH-1 | hypothetical protein FG06636.1 |
| Contig16219 | 168 | 1.00E-40 | 79/118 (66%) | Gibberella zeae PH-1 | hypothetical protein FG06636.1 |
| FQ4QJ5301D1E1X | 144 | 2.00E-16 | 28/39 (71%) | Gibberella zeae PH-1 | hypothetical protein FG06636.1 |
| FQ4QJ5301C78P8 | 100 | 6.00E-03 | 28/54 (51%) | Gibberella zeae PH-1 | hypothetical protein FG06666.1 |
| FQ4QJ5301BV09L | 277 | 1.00E-23 | 50/72 (69%) | Gibberella zeae PH-1 | hypothetical protein FG06669.1 |
| FQ4QJ5301AT4P1 | 312 | 2.00E-27 | 60/73 (82%) | Gibberella zeae PH-1 | hypothetical protein FG06678.1 |
| FQ4QJ5301BDQS5 | 94 | 2.90E-02 | 19/23 (82%) | Gibberella zeae PH-1 | hypothetical protein FG06688.1 |
| FQ92HJ001DFKVT | 271 | 9.00E-23 | 56/74 (75%) | Gibberella zeae PH-1 | hypothetical protein FG06697.1 |
| Contig4458 | 87.4 | 3.00E-16 | 43/60 (71%) | Gibberella zeae PH-1 | hypothetical protein FG06702.1 |
| FQ4QJ5301BTFH4 | 337 | 2.00E-30 | 62/81 (76%) | Gibberella zeae PH-1 | hypothetical protein FG06702.1 |
| Contig11921 | 98.2 | 2.00E-19 | 53/93 (56%) | Gibberella zeae PH-1 | hypothetical protein FG06706.1 |
| FQ4QJ5301ETSKW | 278 | 1.00E-23 | 51/73 (69%) | Gibberella zeae PH-1 | hypothetical protein FG06717.1 |
| FQ4QJ5301ENTH5 | 289 | 8.00E-25 | 56/80 (70%) | Gibberella zeae PH-1 | hypothetical protein FG06721.1 |
| FQ92HJ001DJPG9 | 189 | 3.00E-13 | 36/51 (70%) | Gibberella zeae PH-1 | hypothetical protein FG06731.1 |
| FQ4QJ5301B8RY2 | 196 | 5.00E-14 | 42/74 (56%) | Gibberella zeae PH-1 | hypothetical protein FG06754.1 |
| FQ4QJ5301DUWDL | 159 | 5.00E-21 | 32/37 (86%) | Gibberella zeae PH-1 | hypothetical protein FG06759.1 |
| FQ4QJ5301BY0CY | 369 | 4.00E-34 | 71/74 (95%) | Gibberella zeae PH-1 | hypothetical protein FG06767.1 |
| FQ4QJ5301BXI8L | 178 | 6.00E-12 | 33/52 (63%) | Gibberella zeae PH-1 | hypothetical protein FG06775.1 |
| FQ4QJ5301DKQPT | 234 | 2.00E-18 | 45/78 (57%) | Gibberella zeae PH-1 | hypothetical protein FG06776.1 |
| FQ4QJ5301B1IK1 | 321 | 1.00E-28 | 59/83 (71%) | Gibberella zeae PH-1 | hypothetical protein FG06786.1 |
| Contig13383 | 80.9 | 3.00E-14 | 37/56 (66%) | Gibberella zeae PH-1 | hypothetical protein FG06788.1 |
| FQ92HJ001BXO7G | 110 | 4.00E-04 | 30/69 (43%) | Gibberella zeae PH-1 | hypothetical protein FG06807.1 |
| FQ4QJ5301DK0I6 | 200 | 2.00E-14 | 43/87 (49%) | Gibberella zeae PH-1 | hypothetical protein FG06809.1 |
| FQ4QJ5301ARFH3 | 235 | 1.00E-18 | 46/72 (63%) | Gibberella zeae PH-1 | hypothetical protein FG06813.1 |
| FQ92HJ001A33LK | 108 | 7.00E-04 | 30/83 (36%) | Gibberella zeae PH-1 | hypothetical protein FG06814.1 |
| FQ4QJ5301EC1UC | 383 | 9.00E-36 | 75/83 (90%) | Gibberella zeae PH-1 | hypothetical protein FG06816.1 |
| Contig11002 | 34.3 | 2.90E+00 | 20/47 (42%) | Gibberella zeae PH-1 | hypothetical protein FG06825.1 |
| FQ4QJ5301BF7MG | 139 | 3.00E-18 | 26/30 (86%) | Gibberella zeae PH-1 | hypothetical protein FG06825.1 |
| FQ92HJ001CWC5I | 313 | 1.00E-27 | 57/62 (91%) | Gibberella zeae PH-1 | hypothetical protein FG06825.1 |
| Contig6802 | 112 | 1.00E-23 | 53/69 (76%) | Gibberella zeae PH-1 | hypothetical protein FG06848.1 |
| Contig1689 | 128 | 1.00E-28 | 61/80 (76%) | Gibberella zeae PH-1 | hypothetical protein FG06875.1 |
| Contig8936 | 145 | 9.00E-34 | 71/79 (89%) | Gibberella zeae PH-1 | hypothetical protein FG06875.1 |
| FQ4QJ5301DRQEU | 151 | 8.00E-09 | 29/33 (87%) | Gibberella zeae PH-1 | hypothetical protein FG06876.1 |
| FQ92HJ001BP4UB | 229 | 7.00E-18 | 43/77 (55%) | Gibberella zeae PH-1 | hypothetical protein FG06882.1 |
| FQ4QJ5301EGZ14 | 231 | 4.00E-18 | 41/75 (54%) | Gibberella zeae PH-1 | hypothetical protein FG06904.1 |
| Contig12992 | 165 | 1.00E-39 | 77/96 (80%) | Gibberella zeae PH-1 | hypothetical protein FG06922.1 |
| Contig5195 | 153 | 4.00E-36 | 67/85 (78%) | Gibberella zeae PH-1 | hypothetical protein FG06923.1 |
| FQ4QJ5301EOWRB | 343 | 4.00E-31 | 64/80 (80%) | Gibberella zeae PH-1 | hypothetical protein FG06923.1 |
| Contig14634 | 322 | 1.00E-86 | 161/187 (86%) | Gibberella zeae PH-1 | hypothetical protein FG06924.1 |
| FQ4QJ5301CXKHC | 94 | 3.10E-02 | 19/26 (73%) | Gibberella zeae PH-1 | hypothetical protein FG06934.1 |
| Contig10533 | 177 | 2.00E-43 | 80/101 (79%) | Gibberella zeae PH-1 | hypothetical protein FG06936.1 |
| FQ4QJ5301D8RYJ | 193 | 1.00E-13 | 42/77 (54%) | Gibberella zeae PH-1 | hypothetical protein FG06942.1 |
| Contig12428 | 162 | 9.00E-39 | 78/82 (95%) | Gibberella zeae PH-1 | hypothetical protein FG06947.1 |
| FQ4QJ5301BMHZP | 109 | 6.00E-04 | 21/28 (75%) | Gibberella zeae PH-1 | hypothetical protein FG06951.1 |
| FQ4QJ5301BMROH | 191 | 2.00E-23 | 36/54 (66%) | Gibberella zeae PH-1 | hypothetical protein FG06951.1 |
| FQ92HJ001CNKI7 | 312 | 2.00E-27 | 55/69 (79%) | Gibberella zeae PH-1 | hypothetical protein FG06962.1 |
| FQ4QJ5301AMB85 | 292 | 3.00E-25 | 52/76 (68%) | Gibberella zeae PH-1 | hypothetical protein FG07006.1 |
| FQ4QJ5301BUK9M | 235 | 1.00E-18 | 44/73 (60%) | Gibberella zeae PH-1 | hypothetical protein FG07019.1 |
| FQ4QJ5301DURY8 | 143 | 7.00E-08 | 32/51 (62%) | Gibberella zeae PH-1 | hypothetical protein FG07021.1 |
| FQ4QJ5301BKCKD | 283 | 4.00E-24 | 56/76 (73%) | Gibberella zeae PH-1 | hypothetical protein FG07021.1 |
| FQ4QJ5301CBXIZ | 134 | 7.00E-07 | 26/55 (47%) | Gibberella zeae PH-1 | hypothetical protein FG07030.1 |
| Contig867 | 75.1 | 1.00E-12 | 36/53 (67%) | Gibberella zeae PH-1 | hypothetical protein FG07031.1 |
| Contig15204 | 111 | 1.00E-23 | 51/68 (75%) | Gibberella zeae PH-1 | hypothetical protein FG07031.1 |
| Contig10213 | 56.2 | 1.00E-15 | 22/34 (64%) | Gibberella zeae PH-1 | hypothetical protein FG07041.1 |
| FQ4QJ5301DPZUY | 77 | 2.90E+00 | 19/54 (35%) | Gibberella zeae PH-1 | hypothetical protein FG07061.1 |
| FQ4QJ5301EW8UY | 352 | 4.00E-32 | 68/74 (91%) | Gibberella zeae PH-1 | hypothetical protein FG07075.1 |
| FQ4QJ5301CNYGN | 114 | 1.00E-04 | 24/36 (66%) | Gibberella zeae PH-1 | hypothetical protein FG07086.1 |
| Contig8319 | 128 | 2.00E-28 | 58/74 (78%) | Gibberella zeae PH-1 | hypothetical protein FG07092.1 |
| Contig10467 | 131 | 1.00E-29 | 62/86 (72%) | Gibberella zeae PH-1 | hypothetical protein FG07113.1 |
| Contig15241 | 89.4 | 8.00E-17 | 40/54 (74%) | Gibberella zeae PH-1 | hypothetical protein FG07113.1 |
| FQ92HJ001D67WS | 77 | 2.90E+00 | 12/16 (75%) | Gibberella zeae PH-1 | hypothetical protein FG07131.1 |
| FQ92HJ001C0R57 | 261 | 1.00E-21 | 51/81 (62%) | Gibberella zeae PH-1 | hypothetical protein FG07134.1 |
| FQ92HJ001EFMV8 | 179 | 4.00E-12 | 35/44 (79%) | Gibberella zeae PH-1 | hypothetical protein FG07152.1 |
| FQ4QJ5301EHT02 | 302 | 2.00E-26 | 56/68 (82%) | Gibberella zeae PH-1 | hypothetical protein FG07154.1 |
| FQ4QJ5301D13GL | 137 | 3.00E-07 | 26/38 (68%) | Gibberella zeae PH-1 | hypothetical protein FG07169.1 |
| FQ4QJ5301DBOVA | 364 | 2.00E-33 | 76/78 (97%) | Gibberella zeae PH-1 | hypothetical protein FG07172.1 |
| Contig2542 | 126 | 6.00E-28 | 58/73 (79%) | Gibberella zeae PH-1 | hypothetical protein FG07182.1 |
| Contig3643 | 133 | 5.00E-30 | 68/90 (75%) | Gibberella zeae PH-1 | hypothetical protein FG07186.1 |
| Contig7376 | 74.3 | 3.00E-12 | 36/48 (75%) | Gibberella zeae PH-1 | hypothetical protein FG07186.1 |
| FQ92HJ001BO4FG | 360 | 5.00E-33 | 66/73 (90%) | Gibberella zeae PH-1 | hypothetical protein FG07199.1 |
| FQ4QJ5301AVH22 | 144 | 5.00E-08 | 28/37 (75%) | Gibberella zeae PH-1 | hypothetical protein FG07227.1 |
| FQ4QJ5301EQ1WD | 198 | 3.00E-14 | 32/48 (66%) | Gibberella zeae PH-1 | hypothetical protein FG07227.1 |
| FQ92HJ001EXGPY | 287 | 1.00E-24 | 53/78 (67%) | Gibberella zeae PH-1 | hypothetical protein FG07227.1 |
| Contig6987 | 162 | 9.00E-39 | 80/103 (77%) | Gibberella zeae PH-1 | hypothetical protein FG07228.1 |
| FQ4QJ5301C421A | 121 | 2.00E-05 | 26/51 (50%) | Gibberella zeae PH-1 | hypothetical protein FG07229.1 |
| FQ92HJ001D5C1K | 345 | 2.00E-31 | 64/83 (77%) | Gibberella zeae PH-1 | hypothetical protein FG07246.1 |
| Contig2578 | 51.6 | 2.00E-05 | 26/58 (44%) | Gibberella zeae PH-1 | hypothetical protein FG07250.1 |
| FQ92HJ001CTJ5D | 158 | 1.00E-09 | 33/36 (91%) | Gibberella zeae PH-1 | hypothetical protein FG07257.1 |
| FQ92HJ001CTHF2 | 330 | 1.00E-29 | 63/78 (80%) | Gibberella zeae PH-1 | hypothetical protein FG07262.1 |
| FQ92HJ001BYPSN | 216 | 2.00E-16 | 42/81 (51%) | Gibberella zeae PH-1 | hypothetical protein FG07262.1 |
| Contig5154 | 122 | 6.00E-27 | 59/79 (74%) | Gibberella zeae PH-1 | hypothetical protein FG07268.1 |
| Contig7700 | 45.1 | 2.00E-03 | 18/28 (64%) | Gibberella zeae PH-1 | hypothetical protein FG07269.1 |
| Contig12195 | 66.6 | 5.00E-10 | 29/46 (63%) | Gibberella zeae PH-1 | hypothetical protein FG07269.1 |
| Contig1026 | 70.9 | 3.00E-11 | 38/88 (43%) | Gibberella zeae PH-1 | hypothetical protein FG07279.1 |
| FQ4QJ5301ET13G | 96 | 1.90E-02 | 19/47 (40%) | Gibberella zeae PH-1 | hypothetical protein FG07286.1 |
| Contig3494 | 138 | 1.00E-31 | 61/76 (80%) | Gibberella zeae PH-1 | hypothetical protein FG07307.1 |
| FQ4QJ5301APVNP | 196 | 5.00E-14 | 38/80 (47%) | Gibberella zeae PH-1 | hypothetical protein FG07308.1 |
| FQ92HJ001D2KCF | 137 | 3.00E-07 | 26/54 (48%) | Gibberella zeae PH-1 | hypothetical protein FG07320.1 |
| FQ92HJ001DZ6C4 | 253 | 1.00E-20 | 54/78 (69%) | Gibberella zeae PH-1 | hypothetical protein FG07322.1 |
| FQ92HJ001E1OCG | 86 | 2.70E-01 | 19/43 (44%) | Gibberella zeae PH-1 | hypothetical protein FG07327.1 |
| FQ4QJ5301DT8JA | 258 | 3.00E-21 | 52/72 (72%) | Gibberella zeae PH-1 | hypothetical protein FG07328.1 |
| FQ4QJ5301C3V31 | 227 | 1.00E-17 | 37/57 (64%) | Gibberella zeae PH-1 | hypothetical protein FG07333.1 |
| FQ4QJ5301A584W | 215 | 3.00E-16 | 43/71 (60%) | Gibberella zeae PH-1 | hypothetical protein FG07343.1 |
| FQ4QJ5301CKPLG | 142 | 9.00E-08 | 26/28 (92%) | Gibberella zeae PH-1 | hypothetical protein FG07344.1 |
| FQ4QJ5301EWFA4 | 204 | 6.00E-15 | 34/46 (73%) | Gibberella zeae PH-1 | hypothetical protein FG07369.1 |
| FQ4QJ5301B3JDB | 269 | 2.00E-22 | 47/79 (59%) | Gibberella zeae PH-1 | hypothetical protein FG07374.1 |
| Contig14732 | 113 | 4.00E-24 | 53/88 (60%) | Gibberella zeae PH-1 | hypothetical protein FG07377.1 |
| FQ92HJ001DDN3Z | 226 | 2.00E-17 | 47/80 (58%) | Gibberella zeae PH-1 | hypothetical protein FG07377.1 |
| Contig3503 | 146 | 4.00E-34 | 72/109 (66%) | Gibberella zeae PH-1 | hypothetical protein FG07402.1 |
| FQ4QJ5301A0MUN | 110 | 4.00E-04 | 27/41 (65%) | Gibberella zeae PH-1 | hypothetical protein FG07407.1 |
| FQ92HJ001EY1EA | 166 | 1.00E-10 | 34/61 (55%) | Gibberella zeae PH-1 | hypothetical protein FG07417.1 |
| FQ92HJ001BU6LY | 153 | 5.00E-09 | 27/32 (84%) | Gibberella zeae PH-1 | hypothetical protein FG07419.1 |
| Contig13197 | 100 | 3.00E-20 | 48/64 (75%) | Gibberella zeae PH-1 | hypothetical protein FG07421.1 |
| FQ4QJ5301CWSZ1 | 134 | 7.00E-07 | 26/27 (96%) | Gibberella zeae PH-1 | hypothetical protein FG07421.1 |
| FQ4QJ5301BHD8I | 158 | 1.00E-10 | 30/57 (52%) | Gibberella zeae PH-1 | hypothetical protein FG07427.1 |
| Contig12826 | 173 | 3.00E-42 | 87/118 (73%) | Gibberella zeae PH-1 | hypothetical protein FG07438.1 |
| Contig12899 | 103 | 5.00E-21 | 57/103 (55%) | Gibberella zeae PH-1 | hypothetical protein FG07438.1 |
| FQ92HJ001EVBFA | 142 | 9.00E-08 | 29/60 (48%) | Gibberella zeae PH-1 | hypothetical protein FG07442.1 |
| FQ92HJ001EPHAK | 123 | 1.00E-05 | 22/33 (66%) | Gibberella zeae PH-1 | hypothetical protein FG07443.1 |
| Contig13118 | 60.1 | 5.00E-08 | 28/56 (50%) | Gibberella zeae PH-1 | hypothetical protein FG07450.1 |
| FQ4QJ5301BH8X9 | 141 | 3.00E-09 | 33/66 (50%) | Gibberella zeae PH-1 | hypothetical protein FG07452.1 |
| FQ4QJ5301DR2IL | 83 | 5.80E-01 | 24/76 (31%) | Gibberella zeae PH-1 | hypothetical protein FG07464.1 |
| FQ4QJ5301BL1G2 | 86 | 5.00E-02 | 15/16 (93%) | Gibberella zeae PH-1 | hypothetical protein FG07464.1 |
| FQ4QJ5301BIKF0 | 256 | 5.00E-21 | 49/82 (59%) | Gibberella zeae PH-1 | hypothetical protein FG07466.1 |
| FQ92HJ001DZQTM | 139 | 2.00E-07 | 32/71 (45%) | Gibberella zeae PH-1 | hypothetical protein FG07467.1 |
| FQ4QJ5301A0LB3 | 161 | 5.00E-10 | 29/31 (93%) | Gibberella zeae PH-1 | hypothetical protein FG07470.1 |
| Contig3094 | 128 | 1.00E-28 | 59/79 (74%) | Gibberella zeae PH-1 | hypothetical protein FG07473.1 |
| FQ4QJ5301BOU7P | 364 | 2.00E-33 | 70/81 (86%) | Gibberella zeae PH-1 | hypothetical protein FG07475.1 |
| FQ92HJ001ARZTE | 352 | 4.00E-32 | 62/71 (87%) | Gibberella zeae PH-1 | hypothetical protein FG07476.1 |
| Contig7085 | 298 | 1.00E-79 | 146/161 (90%) | Gibberella zeae PH-1 | hypothetical protein FG07480.1 |
| Contig12335 | 126 | 6.00E-28 | 62/67 (92%) | Gibberella zeae PH-1 | hypothetical protein FG07480.1 |
| Contig15140 | 34.3 | 3.00E+00 | 15/37 (40%) | Gibberella zeae PH-1 | hypothetical protein FG07494.1 |
| Contig14136 | 182 | 7.00E-45 | 82/114 (71%) | Gibberella zeae PH-1 | hypothetical protein FG07851.1 |
| FQ92HJ001ED83S | 205 | 4.00E-15 | 42/86 (48%) | Gibberella zeae PH-1 | hypothetical protein FG07853.1 |
| FQ92HJ001BQ4D8 | 305 | 1.00E-26 | 57/70 (81%) | Gibberella zeae PH-1 | hypothetical protein FG07855.1 |
| FQ92HJ001DHO9U | 77 | 3.00E+00 | 12/26 (46%) | Gibberella zeae PH-1 | hypothetical protein FG07857.1 |
| FQ4QJ5301EBON4 | 111 | 3.00E-04 | 32/84 (38%) | Gibberella zeae PH-1 | hypothetical protein FG07864.1 |
| Contig5697 | 57.4 | 3.00E-07 | 22/38 (57%) | Gibberella zeae PH-1 | hypothetical protein FG07914.1 |
| FQ4QJ5301BC75D | 313 | 1.00E-27 | 56/89 (62%) | Gibberella zeae PH-1 | hypothetical protein FG07934.1 |
| Contig2987 | 148 | 1.00E-34 | 69/79 (87%) | Gibberella zeae PH-1 | hypothetical protein FG07945.1 |
| FQ4QJ5301BL5CZ | 273 | 5.00E-23 | 50/81 (61%) | Gibberella zeae PH-1 | hypothetical protein FG08173.1 |
| Contig7397 | 52.4 | 1.00E-05 | 33/84 (39%) | Gibberella zeae PH-1 | hypothetical protein FG08222.1 |
| Contig3355 | 34.3 | 8.70E+00 | 18/51 (35%) | Gibberella zeae PH-1 | hypothetical protein FG08225.1 |
| FQ92HJ001DRB66 | 159 | 9.00E-10 | 35/59 (59%) | Gibberella zeae PH-1 | hypothetical protein FG08277.1 |
| Contig11796 | 184 | 2.00E-45 | 89/108 (82%) | Gibberella zeae PH-1 | hypothetical protein FG08296.1 |
| Contig12691 | 47.8 | 3.00E-04 | 23/33 (69%) | Gibberella zeae PH-1 | hypothetical protein FG08306.1 |
| FQ4QJ5301DGIAN | 356 | 1.00E-32 | 66/86 (76%) | Gibberella zeae PH-1 | hypothetical protein FG08313.1 |
| FQ4QJ5301BXDKA | 271 | 9.00E-23 | 48/78 (61%) | Gibberella zeae PH-1 | hypothetical protein FG08314.1 |
| FQ4QJ5301DLG79 | 212 | 6.00E-16 | 34/63 (53%) | Gibberella zeae PH-1 | hypothetical protein FG08319.1 |
| Contig4318 | 58.2 | 6.00E-11 | 36/75 (48%) | Gibberella zeae PH-1 | hypothetical protein FG08334.1 |
| FQ4QJ5301E4W6G | 353 | 3.00E-32 | 67/83 (80%) | Gibberella zeae PH-1 | hypothetical protein FG08335.1 |
| FQ4QJ5301AKV09 | 149 | 1.00E-08 | 29/57 (50%) | Gibberella zeae PH-1 | hypothetical protein FG08339.1 |
| FQ92HJ001BJYYF | 265 | 5.00E-22 | 60/85 (70%) | Gibberella zeae PH-1 | hypothetical protein FG08365.1 |
| FQ4QJ5301C0RVF | 178 | 5.00E-12 | 32/54 (59%) | Gibberella zeae PH-1 | hypothetical protein FG08380.1 |
| FQ92HJ001EB4QO | 112 | 3.00E-04 | 22/33 (66%) | Gibberella zeae PH-1 | hypothetical protein FG08382.1 |
| Contig11860 | 95.9 | 8.00E-19 | 56/119 (47%) | Gibberella zeae PH-1 | hypothetical protein FG08383.1 |
| FQ4QJ5301BS5T7 | 352 | 4.00E-32 | 55/77 (71%) | Gibberella zeae PH-1 | hypothetical protein FG08386.1 |
| FQ4QJ5301EZNGY | 337 | 2.00E-30 | 65/82 (79%) | Gibberella zeae PH-1 | hypothetical protein FG08426.1 |
| Contig16806 | 36.2 | 6.00E-03 | 17/23 (73%) | Gibberella zeae PH-1 | hypothetical protein FG08427.1 |
| FQ4QJ5301DK423 | 254 | 9.00E-21 | 58/84 (69%) | Gibberella zeae PH-1 | hypothetical protein FG08427.1 |
| FQ92HJ001DOBM0 | 127 | 3.00E-09 | 22/45 (48%) | Gibberella zeae PH-1 | hypothetical protein FG08428.1 |
| Contig14936 | 92.8 | 7.00E-18 | 44/56 (78%) | Gibberella zeae PH-1 | hypothetical protein FG08442.1 |
| Contig14760 | 201 | 1.00E-50 | 95/136 (69%) | Gibberella zeae PH-1 | hypothetical protein FG08470.1 |
| Contig12261 | 84.7 | 2.00E-15 | 42/78 (53%) | Gibberella zeae PH-1 | hypothetical protein FG08475.1 |
| Contig15106 | 166 | 4.00E-60 | 78/100 (78%) | Gibberella zeae PH-1 | hypothetical protein FG08478.1 |
| FQ4QJ5301BTK07 | 257 | 4.00E-21 | 52/96 (54%) | Gibberella zeae PH-1 | hypothetical protein FG08485.1 |
| FQ92HJ001DRH8I | 98 | 1.10E-02 | 20/30 (66%) | Gibberella zeae PH-1 | hypothetical protein FG08504.1 |
| FQ4QJ5301BSTJ5 | 273 | 5.00E-23 | 54/74 (72%) | Gibberella zeae PH-1 | hypothetical protein FG08521.1 |
| FQ92HJ001AF7RE | 76 | 3.90E+00 | 12/13 (92%) | Gibberella zeae PH-1 | hypothetical protein FG08530.1 |
| Contig6865 | 48.1 | 2.00E-12 | 27/43 (62%) | Gibberella zeae PH-1 | hypothetical protein FG08531.1 |
| Contig3212 | 79 | 1.00E-13 | 41/57 (71%) | Gibberella zeae PH-1 | hypothetical protein FG08537.1 |
| Contig3495 | 124 | 3.00E-27 | 61/80 (76%) | Gibberella zeae PH-1 | hypothetical protein FG08540.1 |
| Contig7681 | 124 | 9.00E-34 | 64/69 (92%) | Gibberella zeae PH-1 | hypothetical protein FG08555.1 |
| FQ92HJ001B2N2W | 185 | 9.00E-13 | 34/40 (85%) | Gibberella zeae PH-1 | hypothetical protein FG08557.1 |
| Contig10261 | 129 | 9.00E-29 | 64/86 (74%) | Gibberella zeae PH-1 | hypothetical protein FG08561.1 |
| FQ4QJ5301BFPTP | 164 | 2.00E-10 | 39/86 (45%) | Gibberella zeae PH-1 | hypothetical protein FG08564.1 |
| FQ4QJ5301BDV3M | 100 | 6.00E-03 | 22/51 (43%) | Gibberella zeae PH-1 | hypothetical protein FG08582.1 |
| FQ92HJ001AQBI8 | 262 | 1.00E-21 | 57/82 (69%) | Gibberella zeae PH-1 | hypothetical protein FG08598.1 |
| FQ4QJ5301A6FU8 | 337 | 2.00E-30 | 65/81 (80%) | Gibberella zeae PH-1 | hypothetical protein FG08603.1 |
| FQ4QJ5301E5SFH | 162 | 2.00E-10 | 35/74 (47%) | Gibberella zeae PH-1 | hypothetical protein FG08605.1 |
| Contig1033 | 121 | 1.00E-26 | 60/88 (68%) | Gibberella zeae PH-1 | hypothetical protein FG08621.1 |
| Contig15874 | 108 | 2.00E-22 | 51/53 (96%) | Gibberella zeae PH-1 | hypothetical protein FG08655.1 |
| FQ4QJ5301EB374 | 155 | 3.00E-09 | 39/74 (52%) | Gibberella zeae PH-1 | hypothetical protein FG08660.1 |
| FQ4QJ5301APQ0N | 372 | 2.00E-34 | 64/83 (77%) | Gibberella zeae PH-1 | hypothetical protein FG08669.1 |
| FQ4QJ5301AVNEA | 265 | 5.00E-22 | 51/74 (68%) | Gibberella zeae PH-1 | hypothetical protein FG08669.1 |
| Contig11384 | 124 | 2.00E-27 | 63/67 (94%) | Gibberella zeae PH-1 | hypothetical protein FG08675.1 |
| FQ4QJ5301EUVAI | 101 | 5.00E-03 | 17/38 (44%) | Gibberella zeae PH-1 | hypothetical protein FG08687.1 |
| FQ4QJ5301C74BS | 346 | 2.00E-31 | 69/81 (85%) | Gibberella zeae PH-1 | hypothetical protein FG08690.1 |
| FQ4QJ5301CWPG8 | 228 | 9.00E-18 | 48/86 (55%) | Gibberella zeae PH-1 | hypothetical protein FG08693.1 |
| FQ4QJ5301E17GV | 305 | 1.00E-26 | 54/73 (73%) | Gibberella zeae PH-1 | hypothetical protein FG08701.1 |
| FQ4QJ5301ALE2Z | 146 | 3.00E-08 | 27/36 (75%) | Gibberella zeae PH-1 | hypothetical protein FG08701.1 |
| FQ4QJ5301C05X5 | 358 | 8.00E-33 | 62/78 (79%) | Gibberella zeae PH-1 | hypothetical protein FG08706.1 |
| FQ4QJ5301D9WOU | 74 | 6.50E+00 | 14/28 (50%) | Gibberella zeae PH-1 | hypothetical protein FG08710.1 |
| FQ92HJ001C58GJ | 189 | 3.00E-13 | 35/44 (79%) | Gibberella zeae PH-1 | hypothetical protein FG08713.1 |
| Contig14018 | 119 | 9.00E-26 | 58/78 (74%) | Gibberella zeae PH-1 | hypothetical protein FG08723.1 |
| Contig1770 | 36.6 | 6.00E-01 | 14/14 (100%) | Gibberella zeae PH-1 | hypothetical protein FG08747.1 |
| Contig3555 | 148 | 1.00E-34 | 69/86 (80%) | Gibberella zeae PH-1 | hypothetical protein FG08747.1 |
| FQ4QJ5301BY545 | 223 | 3.00E-17 | 39/79 (49%) | Gibberella zeae PH-1 | hypothetical protein FG08748.1 |
| FQ92HJ001C7UW1 | 171 | 4.00E-11 | 35/86 (40%) | Gibberella zeae PH-1 | hypothetical protein FG08752.1 |
| Contig7787 | 129 | 5.00E-29 | 64/101 (63%) | Gibberella zeae PH-1 | hypothetical protein FG08754.1 |
| FQ4QJ5301EHRY1 | 276 | 2.00E-23 | 51/72 (70%) | Gibberella zeae PH-1 | hypothetical protein FG08755.1 |
| FQ4QJ5301C0X0M | 223 | 3.00E-17 | 38/76 (50%) | Gibberella zeae PH-1 | hypothetical protein FG08761.1 |
| FQ92HJ001CWCFP | 150 | 1.00E-08 | 37/88 (42%) | Gibberella zeae PH-1 | hypothetical protein FG08795.1 |
| Contig10227 | 52.8 | 8.00E-06 | 20/26 (76%) | Gibberella zeae PH-1 | hypothetical protein FG08814.1 |
| Contig7162 | 115 | 8.00E-25 | 55/80 (68%) | Gibberella zeae PH-1 | hypothetical protein FG08845.1 |
| FQ92HJ001DQ4SG | 188 | 4.00E-13 | 36/76 (47%) | Gibberella zeae PH-1 | hypothetical protein FG08849.1 |
| FQ92HJ001BVCS8 | 140 | 1.00E-07 | 24/37 (64%) | Gibberella zeae PH-1 | hypothetical protein FG08849.1 |
| FQ92HJ001B6C2A | 123 | 1.00E-05 | 25/31 (80%) | Gibberella zeae PH-1 | hypothetical protein FG08866.1 |
| FQ4QJ5301CHX3J | 261 | 5.00E-25 | 43/53 (81%) | Gibberella zeae PH-1 | hypothetical protein FG08874.1 |
| Contig16573 | 57.4 | 3.00E-07 | 24/35 (68%) | Gibberella zeae PH-1 | hypothetical protein FG08888.1 |
| Contig7703 | 248 | 2.00E-64 | 125/160 (78%) | Gibberella zeae PH-1 | hypothetical protein FG08895.1 |
| FQ92HJ001BV5CC | 254 | 9.00E-21 | 52/78 (66%) | Gibberella zeae PH-1 | hypothetical protein FG08910.1 |
| FQ4QJ5301DWTTM | 75 | 5.10E+00 | 14/62 (22%) | Gibberella zeae PH-1 | hypothetical protein FG08967.1 |
| FQ4QJ5301B7YZM | 89 | 1.20E-01 | 24/56 (42%) | Gibberella zeae PH-1 | hypothetical protein FG08973.1 |
| FQ4QJ5301E1IA3 | 194 | 8.00E-14 | 37/51 (72%) | Gibberella zeae PH-1 | hypothetical protein FG08976.1 |
| FQ4QJ5301AK0LG | 122 | 2.00E-05 | 27/71 (38%) | Gibberella zeae PH-1 | hypothetical protein FG08996.1 |
| FQ4QJ5301EWJ2Q | 127 | 1.00E-11 | 22/35 (62%) | Gibberella zeae PH-1 | hypothetical protein FG09002.1 |
| FQ92HJ001AL8BZ | 197 | 4.00E-14 | 38/49 (77%) | Gibberella zeae PH-1 | hypothetical protein FG09011.1 |
| Contig14258 | 127 | 2.00E-28 | 65/89 (73%) | Gibberella zeae PH-1 | hypothetical protein FG09021.1 |
| FQ4QJ5301B8602 | 266 | 3.00E-22 | 51/72 (70%) | Gibberella zeae PH-1 | hypothetical protein FG09021.1 |
| FQ4QJ5301DFPC0 | 203 | 7.00E-15 | 41/76 (53%) | Gibberella zeae PH-1 | hypothetical protein FG09022.1 |
| FQ4QJ5301DD0Y7 | 262 | 1.00E-21 | 49/81 (60%) | Gibberella zeae PH-1 | hypothetical protein FG09025.1 |
| FQ4QJ5301BAU01 | 327 | 3.00E-29 | 58/73 (79%) | Gibberella zeae PH-1 | hypothetical protein FG09034.1 |
| FQ4QJ5301B6BPK | 100 | 1.00E-03 | 22/35 (62%) | Gibberella zeae PH-1 | hypothetical protein FG09035.1 |
| FQ92HJ001ETJSW | 74 | 6.50E+00 | 18/44 (40%) | Gibberella zeae PH-1 | hypothetical protein FG09057.1 |
| Contig9291 | 58.2 | 2.00E-07 | 26/54 (48%) | Gibberella zeae PH-1 | hypothetical protein FG09060.1 |
| FQ92HJ001DNMBH | 372 | 2.00E-34 | 65/76 (85%) | Gibberella zeae PH-1 | hypothetical protein FG09081.1 |
| Contig2106 | 81.3 | 2.00E-14 | 46/89 (51%) | Gibberella zeae PH-1 | hypothetical protein FG09163.1 |
| FQ4QJ5301DEQD0 | 214 | 4.00E-16 | 46/70 (65%) | Gibberella zeae PH-1 | hypothetical protein FG09164.1 |
| FQ4QJ5301BVEX8 | 317 | 4.00E-28 | 58/69 (84%) | Gibberella zeae PH-1 | hypothetical protein FG09165.1 |
| FQ4QJ5301BDS5A | 119 | 4.00E-05 | 24/54 (44%) | Gibberella zeae PH-1 | hypothetical protein FG09203.1 |
| Contig9485 | 70.9 | 3.00E-15 | 34/49 (69%) | Gibberella zeae PH-1 | hypothetical protein FG09216.1 |
| FQ4QJ5301C8M94 | 398 | 2.00E-37 | 71/88 (80%) | Gibberella zeae PH-1 | hypothetical protein FG09223.1 |
| FQ4QJ5301DJ8XC | 175 | 1.00E-11 | 38/78 (48%) | Gibberella zeae PH-1 | hypothetical protein FG09231.1 |
| FQ92HJ001BJ7RI | 256 | 5.00E-21 | 50/55 (90%) | Gibberella zeae PH-1 | hypothetical protein FG09234.1 |
| Contig5880 | 149 | 5.00E-35 | 70/80 (87%) | Gibberella zeae PH-1 | hypothetical protein FG09241.1 |
| FQ4QJ5301E0K8X | 180 | 3.00E-12 | 30/47 (63%) | Gibberella zeae PH-1 | hypothetical protein FG09273.1 |
| FQ4QJ5301DQ3A9 | 87 | 2.00E-06 | 22/48 (45%) | Gibberella zeae PH-1 | hypothetical protein FG09274.1 |
| Contig7970 | 227 | 2.00E-68 | 110/128 (85%) | Gibberella zeae PH-1 | hypothetical protein FG09284.1 |
| Contig15696 | 60.8 | 3.00E-08 | 28/52 (53%) | Gibberella zeae PH-1 | hypothetical protein FG09284.1 |
| Contig1839 | 56.6 | 5.00E-07 | 43/98 (43%) | Gibberella zeae PH-1 | hypothetical protein FG09286.1 |
| Contig2514 | 102 | 9.00E-21 | 79/166 (47%) | Gibberella zeae PH-1 | hypothetical protein FG09286.1 |
| FQ4QJ5301AVNRJ | 201 | 1.00E-14 | 41/50 (82%) | Gibberella zeae PH-1 | hypothetical protein FG09299.1 |
| Contig408 | 120 | 3.00E-26 | 50/80 (62%) | Gibberella zeae PH-1 | hypothetical protein FG09306.1 |
| FQ4QJ5301DCXGL | 102 | 4.00E-03 | 33/88 (37%) | Gibberella zeae PH-1 | hypothetical protein FG09309.1 |
| Contig1305 | 59.7 | 7.00E-08 | 26/55 (47%) | Gibberella zeae PH-1 | hypothetical protein FG09314.1 |
| FQ92HJ001DHHFA | 195 | 6.00E-14 | 35/49 (71%) | Gibberella zeae PH-1 | hypothetical protein FG09316.1 |
| FQ92HJ001B9523 | 131 | 2.00E-06 | 24/35 (68%) | Gibberella zeae PH-1 | hypothetical protein FG09319.1 |
| FQ4QJ5301AQ2KE | 170 | 4.00E-12 | 31/52 (59%) | Gibberella zeae PH-1 | hypothetical protein FG09322.1 |
| FQ4QJ5301BKA6V | 236 | 1.00E-18 | 44/57 (77%) | Gibberella zeae PH-1 | hypothetical protein FG09338.1 |
| FQ92HJ001DWX4V | 131 | 2.00E-15 | 26/30 (86%) | Gibberella zeae PH-1 | hypothetical protein FG09361.1 |
| Contig4262 | 104 | 2.00E-21 | 50/78 (64%) | Gibberella zeae PH-1 | hypothetical protein FG09362.1 |
| FQ92HJ001EEYCG | 299 | 5.00E-26 | 56/78 (71%) | Gibberella zeae PH-1 | hypothetical protein FG09362.1 |
| Contig4586 | 49.3 | 9.00E-05 | 22/29 (75%) | Gibberella zeae PH-1 | hypothetical protein FG09374.1 |
| Contig10186 | 78.2 | 2.00E-13 | 37/65 (56%) | Gibberella zeae PH-1 | hypothetical protein FG09374.1 |
| FQ92HJ001BS0II | 358 | 8.00E-33 | 63/76 (82%) | Gibberella zeae PH-1 | hypothetical protein FG09381.1 |
| Contig2597 | 41.2 | 2.40E-02 | 18/32 (56%) | Gibberella zeae PH-1 | hypothetical protein FG09384.1 |
| Contig7219 | 48.1 | 2.00E-04 | 19/22 (86%) | Gibberella zeae PH-1 | hypothetical protein FG09386.1 |
| FQ4QJ5301EO0RQ | 166 | 2.00E-21 | 29/42 (69%) | Gibberella zeae PH-1 | hypothetical protein FG09393.1 |
| Contig3660 | 91.7 | 2.00E-17 | 48/85 (56%) | Gibberella zeae PH-1 | hypothetical protein FG09396.1 |
| Contig8312 | 59.3 | 9.00E-08 | 29/51 (56%) | Gibberella zeae PH-1 | hypothetical protein FG09396.1 |
| Contig7729 | 46.6 | 6.00E-04 | 21/27 (77%) | Gibberella zeae PH-1 | hypothetical protein FG09402.1 |
| FQ4QJ5301EF822 | 132 | 1.00E-06 | 27/43 (62%) | Gibberella zeae PH-1 | hypothetical protein FG09413.1 |
| FQ4QJ5301C4G9O | 212 | 7.00E-16 | 39/53 (73%) | Gibberella zeae PH-1 | hypothetical protein FG09414.1 |
| FQ92HJ001BUACX | 185 | 9.00E-13 | 37/62 (59%) | Gibberella zeae PH-1 | hypothetical protein FG09426.1 |
| FQ4QJ5301COP8P | 333 | 6.00E-30 | 66/84 (78%) | Gibberella zeae PH-1 | hypothetical protein FG09435.1 |
| FQ4QJ5301DI9O5 | 83 | 5.90E-01 | 22/71 (30%) | Gibberella zeae PH-1 | hypothetical protein FG09447.1 |
| FQ92HJ001A1NHW | 289 | 8.00E-25 | 53/85 (62%) | Gibberella zeae PH-1 | hypothetical protein FG09448.1 |
| FQ4QJ5301CH5XU | 140 | 1.00E-07 | 25/41 (60%) | Gibberella zeae PH-1 | hypothetical protein FG09456.1 |
| Contig11806 | 117 | 2.00E-25 | 52/89 (58%) | Gibberella zeae PH-1 | hypothetical protein FG09469.1 |
| Contig3257 | 152 | 7.00E-36 | 76/101 (75%) | Gibberella zeae PH-1 | hypothetical protein FG09505.1 |
| Contig4369 | 163 | 2.00E-42 | 76/97 (78%) | Gibberella zeae PH-1 | hypothetical protein FG09512.1 |
| FQ92HJ001DTNIH | 171 | 4.00E-11 | 32/37 (86%) | Gibberella zeae PH-1 | hypothetical protein FG09528.1 |
| FQ4QJ5301A2OIJ | 90 | 9.20E-02 | 15/16 (93%) | Gibberella zeae PH-1 | hypothetical protein FG09531.1 |
| FQ4QJ5301B57MR | 110 | 4.00E-04 | 24/43 (55%) | Gibberella zeae PH-1 | hypothetical protein FG09534.1 |
| Contig7009 | 144 | 2.00E-33 | 69/83 (83%) | Gibberella zeae PH-1 | hypothetical protein FG09535.1 |
| FQ4QJ5301D3LSV | 186 | 7.00E-13 | 44/90 (48%) | Gibberella zeae PH-1 | hypothetical protein FG09542.1 |
| Contig12696 | 74.3 | 3.00E-12 | 35/49 (71%) | Gibberella zeae PH-1 | hypothetical protein FG09545.1 |
| Contig5905 | 117 | 3.00E-25 | 48/55 (87%) | Gibberella zeae PH-1 | hypothetical protein FG09546.1 |
| FQ4QJ5301BNNHJ | 358 | 8.00E-33 | 71/80 (88%) | Gibberella zeae PH-1 | hypothetical protein FG09546.1 |
| Contig12952 | 63.5 | 5.00E-09 | 28/38 (73%) | Gibberella zeae PH-1 | hypothetical protein FG09552.1 |
| Contig16545 | 98.2 | 2.00E-19 | 48/88 (54%) | Gibberella zeae PH-1 | hypothetical protein FG09552.1 |
| Contig2583 | 118 | 2.00E-25 | 55/117 (47%) | Gibberella zeae PH-1 | hypothetical protein FG09557.1 |
| FQ4QJ5301BX75I | 264 | 6.00E-22 | 43/67 (64%) | Gibberella zeae PH-1 | hypothetical protein FG09560.1 |
| FQ4QJ5301A0MLU | 81 | 1.00E+00 | 20/46 (43%) | Gibberella zeae PH-1 | hypothetical protein FG09566.1 |
| FQ4QJ5301D83R1 | 373 | 1.00E-34 | 68/81 (83%) | Gibberella zeae PH-1 | hypothetical protein FG09572.1 |
| FQ92HJ001A2T5A | 138 | 2.00E-07 | 29/39 (74%) | Gibberella zeae PH-1 | hypothetical protein FG09576.1 |
| Contig380 | 47 | 4.00E-04 | 24/51 (47%) | Gibberella zeae PH-1 | hypothetical protein FG09580.1 |
| Contig10155 | 108 | 1.00E-22 | 51/66 (77%) | Gibberella zeae PH-1 | hypothetical protein FG09601.1 |
| Contig13601 | 148 | 1.00E-34 | 69/74 (93%) | Gibberella zeae PH-1 | hypothetical protein FG09603.1 |
| FQ4QJ5301B8P2U | 274 | 4.00E-23 | 50/69 (72%) | Gibberella zeae PH-1 | hypothetical protein FG09610.1 |
| FQ4QJ5301CO9DP | 76 | 3.80E+00 | 18/51 (35%) | Gibberella zeae PH-1 | hypothetical protein FG09611.1 |
| FQ4QJ5301DV90W | 200 | 2.00E-14 | 36/52 (69%) | Gibberella zeae PH-1 | hypothetical protein FG09632.1 |
| Contig16611 | 102 | 9.00E-21 | 52/69 (75%) | Gibberella zeae PH-1 | hypothetical protein FG09642.1 |
| FQ92HJ001A6H4F | 294 | 2.00E-25 | 55/85 (64%) | Gibberella zeae PH-1 | hypothetical protein FG09647.1 |
| Contig15869 | 65.1 | 2.00E-09 | 33/77 (42%) | Gibberella zeae PH-1 | hypothetical protein FG09667.1 |
| Contig6423 | 69.3 | 3.00E-13 | 36/67 (53%) | Gibberella zeae PH-1 | hypothetical protein FG09689.1 |
| FQ92HJ001A2RQZ | 274 | 4.00E-23 | 50/62 (80%) | Gibberella zeae PH-1 | hypothetical protein FG09689.1 |
| Contig4460 | 106 | 5.00E-22 | 48/63 (76%) | Gibberella zeae PH-1 | hypothetical protein FG09711.1 |
| FQ4QJ5301BJCEN | 237 | 8.00E-19 | 48/75 (64%) | Gibberella zeae PH-1 | hypothetical protein FG09711.1 |
| FQ4QJ5301EB8GX | 178 | 6.00E-12 | 35/47 (74%) | Gibberella zeae PH-1 | hypothetical protein FG09713.1 |
| FQ92HJ001E259R | 105 | 2.00E-03 | 27/61 (44%) | Gibberella zeae PH-1 | hypothetical protein FG09716.1 |
| Contig5144 | 48.5 | 1.00E-04 | 20/25 (80%) | Gibberella zeae PH-1 | hypothetical protein FG09760.1 |
| FQ4QJ5301BYUI7 | 312 | 2.00E-27 | 57/77 (74%) | Gibberella zeae PH-1 | hypothetical protein FG09760.1 |
| FQ4QJ5301A3CVS | 240 | 4.00E-19 | 45/61 (73%) | Gibberella zeae PH-1 | hypothetical protein FG09760.1 |
| Contig2453 | 101 | 2.00E-20 | 51/73 (69%) | Gibberella zeae PH-1 | hypothetical protein FG09764.1 |
| FQ4QJ5301ESMD1 | 321 | 2.00E-28 | 54/84 (64%) | Gibberella zeae PH-1 | hypothetical protein FG09768.1 |
| FQ4QJ5301C0QE9 | 249 | 2.00E-26 | 54/76 (71%) | Gibberella zeae PH-1 | hypothetical protein FG09783.1 |
| FQ4QJ5301EQGJW | 94 | 2.40E-02 | 17/17 (100%) | Gibberella zeae PH-1 | hypothetical protein FG09785.1 |
| FQ4QJ5301ENQLO | 225 | 2.00E-17 | 45/70 (64%) | Gibberella zeae PH-1 | hypothetical protein FG09794.1 |
| FQ4QJ5301DWZUH | 111 | 1.00E-05 | 24/33 (72%) | Gibberella zeae PH-1 | hypothetical protein FG09794.1 |
| Contig6213 | 39.3 | 9.00E-02 | 22/41 (53%) | Gibberella zeae PH-1 | hypothetical protein FG09798.1 |
| FQ4QJ5301DQK2Q | 168 | 9.00E-24 | 30/41 (73%) | Gibberella zeae PH-1 | hypothetical protein FG09798.1 |
| FQ4QJ5301BYWKO | 308 | 5.00E-27 | 56/70 (80%) | Gibberella zeae PH-1 | hypothetical protein FG09828.1 |
| FQ4QJ5301EL6CJ | 325 | 5.00E-29 | 65/78 (83%) | Gibberella zeae PH-1 | hypothetical protein FG09828.1 |
| Contig2372 | 105 | 1.00E-21 | 47/80 (58%) | Gibberella zeae PH-1 | hypothetical protein FG09831.1 |
| FQ4QJ5301BAEJQ | 199 | 2.00E-14 | 30/36 (83%) | Gibberella zeae PH-1 | hypothetical protein FG09833.1 |
| Contig12529 | 37.7 | 2.70E-01 | 20/40 (50%) | Gibberella zeae PH-1 | hypothetical protein FG09836.1 |
| FQ4QJ5301B1HQT | 164 | 1.00E-14 | 29/38 (76%) | Gibberella zeae PH-1 | hypothetical protein FG09839.1 |
| Contig11127 | 54.3 | 3.00E-06 | 26/63 (41%) | Gibberella zeae PH-1 | hypothetical protein FG09843.1 |
| FQ4QJ5301C3UTM | 333 | 6.00E-30 | 66/79 (83%) | Gibberella zeae PH-1 | hypothetical protein FG09844.1 |
| FQ4QJ5301EA5S9 | 228 | 9.00E-18 | 45/81 (55%) | Gibberella zeae PH-1 | hypothetical protein FG09846.1 |
| FQ4QJ5301A79QH | 386 | 4.00E-36 | 77/82 (93%) | Gibberella zeae PH-1 | hypothetical protein FG09846.1 |
| FQ92HJ001DIFNV | 219 | 1.00E-16 | 36/47 (76%) | Gibberella zeae PH-1 | hypothetical protein FG09852.1 |
| Contig3049 | 80.9 | 3.00E-14 | 38/77 (49%) | Gibberella zeae PH-1 | hypothetical protein FG09861.1 |
| FQ4QJ5301AFZ44 | 322 | 1.00E-28 | 63/78 (80%) | Gibberella zeae PH-1 | hypothetical protein FG09865.1 |
| FQ92HJ001CTFR5 | 184 | 4.00E-24 | 38/45 (84%) | Gibberella zeae PH-1 | hypothetical protein FG09866.1 |
| Contig9623 | 47 | 4.00E-04 | 19/28 (67%) | Gibberella zeae PH-1 | hypothetical protein FG09869.1 |
| FQ4QJ5301A0ZXR | 164 | 2.00E-10 | 30/51 (58%) | Gibberella zeae PH-1 | hypothetical protein FG09875.1 |
| FQ4QJ5301CDEOV | 173 | 2.00E-11 | 32/43 (74%) | Gibberella zeae PH-1 | hypothetical protein FG09879.1 |
| Contig7985 | 60.1 | 5.00E-08 | 31/55 (56%) | Gibberella zeae PH-1 | hypothetical protein FG09892.1 |
| FQ4QJ5301B5Q9P | 195 | 4.00E-27 | 36/41 (87%) | Gibberella zeae PH-1 | hypothetical protein FG09892.1 |
| FQ4QJ5301DFDJO | 124 | 1.00E-05 | 26/42 (61%) | Gibberella zeae PH-1 | hypothetical protein FG09902.1 |
| FQ4QJ5301D0OTS | 332 | 8.00E-30 | 63/80 (78%) | Gibberella zeae PH-1 | hypothetical protein FG09904.1 |
| Contig5510 | 77.4 | 3.00E-13 | 38/76 (50%) | Gibberella zeae PH-1 | hypothetical protein FG09906.1 |
| Contig4063 | 100 | 4.00E-20 | 46/92 (50%) | Gibberella zeae PH-1 | hypothetical protein FG09926.1 |
| FQ4QJ5301COZ0T | 310 | 3.00E-27 | 55/63 (87%) | Gibberella zeae PH-1 | hypothetical protein FG09929.1 |
| FQ4QJ5301C9MG9 | 168 | 8.00E-11 | 34/74 (45%) | Gibberella zeae PH-1 | hypothetical protein FG09954.1 |
| FQ92HJ001CFKMI | 342 | 6.00E-31 | 63/87 (72%) | Gibberella zeae PH-1 | hypothetical protein FG09956.1 |
| FQ4QJ5301CNGM7 | 352 | 4.00E-32 | 64/87 (73%) | Gibberella zeae PH-1 | hypothetical protein FG09958.1 |
| Contig2293 | 65.5 | 1.00E-09 | 34/64 (53%) | Gibberella zeae PH-1 | hypothetical protein FG09962.1 |
| FQ92HJ001D94Q3 | 307 | 6.00E-27 | 62/84 (73%) | Gibberella zeae PH-1 | hypothetical protein FG09962.1 |
| FQ4QJ5301CEI39 | 192 | 1.00E-28 | 36/36 (100%) | Gibberella zeae PH-1 | hypothetical protein FG09966.1 |
| FQ92HJ001B8UO8 | 131 | 2.00E-06 | 23/37 (62%) | Gibberella zeae PH-1 | hypothetical protein FG09980.1 |
| FQ4QJ5301DBVYY | 80 | 1.30E+00 | 15/21 (71%) | Gibberella zeae PH-1 | hypothetical protein FG09983.1 |
| FQ4QJ5301B3L2B | 283 | 4.00E-24 | 50/60 (83%) | Gibberella zeae PH-1 | hypothetical protein FG09984.1 |
| FQ4QJ5301EN2H3 | 357 | 1.00E-32 | 64/83 (77%) | Gibberella zeae PH-1 | hypothetical protein FG09998.1 |
| FQ4QJ5301APYB0 | 300 | 4.00E-26 | 53/61 (86%) | Gibberella zeae PH-1 | hypothetical protein FG09998.1 |
| Contig6494 | 66.2 | 7.00E-10 | 28/29 (96%) | Gibberella zeae PH-1 | hypothetical protein FG10003.1 |
| FQ4QJ5301EA0C3 | 152 | 6.00E-09 | 36/92 (39%) | Gibberella zeae PH-1 | hypothetical protein FG10015.1 |
| FQ4QJ5301DE5WS | 224 | 3.00E-17 | 42/58 (72%) | Gibberella zeae PH-1 | hypothetical protein FG10018.1 |
| Contig14264 | 50.4 | 4.00E-05 | 20/28 (71%) | Gibberella zeae PH-1 | hypothetical protein FG10021.1 |
| FQ4QJ5301DK87C | 263 | 8.00E-22 | 53/70 (75%) | Gibberella zeae PH-1 | hypothetical protein FG10037.1 |
| Contig378 | 99.4 | 7.00E-20 | 46/53 (86%) | Gibberella zeae PH-1 | hypothetical protein FG10040.1 |
| FQ4QJ5301C76G1 | 368 | 5.00E-34 | 72/80 (90%) | Gibberella zeae PH-1 | hypothetical protein FG10040.1 |
| Contig11370 | 107 | 3.00E-22 | 52/55 (94%) | Gibberella zeae PH-1 | hypothetical protein FG10049.1 |
| FQ4QJ5301D5PDB | 363 | 2.00E-33 | 69/78 (88%) | Gibberella zeae PH-1 | hypothetical protein FG10049.1 |
| FQ92HJ001A6ZGH | 257 | 4.00E-21 | 50/80 (62%) | Gibberella zeae PH-1 | hypothetical protein FG10053.1 |
| FQ4QJ5301D9EJ1 | 145 | 5.00E-16 | 29/55 (52%) | Gibberella zeae PH-1 | hypothetical protein FG10054.1 |
| Contig15229 | 215 | 1.00E-54 | 104/151 (68%) | Gibberella zeae PH-1 | hypothetical protein FG10067.1 |
| FQ4QJ5301DW744 | 146 | 3.00E-08 | 25/60 (41%) | Gibberella zeae PH-1 | hypothetical protein FG10071.1 |
| FQ4QJ5301A6ZJ1 | 134 | 5.00E-07 | 26/42 (61%) | Gibberella zeae PH-1 | hypothetical protein FG10072.1 |
| FQ92HJ001AI4M7 | 245 | 1.00E-19 | 45/57 (78%) | Gibberella zeae PH-1 | hypothetical protein FG10077.1 |
| FQ4QJ5301A14LE | 126 | 6.00E-06 | 31/56 (55%) | Gibberella zeae PH-1 | hypothetical protein FG10078.1 |
| FQ92HJ001AQKV2 | 110 | 4.00E-04 | 20/29 (68%) | Gibberella zeae PH-1 | hypothetical protein FG10088.1 |
| FQ92HJ001DTT7Y | 250 | 3.00E-20 | 49/62 (79%) | Gibberella zeae PH-1 | hypothetical protein FG10090.1 |
| FQ4QJ5301E03RI | 258 | 3.00E-21 | 49/80 (61%) | Gibberella zeae PH-1 | hypothetical protein FG10095.1 |
| FQ92HJ001BR8YY | 302 | 2.00E-26 | 56/72 (77%) | Gibberella zeae PH-1 | hypothetical protein FG10123.1 |
| Contig15608 | 53.1 | 6.00E-06 | 31/83 (37%) | Gibberella zeae PH-1 | hypothetical protein FG10125.1 |
| FQ92HJ001D2FLJ | 76 | 3.80E+00 | 14/20 (70%) | Gibberella zeae PH-1 | hypothetical protein FG10125.1 |
| Contig591 | 33.9 | 3.80E+00 | 18/60 (30%) | Gibberella zeae PH-1 | hypothetical protein FG10129.1 |
| FQ4QJ5301DQOG0 | 292 | 3.00E-25 | 56/79 (70%) | Gibberella zeae PH-1 | hypothetical protein FG10138.1 |
| FQ4QJ5301ENQUM | 196 | 5.00E-14 | 40/82 (48%) | Gibberella zeae PH-1 | hypothetical protein FG10147.1 |
| FQ4QJ5301BB63G | 122 | 2.00E-05 | 29/77 (37%) | Gibberella zeae PH-1 | hypothetical protein FG10148.1 |
| FQ4QJ5301B6MPS | 103 | 2.00E-06 | 19/23 (82%) | Gibberella zeae PH-1 | hypothetical protein FG10169.1 |
| FQ4QJ5301A5QY4 | 217 | 2.00E-16 | 45/81 (55%) | Gibberella zeae PH-1 | hypothetical protein FG10180.1 |
| FQ92HJ001B4FEJ | 92 | 5.00E-05 | 16/21 (76%) | Gibberella zeae PH-1 | hypothetical protein FG10185.1 |
| FQ92HJ001CW2G9 | 251 | 2.00E-20 | 48/83 (57%) | Gibberella zeae PH-1 | hypothetical protein FG10194.1 |
| FQ92HJ001A0LKO | 111 | 3.00E-09 | 23/40 (57%) | Gibberella zeae PH-1 | hypothetical protein FG10194.1 |
| FQ92HJ001DF9J6 | 210 | 1.00E-15 | 41/74 (55%) | Gibberella zeae PH-1 | hypothetical protein FG10196.1 |
| Contig9413 | 99.4 | 7.00E-20 | 46/63 (73%) | Gibberella zeae PH-1 | hypothetical protein FG10198.1 |
| FQ4QJ5301BKQDF | 319 | 3.00E-28 | 62/80 (77%) | Gibberella zeae PH-1 | hypothetical protein FG10215.1 |
| Contig1247 | 53.1 | 6.00E-06 | 21/52 (40%) | Gibberella zeae PH-1 | hypothetical protein FG10217.1 |
| Contig6436 | 105 | 1.00E-21 | 47/75 (62%) | Gibberella zeae PH-1 | hypothetical protein FG10217.1 |
| FQ4QJ5301ARKPN | 244 | 1.00E-19 | 38/61 (62%) | Gibberella zeae PH-1 | hypothetical protein FG10226.1 |
| FQ4QJ5301DUAIR | 117 | 7.00E-05 | 26/49 (53%) | Gibberella zeae PH-1 | hypothetical protein FG10239.1 |
| FQ4QJ5301EZ0CJ | 114 | 1.00E-04 | 23/58 (39%) | Gibberella zeae PH-1 | hypothetical protein FG10240.1 |
| Contig2789 | 108 | 1.00E-22 | 55/59 (93%) | Gibberella zeae PH-1 | hypothetical protein FG10245.1 |
| FQ92HJ001BCK51 | 286 | 2.00E-24 | 58/62 (93%) | Gibberella zeae PH-1 | hypothetical protein FG10245.1 |
| Contig9255 | 249 | 6.00E-65 | 117/119 (98%) | Gibberella zeae PH-1 | hypothetical protein FG10246.1 |
| FQ4QJ5301CZM7S | 177 | 7.00E-12 | 30/56 (53%) | Gibberella zeae PH-1 | hypothetical protein FG10253.1 |
| FQ4QJ5301DSHUQ | 280 | 8.00E-24 | 51/54 (94%) | Gibberella zeae PH-1 | hypothetical protein FG10255.1 |
| FQ92HJ001DFVOO | 180 | 3.00E-12 | 43/76 (56%) | Gibberella zeae PH-1 | hypothetical protein FG10283.1 |
| FQ4QJ5301DZH8E | 73 | 8.60E+00 | 12/14 (85%) | Gibberella zeae PH-1 | hypothetical protein FG10286.1 |
| FQ4QJ5301ELG5G | 190 | 2.00E-13 | 33/45 (73%) | Gibberella zeae PH-1 | hypothetical protein FG10293.1 |
| FQ4QJ5301DM9M5 | 299 | 5.00E-26 | 58/80 (72%) | Gibberella zeae PH-1 | hypothetical protein FG10296.1 |
| FQ4QJ5301EA8W0 | 84 | 4.60E-01 | 15/18 (83%) | Gibberella zeae PH-1 | hypothetical protein FG10304.1 |
| Contig15658 | 84 | 3.00E-15 | 41/69 (59%) | Gibberella zeae PH-1 | hypothetical protein FG10305.1 |
| FQ4QJ5301CUEA4 | 365 | 1.00E-33 | 75/78 (96%) | Gibberella zeae PH-1 | hypothetical protein FG10306.1 |
| Contig2227 | 139 | 5.00E-32 | 68/74 (91%) | Gibberella zeae PH-1 | hypothetical protein FG10320.1 |
| Contig4400 | 113 | 1.00E-23 | 54/99 (54%) | Gibberella zeae PH-1 | hypothetical protein FG10352.1 |
| FQ4QJ5301DYQNM | 361 | 3.00E-33 | 72/85 (84%) | Gibberella zeae PH-1 | hypothetical protein FG10367.1 |
| Contig2463 | 170 | 3.00E-41 | 77/110 (70%) | Gibberella zeae PH-1 | hypothetical protein FG10374.1 |
| FQ4QJ5301D414W | 321 | 1.00E-28 | 55/57 (96%) | Gibberella zeae PH-1 | hypothetical protein FG10374.1 |
| FQ4QJ5301DKPZB | 248 | 4.00E-20 | 44/68 (64%) | Gibberella zeae PH-1 | hypothetical protein FG10388.1 |
| FQ92HJ001EEQI2 | 288 | 1.00E-24 | 56/76 (73%) | Gibberella zeae PH-1 | hypothetical protein FG10388.1 |
| FQ92HJ001AZ1Y8 | 91 | 7.00E-02 | 15/23 (65%) | Gibberella zeae PH-1 | hypothetical protein FG10401.1 |
| FQ4QJ5301E1YS8 | 109 | 6.00E-04 | 20/30 (66%) | Gibberella zeae PH-1 | hypothetical protein FG10425.1 |
| FQ4QJ5301C826L | 171 | 4.00E-11 | 31/34 (91%) | Gibberella zeae PH-1 | hypothetical protein FG10511.1 |
| FQ4QJ5301CQY1P | 77 | 2.90E+00 | 14/28 (50%) | Gibberella zeae PH-1 | hypothetical protein FG10516.1 |
| FQ92HJ001EI3TZ | 152 | 6.00E-09 | 32/62 (51%) | Gibberella zeae PH-1 | hypothetical protein FG10529.1 |
| FQ4QJ5301BY7XR | 161 | 5.00E-10 | 34/79 (43%) | Gibberella zeae PH-1 | hypothetical protein FG10610.1 |
| FQ92HJ001AX6SC | 168 | 8.00E-11 | 27/44 (61%) | Gibberella zeae PH-1 | hypothetical protein FG10618.1 |
| FQ4QJ5301CWJUC | 376 | 6.00E-35 | 68/86 (79%) | Gibberella zeae PH-1 | hypothetical protein FG10716.1 |
| FQ92HJ001A5UH6 | 146 | 3.00E-08 | 28/80 (35%) | Gibberella zeae PH-1 | hypothetical protein FG10720.1 |
| FQ4QJ5301DZOUE | 197 | 4.00E-14 | 46/82 (56%) | Gibberella zeae PH-1 | hypothetical protein FG10725.1 |
| FQ4QJ5301DXRXZ | 365 | 1.00E-33 | 67/76 (88%) | Gibberella zeae PH-1 | hypothetical protein FG10731.1 |
| Contig2250 | 59.3 | 3.00E-14 | 30/65 (46%) | Gibberella zeae PH-1 | hypothetical protein FG10734.1 |
| FQ4QJ5301D94BJ | 115 | 1.00E-04 | 28/77 (36%) | Gibberella zeae PH-1 | hypothetical protein FG10736.1 |
| FQ92HJ001DDF1T | 110 | 1.00E-10 | 23/36 (63%) | Gibberella zeae PH-1 | hypothetical protein FG10736.1 |
| FQ4QJ5301C29ZS | 284 | 3.00E-24 | 54/79 (68%) | Gibberella zeae PH-1 | hypothetical protein FG10742.1 |
| FQ4QJ5301BC7QO | 326 | 4.00E-29 | 62/76 (81%) | Gibberella zeae PH-1 | hypothetical protein FG10799.1 |
| FQ4QJ5301EGWD1 | 346 | 2.00E-31 | 63/84 (75%) | Gibberella zeae PH-1 | hypothetical protein FG10819.1 |
| FQ4QJ5301AKZLV | 217 | 2.00E-16 | 42/61 (68%) | Gibberella zeae PH-1 | hypothetical protein FG10821.1 |
| FQ4QJ5301EC2CF | 206 | 3.00E-15 | 41/53 (77%) | Gibberella zeae PH-1 | hypothetical protein FG10822.1 |
| FQ4QJ5301AU07U | 244 | 1.00E-19 | 54/81 (66%) | Gibberella zeae PH-1 | hypothetical protein FG10830.1 |
| Contig10739 | 203 | 2.00E-50 | 97/136 (71%) | Gibberella zeae PH-1 | hypothetical protein FG10831.1 |
| Contig837 | 122 | 1.00E-26 | 62/86 (72%) | Gibberella zeae PH-1 | hypothetical protein FG10836.1 |
| FQ4QJ5301DHXBM | 348 | 1.00E-31 | 66/83 (79%) | Gibberella zeae PH-1 | hypothetical protein FG10840.1 |
| FQ92HJ001EKSFR | 312 | 2.00E-27 | 59/78 (75%) | Gibberella zeae PH-1 | hypothetical protein FG10840.1 |
| Contig6286 | 45.4 | 1.00E-03 | 21/23 (91%) | Gibberella zeae PH-1 | hypothetical protein FG10845.1 |
| Contig16672 | 106 | 6.00E-22 | 49/61 (80%) | Gibberella zeae PH-1 | hypothetical protein FG10854.1 |
| FQ4QJ5301B3SUS | 111 | 3.00E-04 | 27/73 (36%) | Gibberella zeae PH-1 | hypothetical protein FG10862.1 |
| Contig586 | 98.6 | 1.00E-19 | 47/73 (64%) | Gibberella zeae PH-1 | hypothetical protein FG10864.1 |
| Contig2137 | 57.4 | 3.00E-07 | 28/31 (90%) | Gibberella zeae PH-1 | hypothetical protein FG10864.1 |
| FQ4QJ5301A5I71 | 183 | 1.00E-12 | 38/73 (52%) | Gibberella zeae PH-1 | hypothetical protein FG10865.1 |
| FQ4QJ5301C8PFS | 173 | 2.00E-11 | 42/84 (50%) | Gibberella zeae PH-1 | hypothetical protein FG10874.1 |
| Contig2546 | 81.6 | 2.00E-14 | 46/68 (67%) | Gibberella zeae PH-1 | hypothetical protein FG10875.1 |
| FQ92HJ001AU4VI | 119 | 4.00E-05 | 25/57 (43%) | Gibberella zeae PH-1 | hypothetical protein FG10878.1 |
| FQ92HJ001EJ9E7 | 152 | 6.00E-09 | 30/38 (78%) | Gibberella zeae PH-1 | hypothetical protein FG10879.1 |
| Contig10550 | 140 | 2.00E-32 | 65/86 (75%) | Gibberella zeae PH-1 | hypothetical protein FG10893.1 |
| Contig10682 | 78.2 | 2.00E-13 | 36/59 (61%) | Gibberella zeae PH-1 | hypothetical protein FG10908.1 |
| FQ4QJ5301DK5YO | 161 | 5.00E-10 | 30/81 (37%) | Gibberella zeae PH-1 | hypothetical protein FG10910.1 |
| FQ4QJ5301BKJCC | 252 | 2.00E-20 | 40/68 (58%) | Gibberella zeae PH-1 | hypothetical protein FG10913.1 |
| FQ4QJ5301A9F9A | 151 | 7.00E-10 | 29/56 (51%) | Gibberella zeae PH-1 | hypothetical protein FG10913.1 |
| FQ92HJ001CEVO5 | 272 | 7.00E-23 | 50/70 (71%) | Gibberella zeae PH-1 | hypothetical protein FG10925.1 |
| Contig10058 | 102 | 9.00E-21 | 48/56 (85%) | Gibberella zeae PH-1 | hypothetical protein FG10927.1 |
| FQ4QJ5301AS5IL | 254 | 9.00E-21 | 52/82 (63%) | Gibberella zeae PH-1 | hypothetical protein FG10929.1 |
| Contig4317 | 36.2 | 7.70E-01 | 15/23 (65%) | Gibberella zeae PH-1 | hypothetical protein FG10934.1 |
| Contig15641 | 111 | 9.00E-30 | 50/54 (92%) | Gibberella zeae PH-1 | hypothetical protein FG10941.1 |
| FQ92HJ001DSJPY | 253 | 1.00E-20 | 44/66 (66%) | Gibberella zeae PH-1 | hypothetical protein FG10960.1 |
| FQ4QJ5301DJMLN | 136 | 4.00E-07 | 29/78 (37%) | Gibberella zeae PH-1 | hypothetical protein FG10965.1 |
| FQ4QJ5301DG532 | 239 | 5.00E-19 | 40/80 (50%) | Gibberella zeae PH-1 | hypothetical protein FG10965.1 |
| FQ92HJ001AQEH3 | 111 | 3.00E-04 | 19/32 (59%) | Gibberella zeae PH-1 | hypothetical protein FG10975.1 |
| FQ4QJ5301AS3FY | 162 | 4.00E-10 | 30/82 (36%) | Gibberella zeae PH-1 | hypothetical protein FG11010.1 |
| FQ4QJ5301AL901 | 73 | 8.40E+00 | 13/39 (33%) | Gibberella zeae PH-1 | hypothetical protein FG11086.1 |
| Contig3890 | 32.7 | 8.60E+00 | 12/23 (52%) | Gibberella zeae PH-1 | hypothetical protein FG11359.1 |
| FQ4QJ5301BTZIM | 110 | 4.00E-04 | 21/29 (72%) | Gibberella zeae PH-1 | hypothetical protein FG11396.1 |
| FQ92HJ001DMX4P | 75 | 4.90E+00 | 17/37 (45%) | Gibberella zeae PH-1 | hypothetical protein FG11451.1 |
| FQ4QJ5301CR9Q2 | 285 | 2.00E-24 | 55/84 (65%) | Gibberella zeae PH-1 | hypothetical protein FG11596.1 |
| Contig6097 | 107 | 3.00E-22 | 52/52 (100%) | Gibberella zeae PH-1 | hypothetical protein FG11597.1 |
| Contig1 | 51.2 | 1.00E-11 | 21/46 (45%) | Gibberella zeae PH-1 | hypothetical protein FG11602.1 |
| Contig7040 | 95.5 | 1.00E-18 | 41/63 (65%) | Gibberella zeae PH-1 | hypothetical protein FG11602.1 |
| FQ92HJ001BF3IW | 355 | 2.00E-32 | 65/78 (83%) | Gibberella zeae PH-1 | hypothetical protein FG11603.1 |
| Contig5957 | 145 | 7.00E-34 | 69/88 (78%) | Gibberella zeae PH-1 | hypothetical protein FG11617.1 |
| FQ4QJ5301CX66U | 169 | 6.00E-11 | 33/56 (58%) | Gibberella zeae PH-1 | hypothetical protein FG11625.1 |
| Contig15506 | 132 | 8.00E-30 | 67/117 (57%) | Gibberella zeae PH-1 | hypothetical protein FG11628.1 |
| Contig9226 | 94.4 | 2.00E-18 | 39/67 (58%) | Gibberella zeae PH-1 | hypothetical protein FG11632.1 |
| FQ4QJ5301AJ7U3 | 188 | 7.00E-20 | 32/34 (94%) | Gibberella zeae PH-1 | hypothetical protein FG11632.1 |
| FQ4QJ5301A59OK | 333 | 6.00E-30 | 58/81 (71%) | Gibberella zeae PH-1 | hypothetical protein FG11637.1 |
| Contig15051 | 194 | 2.00E-48 | 91/106 (85%) | Gibberella zeae PH-1 | KPYK\_TRIRE Pyruvate kinase |
| Contig3249 | 76.6 | 5.00E-13 | 38/47 (80%) | Gibberella zeae PH-1 | RL16\_NEUCR 60S ribosomal protein L16 |
| Contig3039 | 158 | 1.00E-37 | 82/98 (83%) | Gibberella zeae PH-1 | RL17\_NEUCR 60S ribosomal protein L17 |
| Contig3217 | 124 | 2.00E-27 | 57/77 (74%) | Gibberella zeae PH-1 | RL2A\_ERYGR 60S ribosomal protein L27a (L29) |
| FQ4QJ5301ENLO4 | 424 | 2.00E-40 | 78/84 (92%) | Gibberella zeae PH-1 | RL3\_NEUCR 60S ribosomal protein L3 |
| Contig16640 | 214 | 2.00E-54 | 101/106 (95%) | Gibberella zeae PH-1 | RL44\_PICJA 60S RIBOSOMAL PROTEIN L44 (L41) |
| Contig5190 | 314 | 4.00E-84 | 149/183 (81%) | Gibberella zeae PH-1 | RL5\_NEUCR 60S ribosomal protein L5 (CPR4) |
| FQ4QJ5301DMFOK | 354 | 2.00E-32 | 69/83 (83%) | Gibberella zeae PH-1 | RLA0\_NEUCR 60S acidic ribosomal protein P0 |
| FQ4QJ5301AYWBH | 371 | 2.00E-34 | 72/77 (93%) | Gibberella zeae PH-1 | RS13\_XENLA 40S RIBOSOMAL PROTEIN S13 |
| Contig9520 | 122 | 6.00E-27 | 61/62 (98%) | Gibberella zeae PH-1 | RS14\_NEUCR 40S ribosomal protein S14 (CRP2) |
| Contig810 | 155 | 7.00E-37 | 76/87 (87%) | Gibberella zeae PH-1 | RS21\_NEUCR 40S ribosomal protein S21 (CRP7) |
| Contig8094 | 177 | 2.00E-43 | 88/94 (93%) | Gibberella zeae PH-1 | RS7\_NEUCR 40S ribosomal protein S7 |
| Contig1108 | 165 | 1.00E-39 | 85/85 (100%) | Gibberella zeae PH-1 | RS9\_PODAN 40S ribosomal protein S9 (S7) |
| FQ4QJ5301B47EM | 162 | 4.00E-10 | 31/33 (93%) | Gibberella zeae PH-1 | RS9\_PODAN 40S ribosomal protein S9 (S7) |
| Contig15452 | 233 | 4.00E-60 | 111/123 (90%) | Gibberella zeae PH-1 | SAR1\_TRIRE GTP-binding protein SAR1 |
| FQ4QJ5301CZ1QT | 359 | 6.00E-33 | 71/87 (81%) | Hypocrea jecorina | hexokinase |
| FQ4QJ5301AO2LR | 320 | 2.00E-28 | 60/81 (74%) | Hypocrea jecorina | hexose transporter-like protein |
| FQ4QJ5301DICRU | 342 | 5.00E-31 | 64/80 (80%) | Hypocrea jecorina | hexose transporter-like protein |
| FQ4QJ5301A2WH1 | 325 | 5.00E-29 | 63/76 (82%) | Hypocrea jecorina | isocitrate lyase |
| FQ92HJ001C0D7F | 189 | 3.00E-13 | 35/57 (61%) | Hypocrea jecorina | NADH-ubiquinone oxidoreductase chain 3 |
| FQ4QJ5301CIXNJ | 74 | 6.50E+00 | 20/67 (29%) | Hypocrea jecorina | NADH-ubiquinone oxidoreductase chain 6 |
| FQ92HJ001EQQ01 | 157 | 2.00E-09 | 34/55 (61%) | Hypocrea jecorina | NADH-ubiquinone oxidoreductase chain 6 |
| FQ92HJ001CUU70 | 241 | 3.00E-19 | 49/91 (53%) | Hypocrea jecorina | NADP-dependent glycerol dehydrogenase |
| FQ4QJ5301DPOBT | 81 | 1.00E+00 | 16/21 (76%) | Hypocrea jecorina | serine/threonine kinase IREI |
| FQ92HJ001BXAGY | 92 | 5.40E-02 | 21/73 (28%) | Kluyveromyces lactis | CBF5\_KLULA |
| Contig13868 | 39.3 | 2.50E-01 | 47/183 (25%) | Kluyveromyces lactis | COX1 intron 3 ORF |
| Contig630 | 32.7 | 8.60E+00 | 12/41 (29%) | Kluyveromyces lactis | unnamed protein product |
| Contig697 | 33.1 | 6.50E+00 | 15/32 (46%) | Kluyveromyces lactis | unnamed protein product |
| Contig2134 | 35.4 | 1.30E+00 | 18/55 (32%) | Kluyveromyces lactis | unnamed protein product |
| Contig3144 | 34.3 | 2.90E+00 | 32/108 (29%) | Kluyveromyces lactis | unnamed protein product |
| Contig6170 | 35 | 4.70E+00 | 16/54 (29%) | Kluyveromyces lactis | unnamed protein product |
| Contig7416 | 44.7 | 2.00E-03 | 27/88 (30%) | Kluyveromyces lactis | unnamed protein product |
| Contig9793 | 34.7 | 2.20E+00 | 16/40 (40%) | Kluyveromyces lactis | unnamed protein product |
| Contig10556 | 35.8 | 1.00E+00 | 20/65 (30%) | Kluyveromyces lactis | unnamed protein product |
| Contig11812 | 35.4 | 1.30E+00 | 18/53 (33%) | Kluyveromyces lactis | unnamed protein product |
| Contig13418 | 33.5 | 8.10E+00 | 17/57 (29%) | Kluyveromyces lactis | unnamed protein product |
| Contig14395 | 32.7 | 8.40E+00 | 15/42 (35%) | Kluyveromyces lactis | unnamed protein product |
| Contig15104 | 33.5 | 4.90E+00 | 16/55 (29%) | Kluyveromyces lactis | unnamed protein product |
| FQ4QJ5301BI8XJ | 75 | 5.10E+00 | 15/38 (39%) | Kluyveromyces lactis | unnamed protein product |
| FQ92HJ001BE75M | 161 | 5.00E-10 | 32/57 (56%) | Kluyveromyces lactis | unnamed protein product |
| FQ92HJ001B6NQR | 74 | 6.60E+00 | 19/64 (29%) | Kluyveromyces lactis | unnamed protein product |
| FQ92HJ001BYM3A | 76 | 3.80E+00 | 16/48 (33%) | Kluyveromyces lactis | unnamed protein product |
| FQ92HJ001DDBA6 | 107 | 1.00E-03 | 30/58 (51%) | Kluyveromyces lactis | unnamed protein product |
| FQ92HJ001DRRTL | 77 | 2.90E+00 | 17/47 (36%) | Kluyveromyces lactis | unnamed protein product |
| FQ92HJ001CJNI6 | 179 | 4.00E-12 | 31/73 (42%) | Kluyveromyces lactis | unnamed protein product |
| FQ92HJ001CRW7L | 125 | 8.00E-06 | 23/27 (85%) | Kluyveromyces lactis | unnamed protein product |
| FQ92HJ001AUYQP | 75 | 5.10E+00 | 17/71 (23%) | Kluyveromyces lactis | unnamed protein product |
| FQ92HJ001C9920 | 77 | 3.00E+00 | 20/51 (39%) | Kluyveromyces lactis | unnamed protein product |
| FQ92HJ001DKS5U | 77 | 3.00E+00 | 21/51 (41%) | Kluyveromyces lactis | unnamed protein product |
| FQ92HJ001EZXN0 | 79 | 1.70E+00 | 19/57 (33%) | Kluyveromyces lactis | unnamed protein product |
| FQ92HJ001C2YKM | 81 | 1.00E+00 | 17/45 (37%) | Kluyveromyces lactis | unnamed protein product |
| FQ4QJ5301DWYTK | 77 | 2.90E+00 | 18/36 (50%) | Laccaria bicolor | oligosaccharyl transferase delta subunit |
| FQ92HJ001BZ120 | 217 | 2.00E-16 | 40/73 (54%) | Laccaria bicolor S238N-H82 | cytosine-purine permease |
| Contig15774 | 46.2 | 8.00E-04 | 29/106 (27%) | Laccaria bicolor S238N-H82 | glutaredoxin |
| FQ4QJ5301EG9MS | 74 | 6.50E+00 | 19/49 (38%) | Laccaria bicolor S238N-H82 | GPI-anchored small secreted protein |
| FQ92HJ001C9P4K | 74 | 6.60E+00 | 23/71 (32%) | Laccaria bicolor S238N-H82 | LisH motif-containing protein |
| FQ92HJ001BU9XN | 218 | 1.00E-16 | 39/48 (81%) | Laccaria bicolor S238N-H82 | Mn superoxide dismutase |
| Contig426 | 32.7 | 8.40E+00 | 17/42 (40%) | Laccaria bicolor S238N-H82 | predicted protein |
| Contig493 | 78.2 | 2.00E-13 | 36/54 (66%) | Laccaria bicolor S238N-H82 | predicted protein |
| Contig2355 | 32.7 | 8.40E+00 | 19/58 (32%) | Laccaria bicolor S238N-H82 | predicted protein |
| Contig2731 | 49.3 | 9.00E-05 | 25/49 (51%) | Laccaria bicolor S238N-H82 | predicted protein |
| Contig3236 | 33.5 | 5.00E+00 | 18/56 (32%) | Laccaria bicolor S238N-H82 | predicted protein |
| Contig6133 | 38.5 | 1.60E-01 | 22/60 (36%) | Laccaria bicolor S238N-H82 | predicted protein |
| Contig7617 | 35 | 5.80E+00 | 15/33 (45%) | Laccaria bicolor S238N-H82 | predicted protein |
| Contig8651 | 33.5 | 5.00E+00 | 13/33 (39%) | Laccaria bicolor S238N-H82 | predicted protein |
| Contig9074 | 34.3 | 3.00E+00 | 15/45 (33%) | Laccaria bicolor S238N-H82 | predicted protein |
| Contig10871 | 110 | 4.00E-23 | 48/60 (80%) | Laccaria bicolor S238N-H82 | predicted protein |
| Contig12407 | 33.5 | 5.10E+00 | 19/58 (32%) | Laccaria bicolor S238N-H82 | predicted protein |
| Contig12467 | 32.7 | 8.40E+00 | 15/39 (38%) | Laccaria bicolor S238N-H82 | predicted protein |
| Contig13705 | 33.9 | 3.80E+00 | 16/47 (34%) | Laccaria bicolor S238N-H82 | predicted protein |
| Contig14270 | 33.1 | 6.50E+00 | 14/41 (34%) | Laccaria bicolor S238N-H82 | predicted protein |
| Contig14338 | 32.7 | 8.60E+00 | 17/47 (36%) | Laccaria bicolor S238N-H82 | predicted protein |
| Contig14343 | 36.2 | 7.60E-01 | 29/78 (37%) | Laccaria bicolor S238N-H82 | predicted protein |
| Contig15182 | 33.1 | 6.50E+00 | 15/33 (45%) | Laccaria bicolor S238N-H82 | predicted protein |
| Contig16482 | 33.9 | 9.30E+00 | 15/49 (30%) | Laccaria bicolor S238N-H82 | predicted protein |
| FQ4QJ5301D51FZ | 75 | 4.90E+00 | 24/81 (29%) | Laccaria bicolor S238N-H82 | predicted protein |
| FQ4QJ5301COMO8 | 77 | 2.90E+00 | 17/45 (37%) | Laccaria bicolor S238N-H82 | predicted protein |
| FQ4QJ5301EBK2Z | 81 | 1.00E+00 | 22/61 (36%) | Laccaria bicolor S238N-H82 | predicted protein |
| FQ4QJ5301BJXH7 | 75 | 5.00E+00 | 18/54 (33%) | Laccaria bicolor S238N-H82 | predicted protein |
| FQ4QJ5301AJKIV | 79 | 1.70E+00 | 15/39 (38%) | Laccaria bicolor S238N-H82 | predicted protein |
| FQ4QJ5301CDP6Z | 78 | 2.30E+00 | 18/47 (38%) | Laccaria bicolor S238N-H82 | predicted protein |
| FQ4QJ5301CQ7CT | 75 | 4.90E+00 | 15/37 (40%) | Laccaria bicolor S238N-H82 | predicted protein |
| FQ92HJ001BBIPM | 77 | 2.90E+00 | 18/56 (32%) | Laccaria bicolor S238N-H82 | predicted protein |
| FQ92HJ001CD9YV | 75 | 5.00E+00 | 19/43 (44%) | Laccaria bicolor S238N-H82 | predicted protein |
| FQ92HJ001EJHUQ | 97 | 1.10E-02 | 15/19 (78%) | Laccaria bicolor S238N-H82 | predicted protein |
| FQ92HJ001ELFOB | 74 | 6.50E+00 | 27/72 (37%) | Laccaria bicolor S238N-H82 | predicted protein |
| FQ92HJ001DB70O | 81 | 9.90E-01 | 18/47 (38%) | Laccaria bicolor S238N-H82 | predicted protein |
| FQ92HJ001CHVQ7 | 76 | 3.90E+00 | 16/37 (43%) | Laccaria bicolor S238N-H82 | predicted protein |
| FQ92HJ001B8ULI | 275 | 8.00E-25 | 53/62 (85%) | Laccaria bicolor S238N-H82 | predicted protein |
| FQ92HJ001D70CH | 207 | 2.00E-15 | 37/62 (59%) | Laccaria bicolor S238N-H82 | predicted protein |
| FQ92HJ001ASQW6 | 145 | 4.00E-08 | 30/35 (85%) | Laccaria bicolor S238N-H82 | predicted protein |
| FQ92HJ001EDPL6 | 74 | 6.50E+00 | 16/45 (35%) | Laccaria bicolor S238N-H82 | predicted protein |
| FQ92HJ001BSZBL | 126 | 6.00E-06 | 20/42 (47%) | Laccaria bicolor S238N-H82 | predicted protein |
| FQ92HJ001D5G62 | 81 | 1.00E+00 | 14/51 (27%) | Laccaria bicolor S238N-H82 | predicted protein |
| FQ92HJ001D18FG | 73 | 8.70E+00 | 12/25 (48%) | Laccaria bicolor S238N-H82 | predicted protein |
| FQ92HJ001B66TX | 76 | 3.90E+00 | 21/62 (33%) | Laccaria bicolor S238N-H82 | predicted protein |
| FQ92HJ001ESUTQ | 206 | 3.00E-15 | 40/49 (81%) | Laccaria bicolor S238N-H82 | predicted protein |
| FQ92HJ001AZB5K | 73 | 8.60E+00 | 14/23 (60%) | Laccaria bicolor S238N-H82 | predicted protein |
| FQ92HJ001CPE8B | 185 | 9.00E-13 | 37/50 (74%) | Laccaria bicolor S238N-H82 | predicted protein |
| FQ92HJ001DVA2Y | 106 | 1.00E-03 | 23/46 (50%) | Laccaria bicolor S238N-H82 | predicted protein |
| FQ92HJ001D2ZZT | 76 | 3.90E+00 | 15/35 (42%) | Laccaria bicolor S238N-H82 | predicted protein |
| FQ92HJ001EGMYV | 74 | 6.50E+00 | 19/59 (32%) | Laccaria bicolor S238N-H82 | predicted protein |
| FQ92HJ001BZ5VU | 193 | 1.00E-13 | 38/67 (56%) | Laccaria bicolor S238N-H82 | predicted protein |
| FQ92HJ001BO8Q6 | 77 | 2.90E+00 | 19/48 (39%) | Laccaria bicolor S238N-H82 | predicted protein |
| FQ4QJ5301CGJE0 | 73 | 8.60E+00 | 23/69 (33%) | Laccaria bicolor S238N-H82 | proline-rich protein |
| FQ92HJ001DFNBC | 77 | 3.00E+00 | 22/57 (38%) | Laccaria bicolor S238N-H82 | proline-rich protein |
| FQ4QJ5301C820P | 74 | 6.60E+00 | 16/37 (43%) | Lodderomyces elongisporus | isopentenyl-diphosphate delta-isomerase |
| FQ4QJ5301EWRVR | 88 | 1.50E-01 | 23/50 (46%) | Lodderomyces elongisporus | serine/threonine-protein kinase nrc-2 |
| FQ92HJ001AFTRV | 76 | 3.80E+00 | 17/57 (29%) | Lodderomyces elongisporus NRRL | alpha-1,2-mannosyltransferase ALG11 |
| Contig5234 | 34.3 | 2.90E+00 | 15/57 (26%) | Lodderomyces elongisporus NRRL | conserved hypothetical protein |
| Contig10306 | 33.1 | 6.50E+00 | 23/56 (41%) | Lodderomyces elongisporus NRRL | conserved hypothetical protein |
| Contig11465 | 33.5 | 5.00E+00 | 16/35 (45%) | Lodderomyces elongisporus NRRL | conserved hypothetical protein |
| Contig13116 | 36.6 | 6.00E-01 | 18/40 (45%) | Lodderomyces elongisporus NRRL | conserved hypothetical protein |
| Contig13834 | 33.1 | 6.50E+00 | 17/37 (45%) | Lodderomyces elongisporus NRRL | conserved hypothetical protein |
| FQ4QJ5301CQZBN | 80 | 1.30E+00 | 15/29 (51%) | Lodderomyces elongisporus NRRL | conserved hypothetical protein |
| FQ92HJ001DVGJL | 75 | 5.00E+00 | 20/60 (33%) | Lodderomyces elongisporus NRRL | conserved hypothetical protein |
| FQ92HJ001DQ8V0 | 74 | 6.40E+00 | 13/33 (39%) | Lodderomyces elongisporus NRRL | conserved hypothetical protein |
| FQ4QJ5301AWR6W | 81 | 1.00E+00 | 22/58 (37%) | Lodderomyces elongisporus NRRL | hypothetical protein LELG\_01274 |
| Contig10366 | 35 | 4.90E+00 | 20/61 (32%) | Lodderomyces elongisporus NRRL | hypothetical protein LELG\_03033 |
| FQ4QJ5301BB1Q2 | 73 | 8.50E+00 | 15/51 (29%) | Lodderomyces elongisporus NRRL | hypothetical protein LELG\_03386 |
| FQ4QJ5301BQHV8 | 73 | 8.70E+00 | 14/41 (34%) | Lodderomyces elongisporus NRRL | hypothetical protein LELG\_03446 |
| FQ4QJ5301BAUKK | 76 | 3.80E+00 | 16/39 (41%) | Lodderomyces elongisporus NRRL | hypothetical protein LELG\_03602 |
| Contig13326 | 32.7 | 8.60E+00 | 14/34 (41%) | Lodderomyces elongisporus NRRL | hypothetical protein LELG\_03867 |
| Contig3537 | 39.3 | 9.10E-02 | 26/95 (27%) | Lodderomyces elongisporus NRRL | hypothetical protein LELG\_04203 |
| FQ92HJ001DDXOP | 76 | 3.80E+00 | 21/56 (37%) | Lodderomyces elongisporus NRRL | hypothetical protein LELG\_04802 |
| Contig9699 | 33.5 | 4.90E+00 | 21/80 (26%) | Lodderomyces elongisporus NRRL | hypothetical protein LELG\_05029 |
| FQ92HJ001B0SH2 | 88 | 1.60E-01 | 22/71 (30%) | Lodderomyces elongisporus NRRL | hypothetical protein LELG\_05451 |
| Contig9280 | 32.7 | 8.40E+00 | 12/28 (42%) | Lodderomyces elongisporus NRRL | hypothetical protein LELG\_05710 |
| FQ92HJ001EI360 | 313 | 1.00E-27 | 59/72 (81%) | Lodderomyces elongisporus NRRL YB-4239 | 60S ribosomal protein L2 |
| Contig5620 | 33.9 | 3.80E+00 | 12/36 (33%) | Lodderomyces elongisporus NRRL YB-4239 | predicted protein |
| Contig16025 | 34.3 | 3.00E+00 | 16/42 (38%) | Lodderomyces elongisporus NRRL YB-4239 | predicted protein |
| FQ4QJ5301EVMJ7 | 74 | 6.60E+00 | 14/36 (38%) | Lodderomyces elongisporus NRRL YB-4239 | predicted protein |
| FQ4QJ5301BYS5F | 101 | 5.00E-03 | 23/90 (25%) | Lodderomyces elongisporus NRRL YB-4239 | predicted protein |
| FQ92HJ001D2CKY | 80 | 1.30E+00 | 12/31 (38%) | Lodderomyces elongisporus NRRL YB-4239 | predicted protein |
| FQ92HJ001CSOMV | 84 | 4.50E-01 | 23/99 (23%) | Lodderomyces elongisporus NRRL YB-4239 | predicted protein |
| FQ92HJ001EFM00 | 75 | 5.10E+00 | 19/47 (40%) | Lodderomyces elongisporus NRRL YB-4239 | predicted protein |
| FQ92HJ001CGK15 | 73 | 8.60E+00 | 24/73 (32%) | Lodderomyces elongisporus NRRL YB-4239 | RNA exonuclease 3 |
| FQ4QJ5301AWJB7 | 281 | 7.00E-24 | 58/85 (68%) | Magnaporthe grisea | elicitor protein |
| Contig16704 | 320 | 5.00E-86 | 166/179 (92%) | Magnaporthe grisea | heat shock protein 60, mitochondrial precursor |
| FQ4QJ5301D7C99 | 344 | 3.00E-31 | 62/68 (91%) | Magnaporthe grisea | NADH-ubiquinone oxidoreductase-like protein |
| FQ92HJ001EJ9ON | 241 | 3.00E-19 | 42/52 (80%) | Magnaporthe grisea | oxalate decarboxylase-like protein |
| Contig13310 | 46.2 | 8.00E-04 | 20/33 (60%) | Magnaporthe grisea | peroxisomal hydratase-dehydrogenase-epimerase |
| Contig14967 | 43.9 | 7.00E-03 | 37/114 (32%) | Magnaporthe grisea | putative transposase |
| Contig4919 | 172 | 5.00E-42 | 82/82 (100%) | Magnaporthe grisea 70-15 | 40S ribosomal protein S27 |
| Contig11163 | 221 | 1.00E-56 | 109/113 (96%) | Magnaporthe grisea 70-15 | 40S ribosomal protein S3 |
| Contig7720 | 33.9 | 3.90E+00 | 14/16 (87%) | Magnaporthe grisea 70-15 | ADP,ATP carrier protein |
| Contig5203 | 93.6 | 4.00E-18 | 46/53 (86%) | Magnaporthe grisea 70-15 | ATP synthase beta chain, mitochondrial |
| FQ4QJ5301B45U4 | 219 | 1.00E-16 | 44/52 (84%) | Magnaporthe grisea 70-15 | ATP synthase beta chain, mitochondrial |
| Contig1855 | 102 | 9.00E-21 | 52/89 (58%) | Magnaporthe grisea 70-15 | cofilin, putative |
| Contig376 | 146 | 4.00E-34 | 69/79 (87%) | Magnaporthe grisea 70-15 | conserved hypothetical protein |
| Contig899 | 149 | 8.00E-35 | 68/81 (83%) | Magnaporthe grisea 70-15 | conserved hypothetical protein |
| Contig2141 | 83.6 | 4.00E-15 | 36/44 (81%) | Magnaporthe grisea 70-15 | conserved hypothetical protein |
| Contig2547 | 41.2 | 2.40E-02 | 18/18 (100%) | Magnaporthe grisea 70-15 | conserved hypothetical protein |
| Contig2884 | 156 | 5.00E-37 | 72/83 (86%) | Magnaporthe grisea 70-15 | conserved hypothetical protein |
| Contig5147 | 67.8 | 2.00E-10 | 35/54 (64%) | Magnaporthe grisea 70-15 | conserved hypothetical protein |
| Contig6731 | 177 | 3.00E-43 | 81/91 (89%) | Magnaporthe grisea 70-15 | conserved hypothetical protein |
| Contig8023 | 127 | 2.00E-28 | 58/72 (80%) | Magnaporthe grisea 70-15 | conserved hypothetical protein |
| Contig8404 | 52 | 1.00E-05 | 30/68 (44%) | Magnaporthe grisea 70-15 | conserved hypothetical protein |
| Contig11736 | 64.7 | 2.00E-09 | 30/44 (68%) | Magnaporthe grisea 70-15 | conserved hypothetical protein |
| Contig12450 | 160 | 2.00E-38 | 77/77 (100%) | Magnaporthe grisea 70-15 | conserved hypothetical protein |
| Contig13477 | 72 | 1.00E-11 | 33/41 (80%) | Magnaporthe grisea 70-15 | conserved hypothetical protein |
| Contig14184 | 97.4 | 3.00E-19 | 44/49 (89%) | Magnaporthe grisea 70-15 | conserved hypothetical protein |
| Contig15512 | 80.5 | 4.00E-14 | 36/41 (87%) | Magnaporthe grisea 70-15 | conserved hypothetical protein |
| Contig15603 | 115 | 1.00E-24 | 61/88 (69%) | Magnaporthe grisea 70-15 | conserved hypothetical protein |
| FQ4QJ5301CHD3L | 327 | 3.00E-29 | 66/79 (83%) | Magnaporthe grisea 70-15 | conserved hypothetical protein |
| FQ4QJ5301C0TM4 | 399 | 1.00E-37 | 76/81 (93%) | Magnaporthe grisea 70-15 | conserved hypothetical protein |
| FQ4QJ5301DDJFS | 342 | 5.00E-31 | 61/81 (75%) | Magnaporthe grisea 70-15 | conserved hypothetical protein |
| FQ4QJ5301CXJVH | 98 | 1.10E-02 | 21/31 (67%) | Magnaporthe grisea 70-15 | conserved hypothetical protein |
| FQ4QJ5301A68OB | 360 | 4.00E-33 | 66/81 (81%) | Magnaporthe grisea 70-15 | conserved hypothetical protein |
| FQ4QJ5301DYUAK | 303 | 2.00E-26 | 52/64 (81%) | Magnaporthe grisea 70-15 | conserved hypothetical protein |
| FQ4QJ5301ANTO9 | 279 | 1.00E-23 | 53/66 (80%) | Magnaporthe grisea 70-15 | conserved hypothetical protein |
| FQ4QJ5301E169Q | 87 | 2.10E-01 | 17/17 (100%) | Magnaporthe grisea 70-15 | conserved hypothetical protein |
| FQ4QJ5301EUBVU | 245 | 1.00E-25 | 44/59 (74%) | Magnaporthe grisea 70-15 | conserved hypothetical protein |
| FQ4QJ5301AH8NS | 166 | 1.00E-10 | 30/43 (69%) | Magnaporthe grisea 70-15 | conserved hypothetical protein |
| FQ4QJ5301CG2I2 | 248 | 4.00E-20 | 55/83 (66%) | Magnaporthe grisea 70-15 | conserved hypothetical protein |
| FQ92HJ001BL31H | 182 | 2.00E-12 | 36/50 (72%) | Magnaporthe grisea 70-15 | conserved hypothetical protein |
| FQ92HJ001CLNW0 | 141 | 1.00E-07 | 28/34 (82%) | Magnaporthe grisea 70-15 | conserved hypothetical protein |
| FQ92HJ001ERVF5 | 356 | 1.00E-32 | 64/72 (88%) | Magnaporthe grisea 70-15 | conserved hypothetical protein |
| FQ4QJ5301A1QSK | 360 | 4.00E-33 | 70/83 (84%) | Magnaporthe grisea 70-15 | enolase |
| FQ4QJ5301BYJC0 | 98 | 9.00E-03 | 19/21 (90%) | Magnaporthe grisea 70-15 | ferrochelatase precursor |
| Contig1782 | 107 | 3.00E-22 | 49/52 (94%) | Magnaporthe grisea 70-15 | GTP-binding protein SAR1 |
| Contig14103 | 92.8 | 7.00E-18 | 44/46 (95%) | Magnaporthe grisea 70-15 | histone H2B |
| FQ4QJ5301BF94R | 97 | 1.40E-02 | 19/35 (54%) | Magnaporthe grisea 70-15 | hypothetical protein MGCH7\_ch7g1076 |
| Contig9640 | 42.7 | 8.00E-03 | 22/59 (37%) | Magnaporthe grisea 70-15 | hypothetical protein MGG\_00105 |
| FQ4QJ5301EROB7 | 141 | 1.00E-07 | 36/78 (46%) | Magnaporthe grisea 70-15 | hypothetical protein MGG\_00124 |
| Contig6149 | 40.4 | 4.80E-02 | 25/72 (34%) | Magnaporthe grisea 70-15 | hypothetical protein MGG\_00157 |
| Contig2814 | 68.6 | 1.00E-10 | 32/59 (54%) | Magnaporthe grisea 70-15 | hypothetical protein MGG\_00207 |
| Contig12215 | 48.1 | 2.00E-04 | 20/28 (71%) | Magnaporthe grisea 70-15 | hypothetical protein MGG\_00449 |
| Contig3700 | 117 | 3.00E-25 | 55/59 (93%) | Magnaporthe grisea 70-15 | hypothetical protein MGG\_00470 |
| Contig13890 | 57.8 | 2.00E-07 | 26/36 (72%) | Magnaporthe grisea 70-15 | hypothetical protein MGG\_00476 |
| FQ92HJ001BEA5M | 321 | 1.00E-28 | 54/76 (71%) | Magnaporthe grisea 70-15 | hypothetical protein MGG\_00530 |
| Contig3692 | 63.2 | 6.00E-09 | 32/80 (40%) | Magnaporthe grisea 70-15 | hypothetical protein MGG\_00687 |
| FQ92HJ001DNL47 | 158 | 9.00E-10 | 32/32 (100%) | Magnaporthe grisea 70-15 | hypothetical protein MGG\_00774 |
| FQ4QJ5301EKD8O | 334 | 5.00E-30 | 59/71 (83%) | Magnaporthe grisea 70-15 | hypothetical protein MGG\_00919 |
| FQ92HJ001BQV89 | 286 | 2.00E-24 | 44/72 (61%) | Magnaporthe grisea 70-15 | hypothetical protein MGG\_00984 |
| FQ92HJ001C3UA6 | 137 | 3.00E-07 | 29/68 (42%) | Magnaporthe grisea 70-15 | hypothetical protein MGG\_00984 |
| Contig15490 | 34.7 | 1.00E-02 | 14/20 (70%) | Magnaporthe grisea 70-15 | hypothetical protein MGG\_00987 |
| FQ4QJ5301ENY6M | 115 | 1.00E-04 | 36/73 (49%) | Magnaporthe grisea 70-15 | hypothetical protein MGG\_01081 |
| FQ4QJ5301BEX49 | 338 | 2.00E-30 | 65/70 (92%) | Magnaporthe grisea 70-15 | hypothetical protein MGG\_01092 |
| Contig7880 | 112 | 1.00E-23 | 54/84 (64%) | Magnaporthe grisea 70-15 | hypothetical protein MGG\_01198 |
| Contig3612 | 182 | 5.00E-45 | 86/88 (97%) | Magnaporthe grisea 70-15 | hypothetical protein MGG\_01236 |
| FQ4QJ5301BEDDR | 73 | 8.70E+00 | 15/38 (39%) | Magnaporthe grisea 70-15 | hypothetical protein MGG\_01325 |
| Contig1104 | 171 | 2.00E-41 | 87/137 (63%) | Magnaporthe grisea 70-15 | hypothetical protein MGG\_01490 |
| FQ92HJ001DEISL | 96 | 1.80E-02 | 16/23 (69%) | Magnaporthe grisea 70-15 | hypothetical protein MGG\_01524 |
| FQ4QJ5301CPH8Z | 324 | 7.00E-29 | 58/74 (78%) | Magnaporthe grisea 70-15 | hypothetical protein MGG\_01607 |
| FQ4QJ5301D00EG | 216 | 2.00E-16 | 37/50 (74%) | Magnaporthe grisea 70-15 | hypothetical protein MGG\_01667 |
| Contig187 | 167 | 2.00E-40 | 82/115 (71%) | Magnaporthe grisea 70-15 | hypothetical protein MGG\_01710 |
| FQ4QJ5301C2783 | 197 | 4.00E-14 | 46/90 (51%) | Magnaporthe grisea 70-15 | hypothetical protein MGG\_01722 |
| Contig6564 | 34.7 | 2.20E+00 | 16/37 (43%) | Magnaporthe grisea 70-15 | hypothetical protein MGG\_01844 |
| Contig3285 | 92.8 | 7.00E-18 | 44/67 (65%) | Magnaporthe grisea 70-15 | hypothetical protein MGG\_01978 |
| FQ92HJ001DJC11 | 74 | 6.40E+00 | 14/18 (77%) | Magnaporthe grisea 70-15 | hypothetical protein MGG\_02391 |
| Contig269 | 32.7 | 8.40E+00 | 14/28 (50%) | Magnaporthe grisea 70-15 | hypothetical protein MGG\_02505 |
| FQ92HJ001EQLD2 | 77 | 2.90E+00 | 14/19 (73%) | Magnaporthe grisea 70-15 | hypothetical protein MGG\_02630 |
| FQ92HJ001DRY9B | 120 | 3.00E-05 | 26/77 (33%) | Magnaporthe grisea 70-15 | hypothetical protein MGG\_02705 |
| Contig15325 | 112 | 8.00E-24 | 51/54 (94%) | Magnaporthe grisea 70-15 | hypothetical protein MGG\_02711 |
| FQ4QJ5301CDHDK | 291 | 4.00E-25 | 58/82 (70%) | Magnaporthe grisea 70-15 | hypothetical protein MGG\_02719 |
| FQ92HJ001BFPT9 | 233 | 2.00E-18 | 45/82 (54%) | Magnaporthe grisea 70-15 | hypothetical protein MGG\_02941 |
| Contig3271 | 71.2 | 2.00E-11 | 34/76 (44%) | Magnaporthe grisea 70-15 | hypothetical protein MGG\_02972 |
| Contig597 | 82.8 | 7.00E-15 | 38/45 (84%) | Magnaporthe grisea 70-15 | hypothetical protein MGG\_02980 |
| Contig12077 | 119 | 7.00E-26 | 46/55 (83%) | Magnaporthe grisea 70-15 | hypothetical protein MGG\_02983 |
| FQ4QJ5301C9OEZ | 92 | 5.40E-02 | 18/20 (90%) | Magnaporthe grisea 70-15 | hypothetical protein MGG\_03051 |
| FQ92HJ001B4HD9 | 178 | 6.00E-12 | 34/60 (56%) | Magnaporthe grisea 70-15 | hypothetical protein MGG\_03174 |
| FQ4QJ5301D71S8 | 214 | 4.00E-16 | 40/45 (88%) | Magnaporthe grisea 70-15 | hypothetical protein MGG\_03211 |
| FQ92HJ001A86ZK | 166 | 1.00E-10 | 30/53 (56%) | Magnaporthe grisea 70-15 | hypothetical protein MGG\_03267 |
| FQ4QJ5301D4AET | 74 | 6.60E+00 | 14/46 (30%) | Magnaporthe grisea 70-15 | hypothetical protein MGG\_03291 |
| FQ4QJ5301BJXNX | 73 | 8.50E+00 | 16/56 (28%) | Magnaporthe grisea 70-15 | hypothetical protein MGG\_03487 |
| Contig3523 | 121 | 1.00E-26 | 58/83 (69%) | Magnaporthe grisea 70-15 | hypothetical protein MGG\_03512 |
| FQ4QJ5301EY6T6 | 239 | 5.00E-21 | 44/64 (68%) | Magnaporthe grisea 70-15 | hypothetical protein MGG\_03537 |
| FQ4QJ5301CYCQ2 | 129 | 3.00E-06 | 28/36 (77%) | Magnaporthe grisea 70-15 | hypothetical protein MGG\_03564 |
| Contig3135 | 72.4 | 1.00E-11 | 41/67 (61%) | Magnaporthe grisea 70-15 | hypothetical protein MGG\_03568 |
| FQ92HJ001BXJH0 | 109 | 6.00E-04 | 20/24 (83%) | Magnaporthe grisea 70-15 | hypothetical protein MGG\_03572 |
| FQ4QJ5301EQAEW | 348 | 1.00E-31 | 59/83 (71%) | Magnaporthe grisea 70-15 | hypothetical protein MGG\_03628 |
| Contig2600 | 124 | 2.00E-27 | 57/71 (80%) | Magnaporthe grisea 70-15 | hypothetical protein MGG\_03662 |
| Contig2322 | 65.5 | 1.00E-09 | 39/89 (43%) | Magnaporthe grisea 70-15 | hypothetical protein MGG\_03663 |
| Contig15134 | 115 | 2.00E-24 | 51/71 (71%) | Magnaporthe grisea 70-15 | hypothetical protein MGG\_03930 |
| FQ92HJ001C1JYN | 174 | 2.00E-11 | 34/67 (50%) | Magnaporthe grisea 70-15 | hypothetical protein MGG\_04082 |
| FQ4QJ5301CHQY3 | 94 | 3.10E-02 | 21/35 (60%) | Magnaporthe grisea 70-15 | hypothetical protein MGG\_04090 |
| FQ92HJ001D0A2H | 169 | 6.00E-11 | 37/86 (43%) | Magnaporthe grisea 70-15 | hypothetical protein MGG\_04112 |
| Contig4046 | 57 | 4.00E-07 | 28/76 (36%) | Magnaporthe grisea 70-15 | hypothetical protein MGG\_04115 |
| Contig5563 | 56.6 | 6.00E-07 | 31/85 (36%) | Magnaporthe grisea 70-15 | hypothetical protein MGG\_04119 |
| FQ92HJ001DSSCP | 70 | 5.00E-03 | 11/22 (50%) | Magnaporthe grisea 70-15 | hypothetical protein MGG\_04194 |
| FQ4QJ5301DE20E | 146 | 3.00E-08 | 26/45 (57%) | Magnaporthe grisea 70-15 | hypothetical protein MGG\_04302 |
| FQ4QJ5301EU4MR | 99 | 8.00E-03 | 19/29 (65%) | Magnaporthe grisea 70-15 | hypothetical protein MGG\_04363 |
| FQ92HJ001BPY5R | 227 | 1.00E-17 | 46/61 (75%) | Magnaporthe grisea 70-15 | hypothetical protein MGG\_04454 |
| FQ4QJ5301AUGN8 | 120 | 3.00E-05 | 30/61 (49%) | Magnaporthe grisea 70-15 | hypothetical protein MGG\_04459 |
| FQ4QJ5301DQ5OP | 217 | 2.00E-16 | 43/70 (61%) | Magnaporthe grisea 70-15 | hypothetical protein MGG\_04470 |
| FQ92HJ001EPKH6 | 106 | 1.00E-03 | 22/26 (84%) | Magnaporthe grisea 70-15 | hypothetical protein MGG\_04470 |
| FQ92HJ001DGJSF | 189 | 3.00E-13 | 36/45 (80%) | Magnaporthe grisea 70-15 | hypothetical protein MGG\_04641 |
| FQ92HJ001ANISH | 280 | 8.00E-24 | 53/65 (81%) | Magnaporthe grisea 70-15 | hypothetical protein MGG\_04705 |
| FQ4QJ5301CFQT3 | 145 | 4.00E-08 | 36/74 (48%) | Magnaporthe grisea 70-15 | hypothetical protein MGG\_04756 |
| FQ92HJ001DQ5SR | 195 | 6.00E-14 | 33/42 (78%) | Magnaporthe grisea 70-15 | hypothetical protein MGG\_04759 |
| Contig953 | 140 | 4.00E-32 | 62/81 (76%) | Magnaporthe grisea 70-15 | hypothetical protein MGG\_04837 |
| FQ92HJ001D5RQC | 136 | 4.00E-07 | 27/46 (58%) | Magnaporthe grisea 70-15 | hypothetical protein MGG\_04942 |
| FQ4QJ5301DBTP4 | 190 | 2.00E-13 | 36/41 (87%) | Magnaporthe grisea 70-15 | hypothetical protein MGG\_04967 |
| Contig15412 | 86.7 | 5.00E-16 | 44/68 (64%) | Magnaporthe grisea 70-15 | hypothetical protein MGG\_05073 |
| Contig9843 | 40.8 | 3.10E-02 | 29/104 (27%) | Magnaporthe grisea 70-15 | hypothetical protein MGG\_05152 |
| FQ4QJ5301BY6SM | 246 | 9.00E-24 | 49/60 (81%) | Magnaporthe grisea 70-15 | hypothetical protein MGG\_05265 |
| Contig14770 | 33.1 | 6.60E+00 | 17/50 (34%) | Magnaporthe grisea 70-15 | hypothetical protein MGG\_05292 |
| FQ4QJ5301CWL82 | 74 | 6.50E+00 | 17/51 (33%) | Magnaporthe grisea 70-15 | hypothetical protein MGG\_05312 |
| FQ4QJ5301BE3EM | 175 | 1.00E-11 | 45/89 (50%) | Magnaporthe grisea 70-15 | hypothetical protein MGG\_05755 |
| FQ4QJ5301AQWMJ | 75 | 5.10E+00 | 15/28 (53%) | Magnaporthe grisea 70-15 | hypothetical protein MGG\_05817 |
| FQ92HJ001EAAEH | 97 | 1.40E-02 | 24/77 (31%) | Magnaporthe grisea 70-15 | hypothetical protein MGG\_06029 |
| Contig15822 | 124 | 2.00E-27 | 59/74 (79%) | Magnaporthe grisea 70-15 | hypothetical protein MGG\_06035 |
| FQ4QJ5301CAIYO | 114 | 1.00E-04 | 23/41 (56%) | Magnaporthe grisea 70-15 | hypothetical protein MGG\_06132 |
| FQ4QJ5301DZT13 | 246 | 7.00E-20 | 46/68 (67%) | Magnaporthe grisea 70-15 | hypothetical protein MGG\_06177 |
| FQ4QJ5301CS45Q | 364 | 2.00E-33 | 66/72 (91%) | Magnaporthe grisea 70-15 | hypothetical protein MGG\_06296 |
| FQ92HJ001BFBQL | 159 | 9.00E-10 | 30/38 (78%) | Magnaporthe grisea 70-15 | hypothetical protein MGG\_06328 |
| FQ4QJ5301D4PIX | 222 | 5.00E-17 | 42/63 (66%) | Magnaporthe grisea 70-15 | hypothetical protein MGG\_06336 |
| FQ4QJ5301BZ8GZ | 138 | 3.00E-07 | 37/68 (54%) | Magnaporthe grisea 70-15 | hypothetical protein MGG\_06403 |
| FQ92HJ001E6HW9 | 277 | 2.00E-23 | 52/65 (80%) | Magnaporthe grisea 70-15 | hypothetical protein MGG\_06405 |
| FQ4QJ5301AFSC2 | 122 | 2.00E-05 | 21/30 (70%) | Magnaporthe grisea 70-15 | hypothetical protein MGG\_06429 |
| FQ4QJ5301BDRZY | 147 | 1.00E-10 | 22/26 (84%) | Magnaporthe grisea 70-15 | hypothetical protein MGG\_06458 |
| FQ4QJ5301DEEHI | 139 | 2.00E-07 | 27/45 (60%) | Magnaporthe grisea 70-15 | hypothetical protein MGG\_06519 |
| FQ4QJ5301EUWGA | 366 | 9.00E-34 | 64/73 (87%) | Magnaporthe grisea 70-15 | hypothetical protein MGG\_06569 |
| Contig3001 | 92 | 2.00E-31 | 41/44 (93%) | Magnaporthe grisea 70-15 | hypothetical protein MGG\_06712 |
| FQ4QJ5301DLJIA | 233 | 2.00E-18 | 51/80 (63%) | Magnaporthe grisea 70-15 | hypothetical protein MGG\_06733 |
| Contig2706 | 40.4 | 4.10E-02 | 21/52 (40%) | Magnaporthe grisea 70-15 | hypothetical protein MGG\_06866 |
| FQ4QJ5301BLQB6 | 216 | 1.00E-20 | 44/61 (72%) | Magnaporthe grisea 70-15 | hypothetical protein MGG\_06868 |
| Contig3108 | 105 | 1.00E-21 | 44/81 (54%) | Magnaporthe grisea 70-15 | hypothetical protein MGG\_06922 |
| Contig1807 | 67.4 | 3.00E-10 | 35/75 (46%) | Magnaporthe grisea 70-15 | hypothetical protein MGG\_06973 |
| FQ4QJ5301DC11J | 423 | 2.00E-40 | 78/80 (97%) | Magnaporthe grisea 70-15 | hypothetical protein MGG\_07000 |
| FQ92HJ001BO6PQ | 300 | 4.00E-26 | 57/66 (86%) | Magnaporthe grisea 70-15 | hypothetical protein MGG\_07074 |
| FQ4QJ5301DP6U4 | 130 | 8.00E-07 | 26/39 (66%) | Magnaporthe grisea 70-15 | hypothetical protein MGG\_07089 |
| FQ92HJ001EWCQO | 95 | 2.40E-02 | 21/53 (39%) | Magnaporthe grisea 70-15 | hypothetical protein MGG\_07127 |
| FQ4QJ5301EKVRV | 212 | 5.00E-23 | 38/49 (77%) | Magnaporthe grisea 70-15 | hypothetical protein MGG\_07148 |
| FQ92HJ001A29GI | 83 | 5.80E-01 | 18/40 (45%) | Magnaporthe grisea 70-15 | hypothetical protein MGG\_07175 |
| Contig6721 | 37 | 4.60E-01 | 14/32 (43%) | Magnaporthe grisea 70-15 | hypothetical protein MGG\_07208 |
| Contig5364 | 67.4 | 8.00E-26 | 30/32 (93%) | Magnaporthe grisea 70-15 | hypothetical protein MGG\_07224 |
| Contig9409 | 61.2 | 2.00E-08 | 28/39 (71%) | Magnaporthe grisea 70-15 | hypothetical protein MGG\_07268 |
| FQ4QJ5301C3WS3 | 245 | 1.00E-19 | 51/78 (65%) | Magnaporthe grisea 70-15 | hypothetical protein MGG\_07317 |
| FQ92HJ001AOOOA | 78 | 2.20E+00 | 15/15 (100%) | Magnaporthe grisea 70-15 | hypothetical protein MGG\_07317 |
| Contig4574 | 34.3 | 2.90E+00 | 24/74 (32%) | Magnaporthe grisea 70-15 | hypothetical protein MGG\_07334 |
| FQ4QJ5301BDQXK | 282 | 5.00E-24 | 52/72 (72%) | Magnaporthe grisea 70-15 | hypothetical protein MGG\_07434 |
| FQ4QJ5301AIXNQ | 115 | 1.00E-04 | 23/46 (50%) | Magnaporthe grisea 70-15 | hypothetical protein MGG\_07475 |
| FQ4QJ5301CVHZ3 | 78 | 2.20E+00 | 13/29 (44%) | Magnaporthe grisea 70-15 | hypothetical protein MGG\_07614 |
| Contig15580 | 114 | 2.00E-24 | 59/94 (62%) | Magnaporthe grisea 70-15 | hypothetical protein MGG\_07712 |
| FQ4QJ5301EZI5L | 383 | 9.00E-36 | 72/82 (87%) | Magnaporthe grisea 70-15 | hypothetical protein MGG\_07756 |
| FQ4QJ5301CH1GE | 134 | 7.00E-07 | 19/36 (52%) | Magnaporthe grisea 70-15 | hypothetical protein MGG\_07843 |
| FQ4QJ5301EM3W1 | 187 | 5.00E-13 | 31/44 (70%) | Magnaporthe grisea 70-15 | hypothetical protein MGG\_08010 |
| FQ4QJ5301BRCWN | 139 | 2.00E-07 | 26/35 (74%) | Magnaporthe grisea 70-15 | hypothetical protein MGG\_08143 |
| Contig2805 | 76.3 | 7.00E-13 | 37/80 (46%) | Magnaporthe grisea 70-15 | hypothetical protein MGG\_08175 |
| FQ4QJ5301ED243 | 137 | 3.00E-07 | 25/39 (64%) | Magnaporthe grisea 70-15 | hypothetical protein MGG\_08457 |
| FQ4QJ5301DNZM6 | 247 | 6.00E-20 | 43/67 (64%) | Magnaporthe grisea 70-15 | hypothetical protein MGG\_08597 |
| Contig9730 | 68.2 | 2.00E-10 | 31/41 (75%) | Magnaporthe grisea 70-15 | hypothetical protein MGG\_08808 |
| FQ4QJ5301C9HVR | 284 | 3.00E-24 | 50/75 (66%) | Magnaporthe grisea 70-15 | hypothetical protein MGG\_08929 |
| FQ4QJ5301A6XGW | 272 | 7.00E-23 | 47/76 (61%) | Magnaporthe grisea 70-15 | hypothetical protein MGG\_08929 |
| Contig8644 | 160 | 2.00E-38 | 80/97 (82%) | Magnaporthe grisea 70-15 | hypothetical protein MGG\_09367 |
| Contig4451 | 59.7 | 7.00E-08 | 30/66 (45%) | Magnaporthe grisea 70-15 | hypothetical protein MGG\_09369 |
| Contig12977 | 80.1 | 5.00E-14 | 41/95 (43%) | Magnaporthe grisea 70-15 | hypothetical protein MGG\_09372 |
| FQ92HJ001BRSGP | 77 | 2.90E+00 | 18/41 (43%) | Magnaporthe grisea 70-15 | hypothetical protein MGG\_09414 |
| FQ4QJ5301AISL6 | 185 | 9.00E-13 | 31/43 (72%) | Magnaporthe grisea 70-15 | hypothetical protein MGG\_09465 |
| FQ4QJ5301DG1XX | 90 | 9.20E-02 | 20/40 (50%) | Magnaporthe grisea 70-15 | hypothetical protein MGG\_09473 |
| FQ92HJ001ENOPX | 73 | 8.50E+00 | 15/41 (36%) | Magnaporthe grisea 70-15 | hypothetical protein MGG\_09523 |
| Contig5734 | 104 | 2.00E-21 | 49/73 (67%) | Magnaporthe grisea 70-15 | hypothetical protein MGG\_09541 |
| FQ4QJ5301CSAGI | 177 | 7.00E-12 | 44/94 (46%) | Magnaporthe grisea 70-15 | hypothetical protein MGG\_09559 |
| FQ4QJ5301DOJQD | 158 | 9.00E-16 | 31/36 (86%) | Magnaporthe grisea 70-15 | hypothetical protein MGG\_09942 |
| Contig77 | 82 | 1.00E-14 | 39/73 (53%) | Magnaporthe grisea 70-15 | hypothetical protein MGG\_10005 |
| FQ4QJ5301AJJ5S | 75 | 5.00E+00 | 16/23 (69%) | Magnaporthe grisea 70-15 | hypothetical protein MGG\_10104 |
| FQ4QJ5301DOWKG | 270 | 1.00E-22 | 51/61 (83%) | Magnaporthe grisea 70-15 | hypothetical protein MGG\_10149 |
| FQ4QJ5301CAN34 | 435 | 9.00E-42 | 85/86 (98%) | Magnaporthe grisea 70-15 | hypothetical protein MGG\_10370 |
| FQ92HJ001B3D1W | 300 | 4.00E-26 | 61/93 (65%) | Magnaporthe grisea 70-15 | hypothetical protein MGG\_10412 |
| Contig7062 | 45.1 | 2.00E-03 | 39/138 (28%) | Magnaporthe grisea 70-15 | hypothetical protein MGG\_10590 |
| FQ92HJ001BWAMK | 97 | 1.40E-02 | 18/38 (47%) | Magnaporthe grisea 70-15 | hypothetical protein MGG\_10604 |
| FQ4QJ5301EHM41 | 341 | 7.00E-31 | 60/86 (69%) | Magnaporthe grisea 70-15 | hypothetical protein MGG\_10606 |
| Contig7235 | 102 | 7.00E-21 | 48/64 (75%) | Magnaporthe grisea 70-15 | hypothetical protein MGG\_10668 |
| FQ92HJ001BWOD5 | 111 | 3.00E-04 | 20/25 (80%) | Magnaporthe grisea 70-15 | hypothetical protein MGG\_10691 |
| FQ4QJ5301BBX0S | 224 | 3.00E-17 | 47/80 (58%) | Magnaporthe grisea 70-15 | hypothetical protein MGG\_10832 |
| FQ92HJ001CAZBK | 74 | 6.60E+00 | 12/29 (41%) | Magnaporthe grisea 70-15 | hypothetical protein MGG\_10878 |
| FQ92HJ001CXJUB | 144 | 5.00E-08 | 25/38 (65%) | Magnaporthe grisea 70-15 | hypothetical protein MGG\_11028 |
| FQ4QJ5301BD3BC | 109 | 4.00E-06 | 19/27 (70%) | Magnaporthe grisea 70-15 | hypothetical protein MGG\_11354 |
| Contig10647 | 70.1 | 5.00E-11 | 37/58 (63%) | Magnaporthe grisea 70-15 | hypothetical protein MGG\_11474 |
| Contig5586 | 33.5 | 5.00E+00 | 25/80 (31%) | Magnaporthe grisea 70-15 | hypothetical protein MGG\_12059 |
| FQ4QJ5301C9XMD | 188 | 4.00E-13 | 40/44 (90%) | Magnaporthe grisea 70-15 | hypothetical protein MGG\_12322 |
| FQ92HJ001AY6K9 | 284 | 3.00E-24 | 57/77 (74%) | Magnaporthe grisea 70-15 | hypothetical protein MGG\_12336 |
| FQ4QJ5301EB1Y6 | 165 | 2.00E-10 | 36/73 (49%) | Magnaporthe grisea 70-15 | hypothetical protein MGG\_12344 |
| Contig6847 | 35.4 | 1.30E+00 | 17/53 (32%) | Magnaporthe grisea 70-15 | hypothetical protein MGG\_12807 |
| FQ4QJ5301B065L | 278 | 1.00E-23 | 55/76 (72%) | Magnaporthe grisea 70-15 | hypothetical protein MGG\_12868 |
| Contig4420 | 43.9 | 4.00E-03 | 22/66 (33%) | Magnaporthe grisea 70-15 | hypothetical protein MGG\_13065 |
| FQ4QJ5301DUR23 | 202 | 9.00E-15 | 41/77 (53%) | Magnaporthe grisea 70-15 | hypothetical protein MGG\_13115 |
| FQ92HJ001EVC6E | 131 | 2.00E-06 | 34/64 (53%) | Magnaporthe grisea 70-15 | hypothetical protein MGG\_13161 |
| FQ4QJ5301A2HC3 | 305 | 1.00E-26 | 59/82 (71%) | Magnaporthe grisea 70-15 | hypothetical protein MGG\_13192 |
| FQ4QJ5301CMDO8 | 191 | 2.00E-13 | 41/77 (53%) | Magnaporthe grisea 70-15 | hypothetical protein MGG\_13807 |
| FQ92HJ001CLFMI | 165 | 2.00E-10 | 32/66 (48%) | Magnaporthe grisea 70-15 | hypothetical protein MGG\_13956 |
| Contig5224 | 40.8 | 3.10E-02 | 18/33 (54%) | Magnaporthe grisea 70-15 | hypothetical protein MGG\_14031 |
| Contig6446 | 46.2 | 7.00E-04 | 22/48 (45%) | Magnaporthe grisea 70-15 | hypothetical protein MGG\_14421 |
| FQ4QJ5301C3VR8 | 101 | 5.00E-03 | 19/62 (30%) | Magnaporthe grisea 70-15 | hypothetical protein MGG\_14421 |
| FQ4QJ5301B7D9X | 213 | 5.00E-16 | 41/61 (67%) | Magnaporthe grisea 70-15 | hypothetical protein MGG\_14431 |
| FQ4QJ5301BP6QY | 149 | 1.00E-08 | 27/30 (90%) | Magnaporthe grisea 70-15 | inorganic pyrophosphatase |
| FQ92HJ001DXT3Z | 292 | 3.00E-25 | 54/56 (96%) | Magnaporthe grisea 70-15 | large subunit ribosomal protein L3 |
| Contig9655 | 87.4 | 1.00E-29 | 36/46 (78%) | Magnaporthe grisea 70-15 | ornithine decarboxylase |
| Contig352 | 42.7 | 8.00E-03 | 29/85 (34%) | Magnaporthe grisea 70-15 | predicted protein |
| Contig1306 | 68.2 | 2.00E-12 | 33/52 (63%) | Magnaporthe grisea 70-15 | predicted protein |
| Contig2014 | 33.5 | 5.10E+00 | 18/57 (31%) | Magnaporthe grisea 70-15 | predicted protein |
| Contig14790 | 46.2 | 7.00E-04 | 18/30 (60%) | Magnaporthe grisea 70-15 | predicted protein |
| Contig15387 | 43.1 | 6.00E-03 | 18/37 (48%) | Magnaporthe grisea 70-15 | predicted protein |
| FQ4QJ5301CB2BP | 235 | 1.00E-18 | 41/69 (59%) | Magnaporthe grisea 70-15 | predicted protein |
| FQ4QJ5301DBCNW | 147 | 2.00E-08 | 32/55 (58%) | Magnaporthe grisea 70-15 | predicted protein |
| FQ4QJ5301CAYE2 | 119 | 4.00E-05 | 23/36 (63%) | Magnaporthe grisea 70-15 | predicted protein |
| FQ92HJ001DT77V | 172 | 3.00E-11 | 33/53 (62%) | Magnaporthe grisea 70-15 | predicted protein |
| FQ92HJ001E0ZVR | 248 | 4.00E-20 | 47/81 (58%) | Magnaporthe grisea 70-15 | predicted protein |
| FQ92HJ001CINMI | 242 | 2.00E-19 | 47/67 (70%) | Magnaporthe grisea 70-15 | predicted protein |
| FQ92HJ001B072R | 115 | 1.00E-04 | 29/80 (36%) | Magnaporthe grisea 70-15 | predicted protein |
| Contig15527 | 104 | 2.00E-21 | 47/51 (92%) | Magnaporthe grisea 70-15 | ribosomal protein L39 |
| Contig12322 | 35 | 1.70E+00 | 25/76 (32%) | Malassezia globosa CBS 7966 | hypothetical protein MGL\_0026 |
| Contig13319 | 34.3 | 2.90E+00 | 17/39 (43%) | Malassezia globosa CBS 7966 | hypothetical protein MGL\_0291 |
| FQ92HJ001DWKT3 | 75 | 4.90E+00 | 18/52 (34%) | Malassezia globosa CBS 7966 | hypothetical protein MGL\_0390 |
| FQ92HJ001DR9QY | 83 | 5.80E-01 | 15/30 (50%) | Malassezia globosa CBS 7966 | hypothetical protein MGL\_0862 |
| FQ92HJ001DYGPM | 73 | 8.70E+00 | 21/51 (41%) | Malassezia globosa CBS 7966 | hypothetical protein MGL\_1132 |
| FQ92HJ001BKFM9 | 77 | 2.90E+00 | 18/45 (40%) | Malassezia globosa CBS 7966 | hypothetical protein MGL\_1492 |
| Contig12889 | 33.9 | 3.80E+00 | 21/54 (38%) | Malassezia globosa CBS 7966 | hypothetical protein MGL\_1552 |
| FQ4QJ5301BXA8H | 75 | 5.00E+00 | 17/56 (30%) | Malassezia globosa CBS 7966 | hypothetical protein MGL\_1666 |
| FQ92HJ001B8CQR | 73 | 8.50E+00 | 17/42 (40%) | Malassezia globosa CBS 7966 | hypothetical protein MGL\_1743 |
| FQ92HJ001CHDHU | 79 | 1.70E+00 | 18/50 (36%) | Malassezia globosa CBS 7966 | hypothetical protein MGL\_1822 |
| Contig9874 | 33.1 | 6.50E+00 | 14/32 (43%) | Malassezia globosa CBS 7966 | hypothetical protein MGL\_2132 |
| FQ92HJ001C5BNF | 140 | 1.00E-07 | 28/36 (77%) | Malassezia globosa CBS 7966 | hypothetical protein MGL\_2682 |
| Contig13433 | 116 | 6.00E-25 | 57/74 (77%) | Malassezia globosa CBS 7966 | hypothetical protein MGL\_3591 |
| FQ4QJ5301C8FJX | 115 | 1.00E-04 | 26/65 (40%) | Malassezia globosa CBS 7966 | hypothetical protein MGL\_3838 |
| Contig15386 | 34.7 | 2.30E+00 | 17/45 (37%) | Malassezia globosa CBS 7966 | hypothetical protein MGL\_3923 |
| Contig8613 | 33.1 | 6.50E+00 | 23/73 (31%) | Malassezia globosa CBS 7966 | hypothetical protein MGL\_4139 |
| Contig14599 | 35 | 2.40E+00 | 28/93 (30%) | Malawimonas jakobiformis | ABC transporter channel subunit |
| Contig11065 | 34.3 | 2.90E+00 | 17/46 (36%) | Malawimonas jakobiformis | orf120 |
| FQ92HJ001BOCXF | 287 | 1.00E-24 | 54/66 (81%) | Metarhizium anisopliae | cytochrome oxidase subunit III |
| Contig1536 | 81.6 | 2.00E-14 | 37/40 (92%) | Metarhizium anisopliae | glyceraldehyde-3-phosphate dehydrogenase |
| Contig6730 | 263 | 2.00E-69 | 130/137 (94%) | Metarhizium anisopliae | glyceraldehyde-3-phosphate dehydrogenase |
| FQ92HJ001D248J | 449 | 2.00E-43 | 80/83 (96%) | Metarhizium anisopliae | G-protein beta subunit |
| Contig5900 | 899 | 0.00E+00 | 468/600 (78%) | Metarhizium anisopliae | heat shock protein 90 |
| Contig7396 | 159 | 6.00E-38 | 85/98 (86%) | Metarhizium anisopliae | heat shock protein 90 |
| FQ92HJ001AQ63P | 367 | 7.00E-34 | 71/84 (84%) | Metarhizium anisopliae | NADH dehydrogenase subunit 5 |
| Contig4054 | 168 | 1.00E-40 | 82/85 (96%) | Metarhizium anisopliae | translation elongation factor 1 alpha |
| FQ92HJ001BJOA0 | 78 | 2.30E+00 | 17/55 (30%) | Metarhizium anisopliae | AF291909\_2 recQ family helicase |
| FQ92HJ001AYP8R | 388 | 3.00E-36 | 67/78 (85%) | Metarhizium anisopliae var. anisopliae | neutral trehalase |
| Contig11027 | 53.5 | 5.00E-06 | 28/50 (56%) | Nectria haematococca | putative transposase |
| FQ4QJ5301EU1YE | 201 | 1.00E-14 | 40/71 (56%) | Nectria haematococca | putative transposase |
| FQ4QJ5301DZCL4 | 186 | 3.00E-20 | 35/55 (63%) | Nectria haematococca | putative transposase |
| FQ4QJ5301DRPS3 | 78 | 2.20E+00 | 17/39 (43%) | Nectria haematococca | putative transposase |
| FQ4QJ5301AV2QC | 156 | 2.00E-09 | 33/66 (50%) | Nectria haematococca | putative transposase |
| FQ92HJ001BYTGQ | 153 | 4.00E-09 | 41/80 (51%) | Nectria haematococca | putative transposase |
| FQ92HJ001DAMKR | 205 | 4.00E-15 | 41/70 (58%) | Nectria haematococca | putative transposase |
| FQ92HJ001DZ2OH | 135 | 5.00E-07 | 29/47 (61%) | Nectria haematococca | putative transposase |
| Contig3649 | 62.4 | 1.00E-08 | 33/67 (49%) | Nectria haematococca | AF315315\_2 DNA transposase |
| Contig5344 | 31.2 | 5.10E-02 | 15/37 (40%) | Nectria haematococca | AF315315\_2 DNA transposase |
| Contig6402 | 57.4 | 3.00E-07 | 38/136 (27%) | Nectria haematococca | AF315315\_2 DNA transposase |
| Contig10012 | 77.4 | 3.00E-13 | 40/86 (46%) | Nectria haematococca | AF315315\_2 DNA transposase |
| Contig14808 | 57 | 4.00E-07 | 33/89 (37%) | Nectria haematococca | AF315315\_2 DNA transposase |
| FQ92HJ001C7AWE | 109 | 2.00E-06 | 23/42 (54%) | Nectria haematococca | AF315315\_4 restless-like transposase |
| Contig10282 | 78.6 | 1.00E-13 | 42/82 (51%) | Nectria haematococca mpVI | beta-(1, 3)-D-glucan synthase |
| FQ4QJ5301DJ0WY | 324 | 7.00E-29 | 67/79 (84%) | Neosartorya | antigenic mitochondrial protein HSP60, putative |
| FQ4QJ5301D3H0X | 108 | 7.00E-04 | 21/51 (41%) | Neosartorya | GPI-anchored cell wall beta-1,3-endoglucanase EglC |
| FQ4QJ5301EPWBX | 400 | 1.00E-37 | 71/87 (81%) | Neosartorya | peptidyl prolyl cis-trans isomerase (CypC), putative |
| FQ4QJ5301DZTYK | 75 | 5.00E+00 | 19/45 (42%) | Neosartorya | phospholipid metabolism enzyme regulator, putative |
| FQ92HJ001DUW5G | 74 | 6.60E+00 | 26/98 (26%) | Neosartorya fischeri | glycosyl transferase, group 2 family protein |
| FQ4QJ5301BWNI5 | 75 | 5.10E+00 | 16/37 (43%) | Neosartorya fischeri | mitochondrial inheritance component mdm12 |
| FQ4QJ5301CFJ4M | 154 | 4.00E-12 | 30/46 (65%) | Neosartorya fischeri | mitochondrial ribosomal protein L11, putative |
| Contig467 | 49.7 | 7.00E-05 | 22/60 (36%) | Neosartorya fischeri | phosphatidylinositolglycan class N, putative |
| FQ4QJ5301D9BJD | 237 | 8.00E-19 | 42/60 (70%) | Neosartorya fischeri NRRL | 1,3-beta-glucanosyltransferase, putative |
| Contig3196 | 101 | 1.00E-20 | 47/67 (70%) | Neosartorya fischeri NRRL | nonribosomal peptide synthase, putative |
| FQ4QJ5301EECJ4 | 73 | 8.50E+00 | 15/30 (50%) | Neosartorya fischeri NRRL | pre-mRNA splicing helicase, putative |
| FQ4QJ5301BCBY6 | 88 | 1.50E-01 | 16/18 (88%) | Neosartorya fischeri NRRL | RNA splicing factor (Pad-1), putative |
| FQ4QJ5301EK2CV | 133 | 2.00E-13 | 24/26 (92%) | Neosartorya fischeri NRRL | UbiA-like prenyltransferase, putative |
| FQ4QJ5301AHXUV | 78 | 2.30E+00 | 21/53 (39%) | Neosartorya fischeri NRRL 181 | C6 finger domain protein, putative |
| Contig2572 | 104 | 2.00E-21 | 51/54 (94%) | Neosartorya fischeri NRRL 181 | calcium sensor (NCS-1), putative |
| Contig6542 | 92 | 1.00E-18 | 39/50 (78%) | Neosartorya fischeri NRRL 181 | conserved hypothetical protein |
| Contig8383 | 34.3 | 2.90E+00 | 17/37 (45%) | Neosartorya fischeri NRRL 181 | conserved hypothetical protein |
| FQ4QJ5301BSFPA | 75 | 5.00E+00 | 16/34 (47%) | Neosartorya fischeri NRRL 181 | conserved hypothetical protein |
| FQ92HJ001EOYX7 | 92 | 5.30E-02 | 23/67 (34%) | Neosartorya fischeri NRRL 181 | conserved hypothetical protein |
| FQ4QJ5301AIV32 | 97 | 1.40E-02 | 18/21 (85%) | Neosartorya fischeri NRRL 181 | FAD binding domain protein |
| FQ4QJ5301A89PW | 204 | 5.00E-15 | 39/78 (50%) | Neosartorya fischeri NRRL 181 | FAD binding domain protein |
| Contig15112 | 54.3 | 3.00E-06 | 25/28 (89%) | Neosartorya fischeri NRRL 181 | hypothetical protein NFIA\_043490 |
| FQ92HJ001D5EN5 | 86 | 2.70E-01 | 18/25 (72%) | Neosartorya fischeri NRRL 181 | hypothetical protein NFIA\_043490 |
| FQ92HJ001D2ZUL | 191 | 2.00E-13 | 36/50 (72%) | Neosartorya fischeri NRRL 181 | hypothetical protein NFIA\_061330 |
| FQ4QJ5301B0H02 | 106 | 1.00E-03 | 21/32 (65%) | Neosartorya fischeri NRRL 181 | hypothetical protein NFIA\_063990 |
| FQ92HJ001BUU9L | 73 | 8.50E+00 | 17/27 (62%) | Neosartorya fischeri NRRL 181 | hypothetical protein NFIA\_070270 |
| FQ92HJ001BLN58 | 78 | 2.30E+00 | 19/49 (38%) | Neosartorya fischeri NRRL 181 | hypothetical protein NFIA\_108520 |
| Contig2425 | 82.8 | 7.00E-15 | 39/61 (63%) | Neosartorya fischeri NRRL 181 | predicted protein |
| FQ92HJ001COPZ7 | 318 | 3.00E-28 | 59/85 (69%) | Neosartorya fischeri NRRL 181 | predicted protein |
| Contig14047 | 42.4 | 1.10E-02 | 19/26 (73%) | Neosartorya fischeri NRRL 181 | sucrose transport protein |
| FQ4QJ5301CBZA0 | 403 | 5.00E-38 | 78/82 (95%) | Neurospora | heat shock 70 kDa protein, mitochondrial precursor |
| Contig5237 | 108 | 2.00E-22 | 49/57 (85%) | Neurospora | probable ORNITHINE CARBAMOYLTRANSFERASE PRECURSOR |
| FQ92HJ001AT1Z8 | 231 | 4.00E-18 | 43/46 (93%) | Neurospora crassa | eukaryotic translation initiation factor 3 |
| FQ4QJ5301CY4PO | 356 | 1.00E-32 | 67/82 (81%) | Neurospora crassa | farnesyl pyrophosphate synthetase |
| Contig15514 | 35 | 1.70E+00 | 25/87 (28%) | Neurospora crassa | hypothetical protein |
| FQ4QJ5301DEB6N | 88 | 1.60E-01 | 15/33 (45%) | Neurospora crassa | hypothetical protein |
| FQ92HJ001EB1X4 | 75 | 5.10E+00 | 15/36 (41%) | Neurospora crassa | hypothetical protein |
| FQ4QJ5301BVTGX | 377 | 5.00E-35 | 67/77 (87%) | Neurospora crassa | NADH:ubiquinone oxidoreductase 49kD subunit |
| Contig11254 | 33.1 | 6.60E+00 | 15/61 (24%) | Neurospora crassa | phosphatidylinositol-4-phosphate 5-kinase its3 |
| Contig5282 | 240 | 7.00E-62 | 113/143 (79%) | Neurospora crassa | phospho-2-dehydro-3-deoxyheptonate aldolase |
| FQ4QJ5301CE324 | 96 | 2.00E-05 | 17/26 (65%) | Neurospora crassa | related to DNA repair endonuclease rad2 |
| FQ4QJ5301AMYAH | 211 | 8.00E-16 | 40/53 (75%) | Neurospora crassa | related to MRD1 |
| FQ4QJ5301BYTBH | 85 | 3.40E-01 | 20/57 (35%) | Neurospora crassa | related to spindle assembly checkpoint protein |
| Contig5504 | 155 | 9.00E-37 | 73/85 (85%) | Neurospora crassa | ubiquinone biosynthesis methyltransferase coq5 |
| Contig14875 | 196 | 5.00E-49 | 99/99 (100%) | Neurospora crassa OR74A | 40S ribosomal protein S17 |
| Contig15177 | 67 | 4.00E-10 | 34/42 (80%) | Neurospora crassa OR74A | 40S ribosomal protein S17 |
| Contig4542 | 176 | 6.00E-43 | 86/92 (93%) | Neurospora crassa OR74A | 40S ribosomal protein S22 |
| Contig12582 | 82.8 | 7.00E-15 | 42/43 (97%) | Neurospora crassa OR74A | 40S ribosomal protein S28 |
| Contig16283 | 109 | 7.00E-23 | 48/56 (85%) | Neurospora crassa OR74A | 40S ribosomal protein S29 |
| Contig3767 | 164 | 1.00E-39 | 85/86 (98%) | Neurospora crassa OR74A | 40s ribosomal protein s5 |
| Contig5145 | 158 | 1.00E-37 | 77/83 (92%) | Neurospora crassa OR74A | 40S ribosomal protein S6 |
| Contig11999 | 183 | 1.00E-68 | 92/114 (80%) | Neurospora crassa OR74A | 40S ribosomal protein S8 |
| FQ92HJ001EV3OA | 424 | 2.00E-40 | 84/84 (100%) | Neurospora crassa OR74A | 60S ribosomal protein L11 |
| FQ92HJ001D8P2I | 261 | 1.00E-21 | 53/67 (79%) | Neurospora crassa OR74A | 60S ribosomal protein L11 |
| FQ4QJ5301D1E43 | 218 | 1.00E-16 | 40/50 (80%) | Neurospora crassa OR74A | 60S ribosomal protein L13 |
| FQ92HJ001BV8M3 | 257 | 4.00E-21 | 49/51 (96%) | Neurospora crassa OR74A | 60S ribosomal protein L2 |
| Contig11076 | 111 | 1.00E-23 | 52/54 (96%) | Neurospora crassa OR74A | 60S ribosomal protein L29 |
| FQ4QJ5301A04ZN | 250 | 3.00E-20 | 47/50 (94%) | Neurospora crassa OR74A | 60S ribosomal protein L3 |
| Contig8896 | 317 | 2.00E-85 | 150/167 (89%) | Neurospora crassa OR74A | 60S ribosomal protein L7 |
| FQ4QJ5301A3TJZ | 330 | 1.00E-29 | 62/71 (87%) | Neurospora crassa OR74A | 6-phosphogluconate dehydrogenase |
| FQ4QJ5301DRB6S | 448 | 3.00E-43 | 84/84 (100%) | Neurospora crassa OR74A | AP-1 complex subunit mu |
| FQ4QJ5301ATTV7 | 322 | 1.00E-28 | 63/83 (75%) | Neurospora crassa OR74A | AP-3 complex subunit sigma |
| FQ92HJ001BB5G6 | 326 | 4.00E-29 | 58/72 (80%) | Neurospora crassa OR74A | argininosuccinate lyase |
| FQ4QJ5301EEGZM | 421 | 4.00E-40 | 80/86 (93%) | Neurospora crassa OR74A | ARP2/3 complex 34 kDa subunit |
| FQ4QJ5301E4T0Z | 117 | 7.00E-05 | 25/44 (56%) | Neurospora crassa OR74A | asparaginyl-tRNA synthetase |
| Contig13080 | 151 | 2.00E-35 | 74/99 (74%) | Neurospora crassa OR74A | ATP phosphoribosyltransferase |
| FQ4QJ5301BN5OV | 366 | 9.00E-34 | 70/78 (89%) | Neurospora crassa OR74A | ATP-dependent RNA helicase dbp-2 |
| FQ92HJ001A0YIE | 282 | 5.00E-24 | 53/82 (64%) | Neurospora crassa OR74A | autophagy protein 5 |
| FQ4QJ5301EA63I | 81 | 1.00E+00 | 22/52 (42%) | Neurospora crassa OR74A | conserved hypothetical protein, variant |
| FQ4QJ5301BAM4V | 79 | 1.70E+00 | 21/61 (34%) | Neurospora crassa OR74A | DNA mismatch repair protein msh6 |
| FQ4QJ5301BQ1UC | 150 | 1.00E-08 | 28/50 (56%) | Neurospora crassa OR74A | dolichol-phosphate mannosyltransferase |
| FQ4QJ5301B1SD0 | 294 | 2.00E-25 | 51/55 (92%) | Neurospora crassa OR74A | elongation of fatty acids protein 3 |
| Contig12498 | 88.2 | 2.00E-16 | 38/45 (84%) | Neurospora crassa OR74A | GCY protein |
| Contig3873 | 124 | 2.00E-27 | 60/72 (83%) | Neurospora crassa OR74A | geranylgeranyltransferase beta subunit |
| Contig2205 | 161 | 1.00E-38 | 81/82 (98%) | Neurospora crassa OR74A | glucose-6-phosphate 1-dehydrogenase |
| FQ92HJ001CJFZP | 256 | 5.00E-21 | 45/60 (75%) | Neurospora crassa OR74A | glycogen debranching enzyme |
| FQ4QJ5301DEJHA | 306 | 8.00E-28 | 57/68 (83%) | Neurospora crassa OR74A | GTP-binding protein GUF1 |
| FQ4QJ5301AI9FF | 414 | 2.00E-39 | 76/79 (96%) | Neurospora crassa OR74A | homoserine O-acetyltransferase |
| FQ4QJ5301DO13H | 109 | 6.00E-04 | 23/50 (46%) | Neurospora crassa OR74A | hypothetical protein NCU00092 |
| FQ4QJ5301CGITD | 345 | 2.00E-31 | 63/75 (84%) | Neurospora crassa OR74A | hypothetical protein NCU00333 |
| FQ92HJ001CX7PF | 99 | 8.00E-03 | 22/33 (66%) | Neurospora crassa OR74A | hypothetical protein NCU00527 |
| FQ92HJ001DXF7P | 288 | 1.00E-24 | 53/88 (60%) | Neurospora crassa OR74A | hypothetical protein NCU00586 |
| Contig11727 | 38.9 | 1.20E-01 | 25/89 (28%) | Neurospora crassa OR74A | hypothetical protein NCU00595 |
| Contig12431 | 84.3 | 3.00E-15 | 40/45 (88%) | Neurospora crassa OR74A | hypothetical protein NCU00634 |
| FQ92HJ001EQVJM | 136 | 4.00E-07 | 25/33 (75%) | Neurospora crassa OR74A | hypothetical protein NCU00677 |
| FQ92HJ001EOLR8 | 82 | 7.60E-01 | 23/61 (37%) | Neurospora crassa OR74A | hypothetical protein NCU00723 |
| FQ4QJ5301CUHTI | 353 | 3.00E-32 | 65/80 (81%) | Neurospora crassa OR74A | hypothetical protein NCU00770 |
| FQ92HJ001B0AR2 | 295 | 2.00E-25 | 54/73 (73%) | Neurospora crassa OR74A | hypothetical protein NCU00812 |
| FQ92HJ001CZRJ2 | 182 | 2.00E-12 | 27/46 (58%) | Neurospora crassa OR74A | hypothetical protein NCU00867 |
| FQ4QJ5301AG970 | 154 | 3.00E-09 | 35/81 (43%) | Neurospora crassa OR74A | hypothetical protein NCU01114 |
| Contig15690 | 90.9 | 3.00E-17 | 40/67 (59%) | Neurospora crassa OR74A | hypothetical protein NCU01116 |
| FQ4QJ5301AWS1H | 75 | 5.00E+00 | 15/43 (34%) | Neurospora crassa OR74A | hypothetical protein NCU01272 |
| FQ4QJ5301A0LWR | 175 | 2.00E-12 | 34/60 (56%) | Neurospora crassa OR74A | hypothetical protein NCU01330 |
| Contig4437 | 40 | 5.40E-02 | 18/49 (36%) | Neurospora crassa OR74A | hypothetical protein NCU01344 |
| Contig722 | 49.3 | 9.00E-05 | 23/66 (34%) | Neurospora crassa OR74A | hypothetical protein NCU01425 |
| FQ4QJ5301DQ5ST | 158 | 8.00E-21 | 31/48 (64%) | Neurospora crassa OR74A | hypothetical protein NCU01441 |
| Contig11221 | 33.1 | 6.60E+00 | 16/48 (33%) | Neurospora crassa OR74A | hypothetical protein NCU01531 |
| FQ4QJ5301ATWFP | 97 | 1.40E-02 | 18/25 (72%) | Neurospora crassa OR74A | hypothetical protein NCU01554 |
| FQ92HJ001EXSE2 | 143 | 7.00E-08 | 31/52 (59%) | Neurospora crassa OR74A | hypothetical protein NCU01793 |
| FQ4QJ5301CTVPS | 256 | 5.00E-21 | 44/79 (55%) | Neurospora crassa OR74A | hypothetical protein NCU02031 |
| FQ92HJ001BWI0Z | 257 | 4.00E-21 | 50/62 (80%) | Neurospora crassa OR74A | hypothetical protein NCU02219 |
| FQ4QJ5301ETWOH | 214 | 3.00E-21 | 41/56 (73%) | Neurospora crassa OR74A | hypothetical protein NCU02252 |
| FQ92HJ001AT26X | 91 | 6.90E-02 | 19/58 (32%) | Neurospora crassa OR74A | hypothetical protein NCU02292 |
| FQ4QJ5301CXATA | 245 | 1.00E-19 | 50/85 (58%) | Neurospora crassa OR74A | hypothetical protein NCU02304 |
| FQ4QJ5301AJTPT | 78 | 2.30E+00 | 24/58 (41%) | Neurospora crassa OR74A | hypothetical protein NCU02319 |
| Contig11989 | 140 | 4.00E-32 | 65/76 (85%) | Neurospora crassa OR74A | hypothetical protein NCU02366 |
| Contig3059 | 103 | 5.00E-21 | 57/84 (67%) | Neurospora crassa OR74A | hypothetical protein NCU02402 |
| FQ4QJ5301AV1G3 | 174 | 2.00E-11 | 30/38 (78%) | Neurospora crassa OR74A | hypothetical protein NCU02423 |
| FQ4QJ5301CO1LQ | 171 | 4.00E-11 | 31/37 (83%) | Neurospora crassa OR74A | hypothetical protein NCU02614 |
| FQ4QJ5301DE284 | 249 | 3.00E-20 | 47/79 (59%) | Neurospora crassa OR74A | hypothetical protein NCU02742 |
| FQ4QJ5301EBASJ | 185 | 9.00E-13 | 36/62 (58%) | Neurospora crassa OR74A | hypothetical protein NCU02788 |
| FQ92HJ001EV1C3 | 207 | 3.00E-15 | 49/95 (51%) | Neurospora crassa OR74A | hypothetical protein NCU03020 |
| FQ4QJ5301B3166 | 375 | 8.00E-35 | 73/75 (97%) | Neurospora crassa OR74A | hypothetical protein NCU03051 |
| FQ92HJ001AUY66 | 216 | 2.00E-16 | 45/69 (65%) | Neurospora crassa OR74A | hypothetical protein NCU03113 |
| FQ4QJ5301DU9QG | 97 | 1.40E-02 | 27/71 (38%) | Neurospora crassa OR74A | hypothetical protein NCU03151 |
| FQ4QJ5301DNZU2 | 140 | 1.00E-07 | 30/57 (52%) | Neurospora crassa OR74A | hypothetical protein NCU03235 |
| FQ4QJ5301AICT0 | 317 | 4.00E-28 | 56/67 (83%) | Neurospora crassa OR74A | hypothetical protein NCU03347 |
| Contig14203 | 105 | 8.00E-22 | 55/74 (74%) | Neurospora crassa OR74A | hypothetical protein NCU03405 |
| FQ92HJ001EC14N | 73 | 8.70E+00 | 17/41 (41%) | Neurospora crassa OR74A | hypothetical protein NCU03584 |
| FQ4QJ5301DGBZM | 96 | 1.80E-02 | 23/65 (35%) | Neurospora crassa OR74A | hypothetical protein NCU03819 |
| FQ92HJ001E4SSK | 251 | 4.00E-27 | 44/54 (81%) | Neurospora crassa OR74A | hypothetical protein NCU03838 |
| FQ4QJ5301CJV2F | 323 | 9.00E-29 | 56/81 (69%) | Neurospora crassa OR74A | hypothetical protein NCU03846 |
| FQ92HJ001C3876 | 201 | 1.00E-14 | 48/102 (47%) | Neurospora crassa OR74A | hypothetical protein NCU03906 |
| FQ92HJ001ESST9 | 104 | 2.00E-03 | 18/34 (52%) | Neurospora crassa OR74A | hypothetical protein NCU04003 |
| FQ4QJ5301D80UQ | 171 | 4.00E-11 | 31/46 (67%) | Neurospora crassa OR74A | hypothetical protein NCU04101 |
| Contig12843 | 74.7 | 2.00E-12 | 35/53 (66%) | Neurospora crassa OR74A | hypothetical protein NCU04130 |
| FQ4QJ5301BUTQ7 | 191 | 2.00E-13 | 34/42 (80%) | Neurospora crassa OR74A | hypothetical protein NCU04165 |
| FQ92HJ001ALDSS | 73 | 8.70E+00 | 17/47 (36%) | Neurospora crassa OR74A | hypothetical protein NCU04272 |
| FQ4QJ5301EB4QU | 292 | 3.00E-25 | 58/82 (70%) | Neurospora crassa OR74A | hypothetical protein NCU04400 |
| FQ4QJ5301A6B19 | 240 | 4.00E-19 | 44/68 (64%) | Neurospora crassa OR74A | hypothetical protein NCU04407 |
| FQ4QJ5301AMF50 | 190 | 2.00E-14 | 35/57 (61%) | Neurospora crassa OR74A | hypothetical protein NCU04408 |
| Contig7782 | 92.4 | 6.00E-18 | 46/65 (70%) | Neurospora crassa OR74A | hypothetical protein NCU04411 |
| FQ4QJ5301C8FPM | 392 | 9.00E-37 | 75/92 (81%) | Neurospora crassa OR74A | hypothetical protein NCU04511 |
| Contig3210 | 97.4 | 3.00E-19 | 47/58 (81%) | Neurospora crassa OR74A | hypothetical protein NCU04789 |
| FQ4QJ5301C5OIY | 79 | 1.70E+00 | 19/51 (37%) | Neurospora crassa OR74A | hypothetical protein NCU04852 |
| Contig15440 | 94.7 | 2.00E-18 | 45/84 (53%) | Neurospora crassa OR74A | hypothetical protein NCU04946 |
| FQ92HJ001DQPC6 | 74 | 6.50E+00 | 13/28 (46%) | Neurospora crassa OR74A | hypothetical protein NCU04988 |
| FQ4QJ5301D8UH2 | 162 | 4.00E-10 | 30/48 (62%) | Neurospora crassa OR74A | hypothetical protein NCU05240 |
| FQ4QJ5301BKEG3 | 250 | 3.00E-20 | 43/75 (57%) | Neurospora crassa OR74A | hypothetical protein NCU05242 |
| FQ4QJ5301ALXN0 | 199 | 2.00E-14 | 44/79 (55%) | Neurospora crassa OR74A | hypothetical protein NCU05248 |
| FQ92HJ001CP54J | 76 | 3.80E+00 | 24/64 (37%) | Neurospora crassa OR74A | hypothetical protein NCU05256 |
| FQ4QJ5301CGRDE | 75 | 5.00E+00 | 14/29 (48%) | Neurospora crassa OR74A | hypothetical protein NCU05385 |
| Contig7875 | 52.4 | 1.00E-05 | 24/31 (77%) | Neurospora crassa OR74A | hypothetical protein NCU05512 |
| FQ92HJ001DH7OJ | 78 | 2.20E+00 | 14/32 (43%) | Neurospora crassa OR74A | hypothetical protein NCU05527 |
| FQ4QJ5301D4XGB | 268 | 2.00E-22 | 52/77 (67%) | Neurospora crassa OR74A | hypothetical protein NCU05634 |
| FQ4QJ5301CJDR0 | 176 | 1.00E-11 | 33/55 (60%) | Neurospora crassa OR74A | hypothetical protein NCU05777 |
| FQ4QJ5301CIV7V | 78 | 2.20E+00 | 20/46 (43%) | Neurospora crassa OR74A | hypothetical protein NCU05789 |
| FQ4QJ5301CT3XQ | 265 | 5.00E-22 | 52/85 (61%) | Neurospora crassa OR74A | hypothetical protein NCU05984 |
| FQ4QJ5301EWM1D | 73 | 8.60E+00 | 21/71 (29%) | Neurospora crassa OR74A | hypothetical protein NCU06057 |
| Contig9713 | 124 | 2.00E-27 | 61/92 (66%) | Neurospora crassa OR74A | hypothetical protein NCU06113 |
| FQ4QJ5301BSLWV | 211 | 9.00E-16 | 45/84 (53%) | Neurospora crassa OR74A | hypothetical protein NCU06210 |
| FQ4QJ5301CFJN7 | 97 | 1.00E-04 | 17/25 (68%) | Neurospora crassa OR74A | hypothetical protein NCU06433 |
| Contig4384 | 126 | 4.00E-28 | 58/79 (73%) | Neurospora crassa OR74A | hypothetical protein NCU06436 |
| FQ4QJ5301ELFCV | 216 | 2.00E-16 | 35/47 (74%) | Neurospora crassa OR74A | hypothetical protein NCU06672 |
| FQ4QJ5301B8RT0 | 248 | 4.00E-20 | 49/71 (69%) | Neurospora crassa OR74A | hypothetical protein NCU06726 |
| FQ92HJ001CX7P0 | 77 | 2.90E+00 | 13/30 (43%) | Neurospora crassa OR74A | hypothetical protein NCU06864 |
| Contig3088 | 163 | 4.00E-39 | 74/100 (74%) | Neurospora crassa OR74A | hypothetical protein NCU06870 |
| Contig3413 | 36.6 | 6.00E-01 | 14/49 (28%) | Neurospora crassa OR74A | hypothetical protein NCU07125 |
| FQ4QJ5301B5E9T | 152 | 6.00E-09 | 37/66 (56%) | Neurospora crassa OR74A | hypothetical protein NCU07379 |
| Contig15787 | 100 | 4.00E-20 | 44/68 (64%) | Neurospora crassa OR74A | hypothetical protein NCU07536 |
| FQ4QJ5301DFUJV | 76 | 3.80E+00 | 17/62 (27%) | Neurospora crassa OR74A | hypothetical protein NCU07686 |
| FQ4QJ5301ALSX9 | 157 | 2.00E-09 | 26/55 (47%) | Neurospora crassa OR74A | hypothetical protein NCU07715 |
| Contig7799 | 102 | 9.00E-21 | 52/78 (66%) | Neurospora crassa OR74A | hypothetical protein NCU07735 |
| FQ4QJ5301A7WJA | 78 | 2.20E+00 | 17/48 (35%) | Neurospora crassa OR74A | hypothetical protein NCU07879 |
| FQ4QJ5301DX600 | 144 | 5.00E-08 | 30/62 (48%) | Neurospora crassa OR74A | hypothetical protein NCU08002 |
| Contig12840 | 58.2 | 2.00E-07 | 28/57 (49%) | Neurospora crassa OR74A | hypothetical protein NCU08093 |
| FQ4QJ5301CJCZV | 111 | 3.00E-04 | 23/35 (65%) | Neurospora crassa OR74A | hypothetical protein NCU08145 |
| FQ92HJ001BLCWJ | 83 | 5.90E-01 | 19/57 (33%) | Neurospora crassa OR74A | hypothetical protein NCU08226 |
| Contig14323 | 179 | 7.00E-44 | 81/100 (81%) | Neurospora crassa OR74A | hypothetical protein NCU08505 |
| FQ4QJ5301COCDZ | 191 | 2.00E-22 | 36/44 (81%) | Neurospora crassa OR74A | hypothetical protein NCU08607 |
| FQ92HJ001CUMTC | 295 | 2.00E-25 | 58/79 (73%) | Neurospora crassa OR74A | hypothetical protein NCU08743 |
| FQ4QJ5301B577L | 115 | 1.00E-04 | 23/26 (88%) | Neurospora crassa OR74A | hypothetical protein NCU08811 |
| Contig9504 | 32.7 | 8.60E+00 | 19/47 (40%) | Neurospora crassa OR74A | hypothetical protein NCU08887 |
| FQ4QJ5301C4VT6 | 152 | 4.00E-19 | 30/44 (68%) | Neurospora crassa OR74A | hypothetical protein NCU08909 |
| FQ92HJ001DHWB1 | 362 | 3.00E-33 | 69/76 (90%) | Neurospora crassa OR74A | hypothetical protein NCU08944 |
| FQ4QJ5301AJF73 | 277 | 2.00E-23 | 58/62 (93%) | Neurospora crassa OR74A | hypothetical protein NCU08946 |
| FQ92HJ001CE7X3 | 177 | 8.00E-12 | 30/65 (46%) | Neurospora crassa OR74A | hypothetical protein NCU08954 |
| Contig5960 | 58.9 | 1.00E-07 | 32/76 (42%) | Neurospora crassa OR74A | hypothetical protein NCU08956 |
| Contig5858 | 87 | 4.00E-16 | 44/60 (73%) | Neurospora crassa OR74A | hypothetical protein NCU09014 |
| Contig15426 | 33.9 | 3.80E+00 | 15/42 (35%) | Neurospora crassa OR74A | hypothetical protein NCU09031 |
| FQ92HJ001DFKHP | 76 | 3.90E+00 | 14/24 (58%) | Neurospora crassa OR74A | hypothetical protein NCU09233 |
| FQ4QJ5301CAP6M | 176 | 2.00E-25 | 34/45 (75%) | Neurospora crassa OR74A | hypothetical protein NCU09332 |
| FQ4QJ5301BLYLE | 243 | 2.00E-19 | 46/81 (56%) | Neurospora crassa OR74A | hypothetical protein NCU09543 |
| Contig1732 | 100 | 4.00E-20 | 41/61 (67%) | Neurospora crassa OR74A | hypothetical protein NCU09571 |
| FQ92HJ001D8LX9 | 117 | 7.00E-05 | 20/36 (55%) | Neurospora crassa OR74A | hypothetical protein NCU09575 |
| FQ92HJ001EMWAK | 328 | 2.00E-29 | 59/84 (70%) | Neurospora crassa OR74A | hypothetical protein NCU09705 |
| Contig5233 | 68.6 | 1.00E-10 | 32/75 (42%) | Neurospora crassa OR74A | hypothetical protein NCU10006 |
| FQ4QJ5301A8HPY | 119 | 4.00E-05 | 21/51 (41%) | Neurospora crassa OR74A | hypothetical protein NCU10285 |
| Contig618 | 100 | 4.00E-20 | 47/60 (78%) | Neurospora crassa OR74A | hypothetical protein NCU10351 |
| FQ4QJ5301DOPH5 | 289 | 8.00E-25 | 54/80 (67%) | Neurospora crassa OR74A | hypothetical protein NCU10715 |
| FQ4QJ5301EVC2N | 237 | 8.00E-19 | 47/66 (71%) | Neurospora crassa OR74A | hypothetical protein NCU11174 |
| FQ4QJ5301B0BE8 | 100 | 6.00E-03 | 17/24 (70%) | Neurospora crassa OR74A | hypothetical protein NCU11261 |
| Contig16369 | 162 | 8.00E-39 | 72/81 (88%) | Neurospora crassa OR74A | hypothetical protein NCU11339 |
| Contig3977 | 90.9 | 3.00E-17 | 44/65 (67%) | Neurospora crassa OR74A | hypothetical protein NCU11378 |
| FQ4QJ5301ECS86 | 157 | 2.00E-09 | 26/47 (55%) | Neurospora crassa OR74A | hypothetical protein NCU11398 |
| FQ4QJ5301C4QSS | 310 | 3.00E-27 | 58/67 (86%) | Neurospora crassa OR74A | imidazoleglycerol-phosphate dehydratase |
| FQ92HJ001B9VEL | 213 | 5.00E-16 | 42/50 (84%) | Neurospora crassa OR74A | nitrate reductase |
| Contig15408 | 183 | 3.00E-45 | 84/110 (76%) | Neurospora crassa OR74A | nucleoside diphosphate kinase, variant |
| FQ4QJ5301ELKOQ | 217 | 2.00E-16 | 47/76 (61%) | Neurospora crassa OR74A | pre-mRNA splicing factor slt11 |
| Contig4308 | 102 | 9.00E-21 | 48/51 (94%) | Neurospora crassa OR74A | protein ORM1 |
| Contig8707 | 129 | 7.00E-29 | 58/83 (69%) | Neurospora crassa OR74A | protein PNS1 |
| FQ4QJ5301EXJLR | 257 | 2.00E-29 | 49/53 (92%) | Neurospora crassa OR74A | ran-specific GTPase-activating protein 1 |
| Contig10378 | 47.8 | 3.00E-04 | 22/26 (84%) | Neurospora crassa OR74A | small nuclear ribonucleoprotein Sm D2 |
| Contig7042 | 141 | 1.00E-32 | 70/75 (93%) | Neurospora crassa OR74A | T-complex protein 1 subunit zeta |
| FQ4QJ5301BQ8OW | 178 | 4.00E-27 | 33/36 (91%) | Neurospora crassa OR74A | uracil phosphoribosyltransferase |
| FQ4QJ5301BDQFV | 417 | 1.00E-39 | 76/89 (85%) | Neurospora crassa OR74A | vacuolar ATP synthase catalytic subunit A |
| FQ4QJ5301B13QH | 313 | 3.00E-29 | 63/68 (92%) | Neurospora crassa OR74A | vacuolar ATP synthase subunit B |
| FQ92HJ001E4RGM | 73 | 8.40E+00 | 18/37 (48%) | Ophiostoma ulmi | putative maturase |
| FQ4QJ5301AG33Z | 150 | 1.00E-08 | 27/80 (33%) | Paracoccidioides brasiliensis | hypothetical protein |
| Contig2238 | 82.8 | 7.00E-15 | 46/132 (34%) | Paracoccidioides brasiliensis | reverse transcriptase |
| Contig4527 | 63.2 | 1.00E-09 | 32/57 (56%) | Paracoccidioides brasiliensis | reverse transcriptase |
| Contig9227 | 34.7 | 2.30E+00 | 21/64 (32%) | Paracoccidioides brasiliensis | reverse transcriptase |
| Contig10634 | 33.5 | 5.00E+00 | 23/68 (33%) | Paracoccidioides brasiliensis | reverse transcriptase |
| FQ4QJ5301ALJS8 | 137 | 3.00E-07 | 33/84 (39%) | Paracoccidioides brasiliensis | reverse transcriptase |
| FQ92HJ001B7ATO | 114 | 1.00E-04 | 23/71 (32%) | Paracoccidioides brasiliensis | AF419158\_1 N-acetyl-beta-glucosaminidase |
| Contig1991 | 76.6 | 5.00E-13 | 42/95 (44%) | Paracoccidioides brasiliensis | AF443189\_2 Trev |
| Contig4090 | 38.5 | 3.40E-01 | 18/37 (48%) | Paracoccidioides brasiliensis | AF443189\_2 Trev |
| FQ4QJ5301BVHKS | 181 | 3.00E-12 | 34/53 (64%) | Paracoccidioides brasiliensis | AF443189\_2 Trev |
| FQ92HJ001CDY5S | 98 | 1.10E-02 | 27/81 (33%) | Paracoccidioides brasiliensis | AF443189\_2 Trev |
| FQ92HJ001C38B0 | 102 | 4.00E-03 | 24/53 (45%) | Paracoccidioides brasiliensis | AF443189\_2 Trev |
| FQ92HJ001B4JMA | 67 | 1.90E-02 | 12/20 (60%) | Paracoccidioides brasiliensis | AF443189\_2 Trev |
| FQ92HJ001BMGS1 | 90 | 9.10E-02 | 19/45 (42%) | Paracoccus denitrificans | DNA polymerase III, epsilon subunit |
| FQ4QJ5301CPF8R | 209 | 1.00E-15 | 42/46 (91%) | Paracoccus denitrificans PD1222 | Triosephosphate isomerase |
| FQ92HJ001BS3IY | 144 | 5.00E-08 | 33/61 (54%) | Phaeosphaeria nodorum | polyprotein |
| Contig11689 | 63.2 | 6.00E-09 | 29/41 (70%) | Phaeosphaeria nodorum SN15 | hypothetical protein SNOG\_00185 |
| Contig7657 | 34.3 | 2.90E+00 | 16/46 (34%) | Phaeosphaeria nodorum SN15 | hypothetical protein SNOG\_00301 |
| FQ92HJ001AHUU5 | 108 | 7.00E-04 | 16/26 (61%) | Phaeosphaeria nodorum SN15 | hypothetical protein SNOG\_00999 |
| FQ4QJ5301ENL97 | 80 | 1.30E+00 | 20/79 (25%) | Phaeosphaeria nodorum SN15 | hypothetical protein SNOG\_01204 |
| Contig1797 | 35.8 | 1.00E+00 | 19/54 (35%) | Phaeosphaeria nodorum SN15 | hypothetical protein SNOG\_01234 |
| FQ4QJ5301CNGWC | 87 | 2.10E-01 | 20/41 (48%) | Phaeosphaeria nodorum SN15 | hypothetical protein SNOG\_01693 |
| FQ4QJ5301BUJ2G | 73 | 8.40E+00 | 11/21 (52%) | Phaeosphaeria nodorum SN15 | hypothetical protein SNOG\_01836 |
| FQ92HJ001EEN1V | 78 | 2.20E+00 | 13/24 (54%) | Phaeosphaeria nodorum SN15 | hypothetical protein SNOG\_02141 |
| Contig14610 | 33.5 | 5.00E+00 | 12/27 (44%) | Phaeosphaeria nodorum SN15 | hypothetical protein SNOG\_02269 |
| Contig3677 | 77 | 4.00E-13 | 40/74 (54%) | Phaeosphaeria nodorum SN15 | hypothetical protein SNOG\_02458 |
| Contig10307 | 37 | 4.40E-01 | 24/77 (31%) | Phaeosphaeria nodorum SN15 | hypothetical protein SNOG\_02561 |
| Contig6435 | 32.7 | 8.60E+00 | 12/19 (63%) | Phaeosphaeria nodorum SN15 | hypothetical protein SNOG\_02960 |
| Contig3869 | 156 | 5.00E-37 | 79/80 (98%) | Phaeosphaeria nodorum SN15 | hypothetical protein SNOG\_03347 |
| FQ4QJ5301D6P98 | 402 | 6.00E-38 | 80/88 (90%) | Phaeosphaeria nodorum SN15 | hypothetical protein SNOG\_03571 |
| FQ4QJ5301C13MT | 90 | 2.00E-11 | 17/23 (73%) | Phaeosphaeria nodorum SN15 | hypothetical protein SNOG\_03615 |
| FQ92HJ001EKPEK | 194 | 8.00E-14 | 32/42 (76%) | Phaeosphaeria nodorum SN15 | hypothetical protein SNOG\_04108 |
| FQ4QJ5301DP58A | 101 | 5.00E-03 | 18/28 (64%) | Phaeosphaeria nodorum SN15 | hypothetical protein SNOG\_04468 |
| Contig6588 | 36.6 | 5.90E-01 | 26/80 (32%) | Phaeosphaeria nodorum SN15 | hypothetical protein SNOG\_04493 |
| Contig8146 | 229 | 6.00E-59 | 116/130 (89%) | Phaeosphaeria nodorum SN15 | hypothetical protein SNOG\_04678 |
| FQ92HJ001AFE79 | 77 | 3.00E+00 | 17/53 (32%) | Phaeosphaeria nodorum SN15 | hypothetical protein SNOG\_04694 |
| Contig4164 | 100 | 4.00E-20 | 51/63 (80%) | Phaeosphaeria nodorum SN15 | hypothetical protein SNOG\_04728 |
| FQ92HJ001D7FQC | 186 | 7.00E-13 | 34/50 (68%) | Phaeosphaeria nodorum SN15 | hypothetical protein SNOG\_04963 |
| Contig11339 | 34.7 | 2.20E+00 | 16/41 (39%) | Phaeosphaeria nodorum SN15 | hypothetical protein SNOG\_05057 |
| FQ92HJ001COND8 | 73 | 8.40E+00 | 18/43 (41%) | Phaeosphaeria nodorum SN15 | hypothetical protein SNOG\_05521 |
| Contig3671 | 152 | 6.00E-36 | 74/81 (91%) | Phaeosphaeria nodorum SN15 | hypothetical protein SNOG\_05601 |
| FQ4QJ5301CSDDD | 74 | 6.60E+00 | 15/46 (32%) | Phaeosphaeria nodorum SN15 | hypothetical protein SNOG\_05702 |
| FQ4QJ5301ALJ20 | 156 | 2.00E-09 | 29/35 (82%) | Phaeosphaeria nodorum SN15 | hypothetical protein SNOG\_05948 |
| FQ92HJ001AI3IV | 247 | 6.00E-20 | 46/71 (64%) | Phaeosphaeria nodorum SN15 | hypothetical protein SNOG\_06049 |
| Contig10090 | 33.1 | 6.60E+00 | 15/40 (37%) | Phaeosphaeria nodorum SN15 | hypothetical protein SNOG\_06187 |
| FQ4QJ5301CY819 | 75 | 5.00E+00 | 14/29 (48%) | Phaeosphaeria nodorum SN15 | hypothetical protein SNOG\_06265 |
| FQ92HJ001A01SZ | 76 | 3.80E+00 | 17/56 (30%) | Phaeosphaeria nodorum SN15 | hypothetical protein SNOG\_06769 |
| FQ4QJ5301BYGHE | 79 | 1.70E+00 | 15/38 (39%) | Phaeosphaeria nodorum SN15 | hypothetical protein SNOG\_07507 |
| Contig12787 | 34.7 | 2.20E+00 | 23/65 (35%) | Phaeosphaeria nodorum SN15 | hypothetical protein SNOG\_07613 |
| FQ4QJ5301DXD05 | 141 | 1.00E-07 | 29/38 (76%) | Phaeosphaeria nodorum SN15 | hypothetical protein SNOG\_07618 |
| Contig7306 | 53.1 | 3.00E-08 | 21/38 (55%) | Phaeosphaeria nodorum SN15 | hypothetical protein SNOG\_08491 |
| Contig13045 | 109 | 6.00E-23 | 52/74 (70%) | Phaeosphaeria nodorum SN15 | hypothetical protein SNOG\_08503 |
| FQ4QJ5301BMDLK | 122 | 2.00E-05 | 31/82 (37%) | Phaeosphaeria nodorum SN15 | hypothetical protein SNOG\_08783 |
| Contig1985 | 35.8 | 1.30E+00 | 17/23 (73%) | Phaeosphaeria nodorum SN15 | hypothetical protein SNOG\_09061 |
| Contig9675 | 34.7 | 2.20E+00 | 17/25 (68%) | Phaeosphaeria nodorum SN15 | hypothetical protein SNOG\_09061 |
| FQ4QJ5301APJN2 | 81 | 1.00E+00 | 17/23 (73%) | Phaeosphaeria nodorum SN15 | hypothetical protein SNOG\_09061 |
| FQ92HJ001AXKWL | 84 | 4.50E-01 | 22/58 (37%) | Phaeosphaeria nodorum SN15 | hypothetical protein SNOG\_09178 |
| Contig10278 | 44.7 | 2.00E-03 | 20/24 (83%) | Phaeosphaeria nodorum SN15 | hypothetical protein SNOG\_09248 |
| FQ4QJ5301ALAWK | 206 | 3.00E-15 | 43/45 (95%) | Phaeosphaeria nodorum SN15 | hypothetical protein SNOG\_09278 |
| Contig11643 | 35.8 | 1.00E+00 | 18/48 (37%) | Phaeosphaeria nodorum SN15 | hypothetical protein SNOG\_09297 |
| FQ92HJ001DLKFO | 73 | 8.60E+00 | 23/70 (32%) | Phaeosphaeria nodorum SN15 | hypothetical protein SNOG\_09336 |
| FQ92HJ001EE5AY | 73 | 8.60E+00 | 17/59 (28%) | Phaeosphaeria nodorum SN15 | hypothetical protein SNOG\_09972 |
| Contig15039 | 35.8 | 1.00E+00 | 25/61 (40%) | Phaeosphaeria nodorum SN15 | hypothetical protein SNOG\_10159 |
| FQ4QJ5301D2F0M | 126 | 1.00E-06 | 24/50 (48%) | Phaeosphaeria nodorum SN15 | hypothetical protein SNOG\_10169 |
| FQ4QJ5301BLJSS | 87 | 2.10E-01 | 28/74 (37%) | Phaeosphaeria nodorum SN15 | hypothetical protein SNOG\_10351 |
| FQ4QJ5301EFJ8C | 132 | 1.00E-06 | 29/69 (42%) | Phaeosphaeria nodorum SN15 | hypothetical protein SNOG\_10592 |
| FQ4QJ5301A06AB | 73 | 8.70E+00 | 17/46 (36%) | Phaeosphaeria nodorum SN15 | hypothetical protein SNOG\_10747 |
| FQ4QJ5301ETGYY | 74 | 6.50E+00 | 14/33 (42%) | Phaeosphaeria nodorum SN15 | hypothetical protein SNOG\_10980 |
| FQ92HJ001B9XD8 | 85 | 3.50E-01 | 17/17 (100%) | Phaeosphaeria nodorum SN15 | hypothetical protein SNOG\_11637 |
| FQ4QJ5301BJB4N | 78 | 2.30E+00 | 18/35 (51%) | Phaeosphaeria nodorum SN15 | hypothetical protein SNOG\_11706 |
| FQ4QJ5301CNY7L | 409 | 9.00E-39 | 78/88 (88%) | Phaeosphaeria nodorum SN15 | hypothetical protein SNOG\_11841 |
| Contig4258 | 65.1 | 2.00E-09 | 31/35 (88%) | Phaeosphaeria nodorum SN15 | hypothetical protein SNOG\_12136 |
| Contig5304 | 43.9 | 4.00E-03 | 16/27 (59%) | Phaeosphaeria nodorum SN15 | hypothetical protein SNOG\_12246 |
| FQ4QJ5301AE4TD | 151 | 6.00E-13 | 29/33 (87%) | Phaeosphaeria nodorum SN15 | hypothetical protein SNOG\_12267 |
| Contig16496 | 36.6 | 5.90E-01 | 22/58 (37%) | Phaeosphaeria nodorum SN15 | hypothetical protein SNOG\_12442 |
| Contig355 | 134 | 2.00E-30 | 62/78 (79%) | Phaeosphaeria nodorum SN15 | hypothetical protein SNOG\_12876 |
| FQ92HJ001A02PT | 73 | 8.60E+00 | 13/21 (61%) | Phaeosphaeria nodorum SN15 | hypothetical protein SNOG\_13060 |
| FQ92HJ001B9NLR | 73 | 8.50E+00 | 21/53 (39%) | Phaeosphaeria nodorum SN15 | hypothetical protein SNOG\_13135 |
| FQ4QJ5301D0JW7 | 123 | 1.00E-05 | 22/30 (73%) | Phaeosphaeria nodorum SN15 | hypothetical protein SNOG\_13366 |
| Contig13912 | 33.1 | 6.50E+00 | 18/43 (41%) | Phaeosphaeria nodorum SN15 | hypothetical protein SNOG\_13665 |
| FQ4QJ5301EEEN2 | 88 | 1.50E-01 | 16/28 (57%) | Phaeosphaeria nodorum SN15 | hypothetical protein SNOG\_14173 |
| Contig2081 | 33.9 | 3.90E+00 | 24/78 (30%) | Phaeosphaeria nodorum SN15 | hypothetical protein SNOG\_14842 |
| FQ4QJ5301EAQDO | 156 | 2.00E-09 | 27/28 (96%) | Phaeosphaeria nodorum SN15 | hypothetical protein SNOG\_15214 |
| FQ4QJ5301CO1SK | 112 | 3.00E-04 | 25/54 (46%) | Phaeosphaeria nodorum SN15 | hypothetical protein SNOG\_15528 |
| FQ4QJ5301EOQEQ | 149 | 2.00E-16 | 28/33 (84%) | Phaeosphaeria nodorum SN15 | hypothetical protein SNOG\_15648 |
| Contig12960 | 34.3 | 2.90E+00 | 16/34 (47%) | Phaeosphaeria nodorum SN15 | hypothetical protein SNOG\_16106 |
| FQ92HJ001AGI1O | 84 | 4.60E-01 | 14/20 (70%) | Phaeosphaeria nodorum SN15 | hypothetical protein SNOG\_16370 |
| Contig15053 | 59.7 | 7.00E-08 | 24/55 (43%) | Phaeosphaeria nodorum SN15 | hypothetical protein SNOG\_16450 |
| FQ92HJ001EA1QS | 220 | 8.00E-17 | 42/51 (82%) | Phaeosphaeria nodorum SN15 | hypothetical protein SNOG\_16469 |
| Contig15295 | 33.1 | 6.60E+00 | 19/57 (33%) | Pichia | activator of transcription of nitrogen-regulated genes |
| Contig1661 | 34.3 | 3.00E+00 | 17/52 (32%) | Pichia | ATP dependent RNA helicase and U5 mRNA splicing factor |
| Contig11239 | 34.3 | 2.90E+00 | 19/44 (43%) | Pichia | Dedicator of cytokinesis protein 4 CRK binding protein |
| FQ92HJ001AE9ZI | 73 | 8.60E+00 | 16/41 (39%) | Pichia guilliermondii ATCC 6260 | hypothetical protein PGUG\_00547 |
| FQ4QJ5301CY25A | 75 | 4.90E+00 | 12/24 (50%) | Pichia guilliermondii ATCC 6260 | hypothetical protein PGUG\_00930 |
| FQ92HJ001DOUQ5 | 111 | 3.00E-04 | 27/82 (32%) | Pichia guilliermondii ATCC 6260 | hypothetical protein PGUG\_01136 |
| Contig13063 | 33.1 | 6.60E+00 | 21/68 (30%) | Pichia guilliermondii ATCC 6260 | hypothetical protein PGUG\_02378 |
| FQ4QJ5301ASMJS | 73 | 8.40E+00 | 21/72 (29%) | Pichia guilliermondii ATCC 6260 | hypothetical protein PGUG\_02558 |
| Contig9160 | 33.5 | 5.10E+00 | 20/48 (41%) | Pichia guilliermondii ATCC 6260 | hypothetical protein PGUG\_02926 |
| FQ92HJ001EPYG5 | 76 | 3.80E+00 | 14/32 (43%) | Pichia guilliermondii ATCC 6260 | hypothetical protein PGUG\_03063 |
| FQ92HJ001ARXVL | 74 | 6.60E+00 | 18/51 (35%) | Pichia guilliermondii ATCC 6260 | hypothetical protein PGUG\_03084 |
| Contig4875 | 34.3 | 2.90E+00 | 19/81 (23%) | Pichia guilliermondii ATCC 6260 | hypothetical protein PGUG\_03107 |
| Contig7817 | 37.4 | 3.40E-01 | 21/52 (40%) | Pichia guilliermondii ATCC 6260 | hypothetical protein PGUG\_03248 |
| Contig2769 | 38.5 | 1.50E-01 | 31/111 (27%) | Pichia guilliermondii ATCC 6260 | hypothetical protein PGUG\_03340 |
| FQ92HJ001B5T0V | 77 | 2.90E+00 | 13/23 (56%) | Pichia guilliermondii ATCC 6260 | hypothetical protein PGUG\_03398 |
| FQ92HJ001CBK2L | 78 | 2.30E+00 | 14/40 (35%) | Pichia guilliermondii ATCC 6260 | hypothetical protein PGUG\_03622 |
| FQ92HJ001BLD6Z | 73 | 8.60E+00 | 22/77 (28%) | Pichia guilliermondii ATCC 6260 | hypothetical protein PGUG\_03829 |
| Contig14141 | 34.3 | 3.00E+00 | 22/80 (27%) | Pichia guilliermondii ATCC 6260 | hypothetical protein PGUG\_04030 |
| Contig14631 | 36.6 | 5.80E-01 | 17/52 (32%) | Pichia guilliermondii ATCC 6260 | hypothetical protein PGUG\_04397 |
| Contig12511 | 32.7 | 8.40E+00 | 13/27 (48%) | Pichia guilliermondii ATCC 6260 | hypothetical protein PGUG\_04426 |
| Contig3436 | 37 | 4.90E-01 | 18/60 (30%) | Pichia guilliermondii ATCC 6260 | hypothetical protein PGUG\_05516 |
| FQ4QJ5301D074M | 74 | 6.50E+00 | 16/48 (33%) | Pichia guilliermondii ATCC 6260 | predicted protein |
| FQ92HJ001D2WSS | 75 | 5.00E+00 | 21/57 (36%) | Pichia guilliermondii ATCC 6260 | predicted protein |
| FQ92HJ001A3NJI | 76 | 3.80E+00 | 14/45 (31%) | Pichia guilliermondii ATCC 6260 | predicted protein |
| FQ92HJ001BOQLF | 81 | 1.00E+00 | 16/48 (33%) | Pichia guilliermondii ATCC 6260 | predicted protein |
| FQ4QJ5301CZB23 | 73 | 8.50E+00 | 19/62 (30%) | Pichia stipitis | fungal specific zinc-finger transcription factor |
| FQ4QJ5301CQHHR | 74 | 6.50E+00 | 19/62 (30%) | Pichia stipitis CBS | Inositol-1,4,5-triphosphate 5-phosphatase |
| Contig9292 | 32.7 | 8.40E+00 | 26/82 (31%) | Pichia stipitis CBS | Phosphate metabolism transcription protein |
| FQ92HJ001D3YGB | 80 | 1.30E+00 | 15/33 (45%) | Pichia stipitis CBS 6054 | ATP-dependent DNA helicase |
| FQ92HJ001EB0F8 | 75 | 4.90E+00 | 16/39 (41%) | Pichia stipitis CBS 6054 | F-box/LRR-repeat protein |
| FQ4QJ5301AGCTU | 76 | 3.90E+00 | 14/37 (37%) | Pichia stipitis CBS 6054 | hyphally regulated cell wall protein |
| FQ4QJ5301B95WF | 81 | 1.00E+00 | 16/38 (42%) | Pichia stipitis CBS 6054 | hypothetical protein PICST\_15898 |
| FQ4QJ5301BXPSZ | 73 | 8.40E+00 | 14/36 (38%) | Pichia stipitis CBS 6054 | hypothetical protein PICST\_29844 |
| FQ92HJ001DN3C7 | 75 | 5.00E+00 | 14/41 (34%) | Pichia stipitis CBS 6054 | hypothetical protein PICST\_30373 |
| Contig10738 | 37.4 | 6.70E-01 | 21/70 (30%) | Pichia stipitis CBS 6054 | hypothetical protein PICST\_30663 |
| Contig6918 | 32.7 | 8.40E+00 | 17/50 (34%) | Pichia stipitis CBS 6054 | hypothetical protein PICST\_30756 |
| Contig3082 | 40 | 5.30E-02 | 29/90 (32%) | Pichia stipitis CBS 6054 | hypothetical protein PICST\_31188 |
| Contig4098 | 33.9 | 3.80E+00 | 14/32 (43%) | Pichia stipitis CBS 6054 | hypothetical protein PICST\_32971 |
| FQ92HJ001CQYG6 | 74 | 6.40E+00 | 16/29 (55%) | Pichia stipitis CBS 6054 | hypothetical protein PICST\_50995 |
| FQ4QJ5301EMBWQ | 76 | 3.90E+00 | 21/83 (25%) | Pichia stipitis CBS 6054 | hypothetical protein PICST\_60807 |
| Contig8661 | 34.3 | 2.90E+00 | 12/27 (44%) | Pichia stipitis CBS 6054 | hypothetical protein PICST\_67290 |
| Contig1651 | 40.8 | 3.10E-02 | 25/90 (27%) | Pichia stipitis CBS 6054 | hypothetical protein PICST\_67901 |
| FQ4QJ5301AMZBX | 73 | 8.70E+00 | 15/59 (25%) | Pichia stipitis CBS 6054 | hypothetical protein PICST\_87156 |
| Contig12418 | 34.3 | 2.90E+00 | 17/34 (50%) | Pichia stipitis CBS 6054 | pH-response regulator protein palI/RIM9 |
| Contig4744 | 35 | 1.70E+00 | 19/63 (30%) | Pichia stipitis CBS 6054 | predicted protein |
| Contig16121 | 33.9 | 7.30E+00 | 17/45 (37%) | Pichia stipitis CBS 6054 | predicted protein |
| Contig16336 | 33.5 | 5.00E+00 | 18/51 (35%) | Pichia stipitis CBS 6054 | predicted protein |
| FQ4QJ5301BAX05 | 75 | 5.10E+00 | 16/38 (42%) | Pichia stipitis CBS 6054 | predicted protein |
| FQ4QJ5301A4N2V | 73 | 8.40E+00 | 13/30 (43%) | Pichia stipitis CBS 6054 | predicted protein |
| FQ92HJ001EMFKK | 76 | 3.80E+00 | 12/33 (36%) | Pichia stipitis CBS 6054 | suppressor of gal11 null |
| FQ4QJ5301DWGT5 | 104 | 2.00E-03 | 17/39 (43%) | Pichia stipitis CBS 6054 | zinc finger protein |
| Contig5845 | 33.5 | 4.90E+00 | 16/40 (40%) | Pneumocystis carinii | beta-1,3-glucan synthase GSC-1 |
| FQ4QJ5301CKQN5 | 95 | 2.40E-02 | 17/22 (77%) | Pneumocystis carinii | AF494449\_1 p55 antigen variant V2 |
| Contig657 | 47 | 4.00E-04 | 24/57 (42%) | Podospora anserina | unnamed protein product |
| Contig822 | 142 | 8.00E-33 | 71/75 (94%) | Podospora anserina | unnamed protein product |
| Contig1042 | 59.7 | 7.00E-08 | 27/27 (100%) | Podospora anserina | unnamed protein product |
| Contig1150 | 110 | 4.00E-23 | 51/65 (78%) | Podospora anserina | unnamed protein product |
| Contig1435 | 49.3 | 9.00E-05 | 26/37 (70%) | Podospora anserina | unnamed protein product |
| Contig1753 | 109 | 6.00E-23 | 54/76 (71%) | Podospora anserina | unnamed protein product |
| Contig2190 | 40.4 | 4.10E-02 | 20/45 (44%) | Podospora anserina | unnamed protein product |
| Contig2259 | 50.1 | 5.00E-05 | 21/26 (80%) | Podospora anserina | unnamed protein product |
| Contig2690 | 39.7 | 7.00E-02 | 18/18 (100%) | Podospora anserina | unnamed protein product |
| Contig2738 | 84.7 | 2.00E-15 | 40/46 (86%) | Podospora anserina | unnamed protein product |
| Contig2949 | 154 | 3.00E-36 | 67/135 (49%) | Podospora anserina | unnamed protein product |
| Contig3138 | 164 | 6.00E-39 | 80/104 (76%) | Podospora anserina | unnamed protein product |
| Contig3711 | 43.9 | 4.00E-03 | 31/70 (44%) | Podospora anserina | unnamed protein product |
| Contig3743 | 45.4 | 1.00E-03 | 19/26 (73%) | Podospora anserina | unnamed protein product |
| Contig3891 | 194 | 3.00E-48 | 92/106 (86%) | Podospora anserina | unnamed protein product |
| Contig4852 | 158 | 1.00E-37 | 75/83 (90%) | Podospora anserina | unnamed protein product |
| Contig4962 | 66.6 | 5.00E-10 | 32/36 (88%) | Podospora anserina | unnamed protein product |
| Contig5226 | 110 | 3.00E-23 | 52/62 (83%) | Podospora anserina | unnamed protein product |
| Contig5484 | 80.5 | 4.00E-14 | 34/60 (56%) | Podospora anserina | unnamed protein product |
| Contig5519 | 47.4 | 3.00E-04 | 23/42 (54%) | Podospora anserina | unnamed protein product |
| Contig5730 | 128 | 1.00E-31 | 62/63 (98%) | Podospora anserina | unnamed protein product |
| Contig6055 | 200 | 2.00E-50 | 98/108 (90%) | Podospora anserina | unnamed protein product |
| Contig6145 | 134 | 2.00E-30 | 69/84 (82%) | Podospora anserina | unnamed protein product |
| Contig6263 | 115 | 2.00E-24 | 62/94 (65%) | Podospora anserina | unnamed protein product |
| Contig6742 | 132 | 1.00E-29 | 62/76 (81%) | Podospora anserina | unnamed protein product |
| Contig6858 | 54.3 | 3.00E-06 | 24/30 (80%) | Podospora anserina | unnamed protein product |
| Contig6886 | 251 | 1.00E-65 | 130/157 (82%) | Podospora anserina | unnamed protein product |
| Contig7067 | 172 | 9.00E-42 | 88/109 (80%) | Podospora anserina | unnamed protein product |
| Contig7331 | 150 | 4.00E-35 | 73/80 (91%) | Podospora anserina | unnamed protein product |
| Contig7583 | 85.1 | 1.00E-24 | 38/42 (90%) | Podospora anserina | unnamed protein product |
| Contig7849 | 142 | 8.00E-33 | 74/87 (85%) | Podospora anserina | unnamed protein product |
| Contig8117 | 32.7 | 8.50E+00 | 14/15 (93%) | Podospora anserina | unnamed protein product |
| Contig8129 | 36.2 | 7.80E-01 | 20/59 (33%) | Podospora anserina | unnamed protein product |
| Contig8171 | 64.7 | 2.00E-09 | 33/43 (76%) | Podospora anserina | unnamed protein product |
| Contig8625 | 35.8 | 1.00E+00 | 19/36 (52%) | Podospora anserina | unnamed protein product |
| Contig9264 | 34.3 | 2.90E+00 | 20/51 (39%) | Podospora anserina | unnamed protein product |
| Contig9314 | 83.6 | 4.00E-15 | 35/59 (59%) | Podospora anserina | unnamed protein product |
| Contig10015 | 110 | 4.00E-23 | 58/80 (72%) | Podospora anserina | unnamed protein product |
| Contig10348 | 62.8 | 8.00E-09 | 29/51 (56%) | Podospora anserina | unnamed protein product |
| Contig10830 | 72 | 1.00E-11 | 33/63 (52%) | Podospora anserina | unnamed protein product |
| Contig11243 | 168 | 1.00E-40 | 82/82 (100%) | Podospora anserina | unnamed protein product |
| Contig11305 | 141 | 1.00E-32 | 64/78 (82%) | Podospora anserina | unnamed protein product |
| Contig11406 | 35.4 | 1.30E+00 | 19/44 (43%) | Podospora anserina | unnamed protein product |
| Contig11595 | 43.5 | 5.00E-03 | 28/61 (45%) | Podospora anserina | unnamed protein product |
| Contig11874 | 50.1 | 5.00E-05 | 24/24 (100%) | Podospora anserina | unnamed protein product |
| Contig11964 | 133 | 5.00E-30 | 68/89 (76%) | Podospora anserina | unnamed protein product |
| Contig12118 | 159 | 8.00E-38 | 79/97 (81%) | Podospora anserina | unnamed protein product |
| Contig12367 | 102 | 1.00E-20 | 41/64 (64%) | Podospora anserina | unnamed protein product |
| Contig12769 | 135 | 1.00E-30 | 70/140 (50%) | Podospora anserina | unnamed protein product |
| Contig12875 | 99.4 | 7.00E-20 | 42/71 (59%) | Podospora anserina | unnamed protein product |
| Contig13469 | 115 | 1.00E-24 | 55/55 (100%) | Podospora anserina | unnamed protein product |
| Contig13573 | 94 | 3.00E-18 | 40/41 (97%) | Podospora anserina | unnamed protein product |
| Contig13965 | 257 | 2.00E-67 | 127/135 (94%) | Podospora anserina | unnamed protein product |
| Contig14281 | 70.9 | 3.00E-11 | 36/59 (61%) | Podospora anserina | unnamed protein product |
| Contig14997 | 55.1 | 2.00E-06 | 30/78 (38%) | Podospora anserina | unnamed protein product |
| Contig15031 | 139 | 5.00E-32 | 73/112 (65%) | Podospora anserina | unnamed protein product |
| Contig15081 | 56.6 | 5.00E-07 | 22/25 (88%) | Podospora anserina | unnamed protein product |
| Contig15405 | 49.3 | 9.00E-05 | 24/24 (100%) | Podospora anserina | unnamed protein product |
| Contig15523 | 60.1 | 5.00E-08 | 31/71 (43%) | Podospora anserina | unnamed protein product |
| Contig16451 | 35.4 | 1.30E+00 | 21/59 (35%) | Podospora anserina | unnamed protein product |
| FQ4QJ5301EDEVQ | 157 | 2.00E-09 | 28/49 (57%) | Podospora anserina | unnamed protein product |
| FQ4QJ5301EAN0V | 78 | 2.20E+00 | 20/56 (35%) | Podospora anserina | unnamed protein product |
| FQ4QJ5301CPKUT | 298 | 7.00E-26 | 55/71 (77%) | Podospora anserina | unnamed protein product |
| FQ4QJ5301BKEUM | 127 | 5.00E-06 | 22/38 (57%) | Podospora anserina | unnamed protein product |
| FQ4QJ5301DYYOG | 76 | 3.90E+00 | 23/71 (32%) | Podospora anserina | unnamed protein product |
| FQ4QJ5301DRW99 | 99 | 8.00E-03 | 20/38 (52%) | Podospora anserina | unnamed protein product |
| FQ4QJ5301A2ALW | 402 | 6.00E-38 | 77/84 (91%) | Podospora anserina | unnamed protein product |
| FQ4QJ5301BOCWX | 248 | 4.00E-20 | 47/74 (63%) | Podospora anserina | unnamed protein product |
| FQ4QJ5301CBPPZ | 195 | 6.00E-14 | 39/69 (56%) | Podospora anserina | unnamed protein product |
| FQ4QJ5301EEY12 | 234 | 2.00E-18 | 46/78 (58%) | Podospora anserina | unnamed protein product |
| FQ4QJ5301EE32F | 423 | 2.00E-40 | 79/81 (97%) | Podospora anserina | unnamed protein product |
| FQ4QJ5301EQ4GP | 369 | 4.00E-34 | 70/75 (93%) | Podospora anserina | unnamed protein product |
| FQ4QJ5301DEDQ7 | 99 | 8.00E-03 | 18/18 (100%) | Podospora anserina | unnamed protein product |
| FQ4QJ5301EC653 | 194 | 8.00E-14 | 42/85 (49%) | Podospora anserina | unnamed protein product |
| FQ4QJ5301A4XWW | 183 | 2.00E-12 | 37/37 (100%) | Podospora anserina | unnamed protein product |
| FQ4QJ5301CS6A6 | 405 | 3.00E-38 | 76/83 (91%) | Podospora anserina | unnamed protein product |
| FQ4QJ5301EMUIZ | 77 | 2.90E+00 | 16/20 (80%) | Podospora anserina | unnamed protein product |
| FQ4QJ5301EV4SF | 99 | 8.00E-03 | 22/53 (41%) | Podospora anserina | unnamed protein product |
| FQ4QJ5301DOZRQ | 93 | 4.20E-02 | 20/25 (80%) | Podospora anserina | unnamed protein product |
| FQ4QJ5301EL68D | 216 | 2.00E-16 | 42/54 (77%) | Podospora anserina | unnamed protein product |
| FQ4QJ5301CNW4U | 237 | 8.00E-19 | 52/76 (68%) | Podospora anserina | unnamed protein product |
| FQ4QJ5301A8ZJ6 | 237 | 8.00E-19 | 45/58 (77%) | Podospora anserina | unnamed protein product |
| FQ4QJ5301CA8E9 | 404 | 4.00E-38 | 80/84 (95%) | Podospora anserina | unnamed protein product |
| FQ4QJ5301COMRS | 73 | 8.40E+00 | 15/43 (34%) | Podospora anserina | unnamed protein product |
| FQ4QJ5301DVTNE | 166 | 2.00E-13 | 31/37 (83%) | Podospora anserina | unnamed protein product |
| FQ4QJ5301APHVY | 391 | 1.00E-36 | 69/84 (82%) | Podospora anserina | unnamed protein product |
| FQ4QJ5301EIH1M | 114 | 2.00E-04 | 21/25 (84%) | Podospora anserina | unnamed protein product |
| FQ4QJ5301ELKRC | 214 | 4.00E-16 | 40/70 (57%) | Podospora anserina | unnamed protein product |
| FQ4QJ5301BPGNU | 75 | 5.00E+00 | 15/32 (46%) | Podospora anserina | unnamed protein product |
| FQ4QJ5301D5J49 | 214 | 4.00E-16 | 39/55 (70%) | Podospora anserina | unnamed protein product |
| FQ4QJ5301E1S4V | 118 | 5.00E-05 | 22/31 (70%) | Podospora anserina | unnamed protein product |
| FQ4QJ5301AOWUV | 189 | 3.00E-13 | 32/51 (62%) | Podospora anserina | unnamed protein product |
| FQ4QJ5301DEIXS | 83 | 5.90E-01 | 19/47 (40%) | Podospora anserina | unnamed protein product |
| FQ4QJ5301B5HRH | 133 | 9.00E-07 | 28/43 (65%) | Podospora anserina | unnamed protein product |
| FQ4QJ5301AJ77G | 301 | 3.00E-26 | 55/62 (88%) | Podospora anserina | unnamed protein product |
| FQ4QJ5301BUR29 | 104 | 2.00E-03 | 29/78 (37%) | Podospora anserina | unnamed protein product |
| FQ4QJ5301EFA75 | 147 | 7.00E-11 | 24/28 (85%) | Podospora anserina | unnamed protein product |
| FQ4QJ5301EG9IF | 112 | 3.00E-04 | 27/57 (47%) | Podospora anserina | unnamed protein product |
| FQ4QJ5301DPTB0 | 213 | 5.00E-16 | 38/41 (92%) | Podospora anserina | unnamed protein product |
| FQ4QJ5301EZFQG | 128 | 2.00E-09 | 20/31 (64%) | Podospora anserina | unnamed protein product |
| FQ4QJ5301CPESP | 310 | 3.00E-27 | 61/62 (98%) | Podospora anserina | unnamed protein product |
| FQ4QJ5301CWWG6 | 388 | 3.00E-36 | 71/73 (97%) | Podospora anserina | unnamed protein product |
| FQ4QJ5301C16PG | 81 | 1.00E+00 | 18/57 (31%) | Podospora anserina | unnamed protein product |
| FQ4QJ5301B1DUV | 174 | 2.00E-11 | 29/45 (64%) | Podospora anserina | unnamed protein product |
| FQ4QJ5301BWT0S | 97 | 1.40E-02 | 25/53 (47%) | Podospora anserina | unnamed protein product |
| FQ4QJ5301EXJF7 | 356 | 1.00E-32 | 61/84 (72%) | Podospora anserina | unnamed protein product |
| FQ4QJ5301CZ6E9 | 269 | 2.00E-22 | 49/63 (77%) | Podospora anserina | unnamed protein product |
| FQ4QJ5301AVMP9 | 263 | 6.00E-25 | 48/57 (84%) | Podospora anserina | unnamed protein product |
| FQ4QJ5301CQ6EU | 123 | 1.00E-05 | 22/26 (84%) | Podospora anserina | unnamed protein product |
| FQ4QJ5301E136R | 166 | 1.00E-10 | 38/70 (54%) | Podospora anserina | unnamed protein product |
| FQ4QJ5301C1VCJ | 298 | 7.00E-26 | 53/67 (79%) | Podospora anserina | unnamed protein product |
| FQ4QJ5301DDBHF | 136 | 1.00E-17 | 28/38 (73%) | Podospora anserina | unnamed protein product |
| FQ4QJ5301CRN26 | 78 | 2.20E+00 | 16/45 (35%) | Podospora anserina | unnamed protein product |
| FQ4QJ5301B0S9O | 77 | 2.90E+00 | 16/43 (37%) | Podospora anserina | unnamed protein product |
| FQ4QJ5301B60Z5 | 207 | 2.00E-15 | 41/66 (62%) | Podospora anserina | unnamed protein product |
| FQ4QJ5301B78BS | 62 | 3.70E-01 | 10/23 (43%) | Podospora anserina | unnamed protein product |
| FQ4QJ5301BJZX8 | 81 | 1.00E+00 | 22/51 (43%) | Podospora anserina | unnamed protein product |
| FQ4QJ5301EI3SB | 74 | 6.60E+00 | 15/37 (40%) | Podospora anserina | unnamed protein product |
| FQ4QJ5301BFOWS | 193 | 1.00E-13 | 34/45 (75%) | Podospora anserina | unnamed protein product |
| FQ4QJ5301DNPKN | 84 | 4.50E-01 | 22/56 (39%) | Podospora anserina | unnamed protein product |
| FQ4QJ5301CK2WR | 143 | 6.00E-08 | 29/44 (65%) | Podospora anserina | unnamed protein product |
| FQ4QJ5301B88S9 | 77 | 2.90E+00 | 17/50 (34%) | Podospora anserina | unnamed protein product |
| FQ4QJ5301ATZDD | 247 | 6.00E-20 | 44/55 (80%) | Podospora anserina | unnamed protein product |
| FQ4QJ5301C6S5C | 256 | 5.00E-21 | 51/79 (64%) | Podospora anserina | unnamed protein product |
| FQ4QJ5301CNC1D | 308 | 5.00E-27 | 58/73 (79%) | Podospora anserina | unnamed protein product |
| FQ4QJ5301CUWNO | 111 | 3.00E-04 | 19/25 (76%) | Podospora anserina | unnamed protein product |
| FQ4QJ5301DXRPV | 72 | 4.00E-02 | 17/33 (51%) | Podospora anserina | unnamed protein product |
| FQ4QJ5301CT9XI | 75 | 5.00E+00 | 12/16 (75%) | Podospora anserina | unnamed protein product |
| FQ4QJ5301DWECG | 80 | 1.10E+00 | 16/16 (100%) | Podospora anserina | unnamed protein product |
| FQ4QJ5301B7Q7R | 198 | 3.00E-14 | 36/44 (81%) | Podospora anserina | unnamed protein product |
| FQ4QJ5301AHTVC | 180 | 3.00E-12 | 35/35 (100%) | Podospora anserina | unnamed protein product |
| FQ4QJ5301EWHBK | 214 | 2.00E-18 | 39/44 (88%) | Podospora anserina | unnamed protein product |
| FQ4QJ5301EORNE | 155 | 1.00E-10 | 32/40 (80%) | Podospora anserina | unnamed protein product |
| FQ4QJ5301DY124 | 81 | 1.00E+00 | 19/40 (47%) | Podospora anserina | unnamed protein product |
| FQ4QJ5301DFYXL | 248 | 4.00E-20 | 46/55 (83%) | Podospora anserina | unnamed protein product |
| FQ92HJ001EITPM | 142 | 9.00E-08 | 22/40 (55%) | Podospora anserina | unnamed protein product |
| FQ92HJ001BYEZV | 131 | 2.00E-06 | 25/30 (83%) | Podospora anserina | unnamed protein product |
| FQ92HJ001D3S1P | 288 | 1.00E-24 | 53/59 (89%) | Podospora anserina | unnamed protein product |
| FQ92HJ001B85ZU | 106 | 1.00E-03 | 19/19 (100%) | Podospora anserina | unnamed protein product |
| FQ92HJ001APDZE | 196 | 5.00E-14 | 37/66 (56%) | Podospora anserina | unnamed protein product |
| FQ92HJ001C74KM | 119 | 4.00E-05 | 24/27 (88%) | Podospora anserina | unnamed protein product |
| FQ92HJ001EXMDN | 112 | 3.00E-04 | 22/45 (48%) | Podospora anserina | unnamed protein product |
| FQ92HJ001CZUQS | 155 | 3.00E-09 | 29/35 (82%) | Podospora anserina | unnamed protein product |
| FQ92HJ001DR8IA | 390 | 2.00E-36 | 70/84 (83%) | Podospora anserina | unnamed protein product |
| FQ92HJ001EH51Q | 219 | 1.00E-16 | 42/82 (51%) | Podospora anserina | unnamed protein product |
| FQ92HJ001BKNAF | 106 | 1.00E-03 | 20/20 (100%) | Podospora anserina | unnamed protein product |
| FQ92HJ001BQJ7S | 361 | 3.00E-33 | 67/68 (98%) | Podospora anserina | unnamed protein product |
| FQ92HJ001C23RO | 84 | 4.60E-01 | 22/48 (45%) | Podospora anserina | unnamed protein product |
| FQ92HJ001C32IQ | 87 | 2.10E-01 | 18/21 (85%) | Podospora anserina | unnamed protein product |
| FQ92HJ001DAGP4 | 74 | 6.50E+00 | 22/67 (32%) | Podospora anserina | unnamed protein product |
| FQ92HJ001CZR45 | 122 | 2.00E-05 | 21/43 (48%) | Podospora anserina | unnamed protein product |
| FQ92HJ001EZYTX | 116 | 9.00E-05 | 24/33 (72%) | Podospora anserina | unnamed protein product |
| FQ92HJ001CZ5N7 | 355 | 2.00E-32 | 69/73 (94%) | Podospora anserina | unnamed protein product |
| FQ92HJ001DREUM | 159 | 9.00E-10 | 28/47 (59%) | Podospora anserina | unnamed protein product |
| FQ92HJ001BSN8H | 360 | 5.00E-33 | 68/75 (90%) | Podospora anserina | unnamed protein product |
| FQ92HJ001CC85P | 73 | 8.60E+00 | 14/28 (50%) | Podospora anserina | unnamed protein product |
| FQ92HJ001BOD18 | 167 | 1.00E-10 | 30/32 (93%) | Podospora anserina | unnamed protein product |
| FQ92HJ001AHIDA | 112 | 3.00E-04 | 18/30 (60%) | Podospora anserina | unnamed protein product |
| FQ92HJ001DY5VV | 93 | 8.00E-08 | 17/22 (77%) | Podospora anserina | unnamed protein product |
| FQ92HJ001CKTEZ | 79 | 1.70E+00 | 11/20 (55%) | Podospora anserina | unnamed protein product |
| FQ92HJ001A685U | 147 | 2.00E-08 | 26/30 (86%) | Podospora anserina | unnamed protein product |
| FQ92HJ001A2ZZQ | 131 | 2.00E-06 | 26/54 (48%) | Podospora anserina | unnamed protein product |
| FQ92HJ001E20DM | 173 | 2.00E-11 | 38/86 (44%) | Podospora anserina | unnamed protein product |
| FQ92HJ001D9KGI | 80 | 1.30E+00 | 17/53 (32%) | Podospora anserina | unnamed protein product |
| FQ92HJ001BJ1DJ | 264 | 6.00E-22 | 49/95 (51%) | Podospora anserina | unnamed protein product |
| FQ92HJ001EMRR7 | 94 | 3.00E-02 | 17/18 (94%) | Podospora anserina | unnamed protein product |
| FQ92HJ001CV0G6 | 148 | 2.00E-08 | 33/60 (55%) | Podospora anserina | unnamed protein product |
| FQ92HJ001E1O45 | 85 | 3.20E-01 | 16/23 (69%) | Podospora anserina | unnamed protein product |
| FQ92HJ001DN7LK | 129 | 3.00E-06 | 23/26 (88%) | Podospora anserina | unnamed protein product |
| FQ92HJ001DMU8U | 193 | 1.00E-13 | 35/54 (64%) | Podospora anserina | unnamed protein product |
| FQ92HJ001B0PTN | 86 | 2.70E-01 | 28/87 (32%) | Podospora anserina | unnamed protein product |
| FQ92HJ001EAGX8 | 78 | 2.30E+00 | 14/17 (82%) | Podospora anserina | unnamed protein product |
| FQ92HJ001CDHOR | 194 | 8.00E-14 | 34/43 (79%) | Podospora anserina | unnamed protein product |
| FQ92HJ001DIRZE | 208 | 2.00E-15 | 43/44 (97%) | Podospora anserina | unnamed protein product |
| FQ92HJ001ASZTF | 169 | 6.00E-11 | 32/58 (55%) | Podospora anserina | unnamed protein product |
| FQ92HJ001EE7FP | 181 | 3.00E-12 | 38/81 (46%) | Podospora anserina | unnamed protein product |
| FQ92HJ001CNJFD | 139 | 2.00E-07 | 24/27 (88%) | Podospora anserina | unnamed protein product |
| FQ92HJ001EYSLU | 162 | 4.00E-10 | 32/38 (84%) | Podospora anserina | unnamed protein product |
| FQ92HJ001DE8YQ | 81 | 1.00E+00 | 15/25 (60%) | Podospora anserina | unnamed protein product |
| FQ92HJ001CC4DE | 354 | 2.00E-32 | 64/82 (78%) | Podospora anserina | unnamed protein product |
| FQ92HJ001C4SRX | 149 | 1.00E-08 | 29/60 (48%) | Podospora anserina | unnamed protein product |
| Contig1729 | 199 | 7.00E-50 | 94/94 (100%) | Ricania japonica yeast-like symbiont | uricase |
| FQ92HJ001DDDHG | 381 | 2.00E-35 | 77/77 (100%) | Ricania japonica yeast-like symbiont | uricase |
| Contig2013 | 35.4 | 1.30E+00 | 21/70 (30%) | Saccharomyces cerevisiae | polyadenylated RNA binding protein |
| Contig5870 | 33.5 | 7.00E+00 | 11/40 (27%) | Saccharomyces cerevisiae | unnamed protein product |
| Contig9112 | 37.7 | 2.60E-01 | 17/54 (31%) | Saccharomyces cerevisiae | unnamed protein product |
| FQ4QJ5301E82N5 | 80 | 1.30E+00 | 27/78 (34%) | Saccharomyces cerevisiae | unnamed protein product |
| FQ92HJ001BEBFT | 75 | 5.00E+00 | 16/45 (35%) | Saccharomyces cerevisiae | YCR592 |
| FQ92HJ001CMISG | 79 | 1.70E+00 | 19/73 (26%) | Saccharomyces cerevisiae | YOL101C |
| Contig9064 | 35 | 1.70E+00 | 15/42 (35%) | Saccharomyces cerevisiae YJM789 | choline kinase |
| Contig3063 | 38.5 | 1.60E-01 | 24/78 (30%) | Saccharomyces cerevisiae YJM789 | conserved protein |
| Contig9246 | 34.3 | 2.90E+00 | 21/70 (30%) | Saccharomyces cerevisiae YJM789 | conserved protein |
| Contig14764 | 32.7 | 8.50E+00 | 23/60 (38%) | Saccharomyces cerevisiae YJM789 | conserved protein |
| Contig15747 | 28.9 | 4.10E+00 | 18/44 (40%) | Saccharomyces cerevisiae YJM789 | conserved protein |
| FQ92HJ001BJDNL | 73 | 8.50E+00 | 13/34 (38%) | Saccharomyces cerevisiae YJM789 | conserved protein |
| FQ92HJ001BGSGZ | 81 | 1.00E+00 | 24/73 (32%) | Saccharomyces cerevisiae YJM789 | conserved protein |
| FQ92HJ001CJ2Q4 | 81 | 1.00E+00 | 24/85 (28%) | Saccharomyces cerevisiae YJM789 | conserved protein |
| Contig7678 | 39.3 | 9.30E-02 | 23/66 (34%) | Saccharomyces cerevisiae YJM789 | Gm18 tRNA methyltransferase |
| Contig5844 | 35.4 | 1.30E+00 | 16/47 (34%) | Saccharomyces cerevisiae YJM789 | hypothetical protein SCY\_2877 |
| FQ92HJ001BNU08 | 76 | 3.80E+00 | 18/49 (36%) | Saccharomyces cerevisiae YJM789 | Kre2-related protein |
| Contig7911 | 34.7 | 2.20E+00 | 18/44 (40%) | Saccharomyces cerevisiae YJM789 | mannosyltransferase |
| Contig14337 | 34.3 | 2.90E+00 | 18/63 (28%) | Saccharomyces cerevisiae YJM789 | muddled meiosis-related protein |
| Contig15441 | 33.9 | 4.70E+00 | 19/65 (29%) | Saccharomyces cerevisiae YJM789 | NMD pathway component |
| Contig16159 | 32.7 | 8.50E+00 | 31/113 (27%) | Saccharomyces cerevisiae YJM789 | petite colonies protein |
| FQ4QJ5301EBQUC | 83 | 6.00E-01 | 16/47 (34%) | Saccharomyces cerevisiae YJM789 | RNA polymerase A |
| Contig12264 | 35.4 | 1.30E+00 | 19/56 (33%) | Saccharomyces cerevisiae YJM789 | SAGA-associated factor |
| Contig16126 | 35 | 2.10E+00 | 18/64 (28%) | Saccharomyces cerevisiae YJM789 | THO complex subunit |
| FQ4QJ5301B3QML | 81 | 1.00E+00 | 21/76 (27%) | Saccharomyces cerevisiae YJM789 | Tip1-related protein |
| FQ4QJ5301A4O1H | 74 | 6.60E+00 | 23/79 (29%) | Saccharomyces cerevisiae YJM789 | U3 snoRNP protein |
| Contig13766 | 35.4 | 1.30E+00 | 19/46 (41%) | Saccharomyces cerevisiae YJM789 | ubiquitin-like protein |
| Contig4272 | 43.1 | 6.00E-03 | 25/81 (30%) | Schizosaccharomyces | mitochondrial ribosomal protein subunit Img2 |
| FQ92HJ001DLL4D | 73 | 8.60E+00 | 17/45 (37%) | Schizosaccharomyces pombe | mok13+ |
| FQ4QJ5301DYJ65 | 76 | 3.80E+00 | 17/47 (36%) | Schizosaccharomyces pombe | RAD3 |
| FQ4QJ5301C2IVM | 74 | 6.60E+00 | 17/47 (36%) | Schizosaccharomyces pombe | RAD3 |
| Contig11601 | 34.3 | 2.90E+00 | 14/37 (37%) | Schizosaccharomyces pombe | unnamed protein product |
| FQ4QJ5301A0Y60 | 81 | 1.00E+00 | 22/64 (34%) | Schizosaccharomyces pombe 972h- | adenylate cyclase |
| FQ4QJ5301DGILJ | 76 | 3.90E+00 | 16/53 (30%) | Schizosaccharomyces pombe 972h- | ATM checkpoint kinase |
| FQ92HJ001BFFTK | 74 | 6.50E+00 | 15/38 (39%) | Schizosaccharomyces pombe 972h- | condensin subunit Cnd1 |
| Contig4971 | 33.1 | 6.50E+00 | 24/84 (28%) | Schizosaccharomyces pombe 972h- | conserved fungal protein |
| FQ92HJ001D3S8D | 79 | 1.70E+00 | 13/29 (44%) | Schizosaccharomyces pombe 972h- | palmitoyltransferase |
| Contig2934 | 52 | 1.00E-05 | 22/30 (73%) | Schizosaccharomyces pombe 972h- | phosphomannomutase Pmm1 |
| FQ92HJ001ES3DL | 131 | 2.00E-06 | 26/52 (50%) | Schizosaccharomyces pombe 972h- | Rho family GTPase Cdc42 |
| FQ92HJ001BLN9M | 73 | 8.40E+00 | 18/56 (32%) | Schizosaccharomyces pombe 972h- | sequence orphan |
| FQ92HJ001BCT3C | 73 | 8.60E+00 | 21/71 (29%) | Schizosaccharomyces pombe 972h- | sequence orphan |
| FQ92HJ001DJSKI | 74 | 6.60E+00 | 15/48 (31%) | Schizosaccharomyces pombe 972h- | sequence orphan |
| FQ92HJ001A5O4Q | 78 | 2.20E+00 | 16/50 (32%) | Schizosaccharomyces pombe 972h- | transcription factor (predicted) |
| FQ92HJ001CK3MA | 74 | 6.40E+00 | 18/46 (39%) | Schizosaccharomyces pombe 972h- | UBA/UAS domain protein Ucp10 |
| FQ4QJ5301CAO9Y | 390 | 1.00E-37 | 70/77 (90%) | Sclerotinia sclerotiorum | phenylalanyl-tRNA synthetase alpha chain |
| Contig3679 | 120 | 2.00E-26 | 58/62 (93%) | Sclerotinia sclerotiorum 1980 | 40S ribosomal protein S9 |
| FQ4QJ5301BKPZ9 | 350 | 6.00E-32 | 67/77 (87%) | Sclerotinia sclerotiorum 1980 | 60S ribosomal protein L17 |
| FQ4QJ5301AOYEG | 362 | 3.00E-33 | 68/76 (89%) | Sclerotinia sclerotiorum 1980 | 60S ribosomal protein L2 |
| Contig11271 | 110 | 2.00E-23 | 54/64 (84%) | Sclerotinia sclerotiorum 1980 | arginyl-tRNA synthetase |
| Contig2098 | 132 | 1.00E-29 | 64/81 (79%) | Sclerotinia sclerotiorum 1980 | conserved hypothetical protein |
| Contig16729 | 144 | 5.00E-33 | 66/76 (86%) | Sclerotinia sclerotiorum 1980 | conserved hypothetical protein |
| FQ4QJ5301DBG2Q | 172 | 3.00E-11 | 33/37 (89%) | Sclerotinia sclerotiorum 1980 | conserved hypothetical protein |
| Contig14744 | 59.3 | 8.00E-08 | 30/76 (39%) | Sclerotinia sclerotiorum 1980 | dynein light chain, cytoplasmic |
| FQ92HJ001AEU9O | 317 | 3.00E-28 | 61/64 (95%) | Sclerotinia sclerotiorum 1980 | eukaryotic initiation factor 4A |
| FQ4QJ5301DKOSG | 241 | 3.00E-19 | 47/53 (88%) | Sclerotinia sclerotiorum 1980 | hypothetical protein SS1G\_00058 |
| Contig2264 | 68.2 | 2.00E-10 | 31/48 (64%) | Sclerotinia sclerotiorum 1980 | hypothetical protein SS1G\_00186 |
| FQ4QJ5301EB2UJ | 269 | 2.00E-22 | 43/64 (67%) | Sclerotinia sclerotiorum 1980 | hypothetical protein SS1G\_00201 |
| FQ92HJ001AZV8X | 280 | 8.00E-24 | 53/61 (86%) | Sclerotinia sclerotiorum 1980 | hypothetical protein SS1G\_00219 |
| FQ92HJ001CZLO5 | 78 | 2.30E+00 | 15/26 (57%) | Sclerotinia sclerotiorum 1980 | hypothetical protein SS1G\_00365 |
| FQ92HJ001D33XP | 74 | 6.60E+00 | 14/27 (51%) | Sclerotinia sclerotiorum 1980 | hypothetical protein SS1G\_00373 |
| FQ4QJ5301AONTW | 210 | 1.00E-15 | 33/62 (53%) | Sclerotinia sclerotiorum 1980 | hypothetical protein SS1G\_00550 |
| FQ4QJ5301CPAXT | 74 | 6.60E+00 | 13/21 (61%) | Sclerotinia sclerotiorum 1980 | hypothetical protein SS1G\_00635 |
| Contig13659 | 37.4 | 3.50E-01 | 30/90 (33%) | Sclerotinia sclerotiorum 1980 | hypothetical protein SS1G\_00697 |
| FQ4QJ5301BJZE5 | 378 | 4.00E-35 | 73/75 (97%) | Sclerotinia sclerotiorum 1980 | hypothetical protein SS1G\_01135 |
| FQ4QJ5301BJIQX | 76 | 3.80E+00 | 18/57 (31%) | Sclerotinia sclerotiorum 1980 | hypothetical protein SS1G\_01177 |
| FQ4QJ5301CTMNU | 337 | 2.00E-30 | 60/80 (75%) | Sclerotinia sclerotiorum 1980 | hypothetical protein SS1G\_01372 |
| Contig123 | 65.9 | 9.00E-10 | 33/55 (60%) | Sclerotinia sclerotiorum 1980 | hypothetical protein SS1G\_01582 |
| Contig217 | 160 | 2.00E-38 | 80/80 (100%) | Sclerotinia sclerotiorum 1980 | hypothetical protein SS1G\_01827 |
| Contig3191 | 33.5 | 5.00E+00 | 15/37 (40%) | Sclerotinia sclerotiorum 1980 | hypothetical protein SS1G\_01982 |
| FQ92HJ001CU3HN | 87 | 2.10E-01 | 17/22 (77%) | Sclerotinia sclerotiorum 1980 | hypothetical protein SS1G\_02258 |
| FQ92HJ001A89LF | 77 | 2.90E+00 | 19/51 (37%) | Sclerotinia sclerotiorum 1980 | hypothetical protein SS1G\_02435 |
| FQ4QJ5301DJRV7 | 117 | 7.00E-05 | 23/43 (53%) | Sclerotinia sclerotiorum 1980 | hypothetical protein SS1G\_02919 |
| FQ4QJ5301C7BH4 | 74 | 6.60E+00 | 15/48 (31%) | Sclerotinia sclerotiorum 1980 | hypothetical protein SS1G\_03133 |
| FQ4QJ5301AXNM9 | 90 | 9.00E-02 | 22/40 (55%) | Sclerotinia sclerotiorum 1980 | hypothetical protein SS1G\_04162 |
| Contig2933 | 34.3 | 2.90E+00 | 16/36 (44%) | Sclerotinia sclerotiorum 1980 | hypothetical protein SS1G\_04282 |
| Contig15491 | 32.7 | 8.50E+00 | 13/35 (37%) | Sclerotinia sclerotiorum 1980 | hypothetical protein SS1G\_04490 |
| FQ4QJ5301DS8WF | 152 | 6.00E-09 | 29/53 (54%) | Sclerotinia sclerotiorum 1980 | hypothetical protein SS1G\_04734 |
| FQ4QJ5301C10CP | 92 | 5.50E-02 | 18/52 (34%) | Sclerotinia sclerotiorum 1980 | hypothetical protein SS1G\_04893 |
| Contig881 | 46.2 | 8.00E-04 | 24/63 (38%) | Sclerotinia sclerotiorum 1980 | hypothetical protein SS1G\_04940 |
| FQ4QJ5301AS0TL | 152 | 6.00E-09 | 26/36 (72%) | Sclerotinia sclerotiorum 1980 | hypothetical protein SS1G\_04940 |
| FQ92HJ001EI873 | 143 | 7.00E-08 | 31/51 (60%) | Sclerotinia sclerotiorum 1980 | hypothetical protein SS1G\_04940 |
| Contig10720 | 48.1 | 2.00E-04 | 23/30 (76%) | Sclerotinia sclerotiorum 1980 | hypothetical protein SS1G\_05226 |
| FQ92HJ001DO25H | 76 | 3.80E+00 | 19/46 (41%) | Sclerotinia sclerotiorum 1980 | hypothetical protein SS1G\_05369 |
| Contig5964 | 114 | 2.00E-24 | 47/77 (61%) | Sclerotinia sclerotiorum 1980 | hypothetical protein SS1G\_05875 |
| Contig15982 | 33.9 | 3.80E+00 | 21/57 (36%) | Sclerotinia sclerotiorum 1980 | hypothetical protein SS1G\_05994 |
| FQ4QJ5301AOR1F | 89 | 1.20E-01 | 16/17 (94%) | Sclerotinia sclerotiorum 1980 | hypothetical protein SS1G\_06434 |
| FQ92HJ001COK99 | 80 | 1.30E+00 | 17/36 (47%) | Sclerotinia sclerotiorum 1980 | hypothetical protein SS1G\_06629 |
| Contig5767 | 34.7 | 2.30E+00 | 15/31 (48%) | Sclerotinia sclerotiorum 1980 | hypothetical protein SS1G\_06923 |
| FQ4QJ5301DWV5O | 126 | 6.00E-06 | 24/40 (60%) | Sclerotinia sclerotiorum 1980 | hypothetical protein SS1G\_07001 |
| FQ92HJ001A77CE | 107 | 1.00E-03 | 21/28 (75%) | Sclerotinia sclerotiorum 1980 | hypothetical protein SS1G\_07008 |
| FQ92HJ001DZ1LJ | 73 | 8.60E+00 | 12/43 (27%) | Sclerotinia sclerotiorum 1980 | hypothetical protein SS1G\_07150 |
| FQ4QJ5301BWP1S | 434 | 1.00E-41 | 80/86 (93%) | Sclerotinia sclerotiorum 1980 | hypothetical protein SS1G\_07581 |
| Contig3339 | 39.3 | 9.10E-02 | 16/24 (66%) | Sclerotinia sclerotiorum 1980 | hypothetical protein SS1G\_07640 |
| FQ92HJ001AJJBN | 79 | 1.70E+00 | 22/47 (46%) | Sclerotinia sclerotiorum 1980 | hypothetical protein SS1G\_07710 |
| FQ92HJ001BLY7G | 85 | 3.40E-01 | 22/44 (50%) | Sclerotinia sclerotiorum 1980 | hypothetical protein SS1G\_07878 |
| FQ92HJ001E37OY | 76 | 3.90E+00 | 14/38 (36%) | Sclerotinia sclerotiorum 1980 | hypothetical protein SS1G\_08639 |
| Contig11817 | 33.1 | 6.60E+00 | 15/33 (45%) | Sclerotinia sclerotiorum 1980 | hypothetical protein SS1G\_08814 |
| Contig5876 | 71.2 | 2.00E-11 | 36/80 (45%) | Sclerotinia sclerotiorum 1980 | hypothetical protein SS1G\_08931 |
| FQ92HJ001CT2ZI | 84 | 4.40E-01 | 15/28 (53%) | Sclerotinia sclerotiorum 1980 | hypothetical protein SS1G\_09232 |
| Contig13602 | 37.4 | 3.40E-01 | 23/50 (46%) | Sclerotinia sclerotiorum 1980 | hypothetical protein SS1G\_09342 |
| FQ4QJ5301CC15R | 214 | 4.00E-16 | 36/48 (75%) | Sclerotinia sclerotiorum 1980 | hypothetical protein SS1G\_09443 |
| Contig9189 | 33.5 | 5.10E+00 | 17/38 (44%) | Sclerotinia sclerotiorum 1980 | hypothetical protein SS1G\_09874 |
| FQ92HJ001EVI6E | 74 | 6.50E+00 | 19/75 (25%) | Sclerotinia sclerotiorum 1980 | hypothetical protein SS1G\_10171 |
| Contig4126 | 28.1 | 2.20E-01 | 14/29 (48%) | Sclerotinia sclerotiorum 1980 | hypothetical protein SS1G\_10405 |
| FQ4QJ5301C92Y8 | 154 | 3.00E-09 | 30/66 (45%) | Sclerotinia sclerotiorum 1980 | hypothetical protein SS1G\_10405 |
| Contig2824 | 79 | 2.00E-15 | 35/50 (70%) | Sclerotinia sclerotiorum 1980 | hypothetical protein SS1G\_10561 |
| Contig4810 | 62 | 1.00E-08 | 30/79 (37%) | Sclerotinia sclerotiorum 1980 | hypothetical protein SS1G\_10572 |
| Contig10782 | 95.5 | 1.00E-18 | 43/48 (89%) | Sclerotinia sclerotiorum 1980 | hypothetical protein SS1G\_10585 |
| Contig756 | 88.2 | 2.00E-16 | 38/49 (77%) | Sclerotinia sclerotiorum 1980 | hypothetical protein SS1G\_10642 |
| Contig14695 | 33.5 | 5.90E+00 | 20/60 (33%) | Sclerotinia sclerotiorum 1980 | hypothetical protein SS1G\_10749 |
| Contig3895 | 107 | 3.00E-22 | 47/63 (74%) | Sclerotinia sclerotiorum 1980 | hypothetical protein SS1G\_10808 |
| FQ4QJ5301C8QAZ | 85 | 3.50E-01 | 28/69 (40%) | Sclerotinia sclerotiorum 1980 | hypothetical protein SS1G\_11039 |
| FQ4QJ5301APTKK | 306 | 3.00E-29 | 57/79 (72%) | Sclerotinia sclerotiorum 1980 | hypothetical protein SS1G\_11406 |
| Contig7901 | 60.1 | 5.00E-08 | 33/76 (43%) | Sclerotinia sclerotiorum 1980 | hypothetical protein SS1G\_11839 |
| Contig11245 | 63.9 | 4.00E-09 | 32/35 (91%) | Sclerotinia sclerotiorum 1980 | hypothetical protein SS1G\_12025 |
| FQ92HJ001A7XPK | 240 | 4.00E-19 | 43/45 (95%) | Sclerotinia sclerotiorum 1980 | hypothetical protein SS1G\_12025 |
| FQ4QJ5301DCGR2 | 86 | 2.70E-01 | 16/39 (41%) | Sclerotinia sclerotiorum 1980 | hypothetical protein SS1G\_12104 |
| FQ92HJ001C2AFC | 186 | 7.00E-13 | 36/55 (65%) | Sclerotinia sclerotiorum 1980 | hypothetical protein SS1G\_12684 |
| FQ4QJ5301CIHSW | 116 | 9.00E-05 | 28/71 (39%) | Sclerotinia sclerotiorum 1980 | hypothetical protein SS1G\_12712 |
| FQ92HJ001DFIKA | 88 | 1.60E-01 | 18/37 (48%) | Sclerotinia sclerotiorum 1980 | hypothetical protein SS1G\_12712 |
| FQ4QJ5301DC99W | 129 | 3.00E-06 | 23/26 (88%) | Sclerotinia sclerotiorum 1980 | hypothetical protein SS1G\_12924 |
| FQ4QJ5301CMYAU | 78 | 2.20E+00 | 13/27 (48%) | Sclerotinia sclerotiorum 1980 | hypothetical protein SS1G\_13024 |
| FQ4QJ5301EATHF | 75 | 5.10E+00 | 19/57 (33%) | Sclerotinia sclerotiorum 1980 | hypothetical protein SS1G\_13097 |
| Contig4331 | 154 | 2.00E-36 | 74/87 (85%) | Sclerotinia sclerotiorum 1980 | hypothetical protein SS1G\_13230 |
| Contig8745 | 64.7 | 2.00E-09 | 40/82 (48%) | Sclerotinia sclerotiorum 1980 | hypothetical protein SS1G\_13609 |
| Contig4707 | 38.1 | 1.10E-02 | 14/24 (58%) | Sclerotinia sclerotiorum 1980 | hypothetical protein SS1G\_14014 |
| Contig4750 | 38.9 | 1.20E-01 | 18/31 (58%) | Sclerotinia sclerotiorum 1980 | hypothetical protein SS1G\_14175 |
| FQ92HJ001CGMU2 | 100 | 6.00E-03 | 20/24 (83%) | Sclerotinia sclerotiorum 1980 | hypothetical protein SS1G\_14216 |
| Contig8259 | 34.3 | 2.90E+00 | 15/27 (55%) | Sclerotinia sclerotiorum 1980 | hypothetical protein SS1G\_14256 |
| Contig4954 | 60.5 | 4.00E-08 | 25/32 (78%) | Sclerotinia sclerotiorum 1980 | hypothetical protein SS1G\_14280 |
| Contig2901 | 32.7 | 8.50E+00 | 12/25 (48%) | Sclerotinia sclerotiorum 1980 | predicted protein |
| Contig10774 | 34.3 | 5.40E+00 | 21/58 (36%) | Sclerotinia sclerotiorum 1980 | predicted protein |
| Contig16753 | 38.5 | 1.60E-01 | 29/80 (36%) | Sclerotinia sclerotiorum 1980 | predicted protein |
| FQ4QJ5301B08EY | 83 | 5.80E-01 | 15/34 (44%) | Sclerotinia sclerotiorum 1980 | predicted protein |
| FQ4QJ5301BEUZ8 | 77 | 2.90E+00 | 23/72 (31%) | Sclerotinia sclerotiorum 1980 | predicted protein |
| FQ4QJ5301BJX8O | 82 | 7.60E-01 | 21/73 (28%) | Sclerotinia sclerotiorum 1980 | predicted protein |
| FQ92HJ001D0UGK | 101 | 5.00E-03 | 22/48 (45%) | Sclerotinia sclerotiorum 1980 | predicted protein |
| Contig9561 | 177 | 2.00E-43 | 88/91 (96%) | Stachybotrys elegans | 60S ribosomal protein L12 |
| Contig1291 | 201 | 2.00E-50 | 100/100 (100%) | Stachybotrys elegans | hypothetical protein |
| Contig15417 | 39.3 | 9.00E-02 | 17/17 (100%) | Stachybotrys elegans | hypothetical protein |
| FQ92HJ001BST7J | 352 | 4.00E-32 | 63/76 (82%) | Tolypocladium inflatum | pathway-specific nitrogen regulator |
| FQ92HJ001EOBK4 | 121 | 2.00E-05 | 23/69 (33%) | Tolypocladium inflatum | putative transposase |
| Contig459 | 38.1 | 2.00E-01 | 24/71 (33%) | Trichophyton rubrum | putative lipase 4 |
| FQ92HJ001A75CK | 295 | 2.00E-25 | 59/59 (100%) | Ustilago maydis 521 | 40S ribosomal protein S9 (S7) |
| Contig12860 | 33.5 | 5.10E+00 | 17/34 (50%) | Ustilago maydis 521 | hypothetical protein UM00105.1 |
| FQ92HJ001CEQ0U | 105 | 2.00E-03 | 19/31 (61%) | Ustilago maydis 521 | hypothetical protein UM00205.1 |
| Contig14220 | 38.1 | 2.00E-01 | 20/57 (35%) | Ustilago maydis 521 | hypothetical protein UM00399.1 |
| FQ92HJ001A3GZO | 80 | 1.30E+00 | 18/42 (42%) | Ustilago maydis 521 | hypothetical protein UM00501.1 |
| Contig13925 | 152 | 6.00E-36 | 70/73 (95%) | Ustilago maydis 521 | hypothetical protein UM00718.1 |
| FQ92HJ001ERF85 | 80 | 1.30E+00 | 18/54 (33%) | Ustilago maydis 521 | hypothetical protein UM01032.1 |
| FQ4QJ5301E8812 | 77 | 2.90E+00 | 24/72 (33%) | Ustilago maydis 521 | hypothetical protein UM01115.1 |
| FQ92HJ001D36ST | 79 | 1.70E+00 | 15/47 (31%) | Ustilago maydis 521 | hypothetical protein UM01256.1 |
| FQ92HJ001E506P | 90 | 7.70E-02 | 16/21 (76%) | Ustilago maydis 521 | hypothetical protein UM01349.1 |
| Contig15640 | 33.5 | 5.10E+00 | 19/48 (39%) | Ustilago maydis 521 | hypothetical protein UM01434.1 |
| FQ92HJ001BF9AV | 134 | 3.00E-12 | 27/29 (93%) | Ustilago maydis 521 | hypothetical protein UM01529.1 |
| FQ92HJ001EISEA | 76 | 3.90E+00 | 14/22 (63%) | Ustilago maydis 521 | hypothetical protein UM01855.1 |
| FQ92HJ001E3E7R | 258 | 3.00E-21 | 53/81 (65%) | Ustilago maydis 521 | hypothetical protein UM01868.1 |
| Contig9347 | 110 | 3.00E-23 | 48/63 (76%) | Ustilago maydis 521 | hypothetical protein UM02034.1 |
| FQ92HJ001CYNVP | 74 | 6.60E+00 | 18/36 (50%) | Ustilago maydis 521 | hypothetical protein UM02455.1 |
| FQ4QJ5301AGS6G | 78 | 2.30E+00 | 16/42 (38%) | Ustilago maydis 521 | hypothetical protein UM02483.1 |
| FQ92HJ001D7ZGI | 118 | 5.00E-05 | 21/26 (80%) | Ustilago maydis 521 | hypothetical protein UM02508.1 |
| FQ92HJ001AU2BM | 225 | 4.00E-26 | 43/50 (86%) | Ustilago maydis 521 | hypothetical protein UM02508.1 |
| Contig8148 | 33.9 | 3.80E+00 | 20/51 (39%) | Ustilago maydis 521 | hypothetical protein UM02545.1 |
| FQ4QJ5301C0AM4 | 93 | 4.10E-02 | 16/44 (36%) | Ustilago maydis 521 | hypothetical protein UM02574.1 |
| FQ92HJ001A0RDT | 277 | 2.00E-23 | 49/65 (75%) | Ustilago maydis 521 | hypothetical protein UM02581.1 |
| FQ4QJ5301ASKJ8 | 77 | 2.90E+00 | 18/64 (28%) | Ustilago maydis 521 | hypothetical protein UM02795.1 |
| FQ92HJ001CLXEN | 179 | 4.00E-12 | 33/49 (67%) | Ustilago maydis 521 | hypothetical protein UM02959.1 |
| FQ92HJ001ESG25 | 148 | 2.00E-08 | 35/71 (49%) | Ustilago maydis 521 | hypothetical protein UM03005.1 |
| FQ92HJ001D9ZDC | 310 | 3.00E-27 | 59/70 (84%) | Ustilago maydis 521 | hypothetical protein UM03103.1 |
| Contig2902 | 34.7 | 2.30E+00 | 23/67 (34%) | Ustilago maydis 521 | hypothetical protein UM03341.1 |
| Contig10910 | 35.4 | 1.30E+00 | 14/43 (32%) | Ustilago maydis 521 | hypothetical protein UM03426.1 |
| FQ4QJ5301AZISP | 79 | 1.70E+00 | 18/39 (46%) | Ustilago maydis 521 | hypothetical protein UM03787.1 |
| FQ92HJ001CS7X8 | 282 | 5.00E-24 | 51/81 (62%) | Ustilago maydis 521 | hypothetical protein UM03846.1 |
| Contig13969 | 63.9 | 3.00E-09 | 29/57 (50%) | Ustilago maydis 521 | hypothetical protein UM03999.1 |
| FQ92HJ001BYYJW | 90 | 9.20E-02 | 22/62 (35%) | Ustilago maydis 521 | hypothetical protein UM04477.1 |
| Contig10457 | 34.7 | 5.60E+00 | 16/34 (47%) | Ustilago maydis 521 | hypothetical protein UM04582.1 |
| FQ92HJ001EEVZO | 76 | 3.80E+00 | 19/61 (31%) | Ustilago maydis 521 | hypothetical protein UM04694.1 |
| FQ92HJ001EP345 | 194 | 8.00E-14 | 37/39 (94%) | Ustilago maydis 521 | hypothetical protein UM05062.1 |
| FQ92HJ001A5VDK | 151 | 8.00E-09 | 36/67 (53%) | Ustilago maydis 521 | hypothetical protein UM05140.1 |
| FQ92HJ001DD565 | 273 | 6.00E-23 | 45/73 (61%) | Ustilago maydis 521 | hypothetical protein UM05189.1 |
| FQ92HJ001CRGTU | 244 | 1.00E-19 | 41/56 (73%) | Ustilago maydis 521 | hypothetical protein UM05222.1 |
| Contig11675 | 38.9 | 1.20E-01 | 20/32 (62%) | Ustilago maydis 521 | hypothetical protein UM05244.1 |
| Contig2951 | 38.1 | 2.00E-01 | 20/61 (32%) | Ustilago maydis 521 | hypothetical protein UM05837.1 |
| Contig4428 | 32.7 | 8.40E+00 | 22/63 (34%) | Ustilago maydis 521 | hypothetical protein UM06065.1 |
| Contig16807 | 41.6 | 1.90E-02 | 21/34 (61%) | Ustilago maydis 521 | hypothetical protein UM06147.1 |
| Contig16832 | 33.5 | 5.00E+00 | 14/33 (42%) | Ustilago maydis 521 | hypothetical protein UM06359.1 |
| FQ92HJ001CKQLQ | 73 | 8.40E+00 | 15/44 (34%) | Vanderwaltozyma polyspora DSM | hypothetical protein Kpol\_1010p69 |
| Contig12112 | 33.5 | 5.70E+00 | 15/42 (35%) | Vanderwaltozyma polyspora DSM | hypothetical protein Kpol\_1013p12 |
| Contig16397 | 37 | 4.50E-01 | 18/43 (41%) | Vanderwaltozyma polyspora DSM | hypothetical protein Kpol\_1018p106 |
| FQ92HJ001C3CL5 | 78 | 2.30E+00 | 19/40 (47%) | Vanderwaltozyma polyspora DSM | hypothetical protein Kpol\_1023p99 |
| Contig8671 | 33.9 | 5.00E+00 | 14/37 (37%) | Vanderwaltozyma polyspora DSM | hypothetical protein Kpol\_1028p96 |
| Contig16463 | 34.7 | 2.40E+00 | 19/69 (27%) | Vanderwaltozyma polyspora DSM | hypothetical protein Kpol\_1030p4 |
| Contig9601 | 38.9 | 1.20E-01 | 29/114 (25%) | Vanderwaltozyma polyspora DSM | hypothetical protein Kpol\_1031p60 |
| Contig9187 | 33.9 | 3.80E+00 | 15/60 (25%) | Vanderwaltozyma polyspora DSM | hypothetical protein Kpol\_1033p15 |
| FQ4QJ5301DT3FK | 75 | 5.00E+00 | 20/60 (33%) | Vanderwaltozyma polyspora DSM | hypothetical protein Kpol\_1033p33 |
| Contig12156 | 34.3 | 2.90E+00 | 17/37 (45%) | Vanderwaltozyma polyspora DSM | hypothetical protein Kpol\_1036p73 |
| Contig14108 | 38.5 | 1.50E-01 | 14/36 (38%) | Vanderwaltozyma polyspora DSM | hypothetical protein Kpol\_1036p78 |
| Contig14901 | 34.3 | 3.00E+00 | 16/52 (30%) | Vanderwaltozyma polyspora DSM | hypothetical protein Kpol\_1037p9 |
| FQ92HJ001D6ROQ | 74 | 6.60E+00 | 19/56 (33%) | Vanderwaltozyma polyspora DSM | hypothetical protein Kpol\_1041p45 |
| FQ92HJ001DZ3TU | 351 | 5.00E-32 | 66/68 (97%) | Vanderwaltozyma polyspora DSM | hypothetical protein Kpol\_1043p69 |
| FQ92HJ001BSGCO | 74 | 6.60E+00 | 16/50 (32%) | Vanderwaltozyma polyspora DSM | hypothetical protein Kpol\_1050p88 |
| Contig12245 | 36.2 | 7.80E-01 | 25/82 (30%) | Vanderwaltozyma polyspora DSM | hypothetical protein Kpol\_1052p42 |
| Contig11861 | 32.7 | 8.60E+00 | 13/54 (24%) | Vanderwaltozyma polyspora DSM | hypothetical protein Kpol\_1053p38 |
| FQ92HJ001DF1SV | 75 | 5.00E+00 | 13/28 (46%) | Vanderwaltozyma polyspora DSM | hypothetical protein Kpol\_1053p42 |
| FQ92HJ001BK26M | 80 | 1.30E+00 | 17/42 (40%) | Vanderwaltozyma polyspora DSM | hypothetical protein Kpol\_1054p45 |
| FQ92HJ001BW6FA | 73 | 8.60E+00 | 21/60 (35%) | Vanderwaltozyma polyspora DSM | hypothetical protein Kpol\_1055p38 |
| Contig3111 | 39.3 | 1.40E-01 | 29/104 (27%) | Vanderwaltozyma polyspora DSM | hypothetical protein Kpol\_1056p8 |
| Contig5023 | 33.1 | 6.40E+00 | 18/67 (26%) | Vanderwaltozyma polyspora DSM | hypothetical protein Kpol\_1065p13 |
| Contig12851 | 33.9 | 3.80E+00 | 13/44 (29%) | Vanderwaltozyma polyspora DSM | hypothetical protein Kpol\_1066p46 |
| FQ92HJ001DM33A | 388 | 3.00E-36 | 78/78 (100%) | Vanderwaltozyma polyspora DSM | hypothetical protein Kpol\_1070p6 |
| Contig10921 | 33.5 | 5.00E+00 | 15/53 (28%) | Vanderwaltozyma polyspora DSM | hypothetical protein Kpol\_2001p2 |
| Contig15974 | 35.8 | 1.00E+00 | 20/77 (25%) | Vanderwaltozyma polyspora DSM | hypothetical protein Kpol\_2001p2 |
| FQ92HJ001DAMRX | 76 | 3.80E+00 | 17/46 (36%) | Vanderwaltozyma polyspora DSM | hypothetical protein Kpol\_2001p51 |
| Contig12096 | 33.1 | 6.60E+00 | 16/45 (35%) | Vanderwaltozyma polyspora DSM | hypothetical protein Kpol\_264p3 |
| FQ92HJ001EBVJ5 | 83 | 6.00E-01 | 22/54 (40%) | Vanderwaltozyma polyspora DSM | hypothetical protein Kpol\_298p1 |
| Contig10436 | 33.1 | 6.60E+00 | 19/56 (33%) | Vanderwaltozyma polyspora DSM | hypothetical protein Kpol\_303p2 |
| FQ4QJ5301ERGEP | 73 | 8.40E+00 | 15/41 (36%) | Vanderwaltozyma polyspora DSM | hypothetical protein Kpol\_316p8 |
| FQ4QJ5301AKAY2 | 77 | 3.00E+00 | 20/61 (32%) | Vanderwaltozyma polyspora DSM | hypothetical protein Kpol\_385p13 |
| FQ92HJ001E4Y0C | 81 | 1.00E+00 | 11/40 (27%) | Vanderwaltozyma polyspora DSM | hypothetical protein Kpol\_440p5 |
| FQ92HJ001DB4WJ | 78 | 2.20E+00 | 21/59 (35%) | Vanderwaltozyma polyspora DSM | hypothetical protein Kpol\_480p25 |
| FQ92HJ001ATYSM | 73 | 8.70E+00 | 23/51 (45%) | Vanderwaltozyma polyspora DSM | hypothetical protein Kpol\_495p22 |
| FQ92HJ001EOHOE | 80 | 1.30E+00 | 17/46 (36%) | Vanderwaltozyma polyspora DSM | hypothetical protein Kpol\_526p51 |
| Contig4557 | 33.1 | 6.50E+00 | 14/48 (29%) | Vanderwaltozyma polyspora DSM | hypothetical protein Kpol\_534p4 |
| FQ92HJ001CQ59P | 80 | 1.30E+00 | 20/55 (36%) | Vanderwaltozyma polyspora DSM | hypothetical protein Kpol\_534p6 |
| Contig1482 | 35.4 | 1.30E+00 | 20/56 (35%) | Vanderwaltozyma polyspora DSM | hypothetical protein Kpol\_541p5 |
| FQ4QJ5301D0RTD | 80 | 1.30E+00 | 16/41 (39%) | Vanderwaltozyma polyspora DSM 70294 | Tkp1 protein |
| Contig9984 | 35 | 1.70E+00 | 14/21 (66%) | Vanhornia eucnemidarum | ATP synthase F0 subunit 6 |
| Contig66 | 34.7 | 2.20E+00 | 17/54 (31%) | Yarrowia lipolytica | hypothetical protein |
| Contig7650 | 55.1 | 2.00E-06 | 26/45 (57%) | Yarrowia lipolytica | hypothetical protein |
| Contig10856 | 37.4 | 3.50E-01 | 20/53 (37%) | Yarrowia lipolytica | hypothetical protein |
| Contig12354 | 36.2 | 8.10E-01 | 18/69 (26%) | Yarrowia lipolytica | hypothetical protein |
| Contig13904 | 33.1 | 6.60E+00 | 14/30 (46%) | Yarrowia lipolytica | hypothetical protein |
| Contig14322 | 34.7 | 2.20E+00 | 18/55 (32%) | Yarrowia lipolytica | hypothetical protein |
| Contig14388 | 33.9 | 3.90E+00 | 19/46 (41%) | Yarrowia lipolytica | hypothetical protein |
| Contig14497 | 44.3 | 3.00E-03 | 28/84 (33%) | Yarrowia lipolytica | hypothetical protein |
| Contig15493 | 36.2 | 1.20E+00 | 19/52 (36%) | Yarrowia lipolytica | hypothetical protein |
| Contig15938 | 34.3 | 2.90E+00 | 20/70 (28%) | Yarrowia lipolytica | hypothetical protein |
| FQ4QJ5301EAKOZ | 100 | 6.00E-03 | 31/76 (40%) | Yarrowia lipolytica | hypothetical protein |
| FQ4QJ5301EIL3R | 73 | 8.70E+00 | 17/46 (36%) | Yarrowia lipolytica | hypothetical protein |
| FQ4QJ5301ECZV8 | 78 | 2.20E+00 | 17/50 (34%) | Yarrowia lipolytica | hypothetical protein |
| FQ4QJ5301BTQWR | 136 | 4.00E-07 | 27/29 (93%) | Yarrowia lipolytica | hypothetical protein |
| FQ4QJ5301AHS9V | 93 | 4.10E-02 | 18/51 (35%) | Yarrowia lipolytica | hypothetical protein |
| FQ4QJ5301DS0N4 | 100 | 8.00E-06 | 17/30 (56%) | Yarrowia lipolytica | hypothetical protein |
| FQ4QJ5301B6HFT | 80 | 1.30E+00 | 18/57 (31%) | Yarrowia lipolytica | hypothetical protein |
| FQ92HJ001ENZ7H | 76 | 3.80E+00 | 25/72 (34%) | Yarrowia lipolytica | hypothetical protein |
| FQ92HJ001EGC20 | 80 | 1.30E+00 | 16/45 (35%) | Yarrowia lipolytica | hypothetical protein |
| FQ92HJ001BKUFZ | 74 | 6.60E+00 | 14/32 (43%) | Yarrowia lipolytica | hypothetical protein |
| FQ92HJ001AU05F | 79 | 1.70E+00 | 22/62 (35%) | Yarrowia lipolytica | hypothetical protein |
| FQ92HJ001DFS2U | 97 | 1.40E-02 | 18/49 (36%) | Yarrowia lipolytica | hypothetical protein |
| FQ92HJ001B6B7X | 82 | 7.70E-01 | 21/63 (33%) | Yarrowia lipolytica | hypothetical protein |
| FQ92HJ001BHXH1 | 74 | 6.60E+00 | 14/46 (30%) | Yarrowia lipolytica | hypothetical protein |
| FQ92HJ001D3T9W | 118 | 6.00E-11 | 23/43 (53%) | Yarrowia lipolytica | hypothetical protein |
| FQ92HJ001CBV2S | 73 | 8.70E+00 | 15/48 (31%) | Yarrowia lipolytica | hypothetical protein |
| FQ92HJ001CJIKW | 79 | 1.70E+00 | 13/42 (30%) | Yarrowia lipolytica | hypothetical protein |
| FQ92HJ001DUEQD | 80 | 1.30E+00 | 19/53 (35%) | Yarrowia lipolytica | hypothetical protein |
| FQ92HJ001B0RZG | 78 | 2.20E+00 | 20/59 (33%) | Yarrowia lipolytica | hypothetical protein |
| FQ92HJ001E09HZ | 84 | 4.50E-01 | 17/47 (36%) | Yarrowia lipolytica | hypothetical protein |
| FQ92HJ001ER9PU | 75 | 5.00E+00 | 13/44 (29%) | Yarrowia lipolytica | YlSLA2 |
|  |  |  |  |  |  |
