# Supplementary material for: Massively parallel pyrosequencing-based transcriptome analyses of small brown planthopper (Laodelphax striatellus), a vector insect transmitting rice stripe virus (RSV)
Source: BMC Genomics. 2010 May 13;11:303. doi: 10.1186/1471-2164-11-303 (PMC2885366; doi:10.1186/1471-2164-11-303)
Supplement: Additional file 4 — L. striatellus genes abundantly transcribed in the viruliferous sample. The table provides a list of top 30 genes that highly expressed in the sample of viruliferous insects. [file 1471-2164-11-303-S4.HTM]

Additional file 4


**Additional file
4.** *L. striatellus*genes that are abundantly
transcribed in the viruliferous sample

 

|  |  |  |  |  |  |  |  |
| --- | --- | --- | --- | --- | --- | --- | --- |
| **Contigs** | **Reads in viruliferous sample** | **Reads in naïve sample** | **Ratio of abundance** | **Description** | **Organism** | **Score**a | **e-**  **value** |
| Contig3703 | 2954 | 7 | 422.0 | Vitellogenin | *Nilaparvata lugens* | 919 | 0 |
| Contig4872 | 422 | 1 | 422.0 | Phage tail fiber repeat family protein | *Tetrahymena thermophila* | 45.4 | 0.013 |
| Contig2252 | 1265 | 6 | 210.8 | Vitellogenin | *Nilaparvata lugens* | 568 | 8e-160 |
| Contig8285 | 136 | 1 | 136.0 | Vitellogenin | *Nilaparvata lugens* | 266 | 2e-69 |
| Contig4916 | 134 | 1 | 134.0 | Facilitative hexose transporter 1 | *Nilaparvata lugens* | 223 | 1e-56 |
| Contig2944 | 221 | 2 | 110.5 | Vitellogenin | *Nilaparvata lugens* | 139 | 2e-31 |
| Contig6344 | 109 | 1 | 109.0 | Vitellogenin | *Nilaparvata lugens* | 189 | 1e-46 |
| Contig6671 | 108 | 1 | 108.0 | Proteinase | *Periplaneta americana* | 352 | 3e-95 |
| Contig2802 | 391 | 4 | 97.8 | Elongation factor 1 alpha | *Culicoides sonorensis* | 87.4 | 3e-16 |
| Contig3 | 853 | 11 | 77.5 | PREDICTED: similar to GA15266-PA | *Nasonia vitripennis* | 50.4 | 1e-4 |
| Contig75 | 75 | 1 | 75.0 | AF281654\_1 mRNA cap-binding protein eIF4E | *Spodoptera frugiperda* | 89.4 | 8e-17 |
| Contig3464 | 72 | 1 | 72.0 | No match | *-* | - | - |
| Contig1675 | 279 | 4 | 69.8 | PREDICTED: similar to CG3523-PA | *Tribolium castaneum* | 195 | 1e-48 |
| Contig6073 | 66 | 1 | 66.0 | Hypothetical protein CE2916 | *Corynebacterium efficiens* | 706 | 0.037 |
| Contig7165 | 65 | 1 | 65.0 | Similar to CG3320-PA | *Tribolium castaneum* | 415 | 4e-65 |
| Contig4299 | 509 | 0 | ND | CG34333 CG34333-PA | *Drosophila melanogaster* | 60.8 | 1e-7 |
| Contig1465 | 436 | 0 | ND | PREDICTED: hypothetical protein | *Nasonia vitripennis* | 44.3 | 0.007 |
| Contig362 | 380 | 0 | ND | Vitellogenin | *Nilaparvata lugens* | 379 | 1e-103 |
| Contig6985 | 300 | 0 | ND | Transferrin | *Romalea microptera* | 155 | 3e-036 |
| Contig2639 | 267 | 0 | ND | Hepatopancreas kazal-type proteinase inhibitor | *Penaeus monodon* | 47 | 0.002 |
| Contig2732 | 225 | 0 | ND | Putative enolase | *Anopheles gambiae* | 209 | 1e-052 |
| Contig4555 | 185 | 0 | ND | Vitellogenin | *Nilaparvata lugens* | 128 | 2e-028 |
| Contig1019 | 126 | 0 | ND | Vitellogenin | *Nilaparvata lugens* | 173 | 5e-042 |
| Contig7192 | 123 | 0 | ND | No match | *-* | - | - |
| Contig8372 | 119 | 0 | ND | Putative paramyosin | *Tribolium castaneum* | 246 | 1e-063 |
| Contig5108 | 110 | 0 | ND | Vitellogenin | *Nilaparvata lugens* | 44.3 | 0.004 |
| Contig312 | 99 | 0 | ND | Hypothetical protein | *Paramecium tetraurelia* | 819 | 0.24 |
| Contig3771 | 92 | 0 | ND | Unnamed protein product | *Tetraodon nigroviridis* | 376 | 2.9 |
| Contig8349 | 84 | 0 | ND | Similar to CG12070-PA | *Tribolium* | 1259 | 1e-69 |
| Contig5841 | 73 | 0 | ND | Cuticle protein | *-* | 498 | 9e-16 |

a. The score and e-value are results from the BlastX
search.
