# Supplementary material for: Massively parallel pyrosequencing-based transcriptome analyses of small brown planthopper (Laodelphax striatellus), a vector insect transmitting rice stripe virus (RSV)
Source: BMC Genomics. 2010 May 13;11:303. doi: 10.1186/1471-2164-11-303 (PMC2885366; doi:10.1186/1471-2164-11-303)
Supplement: Additional file 5 — List of genes selected for RT-PCR assay. This table shows the general feature of genes selected for RT-PCR analysis. [file 1471-2164-11-303-S5.HTM]

¡¡¡¡

| **Additional file 5. List of unigenes that amplified by RT-PCR** | | | |
|  |  |  |  |
| **Unigene** | **Organism** | **E-value** | **Description** |
| Contig4916 | *Nilaparvata lugens* | 4.00E-56 | facilitative hexose transporter 1 |
| Contig7165 | *Tribolium castaneum* | 4.00E-65 | PREDICTED: similar to CG3320-PA, isoform A |
| Contig6344 | *Nilaparvata lugens* | 1.00E-46 | vitellogenin |
| Contig6671 | *Periplaneta americana* | 3.00E-95 | homologue of Sarcophaga 26,29kDa proteinase |
| Contig2639 | *Penaeus monodon* | 2.00E-03 | hepatopancreas kazal-type proteinase inhibitor |
| Contig2252 | *Nilaparvata lugens* | 1.00E-160 | vitellogenin |
| Contig8285 | *Nilaparvata lugens* | 1.00E-120 | vitellogenin |
| Contig14453 | *Nematostella* | 3.90E+00 | hypothetical protein NEMVEDRAFT\_v1g153015 |
| Contig3703 | *Nilaparvata lugens* | 0.00E+00 | vitellogenin |
| Contig2732 | *Anopheles gambiae* | 1.00E-50 | AGAP007827-PA |
| Contig4872 | *Tetrahymena thermophila* | 1.80E-02 | Phage tail fiber repeat family protein |
| Contig362 | *Nilaparvata lugens* | 1.00E-103 | vitellogenin |
| Contig8266 | *Anoplophora glabripennis* | 3.00E-82 | muscle protein 20-like protein |
| Contig5603 | *Brevicoryne brassicae picorna-like virus* | 1.00E-110 | polyprotein |
| Contig1981 | *Graphocephala atropunctata* | 1.00E-146 | putative ADP/ATP translocase |
| Contig12404 | *Himetobi P virus* | 0.00E+00 | nonstructural protein precursor |
| Contig4009 | *Homalodisca coagulata* | 0.00E+00 | putative muscle actin |
| Contig13131 |  | 1.00E-26 | PREDICTED: similar to mitochondrial F1F0-ATP synthase subunit c |
| Contig9432 |  | 1.00E-95 | PREDICTED: similar to Probable cytochrome P450 6a13 (CYPVIA13) |
| Contig986 | *Apis mellifera* | 2.00E-15 | PREDICTED: similar to Lk6 CG17342-PA, isoform A |
| Contig3086 | *Riptortus clavatus* | 1.00E-128 | transferrin |
| Contig1243 |  | 1.00E-173 | PREDICTED: similar to Guanine nucleotide-binding protein beta |
| Contig14330 | *Tribolium* | 2.00E-66 | PREDICTED: similar to 40S ribosomal protein S15Aa |
| Contig1253 | *Nilaparvata lugens* | 0.00E+00 | AF302777\_1 carboxylesterase precursor |
| Contig6661 |  | 0.00E+00 | TBB1\_MANSE Tubulin beta-1 chain (Beta-1-tubulin) |
| Contig2551 | *Apis* | 1.00E-179 | PREDICTED: similar to Ribosomal protein L4 CG5502-PA isoform 1 |
| Contig13253 |  | 9.00E-53 | PREDICTED: similar to 60S acidic ribosomal protein P0 |
| Contig2413 |  | 0.00E+00 | putative mitochondrial ATP synthase alpha subunit precursor |
| Contig4917 |  | 1.00E-110 | RS6\_SPOFR 40S ribosomal protein S6 |
| Contig4577 | *Rhodnius prolixus* | 2.00E-26 | odorant-binding protein precursor |
| Contig8344 | *Philaethria dido* | 2.00E-43 | cytochrome oxidase subunit 2 |
| Contig4825 | *Maconellicoccus* | 5.00E-48 | lethal(2)essential for life protein-like protein |
| Contig7899 | *Laodelphax striatellus* | 0.00E+00 | alpha 2-tubulin |
| Contig7591 | *Nasonia vitripennis* | 0.00E+00 | PREDICTED: similar to malic enzyme |
| Contig5174 | *Bombyx mori* | 1.00E-119 | H+ transporting ATP synthase beta subunit isoform 2 |
| Contig2280 | *Aedes aegypti* | 0.00E+00 | ATP-citrate synthase |
| Contig16345 | *Diaphorina citri* | 1.00E-104 | putative S5e ribosomal protein |
| Contig12854 | *Periplaneta americana* | 3.00E-48 | MPA13 allergen |
| Contig2802 | *Culicoides sonorensis* | 1.00E-114 | elongation factor 1 alpha |
| Contig14832 |  | 1.70E+00 | hypothetical protein |
| Contig8372 | *Tribolium castaneum* | 1.00E-61 | PREDICTED: similar to CG5939-PA, isoform A |
| Contig5503 | *Tribolium* | 1.00E-92 | PREDICTED: similar to 60S ribosomal protein L15 |
| Contig6985 |  | 2.00E-37 | TRF\_BLADI Transferrin precursor |
| Contig11752 | *Rattus norvegicus* | 3.00E-43 | hypothetical protein LOC310926 |
| Contig2248 | *Periplaneta americana* | 0.00E+00 | Elongation factor-2 |
|  |  |  |  |
