# Supplementary material for: Massively parallel pyrosequencing-based transcriptome analyses of small brown planthopper (Laodelphax striatellus), a vector insect transmitting rice stripe virus (RSV)
Source: BMC Genomics. 2010 May 13;11:303. doi: 10.1186/1471-2164-11-303 (PMC2885366; doi:10.1186/1471-2164-11-303)
Supplement: Additional file 6 — Sequences of primers used in RT-PCR profiling. [file 1471-2164-11-303-S6.PDF]

Additional file 6. Primers used for RT-PCR expression profiling of putative transcripts generated from 454-pyrosequencing.

Targeted contigs	Sequence of the primer pairs
Contig12404	5’-TTGACCAGGGCGATAGAT-3’
	5’-TTCGGTTGAGAAACTTGATT-3’
Contig4009	5’-TCACCAACTGGGACGACA-3’
	5’-GTGGGGCAAGGCATAACC-3’
Contig13131	5’-CCCACCGCTTTCACTACT-3’
	5’-ATTCGCTACCAACCCTTT-3’
Contig3703	5’-ATTGGGCGAATACTGAAG-3’
	5’-AGAGGTGAAGTGCGTGGT-3’
Contig16345	5’-GCGTGACTGGTTGTAGGT-3’
	5’-CATCATAAGCGAGTTGGT-3’
Contig13611	5’-TTGACAAGGATGAAGTGC-3’
	5’-AGAAGACCAACAAGACCG-3’
Contig12854	5’-ATGAGCGACTTCACCTTG-3’
	5’-CTATCCCCACCCCACTAC-3’
Contig1981	5’-AGTTCCGCTCAAGTCTGC-3’
	5’-TTTCACTCGCTCAATAGG-3’
Contig10993	5’-ATCCGACTATTCCACAAA-3’
	5’-AAGGGTCAAATGAAGAAA-3’
Contig7667	5’-AGCGATTCGTCGGTTAGT-3’
	5’-GCCAAGCAGACCTTTCAG-3’
Contig14330	5’-CGGTATCCGATTGTCCTT-3’
	5’-TGTTTTCGCCTCGCCTCT-3’
Contig362	5’-GTGGGGAATGCTACTTT-3’
	5’-CCAATGATGGAGGATACA-3’
Contig6985	5’-CTGTTCCCACGCCTCAT-3’
	5’-CCTTGGTGCGAATCTCC-3’
Contig2732	5’-ACCCATTTGACCAGGAA-3’
	5’-AAACGCCCAGAATAGCA-3’
Contig8372	5’-ACTGTCTGCTGCTTACAA-3’
	5’-CACCTCGGTCTTCAACT-3’
Contig4872	5’-TCCGACGACTACACGCTA-3’
	5’-TGGCAACGCTCTACCCT-3’
Contig2252	5’-GGTGGTCAAGTCAAGGA-3’
	5’-GAGACAAGGGTGGCATT-3’
Contig5603	5’-GCGTCGCCTGATACCTA-3’
	5’-ACATCCAACCCGTCCAT-3’
Contig8285	5’-AGAAGGGTGATGTGAGG-3’
	5’-CAATGATGATGGTGGAG-3’
Contig4916	5’-TCATAAGCCAAGCGATAA-3’
	5’-CACTCACTGCCACCATT-3’

Contig6344	5’-GACAGCAGCGAGGAAAA-3’
	5’-ATAGTGAAGGAGGTGAGTG-3’
Contig6671	5’-TTCTATCGGTCAAATGG-3’
	5’-ACTTCTTGGCTCACATTA-3’
Contig2802	5’-TGTCTTCCTCTTTCTTCCGTG-3’
	5’-CCCTTGCCCATTTCCTG-3’
Contig7165	5’-TTCCGAACCATCACATC-3’
	5’-TCCTTAGCCTGGGTGTA-3’
Contig1243	5’-TGGAAACTGAAGAAGGA-3’
	5’-TGTTATCAACGGAGAAAG-3’
Contig986	5’-ACGCCGAACGACCGCTTAC-3’
	5’-CACCGCCATTGCCGACT-3’
Contig2248	5’-CTTCGTTGGCAGTCATT-3’
	5’-CCTTGTCACGCTGGTCT-3’
Contig13233	5’-TATTGTGACCCTGAGATG-3’
	5’-TAGATAAGAACCGACCTG-3’
Contig14453	5’-CGGTATCCGACTTTCTC-3’
	5’-TGTAGTTGCACTCACTTCTT-3’
Contig14832	5’-GTCGTGGTTTCCGTCTA-3’
	5’-AGTGTCAGTCAGCCTTGT-3’
Contig11752	5’-ATTTGCCAAGTATGTTTC-3’
	5’-GTGAGTTTTCCCGTGTT-3’
Contig2639	5’-GGATACATTTCAAGCGACA-3’
	5’-CTCCACGGCCACCTACT-3’
Contig5108	5’-TTGTATGGGTAGTAGTAGGG-3’
	5’-AGCAGAAAACCAAGTCC-3’
Contig1253	5’-ACATCAGCAGAGGGAGC-3’
	5’-AATCAACCATCTTAGCG-3’
Contig6661	5’-GTGGCGGACATCTTGAG-3’
	5’-GCCGATCTGCGTAAACT-3’
Contig2551	5’-TGGGTTCCTCCATTTCC-3’
	5’-ATGCGTCCTCCCCTACA-3’
Contig13253	5’-CTTTCGCTCGCACTTTG-3’
	5’-GGTTGCCGATTTCCTTC-3’
Contig2413	5’-CTTTGCGAGATTCCTAT-3’
	5’-TCCTTGATGTGCTTGTC-3’
Contig4917	5’-CCTTTTCCTTCCTATCG-3’
	5’-GCACTGTTGTATCGGTCA-3’
Contig4577	5’-GGTAGCCAACACCAAGG-3’
	5’-TTCCCAATCCAGTATAGACAC-3’
Contig8344	5’-CTACCCGACGCATACAG-3’
	5’-GGATGGCAATAAGGAAA-3’
Contig4825	5’-CGACGAGTCTGACGCTA-3’
	5’-TGCCTGGAAATGAAACC-3’

Contig7899	5’-TGCCCTCCGACAAAACT-3’
	5’-ATGCGGTCCAGCACAAT-3’
Contig5243	5’-ACATTCATTACTGGCTTGG-3’
	5’-TTGATGGCTTTCACTTT-3’
Contig7591	5’-ACAAGCACTGGGCATCC-3’
	5’-CACCGTCAGTCACCACAA-3’
Contig5174	5’-TGGAATACGACCCAGCAG-3’
	5’-GGACCCGTGAAGGCAAC-3’
Contig2280	5’-GCAGCCAGAGTTCAGTG-3’
	5’-ATACCTTCGGCGATAAT-3’
Contig5479	5’-TGGGAGTTGGGATTTTG-3’
	5’-CGAGCGAGTTGAGGATG-3’
Contig8266	5’-CATACAGGATGGCTTTG-3’
	5’-CTTTCTTCTCCCACAGG-3’
Contig9432	5’-TCTAATCAAGAAAGGAACG-3’
	5’-TTGAAATAACCAGCGTC-3’
Contig3086	5’-AACGCCTCAAGAGCAAG-3’
	5’-TTAAGAGCCAGCACGGT-3’
Contig5503	5’-AGTTTTGCCTTTTCCGTTCG-3’
	5’-TGGCTTTGTCAGGTCTGGTTG-3’
